# Probing Anti-inflammatory Properties Independent of NF-κB Through Conformational Constraint of Peptide-Based Interleukin-1 Receptor Biased Ligands

**DOI:** 10.3389/fchem.2019.00023

**Published:** 2019-02-13

**Authors:** Azade Geranurimi, Colin W. H. Cheng, Christiane Quiniou, Tang Zhu, Xin Hou, José Carlos Rivera, Daniel J. St-Cyr, Kim Beauregard, Vadim Bernard-Gauthier, Sylvain Chemtob, William D. Lubell

**Affiliations:** ^1^Département de Chimie, Université de Montréal, Montréal, QC, Canada; ^2^Department of Pharmacology and Therapeutics, McGill University, Montréal, QC, Canada; ^3^CHU Sainte-Justine Research Centre, Montréal, QC, Canada; ^4^Hôpital Maisonneuve-Rosemont Research Centre, Montréal, QC, Canada; ^5^Departments of Pediatrics, Pharmacology and Physiology, and Ophthalmology, Université de Montréal, Montréal, QC, Canada

**Keywords:** β-hydroxy-α-amino-γ-lactam (Hgl), interleukin-1 receptor antagonist, 101.10 (rytvela), β-turn, Freidinger lactams, solid-phase peptide synthesis, retinopathy, prematurity

## Abstract

Interleukin-1β (IL-1β) binds to the IL-1 receptor (IL-1R) and is a key cytokine mediator of inflammasome activation. IL-1β signaling leads to parturition in preterm birth (PTB) and contributes to the retinal vaso-obliteration characteristic of oxygen-induced retinopathy (OIR) of premature infants. Therapeutics targeting IL-1β and IL-1R are approved to treat rheumatoid arthritis; however, all are large proteins with clinical limitations including immunosuppression, due in part to inhibition of NF-κB signaling, which is required for immuno-vigilance and cytoprotection. The all-D-amino acid peptide **1** (101.10, H-d-Arg-d-Tyr-d-Thr-d-Val-d-Glu-d-Leu-d-Ala-NH_2_) is an allosteric IL-1R modulator, which exhibits functional selectivity and conserves NF-κB signaling while inhibiting other IL-1-activated pathways. Peptide **1** has proven effective in experimental models of PTB and OIR. Seeking understanding of the structural requirements for the activity and biased signaling of **1**, a panel of twelve derivatives was synthesized employing the various stereochemical isomers of α-amino-γ-lactam (Agl) and α-amino-β-hydroxy-γ-lactam (Hgl) residues to constrain the D-Thr-D-Val dipeptide residue. Using circular dichroism spectroscopy, the peptide conformation in solution was observed to be contingent on Agl, Hgl, and Val stereochemistry. Moreover, the lactam mimic structure and configuration influenced biased IL-1 signaling in an *in vitro* panel of cellular assays as well as *in vivo* activity in murine models of PTB and OIR. Remarkably, all Agl and Hgl analogs of peptide 1 did not inhibit NF-κB signaling but blocked other pathways, such as JNK and ROCK2 phosphorylation contingent on structure and configuration. Efficacy in preventing preterm labor correlated with a capacity to block IL-1β-induced IL-1β synthesis. Furthermore, the importance of inhibition of JNK and ROCK2 phosphorylation for enhanced activity was highlighted for prevention of vaso-obliteration in the OIR model. Taken together, lactam mimic structure and stereochemistry strongly influenced conformation and biased signaling. Selective modulation of IL-1 signaling was proven to be particularly beneficial for curbing inflammation in models of preterm labor and retinopathy of prematurity (ROP). A class of biased ligands has been created with potential to serve as selective probes for studying IL-1 signaling in disease. Moreover, the small peptide mimic prototypes are promising leads for developing immunomodulatory therapies with easier administration and maintenance of beneficial effects of NF-κB signaling.

## Introduction

**Figure 1:**
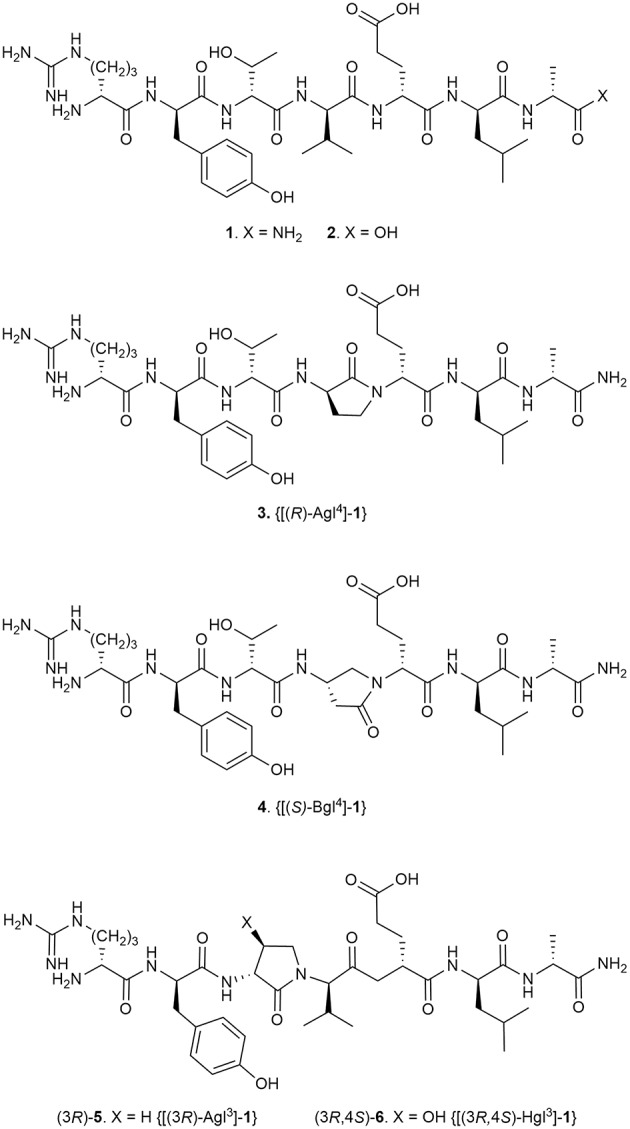
Peptide **1** and representative Agl, Bgl, and Hgl analogs.

Among examples of synthetic methods to create folded peptides, the application of α-amino-γ-lactam (Agl) residues to control folding is a mainstay for biomedical research to improve biological activity (Freidinger et al., 1980; St-Cyr et al., 2017). Previously (Jamieson et al., 2009), an Agl residue scan of the all D-amino acid linear peptide 101.10 (**1**, H-d-Arg-d-Tyr-d-Thr-d-Val-d-Glu-d-Leu-d-Ala-NH_2_, Figure 1) was used to study conformation-activity relationships of this allosteric modulator of the interleukin (IL)-1 receptor (IL-1R), which is of great interest in the treatment of many inflammatory diseases given the shortcomings of currently-approved anti-IL-1 therapeutics. The resulting lactam analogs exhibited properties that were either similar or improved relative to the parent peptide **1** (Jamieson et al., 2009; Boutard et al., 2011). Inspired by the activity of the Agl analogs but wary that the absence of side chain function may diminish potency despite a favorable geometry, the corresponding β-hydroxy-α-amino-γ-lactam (Hgl) residue was conceived to examine constraint of both backbone and side chain conformation of the d-Thr residue (St-Cyr et al., 2010). A detailed study of **1** is now reported in which all configurations of Agl, Hgl, and Val residues (e.g., **5** and **6**, Figure 1) are used to study the central d-Thr-d-Val dipeptide moiety.

IL-1 is a pro-inflammatory cytokine extensively involved in many diseases (Dinarello et al., 2012) and has been described to play a major role in inflammatory episodes leading to preterm birth (PTB) (Romero et al., 1989; Yoshimura and Hirsch, 2005; Puchner et al., 2011; Nadeau-Vallée et al., 2015) and its associated sequela of retinopathy of prematurity (ROP) (Rivera et al., 2013). IL-1 exerts its biological effects through complexation to the ubiquitously expressed IL-1R (Dinarello, 2002) and dimerization with the IL-1R accessory protein (IL-1RacP). IL-1 activates various downstream signaling mediators, notably PGE_2_ and NF-κB, which trigger hyperthermic and pro-inflammatory effects (Dinarello et al., 1999). Agents that block the effects of IL-1 offer the potential to treat diseases included in chronic inflammatory disorders such as rheumatoid arthritis and Crohn's disease (Hallegua and Weisman, 2002). Currently-approved IL-1R antagonists, such as the endogenous IL-1R antagonist (IL-1Ra), compete with the agonist (i.e., IL-1β) by binding at the orthosteric, native-ligand binding site (Braddock and Quinn, 2004). Moreover, all are large proteins (17.5–251 kDa): Kineret® (recombinant IL-1ra; Biovitrium), Canakinumab (Novartis) Gevokizumab (IL-1 monoclonal antibodies; XOMA corporation), and Rilonacept [Regeneron; soluble IL-1R (Fc-bound)]. The use of relatively large immunomodulators in clinical settings presents secondary effects such as immunosuppression, which increases the risk for opportunistic infections and pain at the site of injection. Such drawbacks may account for failures in clinical trials (Opal et al., 1997; Roerink et al., 2017; Mantero et al., 2018) and may be related to non-selective interference of all signals triggered by IL-1β because of the orthosteric competitive nature of these biologics. Preservation of the nuclear factor κ-light-chain-enhancer of activated B cells (NF-κB) has been suggested to be beneficial, due to retained anti-apoptotic activity (Castro-Alcaraz et al., 2002) and promotion of the differentiation and proliferation of B and T cells, which are critical for the immune response against pathogenic insults (Gerondakis and Siebenlist, 2010).

Heptapeptide **1** (850 Da) binds at spatially distinct sites from endogenous IL-1β, and selectively and allosterically regulates downstream signaling of inflammatory pathways (Quiniou et al., 2008; Nadeau-Vallée et al., 2015). Derived from a loop region of the IL-1RacP using a strategy for conceiving peptide modulators based on sequences from protein receptors (Hebert et al., 1996; Tan et al., 2001; Rihakova et al., 2009; Bourguet et al., 2014), peptide **1** significantly attenuated hyperoxia (oxidant stress)-induced increases in IL-1β, tumor necrosis factor-α (TNF-α), intercellular adhesion molecule (ICAM)-1, and semaphorin (Sema)-3A (Rivera et al., 2013). These molecular changes caused by peptide **1** were associated with prevention of retinal vaso-obliteration, acceleration of normal revascularization, and normalization of vascular density. In addition, fluorescein-conjugated **1** was shown to localize principally in microglial and endothelial cells in the inflamed retina subjected to oxygen-induced retinopathy (OIR) conditions. In contrast to currently approved IL-1 inhibitors, peptide **1** exhibited pharmacological selectivity by inhibiting some IL-1-triggered pathways such as p38 mitogen-activated protein kinases (p38 MAPK) and Rho-associated coiled-coil-containing protein kinase 2 (ROCK2), while preserving NF-κB (Nadeau-Vallée et al., 2015). In models of hyperthermia, inflammatory bowel disease and contact dermatitis, peptide **1** also proved superior to corticosteroids and FDA-approved IL-1Ra (Quiniou et al., 2008). In models of PTB, peptide **1** has been shown to be an effective early intervention for suppressing the downstream inflammatory cascade induced by bacterial lipopolysaccharide (LPS, to mimic chorioamnionitis), and for preventing the premature initiation of uterine activation proteins and subsequent onset of labor in mice (Nadeau-Vallée et al., 2015).

Conformational restriction of natural peptides has been used to identify active geometry and to enhance therapeutic potential by mitigating issues such as low oral availability and poor metabolic stability (Carney et al., 2014; Rubin and Qvit, 2016; Lubell, 2017). Since pioneering studies by Freidinger and Veber, the application of Agl residues has proven effective for constraining the backbone sequence to favor β-turn geometry in various peptides (Freidinger et al., 1980, 1982; Yu et al., 1988; Zhang et al., 1996; Aube, 1999; Perdih and Kikelj, 2006; Valle et al., 2009). In the interest of understanding the biologically active conformation responsible for the mechanism of action, Agl residues as well as their β-amino-γ-lactam (Bgl) counterparts have been used in structure-activity relationship studies of peptide **1** (Jamieson et al., 2009; Ronga et al., 2010; Boutard et al., 2011). Although systematic replacement of each residue of peptide **1** with D-Ala indicated that removal of certain side chains may lead to activity loss, analogs possessing one (*R*)-Agl residue, respectively at either the D-Tyr, D-Val, D-Glu, or D-Leu positions demonstrated similar efficacy, and [(*S*)-Bgl^4^]-**1** increased activity compared to peptide **1** in inhibiting IL-1β-induced DNA primase-1-dependent thymocyte TF-1 proliferation (Jamieson et al., 2009; Boutard et al., 2011). An active turn conformation situated about the D-Tyr-D-Thr-D-Val triad appeared key for potent inhibition of IL-1β-induced thymocyte TF-1 proliferation; however, [(*R*)-Agl^3^]-**1** exhibited significantly diminished activity, likely due to loss of side chain (Jamieson et al., 2009).

Employing all configurations of Agl, Hgl, and Val residues to study the d-Thr-d-Val dipeptide of **1**, the influences of backbone and side chain conformation on modulation of IL-1R signaling has now been examined *in vitro* by assessing phosphorylation of downstream IL-1 modulators and transcription of inflammatory genes. Moreover, the effects of their lactam structures and configurations have been assessed *in vivo* on murine models of preterm birth (PTB) and oxygen-induced retinopathy (OIR). These investigations have illustrated the influences of the orientation of the hydroxyl group and backbone for activity and biased signaling, particularly with respect to inhibition of NF-κB.

## Materials and Methods

### General Chemistry Methods

Unless otherwise specified, all non-aqueous reactions were performed under an inert argon atmosphere. All glassware was dried with a flame under flushing argon gas or stored in the oven, and let cool under an inert atmosphere prior to use. Anhydrous solvents (THF, DCM, MeCN, MeOH, toluene, and DMF) were obtained by passage through solvent filtration systems (Glass Contour, Irvine, CA) and solvents were transferred by syringe. Reaction mixture solutions (after aqueous workup) were dried over anhydrous MgSO_4_ or Na_2_SO_4_, filtered, and rotary-evaporated under reduced pressure. The syntheses under microwave conditions were performed on a 0–400 W Biotage® Robot Eight and Robot Sixty microwave synthesizer. Column chromatography was performed on 230–400 mesh silica gel, and thin-layer chromatography was performed on alumina plates coated with silica gel (Merck 60 F_254_ plates). Visualization of the developed chromatogram was performed by UV absorbance or staining with iodine or potassium permanganate solutions. Melting points were obtained on a Buchi melting point B-540 apparatus and are uncorrected. Specific rotations, [α]^D^ values, were calculated from optical rotations measured at 20 and 25°C in CHCl_3_ or MeOH at the specified concentrations (*c* in g/100 mL) using a 0.5 dm cell length (l) on an Anton Paar Polarimeter, MCP 200 at 589 nm, using the following general formula: [α]${}_{25}^{\text{D}}$ = (100 × α)/(l × *c*). Accurate mass measurements were performed on an LC-MSD instrument in electrospray ionization (ESI-TOF) mode at the Université de Montréal Mass Spectrometry facility. Sodium adducts [M + Na]^+^ were used for empirical formula confirmation. Nuclear magnetic resonance spectra (^1^H NMR, ^13^C NMR) were recorded on Bruker 300, 400, 500, and 700 MHz spectrometers. ^1^H NMR spectra were referenced to CDCl_3_ (7.26 ppm), CD_3_OD (3.31 ppm), C_6_D_6_ (7.16 ppm) or DMSO-d_6_ (2.50 ppm), and ^13^C NMR spectra were measured in CDCl_3_ (77.16 ppm), CD_3_OD (49.0 ppm), C_6_D_6_ (128.39 ppm) or DMSO-d_6_ (39.52 ppm) as specified below. Coupling constant *J* values were measured in Hertz (Hz) and chemical shift values in parts per million (ppm). Infrared spectra were recorded in the neat on a Perkin Elmer Spectrometer FT-IR instrument, and are reported in reciprocal centimeters (cm^−1^). Analytical LCMS and HPLC analyses were performed on a 5 μM, 50 mm × 4.6 mm C18 Phenomenex Gemini column™ with a flow rate of 0.5 mL/min using appropriate gradients from pure water containing 0.1% formic acid (FA), to mixtures with either CH_3_CN containing 0.1% FA, or MeOH containing 0.1% FA. Peptides were purified on a preparative column (C18 Gemini column™) using appropriate gradients from pure water containing 0.1% FA to mixtures with MeOH containing 0.1% FA at a flow rate of 10 mL/min.

### Chemical Reagents

Unless specified otherwise, commercially available reagents were purchased from Aldrich, A & C American Chemicals Ltd., Fluka and Advanced Chemtech™ and used without further purification, including PPh_3_, DIAD, *p*-nitrobenzoic acid, NaN_3_, piperidine, DIEA, TFA, TES, TEA, CH_3_I, NaH, Fmoc-OSu, Boc_2_O, NaIO_4_, *m*-CPBA, TFE, and HFIP. Polystyrene Rink amide resin (0.5 mmol/g) was purchased from Advanced Chemtech™, and the manufacturer's reported resin loading was used in the calculation of yields of final product. All commercially available amino acids [e.g., Fmoc-D-Ala-OH, Fmoc-D-Leu-OH, Fmoc-D-Glu(*t*-Bu)-OH, Fmoc-D-Tyr(*t*-Bu)-OH, Fmoc-D-Arg(Pmc)-OH, Boc-D-Arg(Pmc)-OH, H-L-Met-OH, H-D-Met-OH, L-Val-O-*t*-Bu∙HCl, D-Val-O-*t*-Bu∙HCl, Boc-L-Met-OH and Boc-D-Met-OH] and coupling reagents (e.g., HOBt, HBTU and DCC) were purchased from GL Biochem™ and used as received. Solvents were obtained from VWR international.

**H-****d****-Arg-****d****-Tyr-(*R*)-Agl-****d****-Val-****d****-Glu-****d****-Leu-****d****-Ala-NH**_**2**_ **[(3*R*)-Agl**^**3**^**]-1, [(3*R*)-5]**

A 10 mL plastic filtration tube equipped with a polyethylene filter was charged with polystyrene Rink amide resin, 75–100 mesh, 1%, DVB with a 0.5 mmol/g loading, (200 mg) and DCM (about 7 mL). The tube was sealed, shaken for 30 min to induce swelling and the liquid phase was removed. The Fmoc group was cleaved from the resin-bound peptide by treatment with a freshly-prepared 20% piperidine in DMF solution (about 7 mL), shaking for 30 min, and removal of the liquid phase. The resin was repeatedly (3x per solvent) washed (10 mL per wash) over a total of 6 min with DMF and DCM, and the liquid phase was removed. Generation of the free amine resin was confirmed by a positive Kaiser test. Peptide elongation was conducted by treating the DMF-swollen free amine resin with a freshly prepared acylation solution containing Fmoc-D-Ala-OH (3 eq), HBTU (3 eq), and DIEA (6 eq) in DMF (4–7 mL). After agitating for 3–5 h at room temperature, Fmoc cleavage and peptide elongation were reiterated using Fmoc-D-Leu-OH, Fmoc-D-Glu(*t*-Bu)-OH, *N*-Fmoc-(3*R*)-Agl^3^-R-Val-OH dipeptide, Fmoc-D-Tyr(*t*-Bu)-OH, and Fmoc-D-Arg(Pmc)-OH. For the coupling of *N*-Fmoc-(3*R*)*-*Agl^3^-*R*-Val-OH, only a stoichiometric quantity of dipeptide was used; for Fmoc-D-Tyr(*t*-Bu)-OH, coupling was repeated twice using a higher reaction concentration. Synthetic progress was monitored using a combination of the Kaiser test and LC-MS analyses on TFA cleaved resin aliquots, which were concentrated and dissolved in mixtures of water and MeCN. The completed peptide sequence was cleaved from the resin by treatment with TFA/H_2_O/TES (3 mL, 95/2.5/2.5, v/v/v), with shaking for 3 h, and the liquid phase was collected. The resin was repeatedly (2x) washed with TFA and the combined liquid phases were concentrated *in vacuo*. The residue was dissolved in a minimal volume of acetonitrile, precipitated with ice-cold diethyl ether, and centrifuged. The supernatant was removed by decantation and the precipitate was collected. The precipitation and collection process was repeated on the supernatant. The combined white solid precipitate was dissolved in water (5 mL), freeze-dried to give a white powder (44% crude purity), and purified using preparative HPLC on a Waters™ Prep LC instrument equipped with a reverse-phase Gemini™ C18 column (250 × 21.2 mm, 5 μm), using a gradient of MeOH (0.1% FA) in H_2_O (0.1% FA) at a flow rate of 10.0 mL/min and UV detection at 280 nm. Fractions containing pure peptide were combined and lyophilized to afford peptide (3*R*)-**5** (10 mg, 12% yield of >95% purity): LCMS [10–90% MeOH (0.1% FA) in water (0.1% FA) over 14 min; RT 8 min] and [10–90% MeCN (0.1% FA) water (0.1% FA) over 14 min; RT 5.6 min]; HRMS (ESI^+^) calcd m/z for C_38_H_62_N_11_O_10_ [M+H]^+^, 832.4676 found 832.4682.

**(3*R*, 2′*R*)-2-[3-*N*-(Fmoc)amino-2-oxopyrrolidin-1-yl]-3-methylbutanoic acid [(3*R*, 2′*R*)-7]**


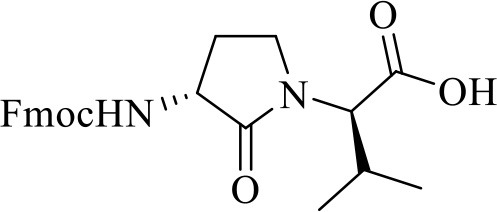


(3*R*, 2′*R*)-Acid (3*R*, 2′*R*)-**14** (1 eq., 67 mg, 0.27 mmol) was dissolved in 1:1 acetone (1 mL) and H_2_O (1 mL), treated with Na_2_CO_3_ (2 eq., 58 mg, 0.55 mmol) followed by Fmoc-OSu (1.1 eq., 100 mg, 0.3 mmol), stirred at rt for 18 h, and concentrated to a residue that was dissolved in H_2_O and acidified to pH 3–4 with citric acid (0.5 N solution). The aqueous solution was extracted multiple times with EtOAc until the aqueous layer no longer exhibited the carbamate by TLC. The organic layer was extracted with saturated NaHCO_3_ (2 × 3 mL). The aqueous extractions were combined, acidified to pH 3–4 with 0.5 N citric acid solution, and extracted with EtOAc. The organic layer was washed with brine, dried over MgSO_4_, filtered, and concentrated to afford (3*R*, 2′*R*)-carbamate (3*R*, 2′*R*)-**7** (87 mg, 0.21 mmol, 75% yield): R_*f*_ = 0.26 (10% MeOH:DCM); [α]${}_{\text{D}}^{25}$ 13.2° (*c* 0.41, MeOH); ^1^H NMR (300 MHz, MeOD) δ 7.80 (d, *J* = 7.6, 2H), 7.68 (d, *J* = 7.4, 2H), 7.40 (t, *J* = 7.1, 2H), 7.31 (t, *J* = 6.9, 2H), 5.60 (d, *J* = 5, 1H), 4.53–4.18 (m, 5H), 3.74 (t, *J* = 9.2, 1H), 3.49–3.35 (m, 1H), 2.56–2.37 (m, 1H), 2.32–2.16 (m, 1H), 2.10–1.85 (m, 1H), 1.44–1.21 (m, 1H), 1.03 (d, *J* = 6.7, 3H), 0.93 (d, *J* = 6.7, 3H); ^13^C NMR (75 MHz, MeOD) δ 173.9, 171.6, 157.2, 143.9, 141.2, 127.4, 126.8, 124.8, 119.5, 66.7, 60.8, 52.2, 41.2, 28.1, 26.2, 18.4, 18.1; HRMS (ESI^+^) calcd *m/z* for C_24_H_27_N_2_O_5_ [M+H]^+^, 423.1914 found 423.1917.

**(*R*)-*N*-(Boc)-Methioninyl-(*R*)-valine *tert*-butyl ester [(*R***, ***R*)-10]**


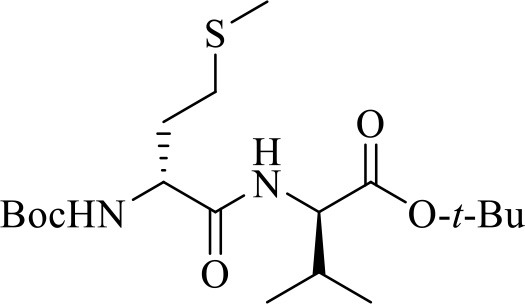


*N*-Boc-(*R*)-Methionine [(*R*)-**8**, 1 eq., 1 g, 4 mmol] and (*R*)-valine *tert*-butyl ester hydrochloride [(*R*)-**9** HCl, 1 eq., 840 mg, 4 mmol] were dissolved in dry DMF (6 mL). On treatment of the mixture with triethyl amine (1 eq., 400 mg, 0.56 mL, 4 mmol) a precipitate was formed, and HOBt (1 eq., 540 mg, 4 mmol) and DCC (1 eq., 830 mg, 4 mmol) were added to the mixture. The mixture was stirred at rt for 24 h, filtered and the filter cake was washed with DCM. The filtrate and washings were combined concentrated under vacuum. The residue was dissolved in EtOAc (6 mL), washed with 0.5 M citric acid (3 × 4 mL), 2 N aqueous NaHCO_3_ (3 × 4 mL) and brine (4 mL), dried over Na_2_SO_4_, filtered and concentrated under vacuum to give (*R*,*R*)-dipeptide (*R*,*R*)-**10** as a white foam (1.27 g, 3.13 mmol, 78% yield): R_*f*_ = 0.28 (20% EtOAc in hexane); [α] ${}_{\text{D}}^{25}$ −4.1° (*c* 1.95, CHCl_3_); ^1^H NMR (300 MHz, CDCl_3_) δ 6.61 (d, *J* = 8.4, 1H), 5.20 (d, *J* = 8.0, 1H), 4.40 (dd, *J* = 8.8, 4.6, 1H), 4.35-4.22 (br, 1H), 2.59 (t, *J* = 7.2, 2H), 2.23-1.86 (m, 5H), 1.46 (s, 9H), 1.44 (s, 9H), 0.91 (dd, *J* = 7.6, 7.1, 6H); ^13^C NMR (75 MHz, CDCl_3_) δ 171.4, 170.7, 155.6, 82.1, 80.2, 57.7, 53.4, 31.6, 31.4, 30.3, 28.4, 28.2, 19.1, 17.6, 15.3; HRMS (ESI^+^) calcd *m/z* for C_19_H_36_N_2_O_5_S [M+H]^+^, 405.2418 found 405.2433.

***N*-Boc-(*R*)-Methioninyl-(*R*)-valine *tert*-butyl ester methylsulfonium iodide [(*R***, ***R*)-11]**


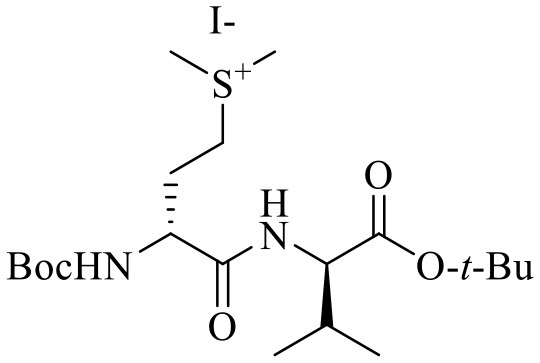


(*R*,*R*)-Dipeptide (*R*,*R*)-**10** (1 eq., 500 mg, 1.24 mmol) was dissolved in CH_3_I (39 eq., 3 mL, 48.2 mmol) and stirred at rt for 24 h, and concentrated under vacuum. The volatile contaminants were removed by repeated (3x) co-evaporation with DCM to give (*R*,*R*)-methylsulfonium iodide (*R*,*R*)-**11** as yellow gummy foam (655 mg, 1.2 mmol, 97% yield): R_*f*_ = 0.2 (5% MeOH in DCM); [α] ${}_{\text{D}}^{25}$ 22° (*c* 1, MeOH); ^1^H NMR (300 MHz, CDCl_3_) δ 7.53 (d, *J* = 7.1, 1H), 5.98 (d, *J* = 7.2, 1H), 4.60 (dd, *J* = 12.5, 7.3, 1H), 4.24 (dd, *J* = 7.8, 5.2, 1H), 3.96-3.81 (m, 1H), 3.73–3.58 (m, 1H), 3.32 (s, 3H), 3.21 (s, 3H), 2.64–2.47 (m, 1H), 2.42–2.14 (m, 2H), 1.45 (s, 9H), 1.42 (s, 9H), 0.99 (t, *J* = 7.3, 6H); HRMS (ESI^+^) calcd *m/z* for C_20_H_39_N_2_O_5_S [M]^+^, 419.2574 found 419.2567.

***tert*-Butyl (3*R*, 4*S*, 2′*R*)-2-[3-(Fmoc)amino-4-hydroxy-2-oxopyrrolidin-1-yl]-3-methylbutanoate [(3*R*, 4*S*, 2′*R*)-12]** was prepared as previously described (Geranurimi and Lubell, 2018).

**(3*R*, 2′*R*)-*tert*-Butyl 2-[3-(Boc)amino-2-oxopyrrolidin-1-yl]-3-methylbutanoate [(3*R*, 2′*R*)-13]**


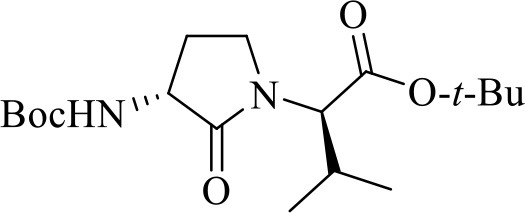


(*R*,*R*)-Methylsulfonium iodide (*R*,*R*)-**11** (1 eq., 158 mg, 0.289 mmol) was dissolved in a 1:1 mixture of DMF (3.5 mL) and DCM (3.5 mL) under argon, cooled to 0°C, treated with NaH (60% dispersion in mineral oil, 1.2 eq., 14 mg, 0.347 mmol), and stirred at 0°C for 2.5 h. The reaction was diluted with methyl acetate (2.5 mL) and H_2_O (0.5 mL) and allowed to warm to rt with stirring overnight. The reaction mixture was concentrated under vacuum, quenched with 1 M NaH_2_PO_4_, and extracted three times with EtOAc. The combined EtOAc layers were dried over MgSO_4_, filtered and concentrated to a residue that was purified by column chromatography on silica gel using 20% of EtOAc in hexane as eluent. Evaporation of the collected fractions provided (3*R*,2′*R*)-lactam (3*R*,2′*R*)-**13** (60 mg, 0.17 mmol, 58% yield): R_*f*_ = 0.31 (30% EtOAc in hexane); [α] ${}_{\text{D}}^{25}$ 35.8° (*c* 1.6, CHCl_3_); ^1^H NMR (300 MHz, CDCl_3_) δ 5.09 (s, 1H), 4.39 (d, *J* = 9.3, 1H), 4.31–4.16 (br s, 1H), 3.72 (t, *J* = 9.1, 1H), 3.27 (m, 1H), 2.72–2.58 (m, 1H), 2.26–2.11 (m, 1H), 1.90–1.71 (m, 1H), 1.45 (s, 9H), 1.44 (s, 9H), 0.98 (d, *J* = 6.7, 3H), 0.89 (d, *J* = 6.8, 3H); ^13^C NMR (75 MHz, CDCl_3_) δ 173.0, 169.7, 156.1, 82.1, 80.0, 61.0, 52.4, 41.7, 29.4, 28.8, 28.5, 28.2, 19.4, 19.3; HRMS (ESI^+^) calcd *m/z* for C_18_H_33_N_2_O_5_ [M+H]^+^, 357.2384 found 357.2399.

**(3*R*, 2′*R*)-2-[3-Amino-2-oxopyrrolidin-1-yl]-3-methylbutanoate trifluoroacetate [(*3R, 2*′*R*)-14]**


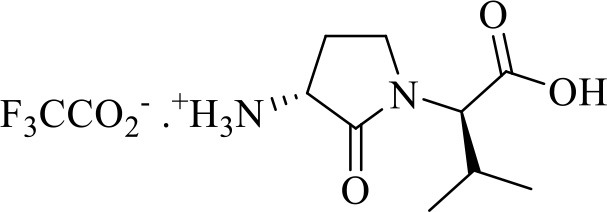


A solution of (3*R*,2′*R*)-lactam (3*S*,2′*S*)-**13** (1 eq., 100 mg, 0.281 mmol) in TFA (1 mL) and DCM (1 mL) was stirred at rt. After TLC analysis revealed complete consumption of the carbamate, the volatiles were evaporated on a rotary evaporator, and the residue was precipitated from ice-cooled diethyl ether and collected using a centrifuge to yield (3*R*,2′*R*)-trifluoroacetate (3*R*,2′*R*)-**14** (77.6 mg, 0.247 mmol, 88% yield) as white precipitate: R_*f*_ = 0.1 (1:9:90 Et_3_N:MeOH:DCM); [α]${}_{\text{D}}^{25}$ 45.6° (*c* 0.5, MeOH); ^1^H NMR (400 MHz, MeOD) δ 4.40 (d, *J* = 9.3, 1H), 4.17 (dd, *J* = 11.0, 8.5, 1H), 3.92–3.81 (m, 1H), 3.60–3.45 (m, 1H), 2.66–2.54 (m, 1H), 2.35–2.19 (m, 1H), 2.14–1.97 (m, 1H), 1.05 (d, *J* = 6.7, 3H), 0.98 (d, *J* = 6.8, 3H); ^13^C NMR (75 MHz, MeOD) δ 171.3, 170.0, 60.8, 50.3, 41.6, 28.2, 24.5, 18.3, 18.2; HRMS (ESI^+^) calcd *m/z* for C_9_H_17_N_2_O_3_ [M+H]^+^, 201.1234 found 201.1226.

***N*-Fmoc-(*R*)-Methionine methyl ester [(*R*)-15] and *N*-Fmoc-(*R*)-Methionine sulfoxide methyl ester [(*R*)-16]** were prepared by a modified version (vide infra) of the previously described method (Sicherl et al., 2010).

***N*-Fmoc-(2*R*)-Vinylglycine methyl ester [(*R*)-17] and (2*R*, 2′*R*)- and (2*R*, 2′*S*)-Methyl 2-(oxiranyl)-*N*-(Fmoc)glycinate [(2*R*, 2′*R*)-18 and (2*R*, 2′*S*)-18]** were prepared as previously described (St-Cyr et al., 2010).

***N*-Fmoc-(3*R*, 4*S*, 2′*R*)-Hgl-Val-*t*Bu [(3*R*, 4*S*, 2′*R*)-19] and (3*R*, 4*R*, 2′*R*)-*tert*-Butyl 2-[3-(Fmoc)Amino-4-*p*-nitrobenzoyloxy-2-oxopyrrolidin-1-yl]-3-methylbutanoate [(3*R*, 4*R*, 2′*R*)-20]** were prepared as previously described (Geranurimi and Lubell, 2018).

***N*-Fmoc-[(3*R*)-Agl**^**3**^**]-D-Val-D-Glu(*t*-Bu)-D-Leu-D-Ala-Rink amide resin (22)**


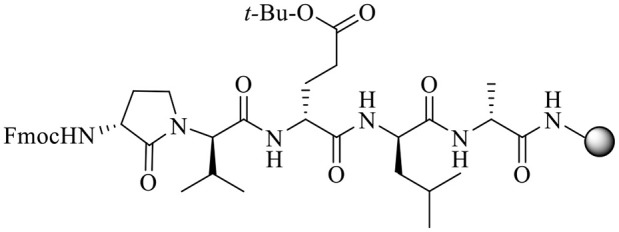


A solution of acid (3*R*,2′*R*)-**7** (1 eq., 87 mg, 0.2 mmol) and 2-(1H-benzotriazol-1-yl)-1,1,3,3- tetramethyluronium hexafluorophosphate (HBTU, 1 eq., 78 mg, 0.2 mmol) in dimethylformamide (DMF, 2 mL) was stirred for 1 min, treated with *N*,*N*-diisopropylethylamine (DIEA, 2 eq., 53.2 mg, 68 μL, 0.4 mmol), stirred for 5 min and added to H-D-Glu(*t*-Bu)-D-Leu-D-Ala-Rink amide resin **21** (400 mg, 0.5 mmol/g, 0.2 mmol), which was placed earlier into a 3-mL plastic filtration tube equipped with a polyethylene filter and swollen in DMF (2 mL). The resin mixture was shaken at rt for 5 h, and the liquid phase was removed by filtration. The resin was repeatedly (3x per solvent) washed (15 s per wash) with DMF and DCM, and dried under vacuum to afford resin **22**. To assess resin-bound peptide purity, a resin aliquot (5 mg) was placed into a 1-mL plastic filtration tube equipped with a polyethylene filter and treated with 20% piperidine in DMF. After 0.5 h, the liquid phase was removed by filtration and the resin was treated with 0.5 mL of a 95:2.5:2.5 cocktail of TFA:H_2_O:triethylsilane (TES) at rt for 1 h, and filtered. The filtrate was collected in a 1.5 mL tube and concentrated by purging with an air stream. The residue was treated with Et_2_O (1 mL), and centrifuged for 2 min. After decantation, the precipitate was dissolved in H_2_O (1 mL) and analyzed by LCMS [10–90% MeOH (0.1% FA)/ water (0.1% FA), 14 min, RT 9.2 min] and [10–90% MeCN (0.1% FA)/ water (0.1% FA), 14 min, RT 7.6 min].

### Circular Dichroism Spectroscopy

CD spectra were recorded on a Chirascan CD Spectrometer (Applied Photophysics, Leatherhead, United Kingdom) using a 1.0 cm path-length quartz cell containing 20 μM of peptide dissolved in Milli-Q water. The experimental settings were as follows: 1 nm, bandwidth; 0.5 nm, step size; 3 s, sampling time.

### Animals

Timed-pregnant CD-1 mice were obtained from Charles River (Saint-Constant, QC, Canada) at gestational day 12 and were acclimatized for 4 days prior to experiments. Animals were used according to a protocol approved by the Animal Care Committee of Hôpital Sainte-Justine in accordance with the principles of the Guide for the Care and Use of Experimental Animals of the Canadian Council on Animal Care. The animals were maintained on standard laboratory chow under a 12 h:12 h light/dark cycle and allowed free access to chow and water.

Two-day-old (P2) Sprague Dawley rat pups and their mothers were ordered from Charles River (Saint-Constant, QC, Canada) and acclimatized for 3 days. The rats were housed in standard cages with *ad libitum* access to food and water and kept in a 12 h:12 h light/dark cycle. To control for the effects of litter size on retinal development, the sizes of litters were reduced to 12 by sacrificing excess pups by decapitation while under 2% isoflurane anesthesia. All procedures and protocols involving the use of these rats were approved by the Animal Care Committee of the research center of Hôpital Maisonneuve-Rosemont and are in accordance with the Statement for the Use of Animals in Ophthalmic and Vision Research approved by the Association for Research in Vision and Ophthalmology (ARVO), and guidelines established by the Canadian Council on Animal Care.

### Reagents

Chemicals were purchased from the following manufacturers: human rIL-1β (200-01B; PeproTech), LPS (L2630; Sigma-Aldrich), rytvela (peptide **1**) (Elim Biopharmaceuticals, Hayward, CA) and Kineret (Anakinra, Sobi, Biovitrum Stockholm, Sweden).

### qPCR Experiments

HEK-Blue IL-33/IL-1β cells were purchased from InvivoGen (San Diego, CA) and used at passages under 15. HEK-Blue cells were cultured in DMEM growth medium supplemented with 10% serum, 50 U/mL penicillin, 50 mg/mL streptomycin, 200 mg/mL zeocin, and 100 mg/mL hygromycin. Cells were grown in regular conditions (37°C, 5% CO_2_). Cells were serum starved overnight and treated with 100 ng/mL IL-1β for 4 h. Cells were pre-incubated for 30 min with peptides **1**, **5**, or **6** (10^−6^M) or Kineret (1.0 mg/mL) prior to the experiments to reach equilibrium (*n* = 4 each treatment). Cells were harvested and incubated for 5 min in RIBOzol (AMRESCO). RNA was extracted according to manufacturer's protocol and RNA concentration and integrity were measured with a NanoDrop 1,000 spectrophotometer. A total of 500 ng RNA was used to synthetize cDNA using iScript Reverse Transcription SuperMix (Bio-Rad, Hercules, CA). Primers were designed using National Center for Biotechnology Information Primer Blast and are shown in Table 1. Quantitative gene expression analysis was performed on Stratagene MXPro3000 (Stratagene) with SYBR Green Master Mix (Bio-Rad). Gene expression levels were normalized to 18S universal primer (Ambion Life Technology, Burlington ON, Canada). Genes analyzed include *IL1*β*, IL6*, and *PTGHS2* [Prostaglandin H synthetase 2 or cyclooxygenase-2 (COX-2)]. Data are representative of 3 experiments (each with *n* = 4 per treatment group).

**Table 1:** List of primers for the human genes assessed by qPCR.

**Gene**	**Forward primer (5^**′**^ → 3^**′**^)**	**Reverse primer (5^**′**^ → 3^**′**^)**
*IL1β*	AGCTGGAGAGTGTAGATCCCAA	ACGGGCATGTTTTCTGCTTG
*IL6*	TTCAATGAGGAGACTTGCCTGG	CTGGCATTTGTGGTTGGGTC
*PTGHS2*	ATATTGGTGACCCGTGGAGC	GTTCTCCGTACCTTCACCCC

### NF-kB QUANTI-Blue Assay

HEK-Blue IL-33/IL-1β cells (InvivoGen) were pretreated with peptides **1**, **5**, or **6** (10^−6^M) or Kineret (1.0 mg/mL) for 30 min, followed by treatment with a constant concentration of IL-1β (100 ng/mL), and then incubated at 37°C for 4 h. Levels of secreted alkaline phosphatase in cell culture supernatant were determined using the QUANTI-Blue assay, according to the manufacturer's instructions (InvivoGen). Alkaline phosphatase activity was assessed by measuring the optical density (OD) at 620–655 nm with a micro plate reader (EnVision Multilabel reader; PerkinElmer, Waltham, MA). Data are representative of 3 experiments (each with *n* = 4 per treatment group).

### p38 MAPK, ROCK2, and JNK Phosphorylation Assay

RAW Blue cells (Invivogen) were grown under standard conditions (37°C, 5% CO_2_) and maintained under passage number 15. Cells were equilibrated with **1**, **5**, or **6** (10^−6^M) or Kineret (1.0 mg/mL) for 30 min, after which time they were exposed to IL-1β (100 ng/mL) for 15 min. Cells were harvested and lysed on ice for 30 min using a radioimmunoprecipitation assay buffer (Cell Signaling) supplemented with 1 mM phenylmethylsulfonyl fluoride (PMSF) and cOmplete™ EDTA-free protease inhibitor cocktail (Roche, Mannheim, Germany, prepared according to the manufacturer's instructions). Protein concentrations were determined using a Bradford protein assay (Bio-Rad) on 96-well plates with a microplate reader (EnVision Multilabel reader) measuring OD at 595 mm. Bovine serum albumin serial dilutions were used to generate a standard curve. Lysates were then mixed with 4X reducing sample buffer (Bio-Rad).

Lysates were loaded (30 μg protein per well) in a 5% acrylamide stacking gel, and samples were electrophoresed in a 12% acrylamide resolving gel for 1.5 h at 120 V, followed by a 1 h transfer onto polyvinylidene difluoride (PDVF) membranes at 100 V. Membranes were blocked and incubated with 1:1,000 dilution of primary antibody and 1:20,000 dilution of secondary antibodies according to the manufacturer's instructions. Antibodies used were for phospho-p38 MAPK (Cell Signaling, #9211), p38 MAPK (Cell Signaling, #9212), SAPK/JNK (Cell Signaling, #9252), phospho-SAPK/JNK (Cell Signaling, #9251), ROCK2 (Thermo Fisher Scientific PA5-21131), phospho-ROCK2 (Thermo Fisher Scientific PA5-34895), and goat anti-rabbit conjugated to horseradish peroxidase (Abcam, ab6721). Membranes were imaged using an Amersham Imager 600 (GE Healthcare) using Clarity Western ECL Substrate (Bio-Rad). The intensity of protein bands was quantified using ImageJ and standardized using total (phosphorylated + non-phosphorylated) protein content. Data are representative of three independent experiments.

### Radioligand Displacement Assay

Displacement of radiolabeled peptide **1** was performed as described previously (Quiniou et al., 2008). Briefly, RAW-Blue macrophage cells (InVivogen; San Diego, CA) were pre-incubated for 20 min with 100 μM of non-radiolabeled (“cold”) peptide **1**, **5**, or **6** followed by incubation for 2 h at 37°C with [^125^I]-peptide **1** at 600 nM concentration to ensure maximal specific binding (Quiniou et al., 2008). Cells were washed four times with PBS buffer and lysed with 0.1 N NaOH/0.1% Triton X-100. Bound radioactivity was measured on cell lysates with a Hidex AMG gamma counter (Hidex; Turku Finland). Data are representative of three independent experiments.

### LPS-Induced Preterm Model in Mice

Timed-pregnant CD-1 mice at 16.5 days of gestation (G16.5) were anesthetized with 2% isoflurane and received an intraperitoneal injection of LPS (*n* = 4 per group, a single dose of 10 μg) (Nadeau-Vallée et al., 2015, and Kakinuma et al., 1997). A dosage of 2 mg/kg/day of peptide **1**, **5**, or **6** or vehicle was injected subcutaneously in the neck, every 12 h until delivery. On G16.5, a dose of 1 mg/kg was injected 30 min before stimulation with LPS (to allow distribution of drugs to target tissues) and 1 mg/kg was injected 12 h after stimulation (*n* = 4 each treatment). Mice delivery was assessed every hour until term (G19–G19.5). A mouse is considered as delivering prematurely if the first pup is delivered earlier than G18.5.

### Oxygen-Induced Retinopathy in Sprague Dawley Rats

On P5, litters were transferred to a controlled hyperoxic environment (Biospherix OxyCycler A84XOV) and maintained at 80 ± 1% O_2_, which is used to induce vaso-obliteration of the retinal vasculature. At this timepoint, the vasculature is immature and particularly susceptible to hypoxic insults resulting in vaso-obliteration, a defining characteristic of the early stage of retinopathies in premature infants (Garner and Ashton, 1971). Control litters were not exposed to hyperoxia and were kept in standard conditions with room air (21% O_2_). To control for the effects of hyperoxia on the lactation of the dams, dams of hyperoxic litters were switched with dams of control litters on P8.

In litters exposed to hyperoxia, pups were randomized to receive phosphate-buffered saline (PBS) vehicle, 2 mg/kg/day of peptides **1**, **5**, or **6** or 3 mg/kg/day of Kineret from P5 to P10. These doses were determined from our previous study (Rivera et al., 2013) and administered in twice-daily intra-peritoneal injections titrated to 20 μL per injection with 28-gauge insulin syringes. A total of 6–8 pups were used for each treatment group.

On P10, pups were euthanized by decapitation under 2% isoflurane anesthesia. Eyes were enucleated and fixed in 4% paraformaldehyde for 1 h at room temperature before washing twice with PBS and storage at 4°C in PBS until further processing.

### Retinal Flatmount and Immunohistochemistry

Under a dissecting microscope, the cornea and lens were gently removed from the eyes and remnants of the hyaloid vasculature were removed from the retinas using surgical scissors and tweezers. The retinas were gently removed from the underlying sclera/choroid complex.

Retinas were treated at room temperature for 1 h with a blocking solution consisting of 1% bovine serum albumin (BSA), 1% normal goat serum, 0.1% Triton X-100 and 0.05% Tween-20 in PBS. The retinas were then double-labeled overnight at 4°C with gentle shaking in an antibody solution consisting of 1 mM CaCl_2_, 1% Triton X-100, 1% TRITC-conjugated lectin cell endothelial marker from *Bandeiraea simplicifolia* (Sigma-Aldrich, St Louis, MO) and a 1:500 dilution of rabbit anti-ionized calcium-binding adapter molecule (Iba)-1 antibody (Wako Chemicals USA) in PBS. The retinas were then washed thrice with PBS and incubated with a secondary antibody solution consisting of 1% BSA, 0.1% Triton X-100, 0.05% Tween-20 and 1:500 Alexa-594-conjugated donkey anti-rabbit IgG (ThermoFisher Scientific, Rockford, IL) in PBS for 2 h at room temperature. Retinas were washed thrice with PBS and then flat-mounted onto glass slides with coverslips and Fluoro-Gel mounting medium (Electron Microscopy Sciences, Hatfield, PA).

### Microscopy

For assessment of vaso-obliteration, retinas were imaged using an epifluorescence microscope at 10X magnification (Zeiss AxioImager Z2) and version 4.8 of the AxioVision software. Images were captured and stitched together using the software's MosaiX feature. Iba-1 staining was assessed with the same microscope at 20X magnification, and a total of 4 fields per retina were imaged halfway between the optic nerve and the edge of the retina.

Representative images of Iba-1 staining were taken using a laser scanning confocal microscope (Olympus IX81 with Fluoview FV1000 Scanhead) with the Fluoview Software at 30X magnification.

### Quantification and Data Analysis

The FIJI software was used to the quantify the area of vaso-obliteration in each retina, expressed as a percentage of the area of the whole retina. The number of Iba-1-positive cells was counted using the cell counter plug-in in FIJI, and the average of cell counts in 4 fields per retina was calculated.

Data was analyzed using GraphPad Prism 7 with one-way ANOVA and Dunnett's test for multiple comparisons. Outliers were detected using Grubb's test. Results were treated as significant when *p* was < 0.05 and expressed as mean ± SEM.

## Results

### Chemical Synthesis

The application of lactam-bridged dipeptides by Freidinger et al. at Merck Sharp and Dohme Research Laboratories in the early 1980s marked an important milestone in the use of heterocycle synthesis to design constrained peptide mimics (Freidinger et al., 1980, 1982; Freidinger, 2003). Such so-called Freidinger-Veber lactams (e.g., Agl and Hgl) restrict backbone conformation by limiting rotation about the ϕ*-*, ψ*-*, and ω-dihedral angles such that Agl and Hgl residues prefer to situate at the *i*+1 residue in β-turn sequences contingent on sequence stereochemistry (Freidinger, 2003; St-Cyr et al., 2017). Moreover, in the case of Hgl, the hydroxyl group side chain orientation is locked due to restriction of the χ-dihedral angle (Sicherl et al., 2010). Although Freidinger-Veber lactams have been used to study a wide range of biologically active peptides (Freidinger, 2003), to the best of our knowledge, the application of all stereoisomers of the heterocyclic dipeptide in a sequence has yet to be explored.

**Figure 2:**
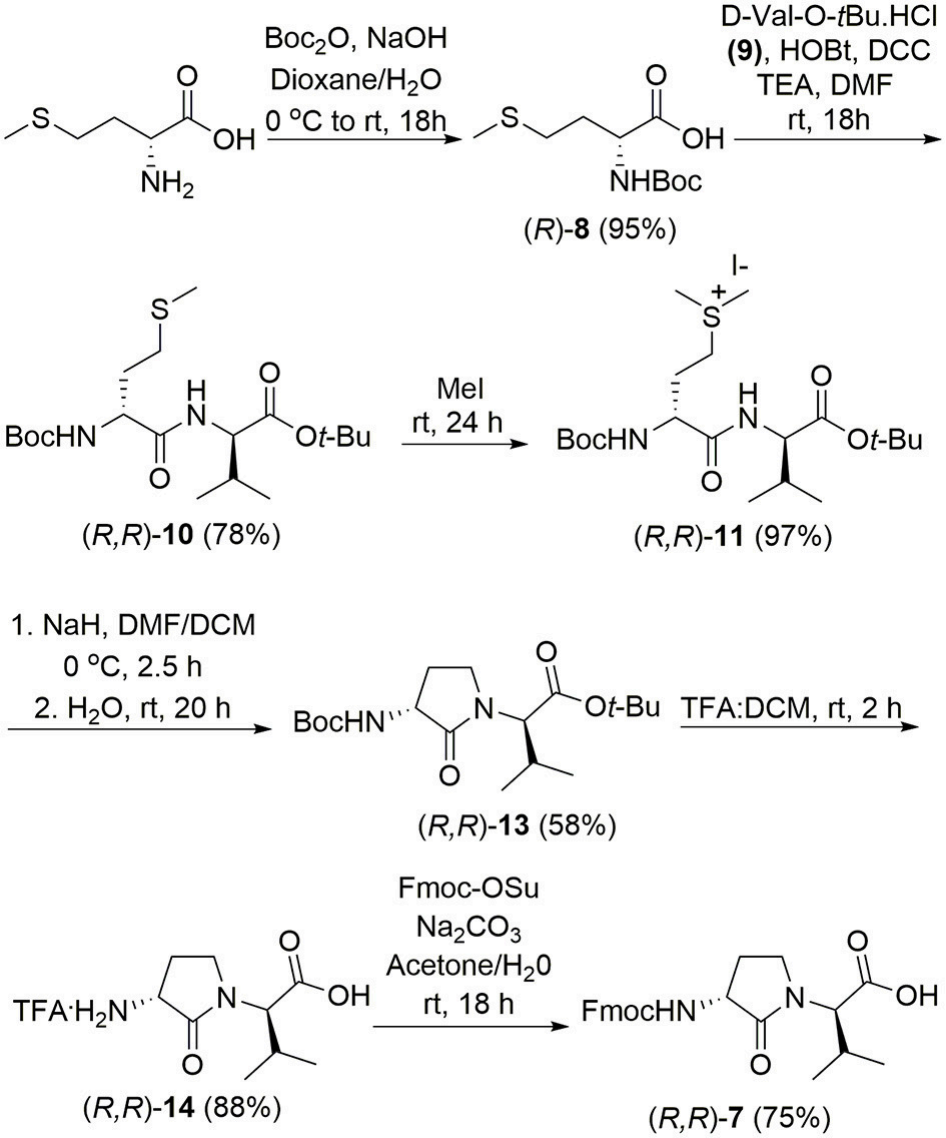
Synthesis of α-amino-γ-lactam (Agl) using protected (*R*)-methionine-(*R*)-valine dipeptide.

The syntheses of all four Agl-Val and eight Hgl-Val diastereomers of peptide **1** (e.g., **5** and **6**) were performed by approaches featuring preparation of the *N*-Fmoc-dipeptides in solution, followed by their incorporation into the final peptides using solid-phase peptide synthesis. Although effective means for introducing Agl and Hgl residues directly on solid phase have been developed (Jamieson et al., 2009, 2013; Ronga et al., 2010; Sicherl et al., 2010; St-Cyr et al., 2010; Boutard et al., 2011), the construction of dipeptide building blocks in solution prior to coupling on resin was selected to ensure significant amounts of each isomer for biological studies, as well as to minimize concerns of epimerization during peptide assembly. The *N*-Fmoc-Agl-Val-OH dipeptide building blocks **7** were synthesized by extension of the original method for Agl residue assembly featuring alkylation of the thioether of *N*-Boc-methionyl dipeptide ester, followed by intramolecular displacement of the resulting sulfonium ion under basic conditions (Freidinger et al., 1980, 1982). As illustrated for the synthesis of *N*-Fmoc-(*R*)-Agl-(*r*)-Val-OH [(*RR*)-**7**, Figure 2], *N*-Boc-d-methionine **8** was coupled to *tert*-butyl (*r*)-valinate **9** using DCC, HOBT and triethylamine to provide protected dipeptide **10** in 78% yield. The lactam was installed by *S*-alkylation using methyl iodide followed by intramolecular *N*-alkylation using sodium hydride to furnish *N*-Fmoc-(*R*)-Agl-(*R*)-Val-O*t*-Bu **11** in 58% yield (Freidinger et al., 1980, 1982). Subsequently, the acid labile carbamate and ester groups were removed using a 50% solution of TFA in DCM, and amine acylation was performed using Fmoc-OSu and sodium carbonate to provide dipeptide **7** for solid-phase synthesis. The other three diastereomers of **7** were, respectively, synthesized using different enantiomers of **8** and **9**, such that all four dipeptide building blocks were obtained in 26–32% overall yields from L- and D-methionine (Figure 2).

**Figure 3:**
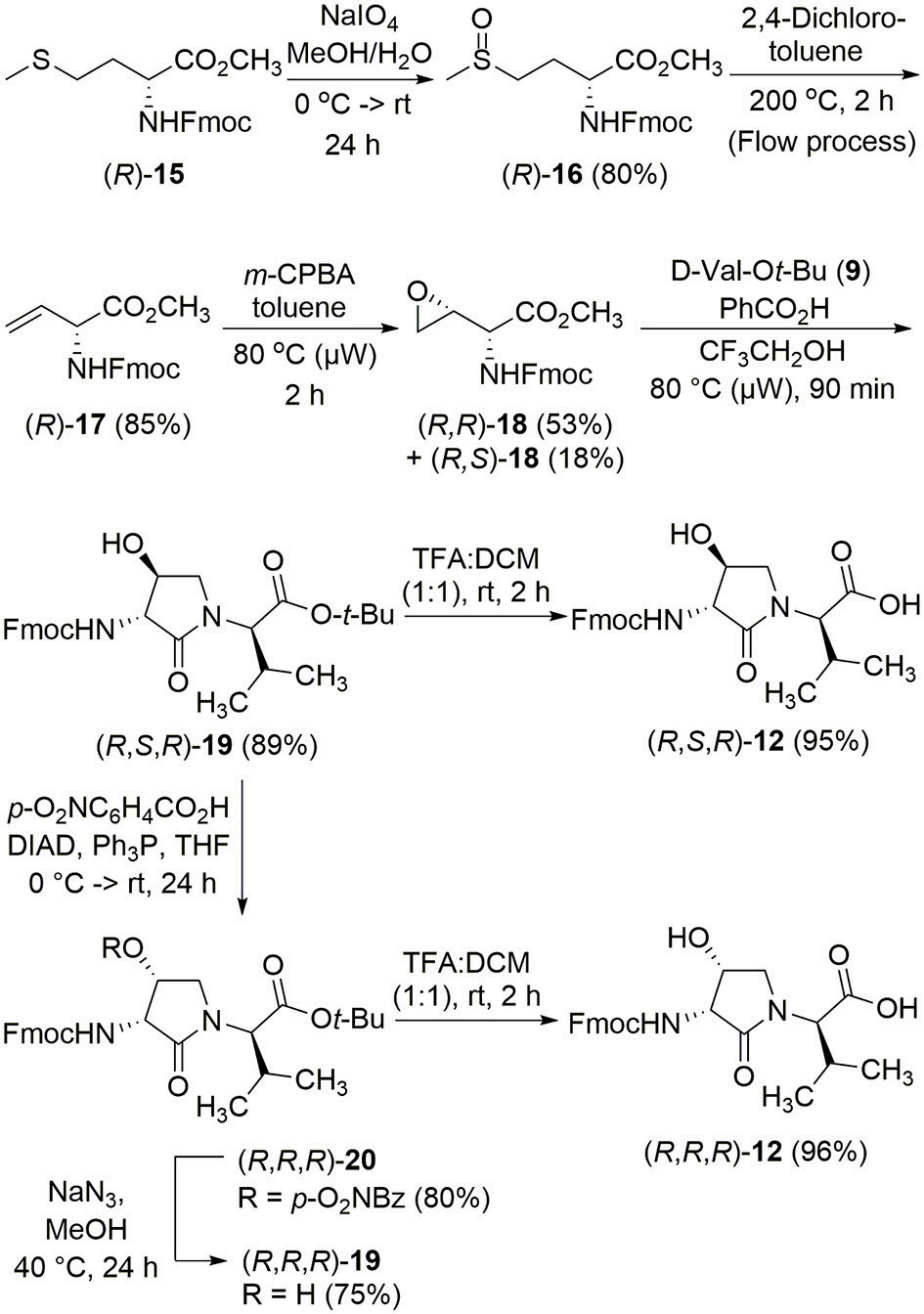
Synthesis of β-hydroxy-α-amino-γ-lactams (Hgl) dipeptides using *N*-(Fmoc)oxiranylglycine.

The synthesis of Hgl peptides in solution and on solid phase has been accomplished using oxiranyl glycine (Sicherl et al., 2010; St-Cyr et al., 2010, 2017). For preparation of *N*-Fmoc-Hgl-Val-OH diastereomers **12** possessing the *trans*-lactam residue, oxiranyl glycines (2*R*,3*R*)- and (2*S*,3*S*)-**18** were prepared from *N*-Fmoc-d- and l-methionine methyl esters (*R*- and *S*-**15**) using a modification of the reported three-step protocol in which *N*-Fmoc-vinylglycine methyl ester **17** was synthesized by a flow process featuring elimination of *N*-Fmoc-methionine sulfoxide methyl ester **16** using 2,4-dichlorotoluene at 200°C, and epoxidation of olefin **17** was performed by microwave irradiation using *m*-CPBA in toluene at 80°C (Figure 3). Epoxide (2*R*,3*R*)-**18** was reacted with *tert*-butyl (*R*)-valinate **9** using a catalytic amount of benzoic acid and trifluoroethanol under microwave irradiation at 80°C for 90 min to provide *N*-Fmoc-(2*R*,3*S*,2'*R*)-Hgl-d-Val-O*t*-Bu [(2*R*,3*S*,2'*R*)-**19**] in 89% yield. A Mitsunobu approach was employed for the synthesis of the *cis*-Hgl isomers by inversion of the alcohol stereochemistry of their *trans*-counterparts (Geranurimi and Lubell, 2018). *tert*-Butyl esters **19** were converted to the corresponding acids **12** using 1:1 TFA/DCM. Employing the respective (2*R*,3*R*)- and (2*S*,3*S*)-diasteromers of epoxide **18** and enantiomers of valine **9**, the *trans*- and *cis*-Hgl dipeptide diasteromers **12** were, respectively, synthesized in 84 and 83% yields.

**Figure 4:**
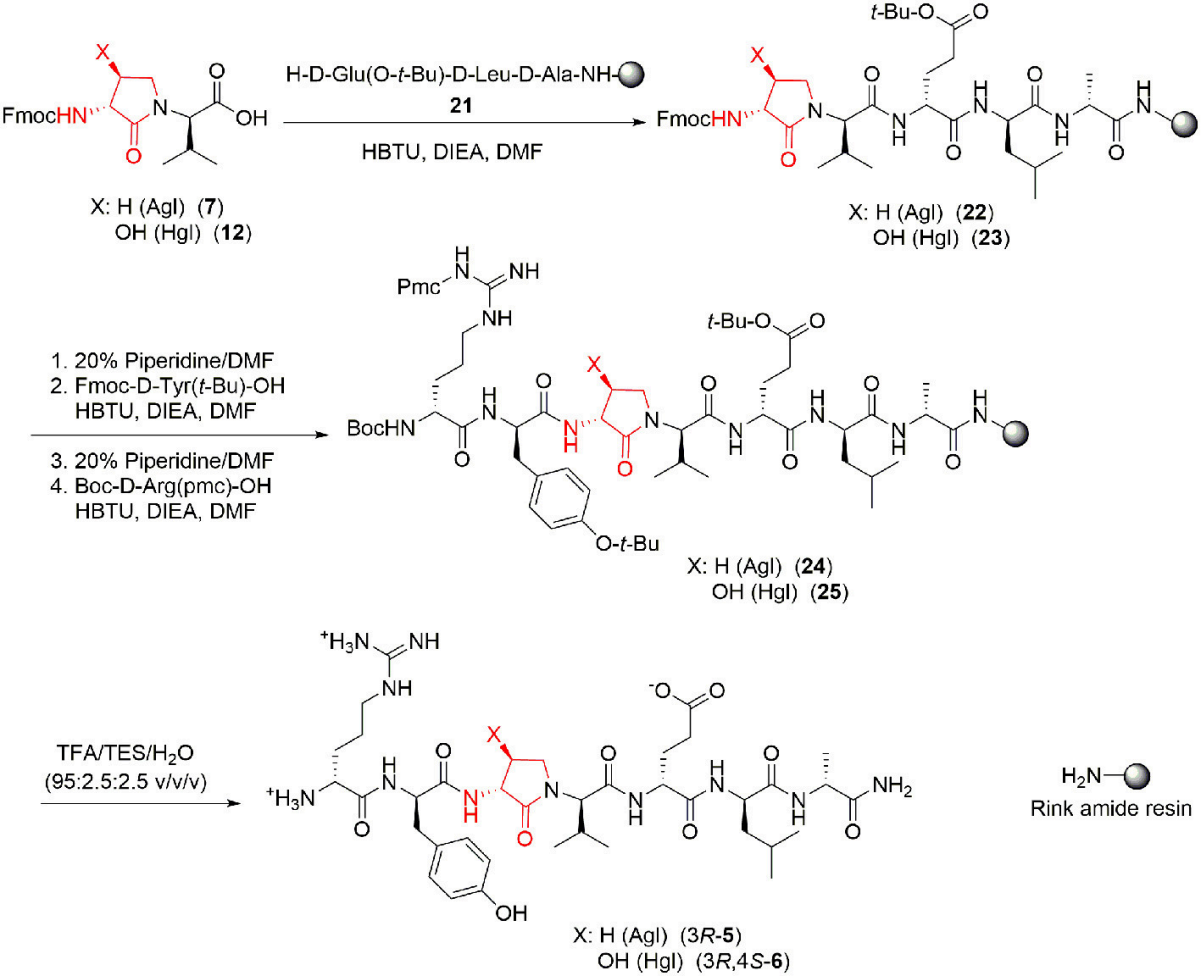
Representative protocols for solid-phase synthesis of Agl and Hgl peptides **5** and **6**.

With Agl and Hgl dipeptide diastereomers **7** and **12** in hand, the syntheses of the respective [Agl^3^]- and [Hgl^3^]-**1** peptides (**5** and **6**) were performed using standard Fmoc-based solid-phase synthesis on Rink amide resin (Lubell et al., 2005; Figure 4). To *O*-*tert*-butyl d-glutamyl-d-leucinyl-d-alanine Rink amide resin **21**, the respective Agl and Hgl dipeptides **7** and **12** were coupled using HBTU, DIEA, and DMF to give the corresponding resin-bound pentapeptides **22** and **23**. Subsequent removals of the Fmoc protection were performed using 20% piperidine in DMF, and peptide elongation was completed by sequential couplings of *N*-Fmoc-*O-tert*-butyl-tyrosine and *N*-Boc-*N-*Pmc-arginine using HBTU and DIEA in DMF to give, respectively, the protected heptapeptide resins **24** and **25**. Resin cleavage was performed using a cocktail of 95:2.5:2.5 TFA/H_2_O/TES to furnish peptides **5** and **6** in 35–69% purities. Purification by HPLC provided peptides **5** and **6** in 8–16% overall yields (Table 2).

**Table 2:** Retention times, crude purity, final purity, yields, and mass spectrometric data for Agl and Hgl peptides **5** and **6**.

**Compound**	**RT (min)**		**Crude purity%**	**Final purity at 280 nm%**	**Yield% (>95%)**	**HRMS [M+1] in MeOH**	
	**CH_**3**_OH**	**CH_**3**_CN**				**m/z (calcd)**	**m/z (obsd)**
[(3*R*)-Agl^3^]-**1**, [(3*R*)-**5**]	8	5.6	44	>99	12	832.4676	832.4682
[(3*R*, 4*R*)-Hgl^3^]-**1**, [(3*R*, 4*R*)-**6**]	7.8	5.4	42	>99	8	848.4625	848.4525
[(3*R*, 4*S*)-Hgl^3^]-**1**, [(3*R*, 4*S*)-**6**]	7.5	5.2	69	>99	14	848.4625	848.4573
[(3*R*)-Agl^3^-(*S*)-Val]-**1**, [(3*R*, 2′*S*)-**5**]	7.6	5.3	63	>97	16	832.4676	832.4687
[(3*R*, 4*R*)-Hgl^3^-(*S*)-Val]-**1**, [(3*R*, 4*R*, 2′*S*)-**6**]	6.8	4.9	55	>99	13	848.4625	848.4629
[(3*R*, 4*S*)-Hgl^3^-(*S*)-Val]-**1**, [(3*R*, 4*S*, 2′*S*)-**6**]	6.9	4.9	52	>99	13	848.4625	848.4529
[(3*S*)-Agl^3^]-**1**, [(3*S*)-**5**]	8	5.4	42	>99	15	832.4676	832.4680
[(3*S*, 4*R*)-Hgl^3^]-**1**, [(3*S*, 4*R*)-**6**]	7.8	5.4	42	>99	11	848.4625	848.4625
[(3*S*, 4*S*)-Hgl^3^]-**1**, [(3*S*, 4*S*)-**6**]	7.3	5.2	35	>99	9	848.4625	848.4627
[(3*S*)-Agl^3^-(*S*)-Val]-**1**, [(3*S*, 2′*S*)-**5**]	7.9	5.5	56	>99	13	832.4676	832.4676
[(3*S*, 4*R*)-Hgl^3^-(*S*)-Val]-**1**, [(3*S*, 4*R*, 2′*S*)-**6**]	7	4.9	39	>99	12	848.4625	848.4618
[(3*S*, 4*S*)-Hgl^3^-(*S*)-Val]-**1**, [(3*S*, 4*S*, 2′*S*)-**6**]	6.6	5.2	45	>99	12	848.4625	848.4635

*Isolated purity ascertained by LC-MS using gradients of 10–90% A [H_2_O (0.1% FA)/MeOH (0.1% FA)] or B [H_2_O (0.1% FA)/MeCN (0.1% FA)] over 14 min*.

### Circular Dichroism Spectra

Understanding the relationships between the preferred conformations of peptides **1**, **5**, and **6** and their modulator activity is critical toward the design of improved prototypes with therapeutic potential. In the absence of crystallographic data of their complex with IL-1R, circular dichroism (CD) spectroscopy of peptides **1**, **5**, and **6** in water were first measured to begin examining the influences of Agl-Val and Hgl-Val dipeptide configuration on conformation. The conformers in water likely represent the geometry that initially binds the receptor. Restraint that pre-organizes a favorable binding conformer may facilitate molecular recognition by diminishing the entropy costs for folding.

**Figure 5:**
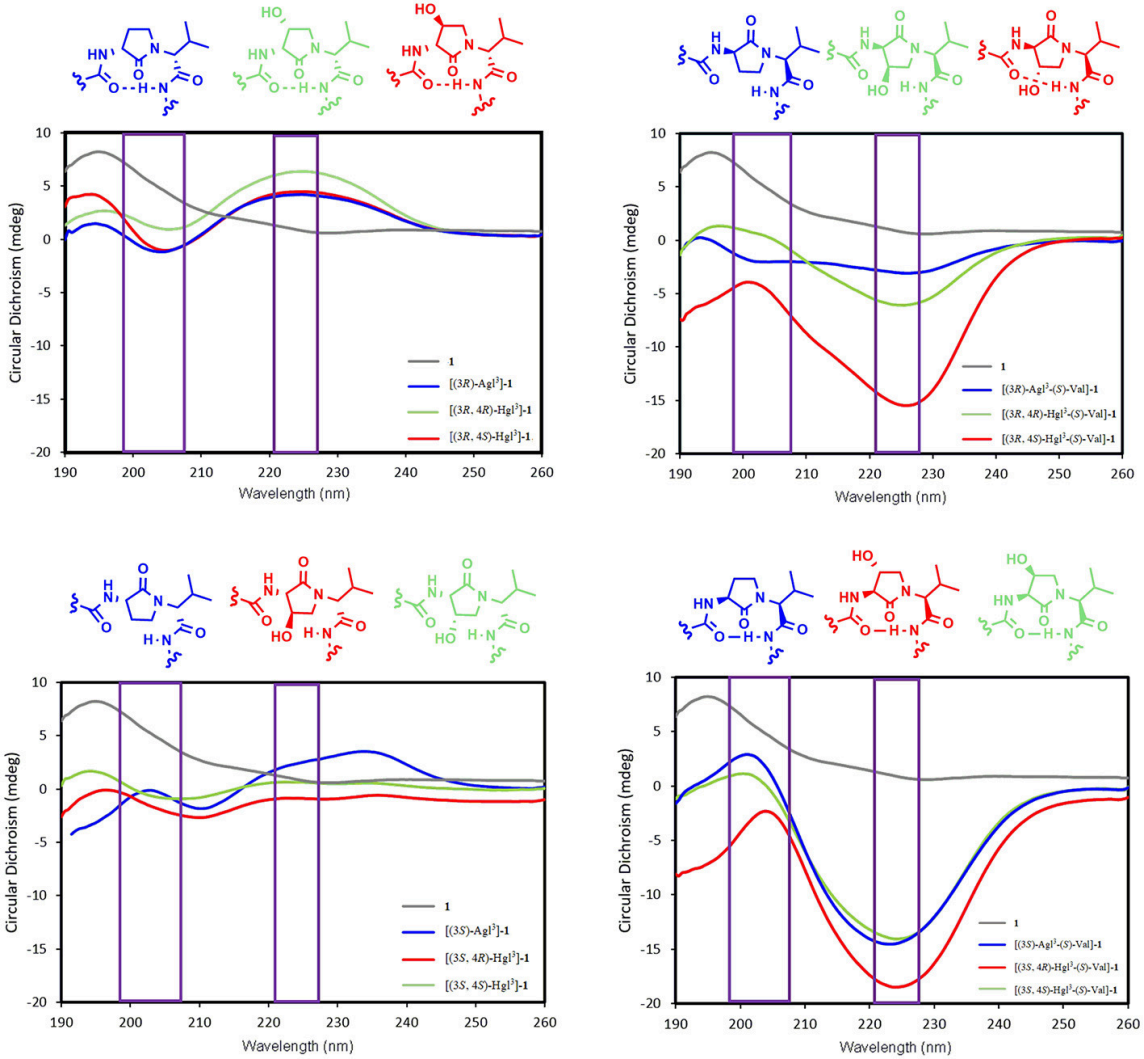
The molar ellipticity circular dichroism spectra of peptides **1**, **5**, and **6**.

The CD spectrum of the parent peptide **1** exhibited a curve shape characteristic of a disordered random coil structure (Figure 5). Contingent on stereochemistry, certain Agl and Hgl analogs exhibited CD spectroscopic curve shapes characteristic of ideal β-turn peptides. For example, analogs with *R*,*R*-backbone conformation including [(3*R*)-Agl^3^]-, [(3*R*,4*R*)-Hgl^3^]- and [(3*R*,4*S*)-Hgl^3^]-**1**, all exhibited negative and positive maximum that were, respectively, observed at 198–207 and 221–227 nm, indicative of a β-turn conformation (Figure 5; Bush et al., 1978; Kelly et al., 2005). Inversion of the stereochemistry of both backbone centers in [(3*S*)-Agl^3^-(*S*)-Val^4^]-, [(3*S*,4*S*)-Hgl^3^-(*S*)-Val^4^]- and [3*S*,4*R*)-Hgl^3^-(*S*)-Val^4^]-**1** gave similar but inverted curve shapes typical of β-turn conformers with notably greater ellipticity (Kelly et al., 2005). On the other hand, among the Agl and Hgl analogs with mixed backbone stereochemistry, only [(3*R*,4*S*)-Hgl^3^-(*S*)-Val^4^]-**1** exhibited a curve shape indicative of a β-turn conformer (Kelly et al., 2005). A similar β-turn conformer curve shape was observed in CD spectra of [(3*R*,4*S*)-Hgl^3^]-**1** examined in trifluoroethanol (TFE), MeOH and hexafluoroisopropanol (HFIP), and the greatest ellipticity was seen in 5% TFE in water.

### *In vitro* Inhibition of Signaling Pathways

**Figure 6:**
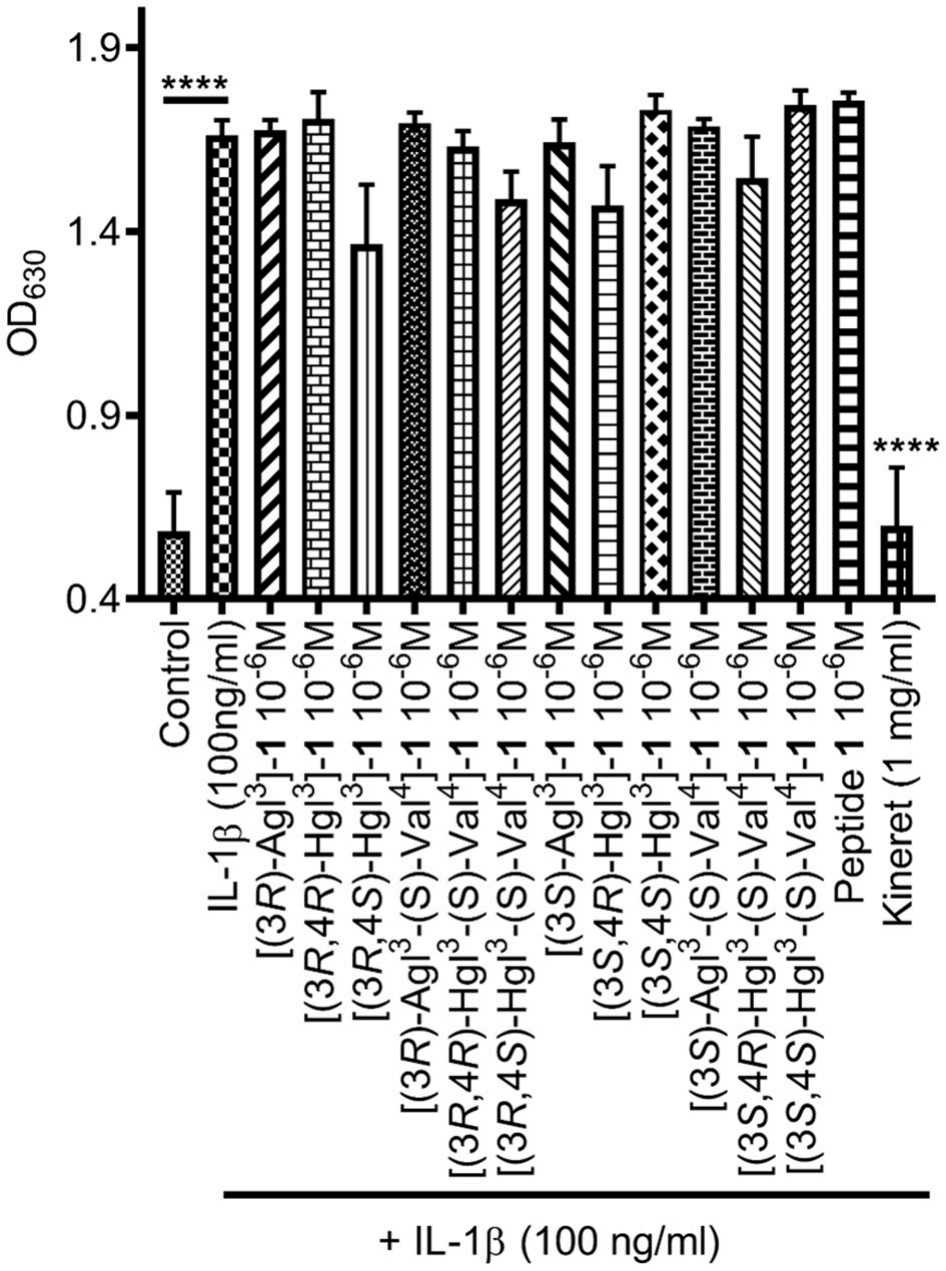
The effects of peptides **1**, **5**, and **6** on IL-1-induced NF-κB signaling. NF-κB activation was quantified on HEK-blue cells using the QUANTI-blue assay, which spectroscopically detects secreted alkaline phosphatase, a reporter product from the transcription of the NF-κB gene. HEK-blue cells were pre-incubated with peptides **1**, **5**, and **6** or vehicle, then stimulated with IL-1β for 4 h. Data shown represents the average of 3 experiments, each with *n* = 4 per treatment group. ^****^p < 0.0001 compared to group treated only with IL-1β. Treatment groups that are not labeled with asterisks are statistically non-significant compared to group treated only with IL-1β.

The biological effects of peptide **1** and derivatives were ascertained *in vitro* in RAW-blue and HEK-blue cells which were stimulated with IL-1β. The QUANTI-blue assay was employed to measure concentrations of secreted alkaline phosphatase, a reporter product from the transcription of the NF-κB gene. No peptides that were tested exhibited any noticeable inhibition of NF-κB signaling (Figure 6). On the other hand, Kineret, which is an FDA-approved recombinant IL-1 receptor antagonist, inhibited NF-κB as previously reported (Nadeau-Vallée et al., 2015).

**Figure 7:**
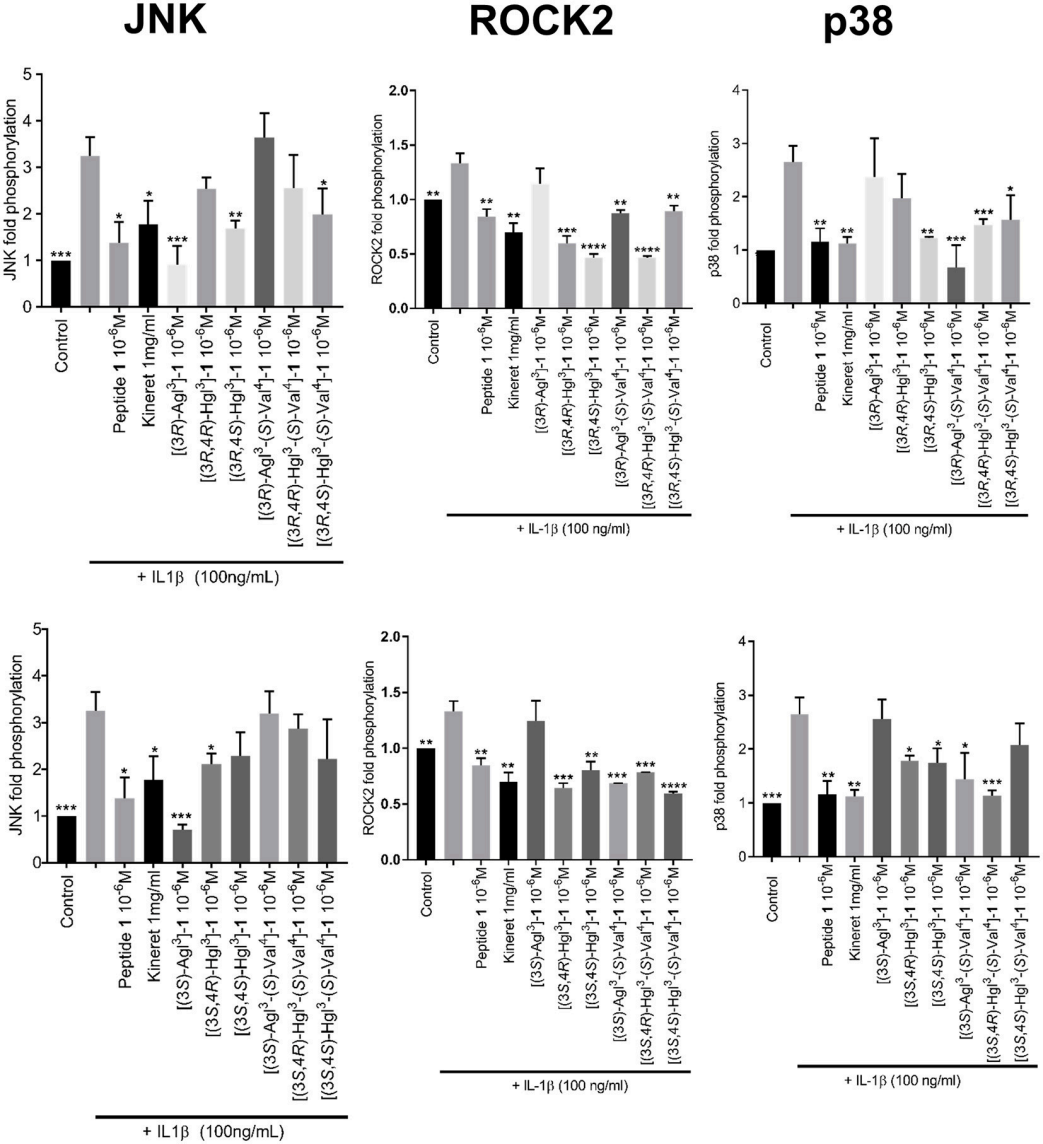
The effects of peptides **1**, **5**, and **6** on IL-1β-induced phosphorylation of JNK, ROCK2, and p38. Graphical representations of band density analysis of Western Blots, sorted into columns based on protein of interest (JNK, ROCK2, or p38) and rows by peptide configuration [(3*R-*) or (3*S*)]. RAW-blue cells were pretreated with peptides **1**, **5**, **6**, Kineret, or vehicle for 30 min and then stimulated with IL-1β for 15 min. Images of representative Western Blots can be found in the Supplementary Figure 1. Results shown are the average of 3 independent experiments: ^*^p < 0.05, ^**^p < 0.01, ^***^p < 0.001, ^****^p < 0.0001 compared to group treated only with IL-1β. Treatment groups that are not labeled with asterisks are statistically non-significant compared to group treated only with IL-1β.

Western Blots were performed to measure phosphorylation of downstream c-Jun *N*-terminal kinases (JNK), p38 mitogen-activated protein kinases (p-38) and Rho-associated, coiled-coil-containing protein kinase 2 (ROCK2) in RAW-blue cells, after pre-incubation with peptides **1**, **5**, or **6** and stimulation with IL-1β (Figure 7, Data Sheet 1). In contrast to peptide **1** which inhibited the effects of all three kinases, the Agl and Hgl analogs exhibited biased signaling contingent on stereochemistry and structure. For example, JNK phosphorylation was inhibited more profoundly by (*r*)- than (*s*)-Val^4^ derivatives **5** and **6**. In the (*r*)-Val series, the Agl and *trans*-Hgl isomers were more effective than the *cis*-Hgl counterparts. Phosphorylation of p-38 was inhibited most effectively by [(3*R*,4*S*)-Hgl^3^]- and [(3*R*)-Agl^3^-(*s*)-Val^4^]-**1**; [(3*S*)-Agl^3^]-, [(3*R*)-Agl^3^]-, [(3*R*,4*R*)-Hgl^3^]- and [(3*S*,4*S*)-Hgl^3^-(*s*)-Val^4^]-**1** were inactive. Finally, ROCK2 phosphorylation was inhibited significantly (*p* < 0.05) by most derivatives, except for [(3*S*)-Agl^3^-(*r*)-Val^4^]- and [(3*R*)-Agl^3^-(*r*)-Val^4^]-**1**. In contrast to peptide **1** which inhibited effectively all three kinases, the inhibitory activity of the Agl and Hgl derivatives was contingent on configuration and the presence of the hydroxyl group. Conversely, these factors did not play a role in NF-κB signaling, which was universally unaffected by all derivatives **5** and **6**.

**Figure 8:**
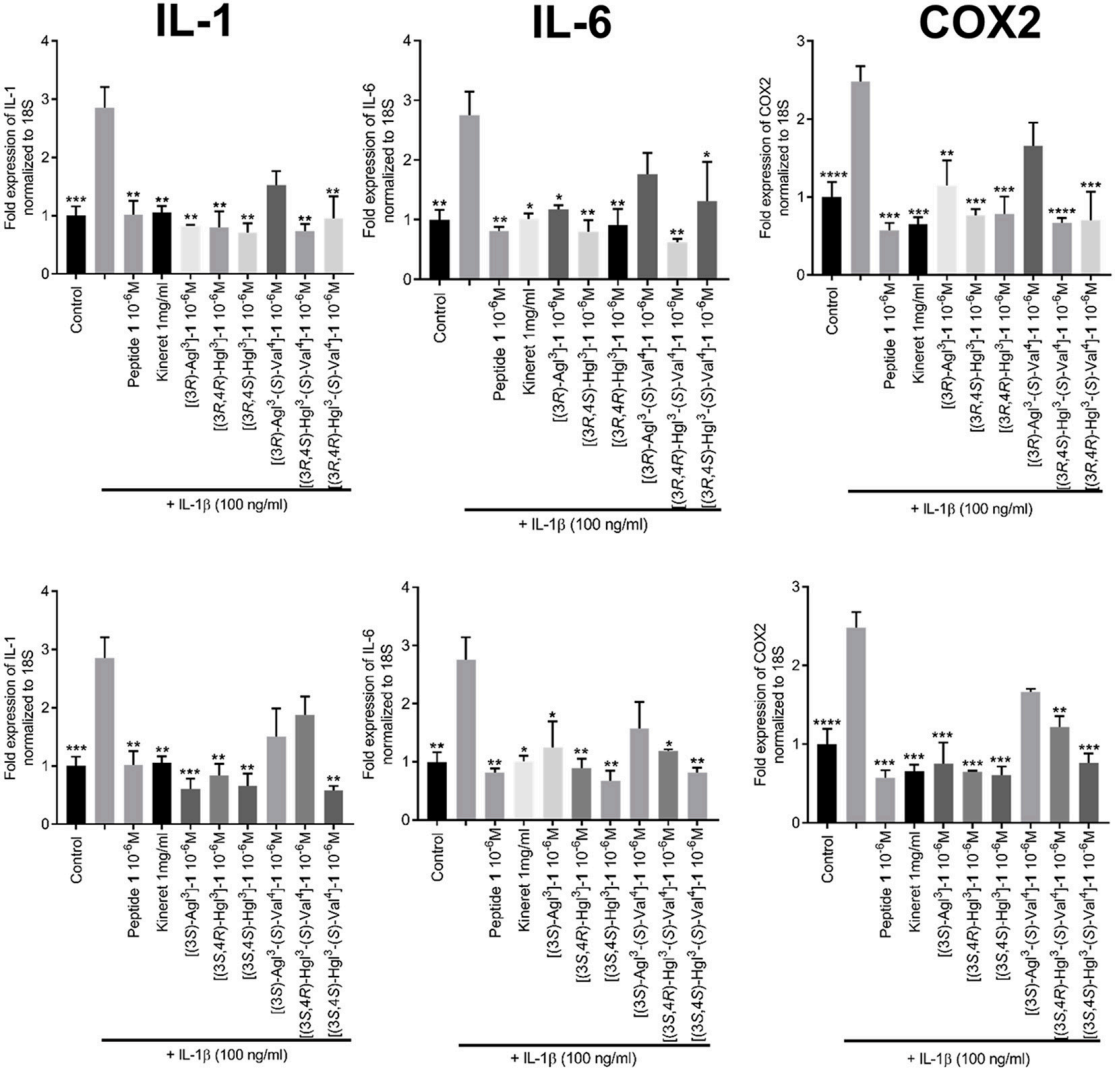
The effects of peptides **1**, **5**, and **6** on the expression of pro-inflammatory genes. HEK-Blue cells were pre-treated with peptides **1**, **5**, and **6** as above and stimulated with IL-1β overnight. qPCR was performed on cell lysates using 18S rRNA as internal control. Graphs are sorted in columns by gene of interest (COX-2, IL-1β, or IL-6) and rows by peptide configuration [(3*R*) or (3*S*)]. Results are representative of an average of 3 independent experiments (each with *n* = 4 per treatment group) and are expressed as a fold-change of the non-stimulated control: ^*^p < 0.05, ^**^p < 0.01, ^***^p < 0.001, ^****^p < 0.0001 compared to group treated only with IL-1β. Treatment groups that are not labeled with asterisks are statistically non-significant compared to group treated only with IL-1β.

The effects of peptides **1**, **5**, and **6** on the transcription of downstream pro-inflammatory genes that are mediated by IL-1β were measured with focus on IL-6, cyclooxygenase-2 (COX2) and IL-1β, which positively increases expression of itself (Weber et al., 2010). Quantitative polymerase chain reaction (qPCR) experiments were performed on HEK blue cells to ascertain the expression levels of the mRNA transcripts after pre-treatment with peptides **1**, **5**, and **6** and stimulation with IL-1β. Parent peptide **1** exhibited strong suppression of transcription of all three genes. Moreover, Agl and Hgl analogs of **1** maintained similar inhibitory potency as the parent peptide (Figure 8). On the other hand, the ability to suppress the transcription of all three genes was lost in [(3*R*)-Agl^3^-(*S*)-Val^4^]- and [(3*S*)-Agl^3^-(*S*)-Val^4^]-**1** and recovered in part in certain Hgl^3^-(*S*)-Val^4^ analogs with [(3*S*, 4*S*)-Hgl^3^-(*S*)-Val^4^]-**1** exhibiting ability to inhibit the expression of all three genes.

### Displacement of Radiolabeled Peptide 1

Peptide **1** is known to bind to IL-1R (Quiniou et al., 2008). To determine if the Agl and Hgl derivatives occupied the same binding site as peptide **1**, a radio-ligand displacement assay was used to determine the extent to which peptides **5** and **6** displaced radiolabeled **1**. Compared to cold unlabeled peptide **1**, which was used to set the baseline for specific binding, a similar capacity to displace [^125^I]-**1** was demonstrated by peptides **5** and **6**, except for [(3*R*,4*S*)-Hgl^3^]-**1**, which exhibited a significantly lower (*p* = 0.0011) ability to compete with radiolabeled peptide **1** (Table 3).

**Table 3:** The ability of peptides **1**, **5**, and **6** to displace radio-labeled [^125^I]-**1** in RAW-blue cells.

**Compound**	**Percentage of displacement of [^**125**^I]-1, relative to unlabeled peptide 1, ±SEM**	***p*-Value**
[(3*R*)-Agl^3^]-**1**	83.55 ± 9.02	0.9908
[(3*R*,4*S*)-Hgl^3^]-1^*^	32.13 ± 16.94	0.0011
[(3*R*,4*R*)-Hgl^3^]-**1**	104.6 ± 6.826	0.9997
[(3*R*)-Agl^3^-(*S*)-Val^4^]-**1**	120.7 ± 15.69	0.954
[(3*R*,4*S*)-Hgl^3^-(*S*)-Val^4^]-**1**	58.63 ± 13.45	0.0724
[(3*R*,4*R*)-Hgl^3^-(*S*)-Val^4^]-**1**	82.12 ± 14.12	0.9308
[(3*S*)-Agl^3^]-**1**	106.6 ± 12.39	0.9996
[(3*S*,4*R*)-Hgl^3^]-**1**	78.64 ± 21.22	0.9428
[(3*S*,4*S*)-Hgl^3^]-**1**	69.73 ± 8.897	0.2864
[(3*S*)-Agl^3^-(*S*)-Val^4^]-**1**	102.7 ± 8.637	0.9999
[(3*S*,4*R*)-Hgl^3^-(*S*)-Val^4^]-**1**	48.32 ± 21.13	0.0649
[(3*S*,4*S*)-Hgl^3^-(*S*)-Val^4^]-**1**	86.06 ± 6.46	0.9806

* *p < 0.05 relative to cold peptide **1-**treated group*.

### *In vivo* Inhibition of Preterm Labor

**Figure 9:**
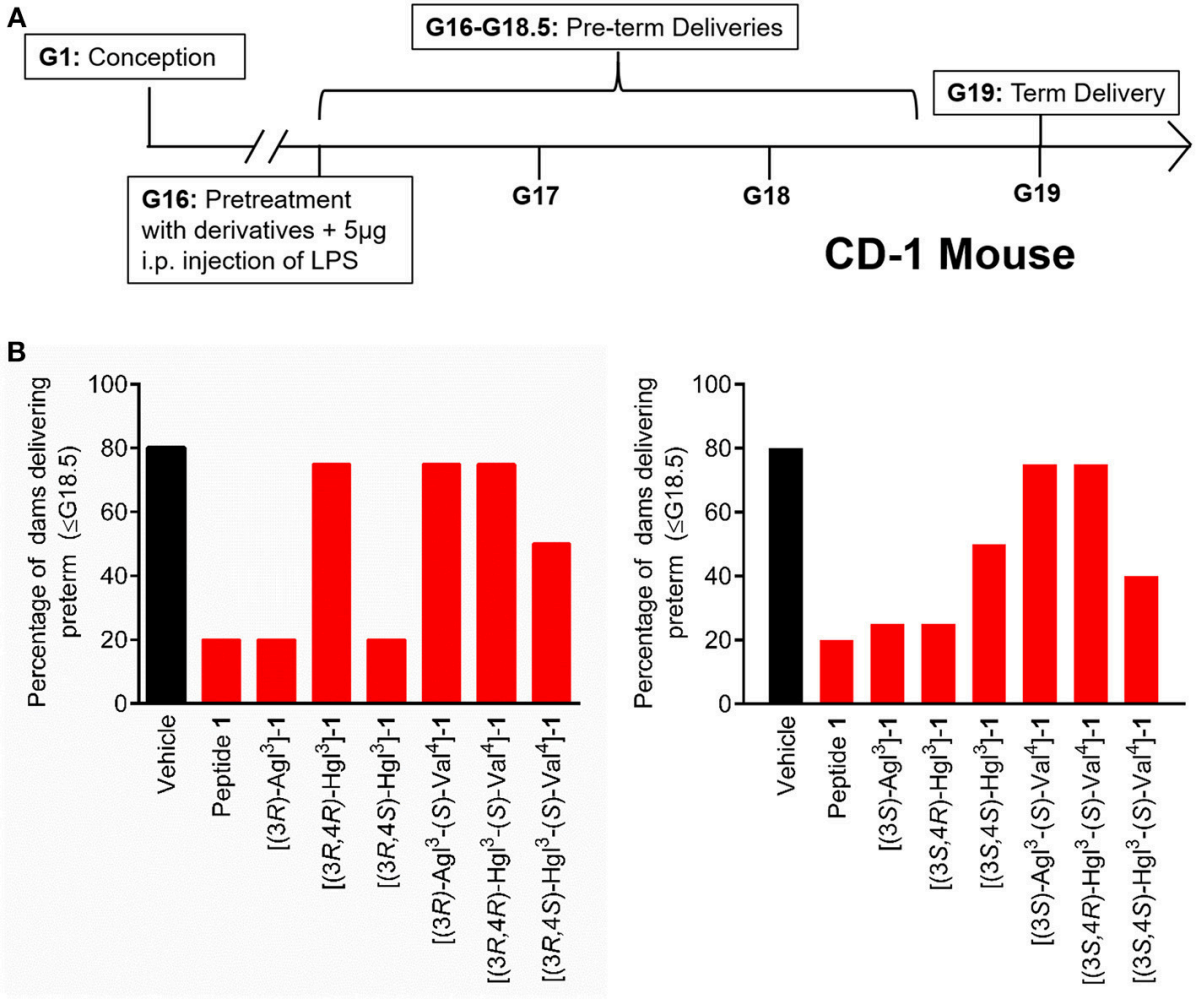
The effects of peptides **1**, **5**, and **6** on prevention of PTB. **(A)** Schematic of the CD-1 mouse PTB model. In brief, pregnant dams on day 16.5 of gestation (G16.5) were subcutaneously pretreated with peptides **1**, **5**, and **6** or vehicle, followed by LPS, and observed for delivery of pups. A dam was considered as delivering preterm if at least one pup was delivered before G18.5. **(B)** The rates of PTB in dams treated with peptides **1**, **5**, and **6** are grouped into (3*R*)- (left) and (3*S*)- (right) derivatives; *n* = 4–5 dams per treatment group.

The spectrum of *in vitro* profiles exhibited by peptides **5** and **6** offers a unique means for probing the specific signaling pathways that contribute to the therapeutic potential of IL-1R modulators. Peptides **1**, **5**, and **6** were first examined in an established CD-1 mouse model of preterm birth (PTB, Figure 9A), featuring induction on administration of lipopolysaccharide (LPS) by intraperitoneal injection into pregnant dams on day 16.5 of gestation. The Agl and *trans*-Hgl analogs of peptide **1** exhibited equal potency as the parent peptide in delaying labor (Figure 9B). On the other hand, *cis*-Hgl derivatives of peptide **1** and (*S*)-Val peptides **5** and **6** exhibited little efficacy.

### *In vivo* Inhibition of Vaso-Obliteration in Oxygen-Induced Retinopathy

A model of OIR was next used to examine peptide **1** and a subset of analogs **5** and **6** that were previously tested in the PTB model: e.g., [(3*S*)-Agl^3^]-, [(3*R*)-Agl^3^]-, [(3*S*,4*R*)-Hgl^3^]-, and [(3*R*,4*S*)-Hgl^3^]-**1** which exhibited the best activity, [3*S*,4*S*-Hgl^3^]-**1** which had partial efficacy and [3*S*,4*R*-Hgl^3^-(*S*)-Val^4^]-**1**, which was inactive in delaying labor.

**Figure 10:**
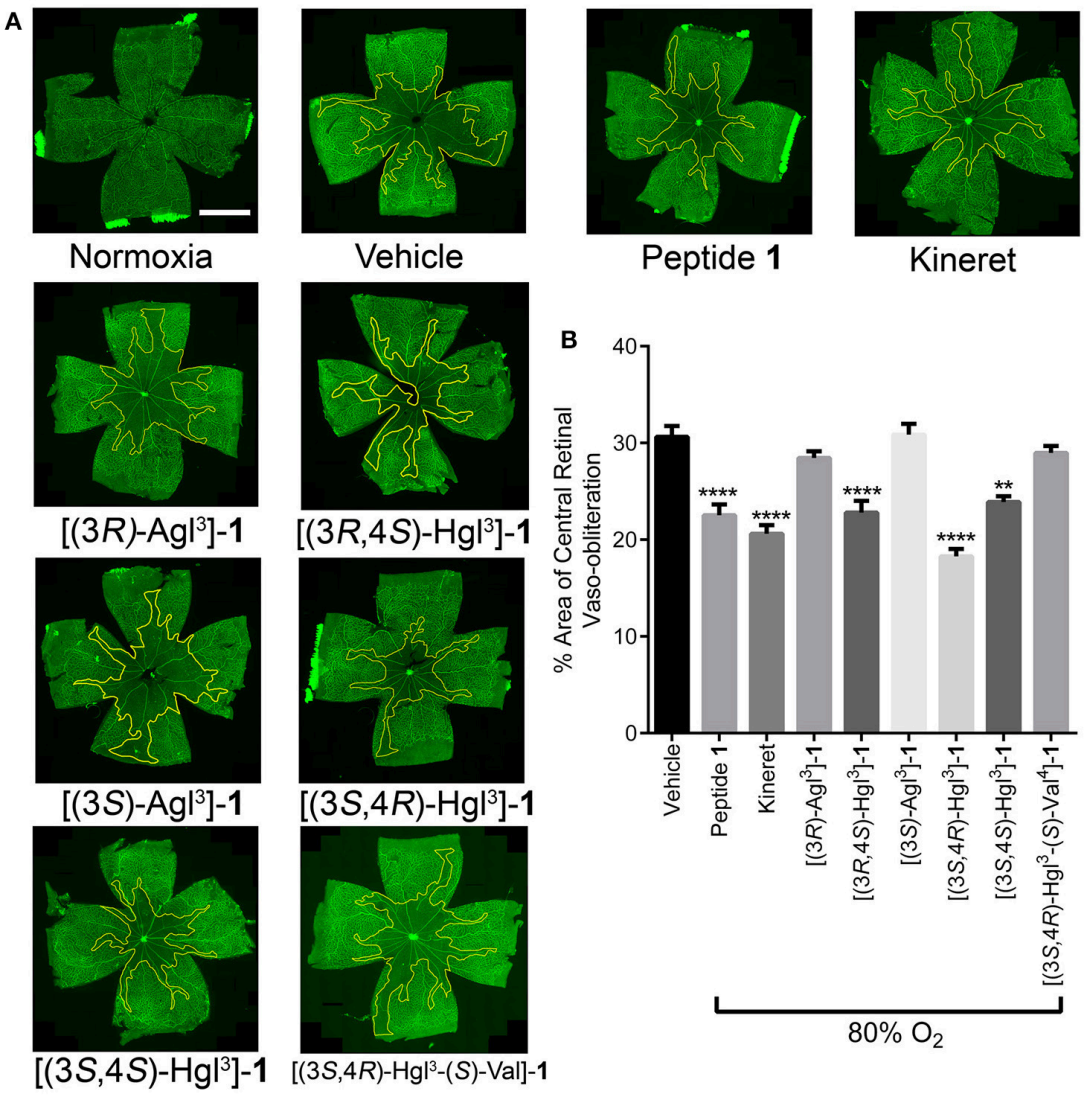
The preventive effects of peptides **1**, **5**, and **6** against vaso-obliteration in an OIR model. Five-days old Sprague Dawley pups and their mothers were kept in 80% oxygen until the 10 day of life, receiving twice-daily intraperitoneal injections of peptides **1**, **5**, and **6** (2 mg/kg/day), Kineret (15 mg/kg/day) or PBS vehicle (each injection was titrated to a volume of 20 μL). **(A)** Representative retinal flatmounts stained with FITC-conjugated *Bandeiraea simplicifolia* lectin, taken at 10X magnification and stitched with MosaiX in Axiovision 4.8. Yellow lines indicate the central area of vaso-obliteration extending from the optic nerve (center of each retina), scale bar 2 mm. **(B)** Quantification of area of vaso-obliteration performed using ImageJ, expressed as a percentage of the total retinal area: *n* = 5–7 of peptides **5** and **6** and Kineret, *n* = 10–12 for vehicle and peptide **1**; ^**^p < 0.01, ^****^p < 0.0001 relative to the vehicle group. Treatment groups that are not labeled with asterisks are statistically non-significant compared to the vehicle group.

Exposure of rat pups to 80% oxygen from days 5 to 10 of life resulted usually in vaso-obliteration of ~35% of the retinal capillaries, extending radially from the optic nerve (Figures 10A,B). Peptide **1** and Kineret both diminished the extent of vaso-obliteration to 15–20% (Figure 10B; Rivera et al., 2013). Among the four peptides that were strongly effective in the PTB model, only two, [3*R*,4*S*-Hgl^3^]- and [3*S*,4*R*-Hgl^3^]-**1** exhibited efficacy in the OIR model and reduced vaso-obliteration to 15–25%. Furthermore, [3*S*,4*S*-Hgl^3^]- **1**, which was moderately effective (~50% efficacy) in the PTB model, demonstrated efficacy in reducing vaso-obliteration to a somewhat lesser extent than peptide **1**. On the other hand, [(3*S*,4*R*)-Hgl^3^-(*S*)-Val^4^]-**1**, which had no effect in the PTB model, was also ineffective in curbing vaso-obliteration.

**Figure 11:**
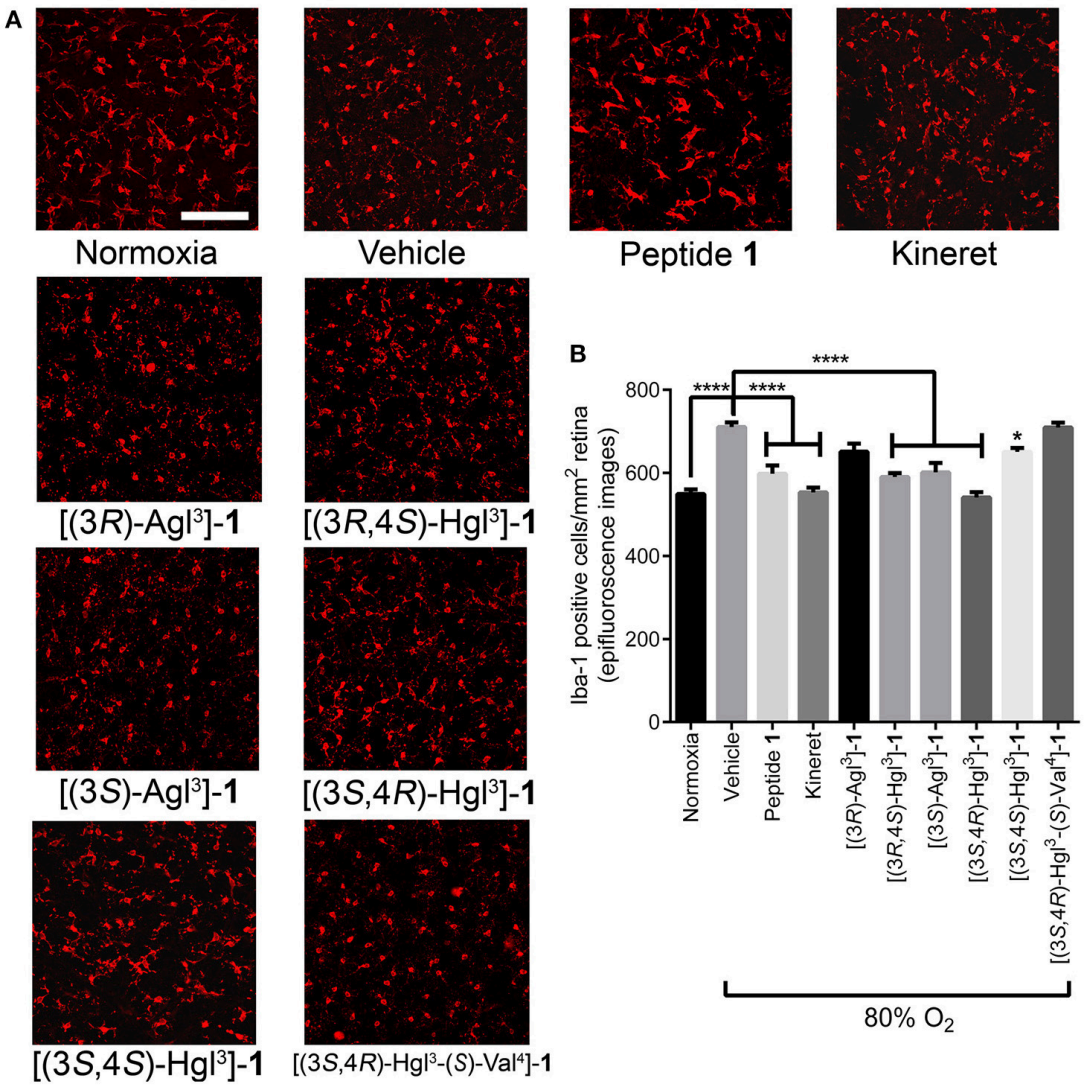
The effects of peptides **1**, **5**, and **6** on retinal microglial activation and density. Retinas were obtained for immunohistochemistry from rat pups treated with the OIR protocol, and incubated with rabbit anti-iba-1 antibody, followed by donkey anti-rabbit antibody conjugated to Alexa 594. **(A)** Representative confocal images of retinal microglia at 30X magnification: scale bar 100 μm. **(B)** Epifluorescence microscopy images at 20 × magnification of retinal microglial density quantified using ImageJ: 4 images per retina were taken at a distance halfway between the optic nerve and the peripheral edge of the retina; *n* = 5–7 for peptide **1**, **5**, and **6**, and Kineret; *n* = 8–10 for normoxia and vehicle; ^*^p < 0.05, ^****^p < 0.0001 relative to the vehicle group. Treatment groups that are not labeled with asterisks are statistically non-significant compared to the vehicle group.

Immuno-histochemical staining for Iba-1 was used to assess microglial activation and density, because microglia have been shown to be mediators of vaso-obliteration in the context of OIR (Rivera et al., 2013). Microglia morphology has been observed to change at different states of activation: microglia that are inactive and ramified possess numerous branches, which were observed in retina under normoxia and after treatment with peptide **1** (Figure 11A); on the other extreme, activated microglia retract their limbs and become amoeboid (Donat et al., 2017), as observed in vehicle-treated retina under hypoxia. Peptides [(3*S*,4*R*)-Hgl^3^]-, [(3*R*,4*S*)-Hgl^3^]- and [(3*S*,4*S*)-Hgl^3^]-**1** prevented partially the activation of microglia; [(3*S*)-Agl^3^]-, [(3*R*)-Agl^3^]- and [(3*S*,4*R*)-Hgl^3^-(*S*)-Val^4^]-**1** had no appreciable effect on microglial morphology (Figure 11A). Quantification of microglial density revealed similar trends (Figure 11B), except for [(3*S*)-Agl^3^]-**1**, which modestly (< 20%) reduced microglial density despite not influencing microglial morphology. Peptides **1**, **5**, and **6**, which prevented microglia activation, caused a statistically significant reduction in vaso-obliteration area. In summary, certain [Hgl^3^]-**1** analogs behaved like the parent peptide and exhibited protection against vaso-obliteration in the hyperoxic phase of OIR, due in part to mitigation of microglial activation.

## Discussion

PTB and ROP are medical conditions strongly associated with dysregulated inflammation. Current treatments for PTB, such as oxytocin antagonists and indomethacin, are targeted at reducing the contractility of the myometrium (tocolysis) but fail to address the underlying inflammatory processes responsible for labor (Olson et al., 2008). Current ROP treatments employ anti-vascular endothelial growth factor (VEGF) antibodies and laser photocoagulation (Hellström et al., 2013) which treat the proliferative phase of the disease but fail to address earlier stage vaso-obliteration and associated inflammation. Modulation of IL-1R signaling offers the potential to mitigate both PTB and ROP as indicated by the *in vivo* results herein and previously reported (Rivera et al., 2013; Nadeau-Vallée et al., 2015, 2017; Beaudry-Richard et al., 2018).

Peptide **1** was shown to adopt a random coil CD spectrum. In the *in vitro* assays, peptide **1** exhibited inhibitory activity on the phosphorylation of the three kinases (JNK, p-38, and ROCK2) and on the transcription of downstream pro-inflammatory genes that are mediated by IL-1β (IL-6, COX2, and IL-1β), but did not affect NF-κB signaling. Moreover, peptide **1** delayed significantly PTB in mice induced with LPS and reduced vaso-obliteration in the OIR murine model.

Conformational constraint of peptide **1** was performed by replacing (2*R*,3*S*)-Thr^3^-(*R*)-Val^4^ with all four possible Agl^3^-Val^4^ (e.g., **5**) and eight possible Hgl^3^-Val^4^ (e.g., **6**) diastereomers. Among the twelve analogs of peptide **1**, those possessing common backbone stereochemistry (e.g., *R*,*R*- and *S*,*S*-) exhibited circular dichroism spectra indicative of β-turn conformers in water: [(3*R*)-Agl^3^]-, [(3*R*,4*R*)-Hgl^3^]- and [(3*R*,4*S*)-Hgl^3^]-, [(3*S*)-Agl^3^-(*S*)-Val^4^]-, [(3*S*,4*S*)-Hgl^3^-(*S*)-Val]-, [(3*S*,4*R*)-Hgl^3^-(*S*)-Val]-**1**. Moreover, [(3*R*,4*S*)-Hgl^3^-(*S*)-Val]-**1** also exhibited a CD curve indicative of a turn conformer.

Among the constrained analogs, [(3*R*,4*S*)-Hgl^3^]-**1** exhibited the most similar activity as the parent peptide in the *in vitro* and *in vivo* assays with slightly reduced potency in inhibiting JNK. Moreover, [(3*S*,4*R*)-Hgl^3^]-**1** was also typically as potent as **1** but had slightly reduced abilities in inhibiting p38 and ROCK2. Although their CD spectra and conformers differed in water, both isomers possess *trans*-Hgl residues and (*R*)-Val stereochemistry, indicating the importance of the β-hydroxyl group and gauche-(–) χ-dihedral angle side chain geometry for maximum activity.

The contrast of high potency and inactivity exhibited in the PTB and OIR models, respectively, by both [(3*R*)-Agl^3^]- and [(3*S*)-Agl^3^]-**1** correlates with their ability to block JNK without inhibitory potency on p-38 and ROCK2. The importance of the hydroxyl group for activity on the latter kinases and for ability to reduce vaso-obliteration in the OIR model is illustrated by the potency of the corresponding Hgl analogs. Notably, [(3*S*,4*S*)-Hgl^3^]-**1**, which positions the hydroxyl group in a gauche-(+) χ-dihedral angle side chain orientation, maintains some potency on all three kinases—with best activity on ROCK2—and exhibits moderate and strong activities in the PTB and OIR models, respectively.

Some inhibitory activity on JNK appears necessary for potency in delaying labor in the PTB model. For example, [(3*R*,4*R*)-Hgl^3^]-**1**, which inhibited strongly ROCK2 but had no effects on JNK and p-38, was inactive in the PTB model. The weaker potency in the PTB assay of the (*S*)-Val analogs correlated with their lack of inhibitory activity on JNK. Although (*R*)- instead of (*S*)-Val may be a prerequisite for binding IL-1R, the latter may also be more susceptible to enzymatic cleavage by proteases (Carmona et al., 2013 and Najjar et al., 2017).

Correlations between peptide structure, *in vitro* activity and *in vivo* potency highlight the relative importance of blocking specific IL-1 signaling pathways to treat certain pathologies (Table 4). For example, inhibition of JNK phosphorylation was most strongly correlated with effectiveness in PTB inhibition. In agreement with an earlier study in which the use of a specific JNK inhibitor had delayed PTB which was induced by a type of LPS that activated both NF-κB and JNK pathways (Pirianov et al., 2015), blocking JNK phosphorylation alone was sufficient for PTB prevention. On the other hand, efficacy in the OIR model necessitated inhibition of both JNK and ROCK2 phosphorylation. The latter was in accordance with a study demonstrating the utility of specific ROCK inhibitors in an *in vivo* model of OIR (Yamaguchi et al., 2016). Inactivity of specific compounds may be due to their pharmacokinetics and would require further study to address such issues. For example, delivery may play a role in efficacy because entrance into the retina is more challenging than the myometrium, due to the presence of a blood-retina-barrier that limits entrance from systemic circulation (del Amo et al., 2017) and may account for the enhanced activity of the Hgl relative to the Agl analogs in the OIR model.

**Table 4:** Heatmap summary of the *in vivo* and *in vitro* effects of peptides **1**, **5**, and **6**.

	**Structure**	**Western Blot**			**qPCR**			**Nf-κB**	***In vivo***	
		**JNK**	**p38**	**ROCK2**	**COX2**	**IL-1β**	**IL-6**		**PTB**	**OIR**
	[(3*R*)-Agl^3^]-**1**	4	0	0	3	4	3	0	4	0
	[(3*R*,4*R*)-Hgl^3^]-**1**	0	0	4	4	4	4	0	0	
	[(3*R*,4*S*)-Hgl^3^]-**1**	4	4	4	4	4	4	0	4	4
	[(3*R*)-Agl^3^-(*S*)-Val^4^]-**1**	0	4	2	1	1	1	0	0	
	[(3*R*,4*R*)-Hgl^3^-(*S*)-Val^4^]-**1**	1	3	4	4	3	4	0	0	
	[(3*R*,4*S*)-Hgl^3^-(*S*)-Val^4^]-**1**	2	2	2	4	4	2	0	2	
	[(3*S*)-Agl^3^]-**1**	4	0	0	4	4	2	0	4	1
	[(3*S*,4*R*)-Hgl^3^]-**1**	3	3	3	4	4	4	0	4	4
	[(3*S*,4*S*)-Hgl^3^]-**1**	1	1	3	4	4	4	0	2	3
	[(3*S*)-Agl^3^-(*S*)-Val^4^]-**1**	0	2	3	1	1	1	0	0	
	[(3*S*,4*R*)-Hgl^3^-(*S*)-Val^4^]-**1**	0	3	3	3	1	3	0	0	0
	[(3*S*,4*S*)-Hgl^3^-(*S*)-Val^4^]-**1**	2	0	4	4	4	4	0	2	
Peptide **1**		4	4	4	4	4	4	0	4	4
Kineret		4	4	4	4	4	4	4	0	3

*Black = not tested*.

*No effect 01234 Maximum inhibition/efficacy*.

Peptide **1** was previously shown to bind to IL-1R (Quiniou et al., 2008). Most of peptides **5** and **6** displaced radio-labeled **1** to the same extent as cold peptide **1**, suggesting they all compete for the same binding site on IL-1R, though not necessarily with the same binding affinity. The sole exception was [(3*R*,4*S*)-Hgl^3^]-**1**, which displaced radio-labeled **1** to a lesser extent despite being the most efficacious molecule *in vitro* and *in vivo* (Table 4). This contradiction may be due to a different IL-1R binding pattern that retains desirable biased signaling and may require crystallographic analyses to confirm this hypothesis.

Compared to the larger proteins and non-selective competitive inhibitors currently used in anti-IL-1 therapies, peptides **1**, **5**, and **6** may offer benefits such as ease of administration, as well as the potential to reduce immunosuppression, immunogenicity and related side effects. The small peptides may be further optimized for oral administration, in contrast to antibodies, which must be administered by injection, predisposing patients to potentially unpleasant injection-site reactions leading to reduced patient compliance. Notably, anti-VEGF antibodies, a mainstay of ROP treatment, must be injected directly into the vitreous of the eye (i.e., intravitreally), a technique that is invasive and technically demanding (del Amo et al., 2017).

To date, there are no clinically-approved allosteric inhibitors of IL-1 or its receptor. The anti-IL-1β antibody Gevokizumab, which is currently under investigation, has been reported to bind to an allosteric site on IL-1β (Blech et al., 2013; Issafras et al., 2014); however, like other antibodies it may be associated with similar drawbacks including large molecule size and high production costs.

## Conclusion

The utility of conformational constraint to create folded synthetic peptides has for the first time been studied in the context of cytokine signaling and inflammation. Toward the development of immunomodulatory therapy, lactam constraint has been used to study the central d-Thr-d-Val dipeptide sequence of the allosteric IL-1R modulator peptide **1**, which has previously exhibited efficacy in curbing inflammation in various models of disease, due in part to ability to maintain the beneficial effects of NF-κB signaling (Castro-Alcaraz et al., 2002; Gerondakis and Siebenlist, 2010; Nadeau-Vallée et al., 2015). Although, the lactam analogs behaved like parent peptide **1** and did not inhibit NF-κB, they exhibited different degrees of inhibitory potency on the kinases and cytokines activated by IL-1β *in vitro*. Lactam analogs **5** and **6** are thus valuable probes for identifying specific inflammation-induced signaling pathways for intervention to treat specific medical conditions. Specifically, inhibition of JNK alone and in combination with ROCK2 were, respectively, identified for delaying preterm labor and mitigating retinopathy of prematurity. Peptides **1**, **5**, and **6** comprise a valuable set of selective probes for studying IL-1 signaling pathways in various inflammatory diseases. As promising leads for immune modulator therapy, they offer benefits over traditional protein-based counterparts due to biased signaling, which may avoid immunosuppression. The demonstrated methods for utilizing a combination of Agl and Hgl residues to make folded peptides may thus have broad utility for biomedical research.

## Author Contributions

AG wrote the manuscript, synthesized and purified compounds, and conducted circular dichroism analyses. CC wrote the manuscript and conducted *in vivo* and *in vitro* experiments. CQ and TZ edited the manuscript and conducted *in vitro* experiments. XH and JCR edited the manuscript and conducted *in vivo* experiments. CQ, DJS-C, KB, and VB-G conceptualized rytvela (peptide **1**) and assisted in synthesis of derivatives. SC and WL supervised the progress of the project, edited, and proofread the manuscript. All authors have read the final manuscript and agree to be accountable for the content of this work.

### Conflict of Interest Statement

The authors declare that the research was conducted in the absence of any commercial or financial relationships that could be construed as a potential conflict of interest.
