# Supplementary material for: Probing Anti-inflammatory Properties Independent of NF-κB Through Conformational Constraint of Peptide-Based Interleukin-1 Receptor Biased Ligands
Source: Front Chem. 2019 Feb 13;7:23. doi: 10.3389/fchem.2019.00023 (PMC6381024; doi:10.3389/fchem.2019.00023)
Supplement: Supplementary file 1 [file Data_Sheet_1.pdf]

Supplementary Material

Probing anti-inflammatory properties independent of NF-*κ*B through conformational constraint of peptide-based interleukin-1β receptor biased ligands

Azade Geranurimi, Colin WH Cheng, Christiane Quiniou, Xin Hou, Tang Zhu, Daniel St-Cyr, Kim Beauregard, Vadim Bernard-Gauthier, Sylvain Chemtob, William D. Lubell*

*** Correspondence:** Prof. William D. Lubell, *william.lubell@umontreal.ca*

Table of Contents

Synthesis of:

[(3*R*)-Agl^3-(*S*)-Val]-1 **[(3*R*, 2'*S*)-5]**....4

[(3*S*)-Agl^3]-1 **[(3*S*)-5]**....4

[(3*S*)-Agl^3-(*S*)-Val]-1 **[(3*S*, 2'*S*)-5]**....4

[(3*R*, 4*R*)-Hgl^3]-1 **[(3*R*, 4*R*)-6]**....5

[(3*R*, 4*S*)-Hgl^3]-1 **[(3*R*, 4*S*)-6]**....5

[(3*R*, 4*R*)-Hgl^3-(*S*)-Val]-1 **[(3*R*, 4*R*, 2'*S*)-6]**....5

[(3*R*, 4*S*)-Hgl^3-(*S*)-Val]-1 **[(3*R*, 4*S*, 2'*S*)-6]**....5

[(3*S*, 4*R*)-Hgl^3]-1 **[(3*S*, 4*R*)-6]**....5

[(3*S*, 4*S*)-Hgl^3]-1 **[(3*S*, 4*S*)-6]**....6

[(3*S*, 4*R*)-Hgl^3-(*S*)-Val]-1 **[(3*S*, 4*R*, 2'*S*)-6]**....6

[(3*S*, 4*S*)-Hgl^3-(*S*)-Val]-1 **[(3*S*, 4*S*, 2'*S*)-6]**....6

(3*R*, 2'*S*)-2-[3-*N*-(Fmoc)amino-2-oxopyrrolidin-1-yl]-3-methylbutanoic acid **[(*3R, 2'S*)-7]** 6

(3*S*, 2'*R*)-2-[3-*N*-(Fmoc)amino-2-oxopyrrolidin-1-yl]-3-methylbutanoic acid **[(*3S, 2'R*)-7]** 7

(3*S*, 2'*S*)-2-[3-*N*-(Fmoc)amino-2-oxopyrrolidin-1-yl]-3-methylbutanoic acid **[(3*S*, 2'*S*)-7]** 7

N-Boc-(*R*)-Methionine **[(*R*)-8]**.. 8

N-Boc-(*S*)-Methionine **[(*S*)-8]**...8

(*R*)-*N*-(Boc)Methioninyl-(*S*)-valine tert-butyl ester **[(*R, S*)-10]**..9

(*S*)-*N*-(Boc)Methioninyl-(*R*)-valine *tert*-butyl ester **[(*S, R*)-10]**..9

(*S*)-*N*-(Boc)Methioninyl-(*S*)-valine *tert*-butyl ester **[(*S, S*)-10]**...9

N-Boc-(*R*)-Methioninyl-(*S*)-valine *tert*-butyl ester methylsulfonium iodide **[(*R, S*)-11]**......................10

N-Boc-(*S*)-Methioninyl-(*R*)-valine *tert*-butyl ester methylsulfonium iodide **[(*S, R*)-11]**......................10

N-Boc-(*S*)-Methioninyl-(*S*)-valine *tert*-butyl ester methylsulfonium iodide **[(*S, S*)-11]**.......................11

N-Fmoc-(3*R*, 4*R*, 2'*R*)-Hgl-Val-OH **[(3*R*, 4*R*, 2'*R*)-12]**..11

N-Fmoc-(3*R*, 4*S*, 2'*R*)-Hgl-Val-OH **[(3*R*, 4*S*, 2'*R*)-12]**...11

N-Fmoc-(3*R*, 4*R*, 2'*S*)-Hgl-Val-OH **[(3*R*, 4*R*, 2'*S*)-12]**...12

N-Fmoc-(3*R*, 4*S*, 2'*S*)-Hgl-Val-OH **[(3*R*, 4*S*, 2'*S*)-12]**..12

N-Fmoc-(3*S*, 4*R*, 2'*R*)-Hgl-Val-OH **[(3*S*, 4*R*, 2'*R*)-12]**...12

N-Fmoc-(3*S*, 4*S*, 2'*R*)-Hgl-Val-OH **[(3*S*, 4*S*, 2'*R*)-12]**..13

N-Fmoc-(3*S*, 4*R*, 2'*S*)-Hgl-Val-OH **[(3*S*, 4*R*, 2'*S*)-12]**..13

N-Fmoc-(3*S*, 4*S*, 2'*S*)-Hgl-Val-OH **[(3*S*, 4*S*, 2'*S*)-12]**...13

(3*R*, 2'*S*)-*tert*-Butyl 2-[3-(Boc)amino-2-oxopyrrolidin-1-yl]-3-methylbutanoate **[(3*R*, 2'*S*)-13]** 14

(3*S*, 2'*R*)-*tert*-Butyl 2-[3-(Boc)amino-2-oxopyrrolidin-1-yl]-3-methylbutanoate **[(3*S*, 2'*R*)-13]** 14

(3*S*, 2'*S*)-*tert*-Butyl 2-[3-(Boc)amino-2-oxopyrrolidin-1-yl]-3-methylbutanoate **[(3*S*, 2'*S*)-13]** 14

(3*R*, 2'*S*)-2-[3-amino-2-oxopyrrolidin-1-yl]-3-methylbutanoate trifluoroacetate **[(3*R*, 2'*S*)-14]**........15

(3*S*, 2'*R*)-2-[3-amino-2-oxopyrrolidin-1-yl]-3-methylbutanoate trifluoroacetate **[(3*S*, 2'*R*)-14]**.........15

(3*S*, 2'*S*)-2-[3-amino-2-oxopyrrolidin-1-yl]-3-methylbutanoate trifluoroacetate **[(*3S, 2'S*)-14]**.........15

N-Fmoc-(*R*)-methionine methyl ester **[(*R*)-15]**..16

N-Fmoc-(*S*)-methionine methyl ester **[(*S*)-15]**..16

N-Fmoc-(*R*)-methionine sulfoxide methyl ester **[(*R*)-16]**..16

N-Fmoc-(*S*)-methionine sulfoxide methyl ester **[(*S*)-16]**.. 17

N-Fmoc-2*R*-vinylglycine- methyl ester **[(*R*)-17]**...17

N-Fmoc-2*S*-vinylglycine- methyl ester **[(*S*)-17]**..18

(2*R*, 2'*R*)- and (2*R*, 2'*S*)-Methyl 2-(oxiranyl)-*N*-(Fmoc)glycinate **[(2*R*, 2'*R*)-18 and (2*R*, 2'*S*)-18]** 18

(2*S*, 2'*S*)- and (2*S*, 2'*R*)-Methyl 2-(oxiranyl)-*N*-(Fmoc)glycinate **[(2*S*, 2'*S*)-18 and (2*S*, 2'*R*)-18]** 19

N-Fmoc-(3*R*, 4*S*, 2'*R*)-Hgl-Val-*t*Bu **[(3*R*, 4*S*, 2'*R*)-19]**..20

N-Fmoc-(3*R*, 4*S*, 2'*S*)-Hgl-Val-*t*Bu **[(3*R*, 4*S*, 2'*S*)-19]**..20

N-Fmoc-(3*S*, 4*R*, 2'*R*)-Hgl-Val-*t*Bu **[(3*S*, 4*R*, 2'*R*)-19]**..21

N-Fmoc-(3*S*, 4*R*, 2'*S*)-Hgl-Val-*t*Bu **[(3*S*, 4*R*, 2'*S*)-19]**..21

N-Fmoc-(3*R*, 4*R*, 2'*R*)-Hgl-Val-*t*Bu **[(3*R*, 4*R*, 2'*R*)-19]**..21

N-Fmoc-(3*R*, 4*R*, 2'*S*)-Hgl-Val-*t*Bu **[(3*R*, 4*R*, 2'*S*)-19]**..22

N-Fmoc-(3*S*, 4*S*, 2'*R*)-Hgl-Val-*t*Bu **[(3*S*, 4*S*, 2'*R*)-19]**..22

N-Fmoc-(3*S*, 4*S*, 2'*S*)-Hgl-Val-*t*Bu **[(3*S*, 4*S*, 2'*S*)-19]**..23

(3*R*, 4*R*, 2'*R*)-*tert*-Butyl 2-[3-(Fmoc)amino-4-*p*-nitrobenzoyloxy-2-oxopyrrolidin-1-yl]-3-methylbutanoate **[(3*R*, 4*R*, 2'*R*)-20]** 23

(3*R*, 4*R*, 2'S)-*tert*-Butyl 2-[3-(Fmoc)amino-4-*p*-nitrobenzoyloxy-2-oxopyrrolidin-1-yl]-3-methylbutanoate **[(3*R*, 4*R*, 2'*S*)-20]** 24

(3*S*, 4*S*, 2'*R*)-*tert*-Butyl 2-[3-(Fmoc)amino-4-*p*-nitrobenzoyloxy-2-oxopyrrolidin-1-yl]-3-methylbutanoate **[(3*S*, 4*S*, 2'*R*)-20]** 25

(3*S*, 4*S*, 2'*S*)-*tert*-Butyl 2-[3-(Fmoc)amino-4-*p*-nitrobenzoyloxy-2-oxopyrrolidin-1-yl]-3-methylbutanoate **[(3*S*, 4*S*, 2'*S*)-20]** 25

References....26

LC-MS....27-39

NMR spectra....40-93

H-D-Arg-D-Tyr-(*R*)-Agl-(*S*)-Val-D-Glu-D-Leu-D-Ala-NH$_2$ [(3*R*)-Agl^3-(*S*)-Val]-1 [(3*R*, 2'*S*)-5]

Employing the representative procedure described for peptide (3*R*)-**5**, (3*R*,2'*S*)-**5** was synthesized (13 mg, 16% yield of >95% purity); LCMS [10-90% MeOH (0.1% FA) in water (0.1% FA) over 14 min; RT 7.6 min] and [10-90% MeCN (0.1% FA) in water (0.1% FA) over 14 min; RT 5.3 min]; HRMS (ESI$^+$) calcd m/z for $C_{38}H_{62}N_{11}O_{10}$ [M+H]$^+$, 832.4676 found 832.4687.

H-D-Arg-D-Tyr-(*S*)-Agl-D-Val-D-Glu-D-Leu-D-Ala-NH$_2$ [(3*S*)-Agl^3]-1 [(3*S*)-5]

Employing the representative procedure described for peptide (3*R*)-**5**, (3*S*)-**5** was synthesized (12 mg, 15% yield of >95% purity); LCMS [10-90% MeOH (0.1% FA) in water (0.1% FA) over 14 min; RT 8 min] and [10-90% MeCN (0.1% FA) in water (0.1% FA) over 14 min; RT 5.4 min]; HRMS (ESI$^+$) calcd m/z for $C_{38}H_{62}N_{11}O_{10}$ [M+H]$^+$, 832.4676 found 832.4680.

H-D-Arg-D-Tyr-(*S*)-Agl-(*S*)-Val-D-Glu-D-Leu-D-Ala-NH$_2$ [(3*S*)-Agl^3-(*S*)-Val]-1 [(3*S*, 2'*S*)-5]

Employing the representative procedure described for peptide (3*R*)-**5**, (3*S*,2'*S*)-**5** was synthesized (11 mg, 13% yield of >95% purity); LCMS [10-90% MeOH (0.1% FA) in water (0.1% FA) over 14 min; RT 7.9 min] and [10-90% MeCN (0.1% FA) in water (0.1% FA) over 14 min; RT 5.5 min]; HRMS (ESI$^+$) calcd m/z for $C_{38}H_{62}N_{11}O_{10}$ [M+H]$^+$, 832.4676 found 832.4692.

H-D-Arg-D-Tyr-(3*R*,4*R*)-Hgl-D-Val-D-Glu-D-Leu-D-Ala-NH$_2$ [(3*R*, 4*R*)-Hgl^3]-1 [(3*R*, 4*R*)-6]

Employing the representative procedure described for peptide (3*R*)-**5**, (3*R*,4*R*)-**6** was synthesized (7 mg, 8% yield of >95% purity); LCMS [10-90% MeOH (0.1% FA) in water (0.1% FA) over 14 min; RT 7.8 min] and [10-90% MeCN (0.1% FA) in water (0.1% FA) over 14 min; RT 5.4 min]; HRMS (ESI$^+$) calcd m/z for $C_{38}H_{63}N_{11}O_{11}$ [M+2H]$^{+2}$, 424.7349 found 424.73556 and calcd m/z for $C_{38}H_{62}N_{11}O_{11}$ [M+H]$^+$, 848.4625 found 848.4525.

H-D-Arg-D-Tyr-(3*R*,4*S*)-Hgl-D-Val-D-Glu-D-Leu-D-Ala-NH$_2$ [(3*R*, 4*S*)-Hgl^3]-1 [(3*R*, 4*S*)-6]

Employing the representative procedure described for peptide (3*R*)-**5**, (3*R*,4*S*)-**6** was synthesized (12 mg, 14% yield of >95% purity); LCMS [10-90% MeOH (0.1% FA) in water (0.1% FA) over 14 min; RT 7.5 min] and [10-90% MeCN (0.1% FA) in water (0.1% FA) over 14 min; RT 5.2 min]; HRMS (ESI$^+$) calcd m/z for $C_{38}H_{63}N_{11}O_{11}$ [M+2H]$^{+2}$, 424.7349 found 424.7342 and calcd m/z for $C_{38}H_{62}N_{11}O_{11}$ [M+H]$^+$, 848.4625 found 848.4573.

H-D-Arg-D-Tyr-(3*R*,4*R*)-Hgl-(*S*)-Val-D-Glu-D-Leu-D-Ala-NH$_2$ [(3*R*, 4*R*)-Hgl^3-(*S*)-Val]-1 [(3*R*, 4*R*, 2'*S*)-6]

Employing the representative procedure described for peptide (3*R*)-**5**, (3*R*,4*R*,2'*S*)-**6** was synthesized (11 mg, 13% yield of >95% purity); LCMS [10-90% MeOH (0.1% FA) in water (0.1% FA) over 14 min; RT 6.8 min] and [10-90% MeCN (0.1% FA) in water (0.1% FA) over 14 min; RT 4.9 min]; HRMS (ESI$^+$) calcd m/z for $C_{38}H_{63}N_{11}O_{11}$ [M+2H]$^{+2}$, 424.7349 found 424.7358 and calcd m/z for $C_{38}H_{62}N_{11}O_{11}$ [M+H]$^+$, 848.4625 found 848.4629.

H-D-Arg-D-Tyr-(3*R*,4*S*)-Hgl-(*S*)-Val-D-Glu-D-Leu-D-Ala-NH$_2$ [(3*R*, 4*S*)-Hgl^3-(*S*)-Val]-1 [(3*R*, 4*S*, 2'*S*)-6]

Employing the representative procedure described for peptide (3*R*)-**5**, (3*R*,4*S*,2'*S*)-**6** was synthesized (11 mg, 13% yield of >95% purity); LCMS [10-90% MeOH (0.1% FA) in water (0.1% FA) over14 min; RT 6.9 min] and [10-90% MeCN (0.1% FA) in water (0.1% FA) over 14 min; RT 4.9 min]; HRMS (ESI$^+$) calcd m/z for $C_{38}H_{63}N_{11}O_{11}$ [M+2H]$^{+2}$, 424.7349 found 424.7352 and calcd m/z for $C_{38}H_{62}N_{11}O_{11}$ [M+H]$^+$, 848.4625 found 848.4629.

H-D-Arg-D-Tyr-(3*S*,4*R*)-Hgl-D-Val-D-Glu-D-Leu-D-Ala-NH$_2$ [(3*S*, 4*R*)-Hgl^3]-1 [(3*S*, 4*R*)-6]

Employing the representative procedure described for peptide (3*R*)-**5**, (3*S*,4*R*)-**6** was synthesized (9 mg, 11% yield of >95% purity) as a white solid; LCMS [10-90% MeOH (0.1% FA) in water (0.1% FA) over 14 min; RT 7.8 min] and [10-90% MeCN (0.1% FA) in water (0.1% FA) over 14 min; RT 5.4 min]; HRMS (ESI$^+$) calcd m/z for $C_{38}H_{63}N_{11}O_{11}$ [M+2H]$^{+2}$, 424.7349 found 424.7340 and calcd m/z for $C_{38}H_{62}N_{11}O_{11}$ [M+H]$^+$, 848.4625 found 848.4625.

H-D-Arg-D-Tyr-(3*S*,4*S*)-Hgl-D-Val-D-Glu-D-Leu-D-Ala-NH$_2$ [(3*S*, 4*S*)-Hgl^3]-1 [(3*S*, 4*S*)-6]

Employing the representative procedure described for peptide (3*R*)-**5**, (3*S*,4*S*)-**6** was synthesized (8 mg, 9% yield of >95% purity); LCMS [10-90% MeOH (0.1% FA) in water (0.1% FA) over 14 min; RT 7.3 min] and [10-90% MeCN (0.1% FA) in water (0.1% FA) over 14 min; RT 5.2 min]; HRMS (ESI$^+$) calcd m/z for $C_{38}H_{63}N_{11}O_{11}$ [M+2H]$^{+2}$, 424.7349 found 424.7356 and calcd m/z for $C_{38}H_{62}N_{11}O_{11}$ [M+H]$^+$, 848.4625 found 848.4627.

H-D-Arg-D-Tyr-(3*S*,4*R*)-Hgl-(*S*)-Val-D-Glu-D-Leu-D-Ala-NH$_2$ [(3*S*, 4*R*)-Hgl^3-(*S*)-Val]-1 [(3*S*, 4*R*, 2'*S*)-6]

Employing the representative procedure described for peptide (3*R*)-**5**, (3*S*,4*R*,2'*S*)-**6** was synthesized (10 mg, 12% yield of >95% purity); LCMS [10-90% MeOH (0.1% FA) in water (0.1% FA) over 14 min; RT 7 min] and [10-90% MeCN (0.1% FA) in water (0.1% FA) over 14 min; RT 4.9 min]; HRMS (ESI$^+$) calcd m/z for $C_{38}H_{63}N_{11}O_{11}$ [M+2H]$^{+2}$, 424.7349 found 424.7353 and calcd m/z for $C_{38}H_{62}N_{11}O_{11}$ [M+H]$^+$, 848.4625 found 848.4618.

H-D-Arg-D-Tyr-(3*S*,4*S*)-Hgl-(*S*)-Val-D-Glu-D-Leu-D-Ala-NH$_2$ [(3*S*, 4*S*)-Hgl^3-(*S*)-Val]-1 [(3*S*, 4*S*, 2'*S*)-6]

Employing the representative procedure described for peptide (3*R*)-**5**, (3*S*,4*S*,2'*S*)-**6** was synthesized (10 mg, 12% yield of >95% purity); LCMS [10-90% MeOH (0.1% FA) in water (0.1% FA) over 14 min; RT 6.6 min] and [10-90% MeCN (0.1% FA) in water (0.1% FA) over 14 min; RT 5.2 min]; HRMS (ESI$^+$) calcd m/z for $C_{38}H_{63}N_{11}O_{11}$ (M+2H)$^{+2}$, 424.7349 found 424.7371 and calcd m/z for $C_{38}H_{62}N_{11}O_{11}$ (M+H)$^+$, 848.4625 found 848.4635.

(3*R*, 2'*S*)-2-[3-*N*-(Fmoc)amino-2-oxopyrrolidin-1-yl]-3-methylbutanoic acid [(*3R, 2'S*)-7]

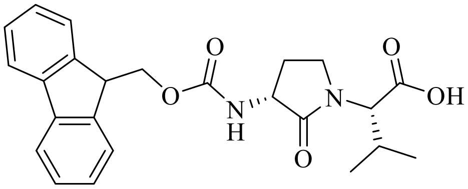

(3*R*, 2'*S*)-**7**

Employing the representative procedure described for the synthesis of (3*R*,2'*R*)-carbamate (3*R*,2'*R*)-**7**, (3*R*,2'*S*)-acid (3*R*,2'*S*)-**14** (51 mg, 0.209 mmol) was converted to (3*R* 2'*S*)-carbamate (3*R*,2'*S*)-**7** (75 mg, 0.178 mmol, 85%): R_f = 0.17 (10% MeOH:DCM); $[\alpha]_D^{25}$ –43.2° (*c* 0.5, MeOH); ^{1}H NMR (300 MHz, MeOD) δ 7.80 (d, *J* = 7.3, 2H), 7.68 (d, *J* = 7.4, 2H), 7.39 (t, *J* = 7.1, 2H), 7.31 (td, *J* = 7.4, 1.2, 2H), 5.57 (d, *J* = 5.6, 1H); 4.48-4.14 (m, 5H), 3.57-3.42 (m, 2H), 2.54-2.33 (m, 1H), 2.33-2.10 (m, 1H), 2.09-1.90 (m, 1H), 1.40-1.23 (m, 1H), 1.05 (d, *J* = 6.6, 3H), 0.95 (d, *J* = 6.6, 3H); ^{13}C NMR (75 MHz, MeOD) δ 173.9, 171.4, 157.1 143.9, 141.2, 127.3, 126.7, 124.8, 119.5, 66.6, 60.9, 52.4, 40.7, 27.0, 25.7, 24.8, 18.5, 18.0; HRMS (ESI$^+$) calcd *m/z* for $C_{24}H_{27}N_2O_5$ $[M+H]^+$, 423.1914 found 423.1906.

(3*S*, 2'*R*)-2-[3-*N*-(Fmoc)amino-2-oxopyrrolidin-1-yl]-3-methylbutanoic acid [(*3S, 2'R*)-7]

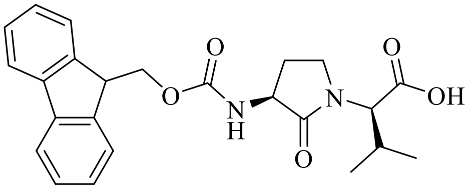

(3*S*, 2'*R*)-**7**

Employing the representative procedure described for the synthesis of (3*R*,2'*R*)-carbamate (3*R*,2'*R*)-**7**, (3*S*,2'*R*)-acid (3*S*,2'*R*)-**14** (1 eq., 86 mg, 0.352 mmol) was converted to (3*S*,2'*R*)-carbamate (3*S*,2'*R*)-**7** (100 mg, 0.237 mmol, 67%): $[\alpha]_D^{25}$ 2.4° (*c* 0.67, MeOH).

(3*S*, 2'*S*)-2-[3-*N*-(Fmoc)amino-2-oxopyrrolidin-1-yl]-3-methylbutanoic acid [(3*S*, 2'*S*)-7]

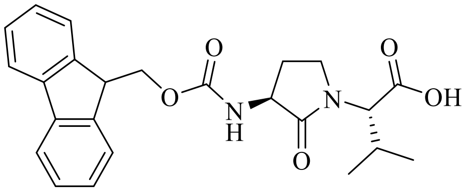

(3*S*, 2'*S*)-**7**

Employing the representative procedure described for the synthesis of (3*R*,2'*R*)-carbamate (3*R*,2'*R*)-**7**, (3*S*,2'*S*)-acid (3*S*,2'*S*)-**14** (1 eq., 67 mg, 0.274 mmol) was converted to (3*S*,2'*S*)-carbamate (3*S*,2'*S*)-**7** (81 mg, 0.192 mmol, 70%): $[\alpha]_D^{25}$ –89.9° (*c* 0.76, MeOH).

N-Boc-(R)-Methionine [(R)-8]

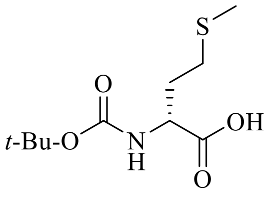

(*R*)-**8**

D-Met-OH (1 eq., 2 g, 13.4 mmol), was dissolved in a 1:1 mixture of dioxane (10 mL) and H_2O (10 mL). The addition of NaOH (2 eq., 2 M, 13.4 mL, 26.8 mmol) to this reaction mixture dissolved the reactant and afforded a miscible solution. The reaction mixture was then cooled to 0 °C and stirred for 15 min. Finally, $(Boc)_2O$ (1.1 eq., 3.22 g, 3.15 mL, 14.7 mmol) was added, and the reaction mixture was stirred at 0 °C for 10 min. The ice bath was then removed, and the temperature of the reaction mixture warmed to rt with stirring for 18 h. On completion of the reaction, the reaction mixture was concentrated under reduced pressure. The aqueous layer was acidified with citric acid 0.5 N (pH 2 to 3) and extracted with EtOAc. The organic extractions were cobined, washed with brine, dried over Na_2SO_4, and concentrated under reduced pressure to provide *N*-Boc-D-methionine [(*R*)-**8**, 3.17 g, 12.7 mmol, 95%] as white solid[1]: mp 47-50 °C (Lit.[2] 50-51 °C); R_f = 0.6 (10% EtOH in $CHCl_3$); $[\alpha]_D^{20}$ 23° (*c* 1, MeOH) [Lit.[2] $[\alpha]_D^{20}$ 22°, (*c* 1, MeOH)].

N-Boc-(S)-Methionine [(S)-8]

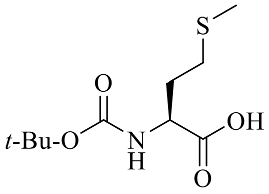

(*S*)-**8**

Employing the representative procedure described for the synthesis of *N*-Boc-D-methionine (*R*)-**8**, NaOH (2 eq., 2 M, 13.4 mL, 26.8 mmol) and $(Boc)_2O$ (1.1 eq., 3.22 g, 3.15 mL, 14.7 mmol) were

added to H-L-Met-OH (1 eq., 2 g, 13.4 mmol) respectively to yield *N*-Boc-L-methionine (*S*)-**8** (3.28 g, 13.1 mmol, 98%): $[\alpha]_D^{20}$ –25° (*c* 1, MeOH) {Lit.[2] $[\alpha]_D^{22}$ –22.8°, (*c* 1, MeOH)}.

(*R*)-*N*-(Boc)-Methioninyl-(*S*)-valine *tert*-butyl ester [(*R*, *S*)-10]

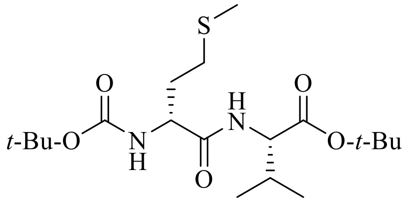

(*R*, *S*)-**10**

Employing the representative procedure described for the synthesis of (*R*,*R*)-dipeptide (*R*,*R*)-**10**, *N*-Boc-(*R*)-methionine (1 eq., 1 g, 4 mmol) and (*S*)-valine *tert*-butyl ester hydrochloride (*S*)-**9**•HCl (1 eq., 840 mg, 4 mmol) were coupled to give (*R*,*S*)-dipeptide (*R*,*S*)-**10** as light yellow oil (1.13 g, 2.8 mmol, 70%): R_f = 0.3 (20% EtOAc in hexane); $[\alpha]_D^{25}$ 13.7° (*c* 1.9, $CHCl_3$); ^{1}H NMR (300 MHz, $CDCl_3$) δ 6.67 (d, *J* = 8.3, 1H), 5.23 (d, *J* = 7.2, 1H), 4.39 (dd, *J* = 8.7, 4.4, 1H), 4.36-4.22 (m, 1H), 2.63-2.47 (m, 2H), 2.22-2.04 (m, 4H), 1.90 (tt, *J* = 7.6, 3.9, 1H), 1.44 (s, 9H), 1.43 (s, 9H), 0.92 (d, *J* = 6.9, 3H), 0.88 (d, *J* = 6.9, 3H); ^{13}C NMR (75 MHz, $CDCl_3$) δ 171.4, 170.8, 155.6, 82.1, 80.2, 57.5, 53.9, 31.8, 31.5, 30.3, 28.4, 28.1, 19.0, 17.6, 15.4; HRMS (ESI$^+$) calcd *m/z* for $C_{19}H_{36}N_2O_5S$ $[M+H]^+$, 405.2418 found 405.2424.

(*S*)-*N*-(Boc)-Methioninyl-(*R*)-valine *tert*-butyl ester [(*S*, *R*)-10]

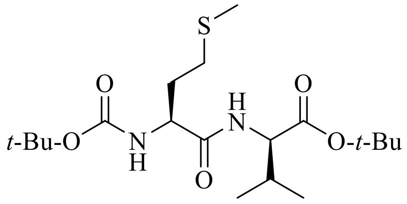

(*S*, *R*)-**10**

Employing the representative procedure described for the synthesis of (*R*,*R*)-dipeptide (*R*,*R*)-**10**, *N*-Boc-(*S*)-methionine (1 eq., 1 g, 4 mmol) and (*R*)-valine *tert*-butyl ester hydrochloride (*R*)-**9** HCl (1 eq., 840 mg, 4 mmol) were coupled to give (*S*,*R*)-dipeptide (*S*,*R*)-**10** as light yellow oil (1.22 g, 3.01 mmol, 75%): $[\alpha]_D^{25}$ –23.3° (*c* 3.7, $CHCl_3$).

(*S*)-*N*-(Boc)-Methioninyl-(*S*)-valine *tert*-butyl ester [(*S*, *S*)-10]

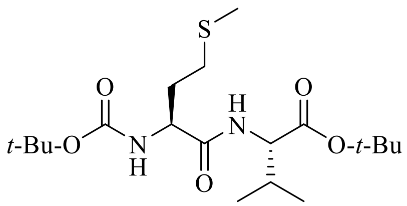

(*S*, *S*)-**10**

Employing the representative procedure described for the synthesis of (*R*,*R*)-dipeptide (*R*,*R*)-**10**, *N*-Boc-(*S*)-methionine (*S*)-**8** (1 eq., 1 g, 4 mmol) and (*S*)-valine *tert*-butyl ester hydrochloride (*S*)-**9** HCl (1 eq., 840 mg, 4 mmol) were coupled to give (*S*,*S*)-dipeptide (*S*,*S*)-**10** (1.33 g, 3.28 mmol, 82%): $[\alpha]_D^{25}$ 13.0° (*c* 1, $CHCl_3$).

N-Boc-(*R*)-Methioninyl-(*S*)-valine *tert*-butyl ester methylsulfonium iodide [(*R*, *S*)-11]

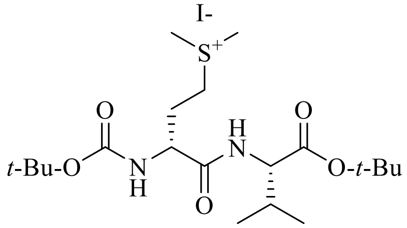

(*R*, *S*)-**11**

Employing the representative procedure described for the synthesis of (*R*,*R*)-methylsulfonium iodide (*R*,*R*)-**11**, (*R*,*S*)-dipeptide (*R*,*S*)-**10** (1 g, 2.47 mmol) was converted to (*R*,*S*)-methylsulfonium iodide (*R*,*S*)-**11** as light yellow foam (1.065 g, 1.95 mmol, 79%): R_f = 0.18 (5% MeOH in DCM); $[\alpha]_D^{25}$ 14° (*c* 1, MeOH); ^{1}H NMR (300 MHz, $CDCl_3$) δ 7.52-7.36 (br s, 1H), 6.00 (d, *J* = 7.3, 1H), 4.54-4.43 (br s, 1H), 4.27 (dd, *J* = 8.3, 5.1, 1H), 3.95-3.82 (m, 1H), 3.78-3.60 (br s, 1H), 3.31 (s, 3H), 3.23 (s, 3H), 2.60-2.52 (m, 1H), 2.31-2.16 (m, 2H), 1.44 (dd, *J* = 5.9, 2.4, 18H), 0.95 (dd, *J* = 6.8, 4.6, 6H); HRMS (ESI^+) calcd *m/z* for $C_{20}H_{39}N_2O_5S$ $[M]^+$, 419.2574 found 419.2573.

N-Boc-(*S*)-Methioninyl-(*R*)-valine *tert*-butyl ester methylsulfonium iodide [(*S*, *R*)-11]

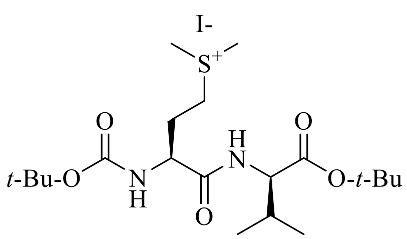

(*S*, *R*)-**11**

Employing the representative procedure described for the synthesis of (*R*,*R*)-methylsulfonium iodide (*R*,*R*)-**11**, (*S*,*R*)-dipeptide (*S*,*R*)-**10** (1 g, 2.47 mmol) was converted to (*S*,*R*)-methylsulfonium iodide (*S*,*R*)-**11** as light yellow foam (1.187 g, 2.17 mmol, 88%): $[\alpha]_D^{25}$ –31° (*c* 1, MeOH).

_N_-Boc-(_S_)-Methioninyl-(_S_)-valine _tert_-butyl ester methylsulfonium iodide [(_S, S_)-11]

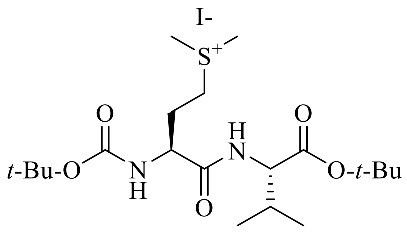

(*S*, *S*)-**11**

Employing the representative procedure described for the synthesis of (*R*,*R*)-methylsulfonium iodide (*R*,*R*)-**11**, (*S*,*S*)-dipeptide (*S*,*S*)-**10** (1 g, 2.47 mmol) was converted to (*S*,*S*)-methylsulfonium iodide (*S*,*S*)-**11** as yellow gummy foam (670 mg, 1.23 mmol, 99%): $[\alpha]_D^{25}$ –28° (*c* 1, MeOH).

_N_-Fmoc-(3_R_, 4_R_, 2'_R_)-Hgl-Val-OH [(3_R_, 4_R_, 2'_R_)-12]

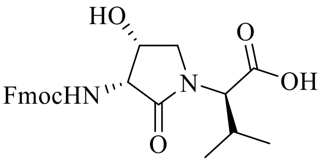

(3*R*, 4*R*, 2'*R*)-**12**

Employing the previously described procedure,[3] (3*R*,4*R*,2'*R*)-ester (3*R*,4*R*,2'*R*)-**19** (133 mg, 0.269 mmol) was converted into (3*R*,4*R*,2'*R*)-acid (3*R*,4*R*,2'*R*)-**12** (113 mg, 0.258 mmol, 96%): R_f = 0.08 (10% MeOH in DCM); $[\alpha]_D^{20}$ 14.8 (*c* 1.3, MeOH); HRMS (ESI$^+$) calcd m/z for $C_{24}H_{27}N_2O_6$ $[M+H]^+$, 439.1864 found 439.1868.

_N_-Fmoc-(3_R_, 4_S_, 2'_R_)-Hgl-Val-OH [(3_R_, 4_S_, 2'_R_)-12]

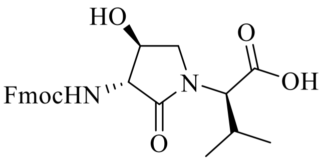

(3*R*, 4*S*, 2'*R*)-**12**

Employing the previously described procedure,[3] (3*R*,4*S*,2'*R*)-ester (3*R*,4*S*,2'*R*)-**19** (450 mg, 0.91 mmol) was converted into (3*R*,4*S*,2'*R*)-acid (3*R*,4*S*,2'*R*)-**12** (379 mg, 0.86 mmol, 95 %): R_f = 0.16 (5% THF in DCM); HRMS (ESI$^+$) calcd m/z for $C_{24}H_{27}N_2O_6$ [M+H]$^+$, 439.1864 found 439.1861.

_N_-Fmoc-(3_R_, 4_R_, 2'_S_)-Hgl-Val-OH [(3_R_, 4_R_, 2'_S_)-12]

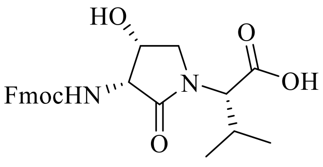

(3*R*, 4*R*, 2'*S*)-**12**

Employing the previously described procedure,[3] (3*R*,4*R*,2'*S*)-ester (3*R*,4*R*,2'*S*)-**19** (220 mg, 0.445 mmol) was converted into (3*R*,4*R*,2'*S*)-acid (3*R*,4*R*,2'*S*)-**12** (181 mg, 0.414 mmol, 93 %): R_f = 0.07 (10% MeOH in DCM); ^{1}H NMR (300 MHz, MeOD) δ 7.82 (d, *J* = 7.3, 2H), 7.73 (dd, *J* = 7.1, 3.4, 2H), 7.41 (t, *J* = 7.3, 2H), 7.34 (td, *J* = 7.3, 0.9, 2H), 4.52 (d, *J* = 4.9, 1H), 4.44 (dd, *J* = 7.1, 3.2, 2H), 4.39 (d, *J* = 5.2, 1H), 4.35 (d, *J* = 3.3, 1H), 4.29 (dd, *J* = 14.0, 7.0, 1H), 3.75 (dd, *J* = 11.4, 3.6, 1H), 3.41 (d, *J* = 11.4, 1H), 2.31-2.14 (m, 1H), 1.07 (d, *J* = 6.6, 3H), 0.99 (d, *J* = 6.7, 3H); ^{13}C NMR (75 MHz, MeOD) δ 172.5, 171.4, 157.6, 143.7, 141.2, 127.4, 126.8, 124.9, 119.5, 69.1, 67.1, 65.7, 60.5, 56.7, 50.3, 27.0, 20.9, 18.4, 17.7; HRMS (ESI$^+$) calcd m/z for $C_{24}H_{27}N_2O_6$ [M+H]$^+$, 439.1864 found 439.1871.

_N_-Fmoc-(3_R_, 4_S_, 2'_S_)-Hgl-Val-OH [(3_R_, 4_S_, 2'_S_)-12]

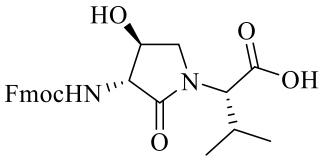

(3*R*, 4*S*, 2'*S*)-**12**

Employing the previously described procedure,[3] (3*R*,4*S*,2'*S*)-ester (3*R*,4*S*,2'*S*)-**19** (220 mg, 0.445 mmol) was converted into (3*R*,4*S*,2'*S*)-acid (3*R*,4*S*,2'*S*)-**12** (150 mg, 0.342 mmol, 77%): R_f = 0.1 (5% THF in DCM); HRMS (ESI$^+$) calcd m/z for $C_{24}H_{27}N_2O_6$ [M+H]$^+$, 439.1864 found 439.1853.

_N_-Fmoc-(3_S_, 4_R_, 2'_R_)-Hgl-Val-OH [(3_S_, 4_R_, 2'_R_)-12]

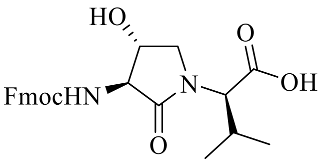

(3*S*, 4*R*, 2'*R*)-**12**

Employing the previously described procedure,[3] (3*S*,4*R*,2’*R*)-ester (3*S*,4*R*,2’*R*)-**19** (120 mg, 0.243 mmol) was converted into (3*S*,4*R*,2’*R*)-acid (3*S*,4*R*,2’*R*)-**12** (85.1 mg, 0.194 mmol, 80%).

***N*-Fmoc-(3*S*, 4*S*, 2’*R*)-Hgl-Val-OH [(3*S*, 4*S*, 2’*R*)-12]**

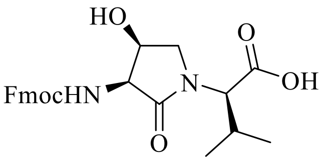

(3*S*, 4*S*, 2'*R*)-**12**

Employing the previously described procedure,[3] (3*S*,4*S*,2’*R*)-ester (3*S*,4*S*,2’*R*)-**19** (145 mg, 0.293 mmol) was converted into (3*S*,4*S*,2’*R*)-acid (3*S*,4*S*,2’*R*)-**12** (106 mg, 0.243 mmol, 83 %).

***N*-Fmoc-(3*S*, 4*R*, 2’*S*)-Hgl-Val-OH [(3*S*, 4*R*, 2’*S*)-12]**

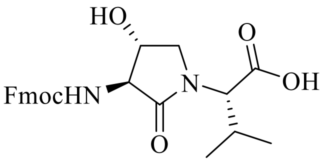

(3*S*, 4*R*, 2'*S*)-**12**

Employing the previously described procedure,[3] (3*S*,4*R*,2’*S*)-ester (3*S*,4*R*,2’*S*)-**19** (250 mg, 0.505 mmolwas converted to (3*S*,4*R*,2’*S*)-acid (3*S*,4*R*,2’*S*)-**12** (160 mg, 0.365 mmol, 72%).

***N*-Fmoc-(3*S*, 4*S*, 2’*S*)-Hgl-Val-OH [(3*S*, 4*S*, 2’*S*)-12]**

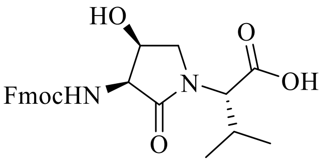

(3*S*, 4*S*, 2'*S*)-**12**

Employing the previously described procedure,[3] (3*S*,4*S*,2’*S*)-ester (3*S*,4*S*,2’*S*)-**19** (80 mg, 0.162 mmol) was converted into (3*S*,4*S*,2’*S*)-acid 3*S*,4*S*,2’*S*)-**12** (62.4 mg, 0.142 mmol, 88%).

(3*R*, 2'*S*)-*tert*-Butyl 2-[3-(Boc)amino-2-oxopyrrolidin-1-yl]-3-methylbutanoate [(3*R*, 2'*S*)-13]

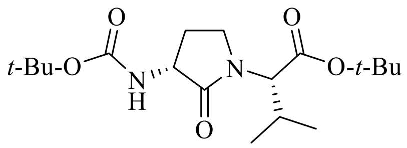

(3*R*, 2'*S*)-**13**

Employing the representative procedure described for the synthesis of (3*R*,2'*R*)-lactam (3*R*,2'*R*)-**13**, (*R*,*S*)-methylsulfonium iodide (*R*,*S*)-**11** (274 mg, 0.501 mmol) was converted to (3*R*,2'*S*)-lactam (3*R*,2'*S*)-**13** (109 mg, 0.306 mmol, 61%): R_f = 0.30 (30% EtOAc in Hexane); $[\alpha]_D^{25}$ –83.7° (*c* 1, $CHCl_3$); ^1H NMR (300 MHz, $CDCl_3$) δ 5.12 (s, 1H), 4.32 (d, *J* = 10.1, 1H), 4.26-4.09 (m, 1H), 3.50-3.29 (m, 2H), 2.77-2.58 (m, 1H), 2.17 (ddt, *J* = 13.4, 10.3, 6.7, 1H), 1.86-1.67 (m, 1H), 1.44 (s, 18H), 1.00 (d, *J* = 6.6, 3H), 0.86 (d, *J* = 6.7, 3H); ^{13}C NMR (75 MHz, $CDCl_3$) δ 173.1, 169.4, 156.0, 82.2, 80.0, 61.4, 52.9, 41.4, 29.2, 28.5, 28.2, 27.6, 19.5, 19.2; HRMS (ESI^+) calcd *m/z* for $C_{18}H_{33}N_2O_5$ $[M+H]^+$, 357.2384 found 357.2394.

(3*S*, 2'*R*)-*tert*-Butyl 2-[3-(Boc)amino-2-oxopyrrolidin-1-yl]-3-methylbutanoate [(3*S*, 2'*R*)-13]

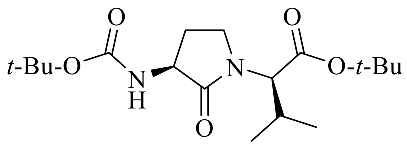

(3*S*, 2'*R*)-**13**

Employing the representative procedure described for the synthesis of (3*R*,2'*R*)-lactam (3*R*,2'*R*)-**13**, (*S*,*R*)-methylsulfonium iodide (*S*,*R*)-**11** (1 eq., 450 mg, 0.8 mmol) was converted (3*S*,2'*R*)-lactam (3*S*,2'*R*)-**13** (181 mg, 0.511 mmol, 62%): $[\alpha]_D^{25}$ 53.1° (*c* 1.2, $CHCl_3$).

(3*S*, 2'*S*)-*tert*-Butyl 2-[3-(Boc)amino-2-oxopyrrolidin-1-yl]-3-methylbutanoate [(3*S*, 2'*S*)-13]

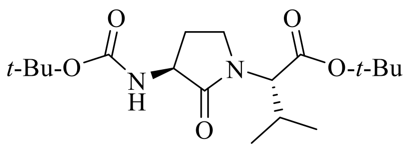

(3*S*, 2'*S*)-**13**

Employing the representative procedure described for the synthesis of (3*R*,2'*R*)-lactam (3*R*,2'*R*)-**13**, (*S*,*S*)-methylsulfonium iodide (*S*,*S*)-**11** (1 eq., 158 mg, 0.3 mmol) was converted to (3*S*,2'*S*)-lactam (3*S*,2'*S*)-**13** (65 mg, 0.182 mmol, 63%): $[\alpha]_D^{25}$ –39.7° (*c* 1, $CHCl_3$).

(3*R*, 2'*S*)-2-[3-amino-2-oxopyrrolidin-1-yl]-3-methylbutanoate trifluoroacetate [(3*R*, 2'*S*)-14]

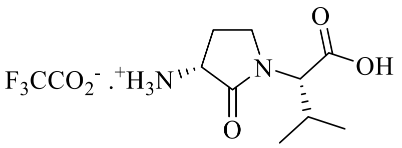

(3*R*, 2'*S*)-**14**

Employing the representative procedure described for the synthesis of (3*R*,2'*R*)-trifluoroacetate (3*R*,2'*R*)-**14**, (3*R* 2'*S*)-lactam (3*R*,2'*S*)-**13** (74 mg, 0.208 mmol) was converted to a white precipitate, (3*R*,2'*S*)-trifluoroacetate (3*R*,2'*S*)-**14** (62 mg, 0.197 mmol, 95%): R_f = 0.12 (1:9:90 Et_3N:MeOH:DCM); $[\alpha]_D^{25}$ –69.5° (*c* 0.42, MeOH); ^{1}H NMR (300 MHz, MeOD) δ 4.31 (d, *J* = 10.1, 1H), 4.10 (dd, *J* = 11.1, 8.5, 1H), 3.65-3.45 (m, 2H), 2.67-2.52 (m, 1H), 2.35-2.17 (m, 1H), 2.11-1.91 (m, 1H), 1.07 (d, *J* = 6.6, 3H), 0.95 (d, *J* = 6.7, 3H); ^{13}C NMR (75 MHz, MeOD) δ 171.0, 170.0, 61.0, 50.6, 41.2, 27.1, 24.5, 18.4 18.0; HRMS (ESI$^+$) calcd *m/z* for $C_9H_{17}N_2O_3$ [M+H]$^+$, 201.1234 found 201.1225.

(3*S*, 2'*R*)-2-[3-amino-2-oxopyrrolidin-1-yl]-3-methylbutanoate trifluoroacetate [(3*S*, 2'*R*)-14]

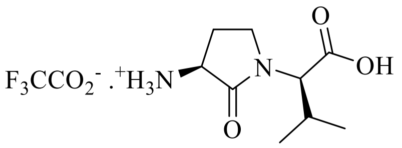

(3*S*, 2'*R*)-**14**

Employing the representative procedure described for the synthesis of (3*R*,2'*R*)-trifluoroacetate (3*R*,2'*R*)-**14**, (3*S*,2'*R*)-lactam (3*S*,2'*R*)-**13** (115 mg, 0.323 mmol) was converted to a white precipitate, (3*S*,2'*R*)-trifluoroacetate (3*S*,2'*R*)-**14** (97 mg, 0.309 mmol, 96%): $[\alpha]_D^{25}$ 8.0° (*c* 0.8, MeOH).

(3*S*, 2'*S*)-2-[3-amino-2-oxopyrrolidin-1-yl]-3-methylbutanoate trifluoroacetate [(*3S, 2'S*)-14]

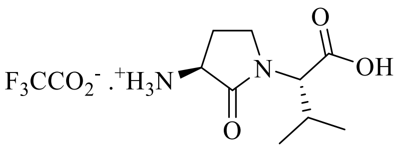

(3*S*, 2'*S*)-**14**

Employing the representative procedure described for the synthesis of (3*R*,2'*R*)-trifluoroacetate (3*R*,2'*R*)-**14**, (3*S*,2'*S*)-lactam (3*S*,2'*S*)-**13** (1 eq., 100 mg, 0.281 mmol) was converted to (3*S*,2'*S*)-trifluoroacetate (3*S*,2'*S*)-**14** (80 mg, 0.255 mmol, 91%): $[\alpha]_D^{25}$ –69.2° (*c* 1, MeOH).

_N_-Fmoc-(_R_)-methionine methyl ester [(_R_)-15]

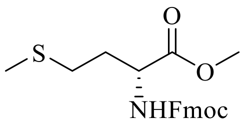

(*R*)-**15**

Employing the previously described procedure,[4] (*R*)-Met-OMe•HCl (1 eq., 7.5 g, 37.6 mmol, prepared from (*R*)-methionine as previously described)[5] was converted to *N*-Fmoc-(*R*)-Met-OMe (*R*)-**15** as light yellow solid (14.5 g, 37.6 mmol, 100 %) that was used without further purification: R_f = 0.32 (20% EtOAc in hexane); mp 93 °C (Lit.[6] 88-89 °C); $[\alpha]_D^{20}$ –12° (*c* 1, $CHCl_3$); ^{1}H NMR (300 MHz, $CDCl_3$) δ 7.77 (d, *J* = 7.7, 2H), 7.60 (d, *J* = 6.6, 2H), 7.41 (t, *J* = 7.5, 2H), 7.32 (tt, *J* = 7.4, 1.1, 2H), 5.46 (d, *J* = 8.2, 1H), 4.51 (dd, *J* = 12.9, 7.7, 1H), 4.42 (d, *J* = 7.0, 2H), 4.23 (t, *J* = 6.8, 1H), 3.76 (s, 3H), 2.53 (t, *J* = 7.3, 2H), 2.26-1.90 (m, 5H); ^{13}C NMR (75 MHz, $CDCl_3$) δ 172.5, 155.9, 143.8, 141.3, 127.7, 127.1, 125.0, 120.0, 67.0, 53.1, 52.6, 47.2, 31.9, 29.9, 15.5.

_N_-Fmoc-(_S_)-methionine methyl ester [(_S_)-15]

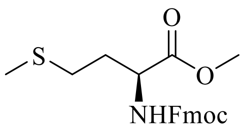

(*S*)-**15**

Employing the previously described procedure,[4] (*S*)-Met-OMe•HCl (1 eq., 7.5 g, 37.6 mmol) was converted to *N*-Fmoc-(*S*)-Met-OMe (*S*)-**15** as light yellow solid (14.5 g, 37.6 mmol, 100 %) that was used without further purification: $[\alpha]_D^{20}$ 18° (*c* 1, $CHCl_3$).

_N_-Fmoc-(_R_)-Methionine sulfoxide methyl ester [(_R_)-16]

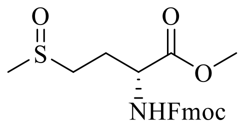

(*R*)-**16**

Employing the previously described procedure,[4] (*R*)-Fmoc-Met-OMe (1 eq., 12 g, 31.1 mmol) was converted to sulfoxide (*R*)-**16** as yellow sticky oil (10 g, 24.9 mmol, 80 %): R_f = 0.28 (3% MeOH in DCM); $[\alpha]_D^{20}$ –25° (*c* 1, $CHCl_3$); ^{1}H NMR (300 MHz, $CDCl_3$) δ 7.77 (d, *J* = 7.5, 2H), 7.65-7.54 (br, 2H), 7.41 (t, *J* = 7.5, 2H), 7.32 (tt, *J* = 7.4, 1.3, 2H), 5.77 (d, *J* = 8.3, 0.5H), 5.68 (d, *J* = 7.5, 0.5H), 4.58-4.47 (m, 1H), 4.42 (d, *J* = 7.4, 2H), 4.22 (t, *J* = 6.8, 1H), 3.78 (s, 3H), 2.88-2.62 (m, 2H), 2.57 (s, 3H), 2.51-2.31 (m, 1H), 2.30-2.08 (m, 1H); ^{13}C NMR (75 MHz, $CDCl_3$) δ 171.7, 156.2, 143.6, 141. 3, 127.8, 127.1, 125.1, 120.0, 67.1, 60.4, 52.8, 50.1, 47.1, 38.6, 25.8.

***N*-Fmoc-(S)-Methionine sulfoxide methyl ester [(*S*)-16]**

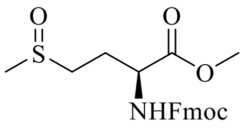

(*S*)-**16**

Employing the previously described procedure,[4] *N*-Fmoc-(*S*)-Met-OMe (*S*)-**15** (1 eq., 14.5 g, 37.6 mmol) was converted to a 1:1 mixture of diasteromers of sulfoxide (*S*)-**16** as white foam (11.6 g, 28.9 mmol, 77 %): $[\alpha]_D^{20}$ 44° (*c* 0.8, $CHCl_3$).

***N*-Fmoc-(2*R*)-Vinylglycine- methyl ester [(*R*)-17]**

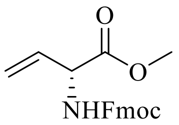

(*R*)-**17**

Employing the previously described procedure,[7] using a longer reaction time, 2 h, *N*-Fmoc-(*R*)-Met-sulfoxide-OMe (*R*)-**16** (1 eq., 35 mg, 87.2 mol) was transformed into vinylglycine (*R*)-**17** (24 mg, 0.074 mmol, 84 %); R_f = 0.34 (20% EtOAc in hexane); $[\alpha]_D^{25}$ –28 (c 0.7, $CHCl_3$); ^{1}H NMR (300 MHz, $CDCl_3$) δ 7.77 (d, *J* = 7.5, 2H), 7.61 (d, *J* = 6.9, 2H), 7.41 (t, *J* = 7.4, 2H), 7.32 (t, *J* = 7.4, 2H), 6.00-5.82 (m, 1H), 5.51 (d, *J* = 7.7, 1H), 5.32 (dd, *J* = 18.9, 13.9, 2H), 4.96 (s, 1H), 4.43 (d, *J* = 7.0, 2H),

4.24 (t, J = 6.9, 1H), 3.79 (s, 3H); ^{13}C NMR (75 MHz, $CDCl_3$) δ 170.9, 155.6, 143.8, 141.3, 132.3, 127.7, 127.1, 125.1, 120.0, 117.8, 67.1, 56.1, 52.8, 47.2.

***N*-Fmoc-(2*S*)-Vinylglycine- methyl ester [(*S*)-17]**

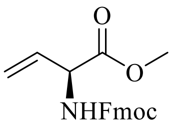

(*S*)-**17**

Employing the previously described procedure,[7] using a longer reaction time, 2 h, *N*-Fmoc-(*S*)-Met-sulfoxide-OMe (*S*)-**16** (1 eq., 35 mg, 87.2 mol) was converted to vinylglycine (*S*)-**17** (25 mg, 0.074 mmol, 85 %): $[\alpha]_D^{20}$ 3° (c 0.5, $CHCl_3$) {Lit.[8] $[\alpha]_D^{20}$ 2.6° (c 5.5, $CHCl_3$)}.

***N*-Fmoc-(2*S*)-Vinylglycine methyl ester [(*S*)-17]**

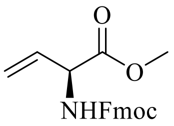

(*S*)-**17**

Employing the previously described procedure,[9] *N*-Fmoc-(*S*)-Met-sulfoxide-OMe (*S*)-**16** (1 eq., 5.36 g, 13.4 mmol) was converted to vinylglycine (*S*)-**17** (3.15 g, 9.35 mmol, 70 %): $[\alpha]_D^{20}$ 3° (c 0.5, $CHCl_3$) {Lit.[8] $[\alpha]_D^{20}$ 2.6° (c 5.5, $CHCl_3$)}.

(2*R*, 2'*R*)- and (2*R*, 2'*S*)-Methyl 2-(oxiranyl)-*N*-(Fmoc)glycinate [(2*R*, 2'*R*)-18 and (2*R*, 2'*S*)-18]

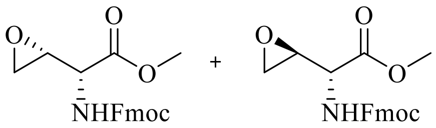

(2*R*, 2'*R*)-**18** (2*R*, 2'*S*)-**18**

A solution of vinylglycine (*R*)-**17** (1 eq., 100 mg, 0.296 mmol) in toluene (4 mL) was treated with *m*-CPBA (77%, 5 eq., 332 mg, 1.48 mmol), heated to 80 °C using microwave irradiation for 2 h, cooled using an ice bath and treated with 30 mL of saturated Na_2SO_3. The phases were separated. The organic layer was washed with Na_2SO_3, saturated $NaHCO_3$ and brine, and the washing sequences were

repeated. After drying the organic layer over Na_2SO_4, the volatiles were evaporated to a residue that was purified by column chromatography eluting with a step gradient of 5-20% EtOAc in toluene, which after evaporation furnished three fractions [(20 mg, 0.06 mmol, 19 %), (52.4 mg, 0.15 mmol, 50 %), and (3 mg, 0.009 mmol, 3 %)] with 9:1, 2.7:1, and 1:9 ratios of (2*R*,2'*R*) to (2*R*,2'*S*) assessed by NMR analysis comparing the relative intensities of the peaks at 3.48 and 3.24 ppm respectively, providing a combined (75.4 mg, 0.213 mmol, 71 %) of oxiranylglycine (2*R*,2'*R*)-**18** and (2*R*,2'*S*)-**18** with ratio of 3:1.

Oxiranylglycine (2*R*,2'*R*)-**18**; R_f = 0.23 (15% EtOAc in toluene); $[\alpha]_D^{20}$ –5.4° (*c* 0.8, $CHCl_3$); ^1H NMR (500 MHz, $CDCl_3$) δ 7.77 (d, *J* = 7.6, 2H), 7.60 (t, *J* = 6.6, 2H), 7.41 (t, *J* = 7.5, 2H), 7.32 (tdd, *J* = 7.4, 3.5, 1.0, 2H), 5.32 (d, *J* = 8.8, 1H), 4.73 (dd, *J* = 8.9, 1.6, 1H), 4.43 (d, *J* = 7.0, 2H), 4.22 (t, *J* = 6.9, 1H), 3.83 (s, 3H), 3.48 (s, 1H), 2.79 (t, *J* = 4.2, 1H), 2.62 (dd, *J* = 4.1, 2.5, 1H); ^{13}C NMR (126 MHz, $CDCl_3$) δ 170.1, 156.2, 143.7, 143.5, 141.3(2C), 127.7 (2C), 127.1 (2C), 125.0 (2C), 120.0 (2C), 67.2, 53.0 (2C), 51.1, 47.1, 43.8; HRMS (ESI^+) calcd *m/z* for $C_{20}H_{20}NO_5$ $[M+H]^+$, 354.1336 found 354.1330.

Oxiranylglycine (2*R*,2'*S*)-**18**; R_f = 0.21 (15% EtOAc in toluene); $[\alpha]_D^{20}$ –31.2° (*c* 0.8, $CHCl_3$); ^1H NMR (500 MHz, $CDCl_3$) δ 7.77 (d, *J* = 7.6, 2H), 7.60 (d, *J* = 7.5, 2H), 7.41 (td, *J* = 7.7, 2.8, 2H), 7.32 (td, *J* = 7.4, 1.1, 2H), 5.64 (d, *J* = 7.5, 1H), 4.53 (dd, *J* = 7.6, 5.0, 1H), 4.42 (d, *J* = 6.9, 2H), 4.23 (t, *J* = 6.8, 1H), 3.82 (s, 3H), 3.25 (s, 1H), 2.82 (d, *J* = 2.5, 2H); ^{13}C NMR (126 MHz, $CDCl_3$) δ 169.7, 155.8, 143.7, 143.6, 141.2 (2C), 128.2 (2C), 127.7, 127.0, 125.0 (2C), 120.0 (2C), 67.3, 54.7, 52.9, 51.4, 47.0, 45.0; HRMS (ESI^+) calcd *m/z* for $C_{20}H_{20}NO_5$ $[M+H]^+$, 354.1336 found 354.1330.

(2*S*, 2'*S*)- and (2*S*, 2'*R*)-Methyl 2-(oxiranyl)-*N*-(Fmoc)glycinate [(2*S*, 2'*S*)-18 and (2*S*, 2'*R*)-18]

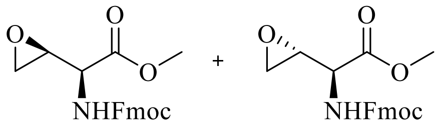

(2*S*, 2'*S*)-**18** (2*S*, 2'*R*)-**18**

Employing the representative procedure described for oxiranylglycines (2*R*,2'*R*)- and (2*R*,2'*S*)-**18**, vinylglycine (*S*)-**17** (1 eq., 100 mg, 0.296 mmol) was converted to oxiranylglycine (2*S*,2'*S*)-**18**; $[\alpha]_D^{20}$ –18.3° (*c* 1.1, $CHCl_3$) and (2*S*,2'*R*)-**18**; $[\alpha]_D^{20}$ –10.9° (*c* 1.2, $CHCl_3$) (75.4 mg, 0.213 mmol, 73 %) with ratio of 3:1.

***N*-Fmoc-(3*R*, 4*S*, 2'*R*)-Hgl-Val-*t*Bu [(3*R*, 4*S*, 2'*R*)-19]**

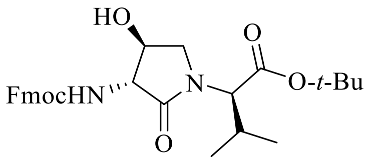

(3*R*, 4*S*, 2'*R*)-**19**

Employing the previously described procedure,[3] (2*R*,2'*R*)-oxiranylglycine (2*R*,2'*R*)-**18** (1 eq., 500 mg, 1.41 mmol) was converted to (3*R*,4*S*,2'*R*)-lactam (3*R*,4*S*,2'*R*)-**19** (545 mg, 1.1 mmol, 78%): R_f = 0.45 (3% THF in DCM); $[\alpha]_D^{20}$ 7.7° (*c* 2, $CHCl_3$); ^{1}H NMR (500 MHz, $CDCl_3$) δ 7.77 (dd, *J* = 7.6, 0.5, 2H), 7.59 (d, *J* = 7.5, 2H), 7.41 (t, *J* = 7.5, 2H), 7.33 (td, *J* = 7.5, 1.1, 2H), 5.76 (s, 1H), 5.01 (s, 1H), 4.44 (d, *J* = 1.8, 1H), 4.43 (dd, *J* = 5.0, 3.9, 2H), 4.34 (q, *J* = 8.1, 1H), 4.23 (t, *J* = 7.0, 1H), 4.12 (dd, *J* = 8.1, 1.8, 1H), 4.02 (dd, *J* = 9.5, 8.0, 1H), 3.23 (dd, *J* = 9.4, 8.3, 1H), 2.27-2.16 (m, 1H), 1.47 (s, 9H), 1.01 (d, *J* = 6.7, 3H), 0.95 (d, *J* = 6.8, 3H); ^{13}C NMR (126 MHz, $CDCl_3$) δ 169.4, 168.6, 158.1, 143.5, 141.3, 127.9, 127.1, 125.0, 120.1, 82.4, 73.6, 67.7, 61.1, 60.8, 47.5, 47.0, 28.1, 27.5, 19.3, 19.0; HRMS (ESI^+) calcd *m/z* for $C_{28}H_{35}N_2O_6^+$ $[M+H]^+$, 495.2490 found 495.2496.

***N*-Fmoc-(3*R*, 4*S*, 2'*S*)-Hgl-Val-*t*Bu [(3*R*, 4*S*, 2'*S*)-19]**

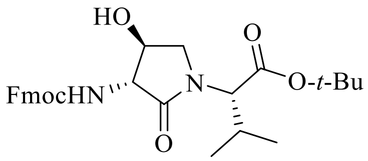

(3*R*, 4*S*, 2'*S*)-**19**

Employing the previously described procedure,[3] (2*R*,2'*R*)-oxiranylglycine (2*R*,2'*R*)-**18** (1 eq., 225 mg, 0.637 mmol) was converted to (3*R*,4*S*,2'*S*)-lactam (3*R*,4*S*,2'*S*)-**7** (258 mg, 0.522 mmol, 82%): R_f = 0.39 (3% THF in DCM); $[\alpha]_D^{20}$ –44.1° (*c* 1.2, $CHCl_3$); ^{1}H NMR (500 MHz, $CDCl_3$) δ 7.77 (dd, *J* = 7.6, 0.7, 2H), 7.59 (dd, *J* = 7.5, 0.6, 2H), 7.41 (td, *J* = 7.5, 0.6, 2H), 7.33 (td, *J* = 7.4, 0.9, 2H), 5.75 (s, 1H), 5.02 (s, 1H), 4.44 (d, *J* = 7.0, 2H), 4.33 (d, *J* = 10.2, 1H), 4.28 (t, *J* = 8.1, 1H), 4.23 (t, *J* = 6.9, 1H), 4.09 (dd, *J* = 8.1, 1.7, 1H), 3.67 (dd, *J* = 10.2, 7.9, 1H), 3.37 (dd, *J* = 10.2, 8.5, 1H), 2.22-2.12 (m, 1H), 1.47 (s, 9H), 1.02 (d, *J* = 6.6, 3H), 0.86 (d, *J* = 6.7, 3H); ^{13}C NMR (126 MHz, $CDCl_3$) δ 169.4, 168.6, 158.1, 143.5, 141.3, 127.9, 127.1, 125.0, 120.1, 82.4, 73.6, 67.7, 61.1, 60.8, 47.5, 47.0, 28.1, 27.5, 19.3, 19.0; HRMS (ESI^+) calcd *m/z* for $C_{28}H_{35}N_2O_6^+$ $[M+H]^+$, 495.2490 found 495.2480.

_N_-Fmoc-(3_S_, 4_R_, 2'_R_)-Hgl-Val-_t_Bu [(3_S_, 4_R_, 2'_R_)-19]

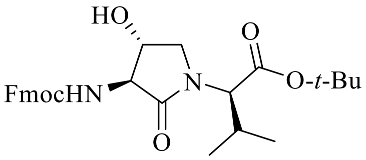

(3*S*, 4*R*, 2'*R*)-**19**

Employing the previously described procedure,[3] (2*S*,2'*S*)-oxiranylglycine (2*S*,2'*S*)-**18** (1 eq., 150 mg, 0.424 mmol) was converted to (3*S*,4*R*,2'*R*)-lactam (3*S*,4*R*,2'*R*)-**7** (151 mg, 0.305 mmol, 72%): R_f = 0.37 (3% THF in DCM); $[\alpha]_D^{20}$ 37.9° (*c* 1.3, $CHCl_3$); ^{1}H NMR (500 MHz, $CDCl_3$) δ 7.77 (d, *J* = 7.5, 2H), 7.59 (d, *J* = 7.5, 2H), 7.41 (t, *J* = 7.5, 2H), 7.33 (td, *J* = 7.4, 0.7, 2H), 5.77 (s, 1H), 5.02 (s, 1H), 4.44 (d, *J* = 7.0, 2H), 4.33 (d, *J* = 10.2, 1H), 4.28 (t, *J* = 8.1, 1H), 4.23 (t, *J* = 6.9, 1H), 4.09 (dd, *J* = 8.1, 1.6, 1H), 3.67 (dd, *J* = 10.2, 7.9, 1H), 3.37 (dd, *J* = 10.1, 8.5, 1H), 2.24-2.11 (m, 1H), 1.47 (s, 9H), 1.02 (d, *J* = 6.6, 3H), 0.86 (d, *J* = 6.7, 3H); ^{13}C NMR (75 MHz, $CDCl_3$) δ 169.4, 168.6, 158.1, 143.5, 141.3, 127.8, 127.1, 125.0, 120.1, 82.4, 73.6, 67.7, 61.0, 60.8, 47.5, 47.0, 28.0, 27.5, 19.3, 19.0; HRMS (ESI^+) calcd *m/z* for $C_{28}H_{35}N_2O_6^+$ $[M+H]^+$, 495.2490 found 495.2499.

_N_-Fmoc-(3_S_, 4_R_, 2'_S_)-Hgl-Val-_t_Bu [(3_S_, 4_R_, 2'_S_)-19]

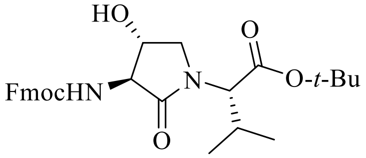

(3*S*, 4*R*, 2'*S*)-**19**

Employing the previously described procedure,[3] (2*S*,2'*S*)-oxiranylglycine (2*S*,2'*S*)-**18** (1 eq., 220 mg, 0.623 mmol) was converted to (3*S*,4*R*,2'*S*)-lactam [(3*S*,4*R*,2'*S*)-**19**] (258 mg, 0.523 mmol, 84%): R_f = 0.44 (3% THF in DCM); $[\alpha]_D^{20}$ –18.9° (*c* 1.3, $CHCl_3$); ^{1}H NMR (500 MHz, $CDCl_3$) δ 7.77 (d, *J* = 7.6, 2H), 7.59 (d, *J* = 7.5, 2H), 7.41 (t, *J* = 7.5, 2H), 7.33 (td, *J* = 7.5, 1.0, 2H), 5.74 (s, 1H), 5.01 (s, 1H), 4.45-4.40 (m, 3H), 4.34 (q, *J* = 8.2, 1H), 4.23 (t, *J* = 7.0, 1H), 4.12 (dd, *J* = 8.1, 1.7, 1H), 4.02 (dd, *J* = 9.5, 8.0, 1H), 3.23 (dd, *J* = 9.4, 8.3, 1H), 2.27-2.17 (m, 1H), 1.47 (s, 9H), 1.01 (d, *J* = 6.7, 3H), 0.95 (d, *J* = 6.8, 3H); ^{13}C NMR (126 MHz, $CDCl_3$) δ 169.7, 169.1, 158.2, 143.6, 141.5, 128.0, 127.3, 125.1, 120.2, 82.4, 73.8, 67.8, 60.6, 60.5, 48.2, 47.1, 29.0, 28.2, 19.5, 19.4; HRMS (ESI^+) calcd *m/z* for $C_{28}H_{35}N_2O_6$ $[M+H]^+$, 495.2490 found 495.2493.

_N_-Fmoc-(3_R_, 4_R_, 2'_R_)-Hgl-Val-_t_Bu [(3_R_, 4_R_, 2'_R_)-19]

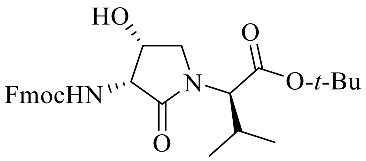

(3*R*, 4*R*, 2'*R*)-**19**

Employing the previously described procedure,[3] (3*R*,4*R*,2'*R*)-benzoate (3*R*,4*R*,2'*R*)-**20** (50 mg, 0.078 mmol) was converted to (3*R*,4*R*,2'*R*)-alcohol (3*R*,4*R*,2'*R*)-**19** (23 mg, 0.047 mmol, 60%): R_f = 0.18 (3% THF in DCM); $[\alpha]_D^{20}$ 10.9° (*c* 1.2, $CHCl_3$), ^{1}H NMR (400 MHz, DMSO) δ 7.89 (d, *J* = 7.5, 2H), 7.81 (t, *J* = 7.4, 2H), 7.42 (t, *J* = 7.4, 2H), 7.37 (d, *J* = 9.1, 1H), 7.33 (t, *J* = 7.4, 2H), 5.33 (d, *J* = 4.2, 1H), 4.43 (dd, *J* = 9.1, 5.1, 1H), 4.31-4.19 (m, 4H), 4.11 (d, *J* = 10.4, 1H), 3.52 (dd, *J* = 10.5, 3.6, 1H), 3.44 (d, *J* = 10.5, 1H), 2.08-1.96 (m, 1H), 1.42 (s, 9H), 0.90 (d, *J* = 6.6, 3H), 0.79 (d, *J* = 6.6, 3H); ^{13}C NMR (75 MHz, $CDCl_3$) δ 169.9, 143.8, 141.3, 127.7, 127.1, 125.7, 119.9, 82.7, 67.6, 67.5, 60.5, 57.2, 50.6, 47.1, 28.7, 28.0, 19.4, 19.3; HRMS (ESI$^+$) calcd *m/z* for $C_{28}H_{35}N_2O_6^+$ [M+H]$^+$, 495.2490 found 495.2502.

_N_-Fmoc-(3_R_, 4_R_, 2'_S_)-Hgl-Val-_t_Bu [(3_R_, 4_R_, 2'_S_)-19]

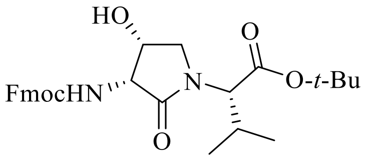

(3*R*, 4*R*, 2'*S*)-**19**

Employing the procedure described in reference[3] (3*R*,4*R*,2'*S*)-benzoate (3*R*,4*R*,2'*S*)-**20** (425 mg, 0.66 mmol) was converted to (3*R*,4*R*,2'*S*)-alcohol (3*R*,4*R*,2'*S*)-**19** (220 mg, 0.44 mmol, 67%): R_f = 0.22 (3% THF in DCM); $[\alpha]_D^{20}$ –45.5° (*c* 1.4, $CHCl_3$); ^{1}H NMR (300 MHz, DMSO) δ 7.89 (d, *J* = 7.5, 2H), 7.81 (dd, *J* = 7.3, 4.2, 2H), 7.42 (t, *J* = 7.2, 2H), 7.33 (m, 3H), 5.43 (d, *J* = 3.7, 1H), 4.36 (dd, *J* = 9.0, 4.8, 1H), 4.32-4.17 (m, 4H), 4.09 (d, *J* = 10.5, 1H), 3.54 (dd, *J* = 10.8, 3.5, 1H), 3.18 (d, *J* = 10.8, 1H), 2.15-1.98 (m, 1H), 1.41 (s, 9H), 0.93 (d, *J* = 6.6, 3H), 0.84 (d, *J* = 6.6, 3H); ^{13}C NMR (75 MHz, $CDCl_3$) δ 170.4, 169.4, 156.7, 143.7, 141.4, 127.8, 127.2, 125.0, 120.0, 82.2, 70.1, 66.5, 61.0, 57.5, 49.8, 47.2, 28.0, 27.7, 19.2, 18.7; HRMS (ESI$^+$) calcd *m/z* for $C_{28}H_{35}N_2O_6$ [M+H]$^+$, 495.2490 found 495.2494.

_N_-Fmoc-(3_S_, 4_S_, 2'_R_)-Hgl-Val-_t_Bu [(3_S_, 4_S_, 2'_R_)-19]

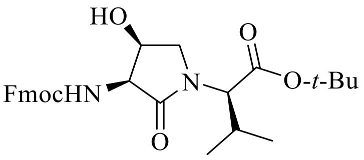

(3*S*, 4*S*, 2'*R*)-**19**

Employing the previously described procedure,[3] (3*S*,4*S*,2'*R*)-benzoate (3*S*,4*S*,2'*R*)-**20** (245 mg, 0.38 mmol) was converted to (3*S*,4*S*,2'*R*)-alcohol (3*S*,4*S*,2'*R*)-**19** (180 mg, 0.36 mmol, 96%): R_f = 0.25 (3% THF in DCM); $[\alpha]_D^{20}$ 30.6° (*c* 1.5, $CHCl_3$); ^{1}H NMR (300 MHz, DMSO) δ 7.89 (d, *J* = 7.5, 2H), 7.81 (dd, *J* = 7.2, 4.2, 2H), 7.42 (t, *J* = 7.3, 2H), 7.37-7.27 (m, 3H), 5.43 (d, *J* = 3.8, 1H), 4.36 (dd, *J* = 8.9, 5.0, 1H), 4.31-4.18 (m, 4H), 4.09 (d, *J* = 10.4, 1H), 3.54 (dd, *J* = 10.9, 3.4, 1H), 3.18 (d, *J* = 11.0, 1H), 2.15-1.95 (m, 1H), 1.41 (s, 9H), 0.93 (d, *J* = 6.6, 3H), 0.84 (d, *J* = 6.6, 3H); ^{13}C NMR (75 MHz, $CDCl_3$) δ 170.4, 169.4, 143.7, 141.4, 141.3, 127.8, 127.2, 125.0, 120.0, 82.2, 70.1, 66.5, 61.0, 57.5, 49.8, 47.1, 28.0, 27.7, 19.2, 18.7; HRMS (ESI$^+$) calcd *m/z* for $C_{28}H_{35}N_2O_6$ $[M+H]^+$, 495.2490 found 495.2500.

***N*-Fmoc-(3*S*, 4*S*, 2'*S*)-Hgl-Val-*t*Bu [(3*S*, 4*S*, 2'*S*)-19]**

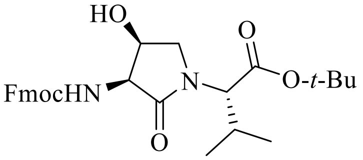

(3*S*, 4*S*, 2'*S*)-**19**

Employing the previously described procedure,[3] (3*S*,4*S*,2'*S*)-benzoate (3*S*,4*S*,2'*S*)-**20** (1 eq., 160 mg, 0.249 mmol) was converted to (3*S*,4*S*,2'*S*)-alcohol (3*S*,4*S*,2'*S*)-**19** (94 mg, 0.19 mmol, 76%): R_f = 0.17 (3% THF in DCM); $[\alpha]_D^{20}$ –13.7° (*c* 0.8, $CHCl_3$); ^{1}H NMR (300 MHz, DMSO) δ 7.89 (d, *J* = 7.5, 2H), 7.80 (m, 2H), 7.46-7.28 (m, 5H), 5.32 (d, *J* = 4.2, 1H), 4.42 (dd, *J* = 9.2, 5.0, 1H), 4.29-4.17 (m, 4H), 4.11 (d, *J* = 10.4, 1H), 3.51 (dd, *J* = 10.1, 3.3, 1H), 3.43 (d, *J* = 10.3, 1H), 2.11-1.93 (m, 1H), 1.42 (s, 9H), 0.90 (d, *J* = 6.6, 3H), 0.78 (d, *J* = 6.6, 3H); ^{13}C NMR (75 MHz, $CDCl_3$) δ 170.1, 156.8, 144.0, 143.8, 141.4, 127.9, 127.3, 125.3, 120.0, 82.9, 67.8, 67.5, 60.6, 57.3, 50.7, 47.3, 28.9, 28.1, 19.6, 19.5; HRMS (ESI$^+$) calcd *m/z* for $C_{28}H_{35}N_2O_6$ $[M+H]^+$, 495.2490 found 495.2492.

(3*R*,4*R*,2'*R*)-*tert*-Butyl 2-[3-(Fmoc)amino-4-*p*-nitrobenzoyloxy-2-oxopyrrolidin-1-yl]-3-methylbutanoate [(3*R*, 4*R*, 2'*R*)-20]

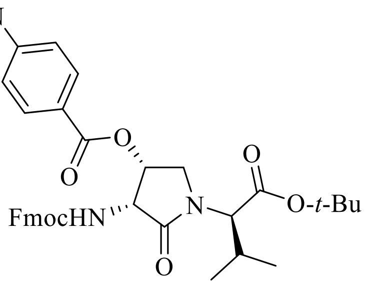

(3*R*, 4*R*, 2'*R*)-**20**

Employing the previously described procedure,[3] (3*R*,4*S*,2'*R*)-alcohol (3*R*,4*S*,2'*R*)-**19** (65 mg, 0.131 mmol) was transformed into (3*R*,4*R*,2'*R*)-benzoate (3*R*,4*R*,2'*R*)-**20** (58.4 mg, 0.0907 mmol, 69%): R_f = 0.22 (30% EtOAc in hexane); $[\alpha]_D^{20}$ –93.9° (*c* 0.2, $CHCl_3$); ^{1}H NMR (300 MHz, $CDCl_3$) δ 8.22 (d, *J* = 8.8, 2H), 8.12 (d, *J* = 8.7, 2H), 7.71 (t, *J* = 7.8, 2H), 7.50 (dd, *J* = 19.8, 7.5, 2H), 7.42-7.29 (m, 2H), 7.24-7.13 (m, 2H), 5.83 (m, 1H), 5.49 (d, *J* = 6.1, 1H), 4.72 (m, 1H), 4.50-4.39 (m, 2H), 4.33 (dd, *J* = 10.5, 7.2, 1H), 4.18 (d, *J* = 7.2, 1H), 4.13 (d, *J* = 5.8, 1H), 3.74 (dd, *J* = 12.1, 3.3, 1H), 2.27-2.08 (m, 1H), 1.36 (s, 9H), 1.01 (d, *J* = 6.7, 3H), 0.93 (d, *J* = 6.7, 3H); ^{13}C NMR (75 MHz, $CDCl_3$) δ 170.0, 169.2, 163.7, 156.2, 150.7, 143.6, 141.3, 134.8, 130.9, 127.7, 127.0, 124.9, 123.5, 120.0, 82.2, 70.4, 67.4, 60.7, 54.9, 48.0, 47.0, 28.9, 27.9, 19.4, 19.2; HRMS (ESI$^+$) calcd *m/z* for $C_{35}H_{38}N_3O_9$ $[M+H]^+$, 644.2603 found 644.2634.

(3*R*,4*R*,2'S)-*tert*-Butyl 2-[3-(Fmoc)amino-4-*p*-nitrobenzoyloxy-2-oxopyrrolidin-1-yl]-3-methylbutanoate [(3*R*, 4*R*, 2'*S*)-20]

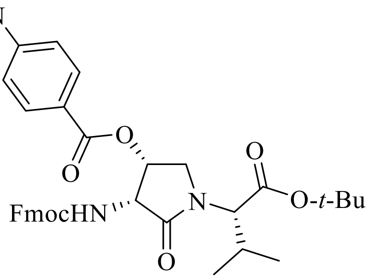

(3*R*, 4*R*, 2'*S*)-**20**

Employing the previously described procedure,[3] (3*R*,4*S*,2'*S*)-alcohol (3*R*,4*S*,2'*S*)-**19** (233 mg, 0.47 mmol) was transformed into (3*R*,4*R*,2'*S*)-benzoate (3*R*,4*R*,2'*S*)-**20** (269 mg, 0.42 mmol, 89%): R_f = 0.31 (30% EtOAc in hexane); $[\alpha]_D^{20}$ –39.4° (*c* 2.5, $CHCl_3$); ^{1}H NMR (300 MHz, $CDCl_3$) δ 8.24 (d, *J* = 8.9, 2H), 8.12 (d, *J* = 8.8, 2H), 7.69 (t, *J* = 8.1, 2H), 7.48 (dd, *J* = 25.2, 7.5, 2H), 7.39-7.28 (m, 2H), 7.24-7.10 (m, 2H), 5.91 (d, *J* = 6.3, 1H), 5.86 (m, 1H), 4.70 (m, 1H), 4.43 (dd, *J* = 10.2, 6.4, 2H), 4.29

(dd, J = 10.6, 7.2, 1H), 4.14 (t, J = 6.8, 1H), 4.00 (dd, J = 12.7, 3.6, 1H), 3.63 (d, J = 12.9, 1H), 2.19-2.05 (m, 1H), 1.47 (s, 9H), 1.00 (d, J = 6.6, 3H), 0.85 (d, J = 6.7, 3H); ^{13}C NMR (75 MHz, $CDCl_3$) δ 170.0, 168.9, 163.6, 156.4, 150.8, 143.6, 141.2, 134.5, 130.8, 127.7, 127.0, 124.9, 123.7, 119.9, 82.6, 70.2, 67.3, 61.1, 55.3, 48.1, 47.0, 28.0, 27.6, 19.1, 18.7; HRMS (ESI^+) calcd *m/z* for $C_{35}H_{38}N_3O_9^+$ $[M+H]^+$, 644.2603 found 644.2592.

(3*S*,4*S*,2'*R*)-*tert*-Butyl 2-[3-(Fmoc)amino-4-*p*-nitrobenzoyloxy-2-oxopyrrolidin-1-yl]-3-methylbutanoate [(3*S*, 4*S*, 2'*R*)-20]

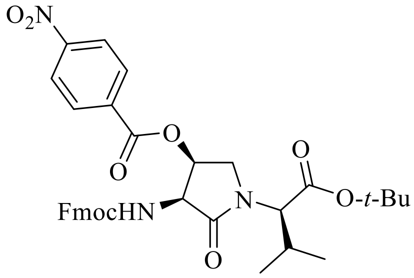

(3*S*, 4*S*, 2'*R*)-**20**

Employing the previously described procedure,[3] (3*S*,4*R*,2'*R*)-alcohol (3*S*,4*R*,2'*R*)-**19** (233 mg, 0.471 mmol) was transformed into (3*S*,4*S*,2'*R*)-benzoate (3*S*,4*S*,2'*R*)-**20** (260 mg, 0.404 mmol, 86%): R_f = 0.29 (30% EtOAc in hexane); $[\alpha]_D^{20}$ 50.2° (*c* 1.3, $CHCl_3$); ^{1}H NMR (300 MHz, $CDCl_3$) δ 8.25 (d, J = 8.8, 2H), 8.11 (d, J = 8.7, 2H), 7.70 (t, J = 8.2, 2H), 7.49 (dd, J = 24.3, 7.5, 2H), 7.41-7.28 (m, 2H), 7.25-7.11 (m, 2H), 5.88 (t, J = 4.6, 1H), 5.69 (d, J = 6.2, 1H), 4.69 (t, J = 5.4, 1H), 4.42 (d, J = 9.6, 2H), 4.31 (dd, J = 10.6, 7.1, 1H), 4.15 (t, J = 6.8, 1H), 4.00 (dd, J = 13.0, 3.4, 1H), 3.64 (d, J = 12.7, 1H), 2.22-2.05 (m, 1H), 1.48 (s, 9H), 1.01 (d, J = 6.5, 3H), 0.86 (d, J = 6.7, 3H); ^{13}C NMR (75 MHz, $CDCl_3$) δ 170.1, 168.9, 163.5, 156.1, 150.8, 143.6, 141.3, 134.4, 130.8, 127.7, 126.9, 124.9, 123.7, 119.9, 82.7, 70.3, 67.3, 61.1, 55.3, 48.1, 47.0, 28.1, 27.6, 19.1, 18.7; HRMS (ESI^+) calcd *m/z* for $C_{35}H_{38}N_3O_9$ $[M+H]^+$, 644.2603 found 644.2603.

(3*S*,4*S*,2'*S*)-*tert*-Butyl 2-[3-(Fmoc)amino-4-*p*-nitrobenzoyloxy-2-oxopyrrolidin-1-yl]-3-methylbutanoate [(3*S*, 4*S*, 2'*S*)-20]

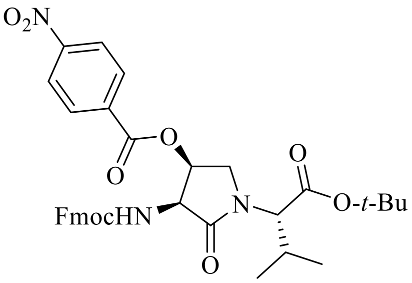

(3*S*, 4*S*, 2'*S*)-**20**

Employing the previously described procedure,[3] (3*S*,4*R*,2'*S*)-alcohol (3*S*,4*R*,2'*S*)-**19** (1 eq., 200 mg, 0.4 mmol) was converted to (3*S*,4*S*,2'*S*)-benzoate (3*S*,4*S* 2'*S*)-**20** (200 mg, 0.3 mmol, 77%): R_f = 0.25 (30% EtOAc in hexane); $[\alpha]_D^{20}$ 40.7° (*c* 1.1, $CHCl_3$); ^{1}H NMR (300 MHz, $CDCl_3$) δ 8.22 (dt, *J* = 8.8, 2.0, 2H), 8.12 (d, *J* = 8.8, 2H), 7.71 (t, *J* = 7.7, 2H), 7.50 (dd, *J* = 19.7, 7.6, 2H), 7.43-7.28 (m, 2H), 7.25-7.11 (m, 2H), 5.83 (m, 1H), 5.51 (d, *J* = 5.9, 1H), 4.74 (m, 1H), 4.44 (m, 2H), 4.34 (dd, *J* = 10.7, 6.9, 1H), 4.18 (d, *J* = 6.7, 1H), 4.13 (d, *J* = 3.4, 1H), 3.74 (dd, *J* = 12.4, 3.3, 1H), 2.30-2.04 (m, 1H), 1.36 (s, 9H), 1.01 (d, *J* = 6.7, 3H), 0.94 (d, *J* = 6.7, 3H); ^{13}C NMR (75 MHz, $CDCl_3$) δ 170.1, 169.2, 167.0, 163.7, 156.2, 150.7, 143.6, 141.3, 134.8, 131.0, 127.7, 127.0, 124.9, 123.6, 120.0, 82.2, 70.4, 67.4, 60.7, 55.0, 48.0, 47.0, 28.9, 27.9, 19.4, 19.2; HRMS (ESI^+) calcd *m/z* for $C_{35}H_{38}N_3O_9$ $[M+H]^+$, 644.2603 found 644.2599.

References:

1. (a) Dola, V. R.; Soni, A.; Agarwal, P.; Ahmad, H.; Raju, K. S. R.; Rashid, M.; Wahajuddin, M.; Srivastava, K.; Haq, W.; Dwivedi, A., *Antimicrob. Agents Chemother.* **2017,** *61*, e01152-16; (b) Pícha, J.; Vaněk, V.; Buděšínský, M.; Mládková, J.; Garrow, T. A.; Jiráček, J., *European J. Med. Chem.* **2013,** *65*, 256-275.
2. Keller, O.; Keller, W. E.; Look, G. v.; Wersin, G., *Org. Synth.* **2003,** *63*, 160-160.
3. Geranurimi, A.; Lubell, W. D., *Org. Lett.* **2018,** *20*, 6126−6129..
4. Sicherl, F.; Cupido, T.; Albericio, F., *Chem. Commun.* **2010,** *46*, 1266-1268.
5. (a) Carrasco, M.; Jones, R. J.; Kamel, S.; Rapoport, H.; Truong, T., *Org. Synth.* **1992**, 29-29; (b) Lumbroso, A.; Coeffard, V.; Le Grognec, E.; Beaudet, I.; Quintard, J.-P., *Tetrahedron Lett.* **2010,** *51*, 3226-3228.
6. Buglioni, L.; Bizet, V.; Bolm, C., *Advanced Synthesis & Catalysis* **2014,** *356*, 2209-2213.
7. Lamborelle, N.; Simon, J. F.; Luxen, A.; Monbaliu, J.-C. M., *Org. Biomol. Chem.* **2015,** *13*, 11602-11606.
8. Organ, M. G.; Xu, J.; N'Zemba, B., *Tetrahedron Lett.* **2002,** *43*, 8177-8180.
9. St-Cyr, D. J.; Jamieson, A. G.; Lubell, W. D., *Org. Lett.* **2010,** *12*, 1652-1655.

LC-MS

[(3*R*)-Agl[3]]-101.10 [(3*R*, 2'*R*)-5]

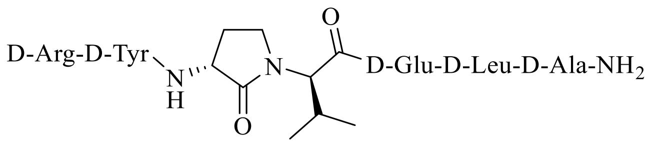

[(3*R*, 2'*R*)-5] LCMS chromatogram [10-90% MeOH (0.1% FA)/water (0.1% FA), 14 min]; RT = 8 on a Sunfire C18 analytical column (100Å, 3.5 µm, 4.6 mm X 100 mm).

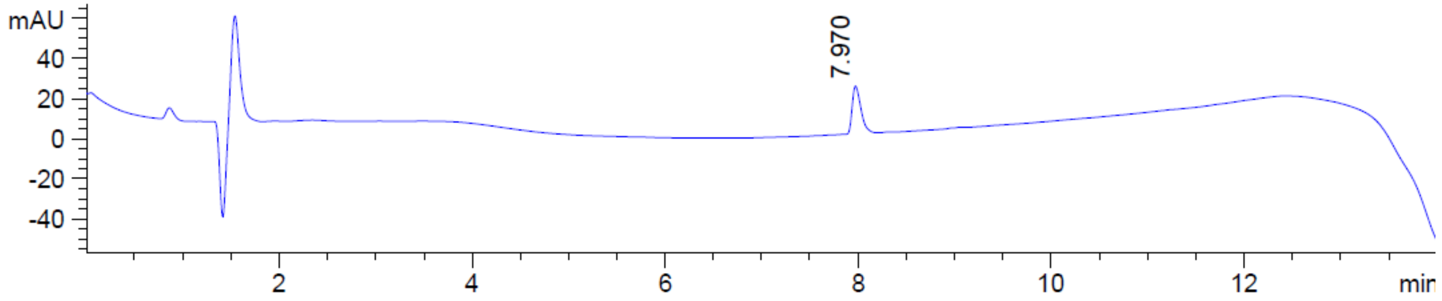

[(3*R*, 2'*R*)-5] LCMS chromatogram [10-90% MeCN (0.1% FA)/water (0.1% FA), 14 min]; RT = 5.6 on a Sunfire C18 analytical column (100Å, 3.5 µm, 4.6 mm X 100 mm).

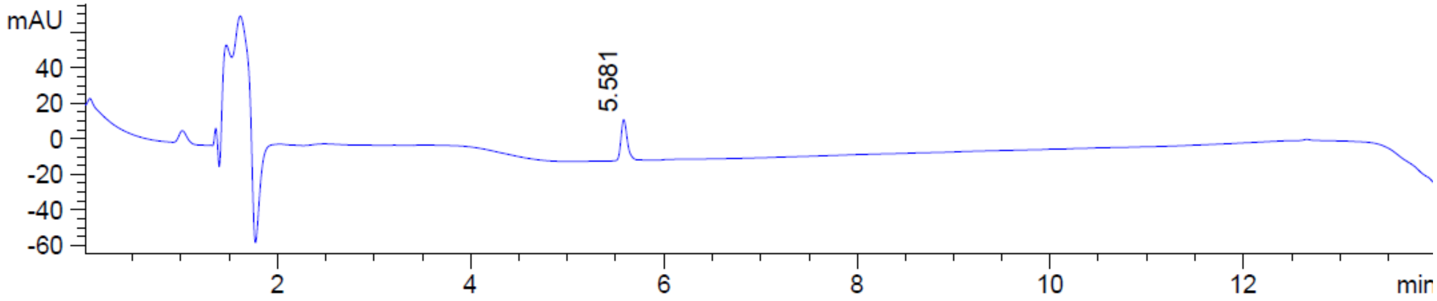

[(3*R*)-Agl[3]-L-Val]-1 [(3*R*, 2'*S*)-5]

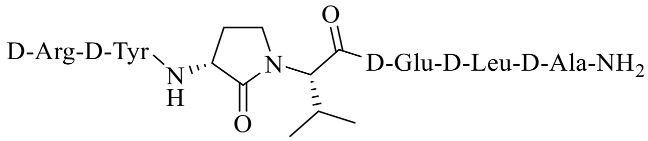

[(3*R*, 2'*S*)-5] LCMS chromatogram [10-90% MeOH (0.1% FA)/water (0.1% FA), 14 min]; RT = 7.6 on a Sunfire C18 analytical column (100Å, 3.5 µm, 4.6 mm X 100 mm).

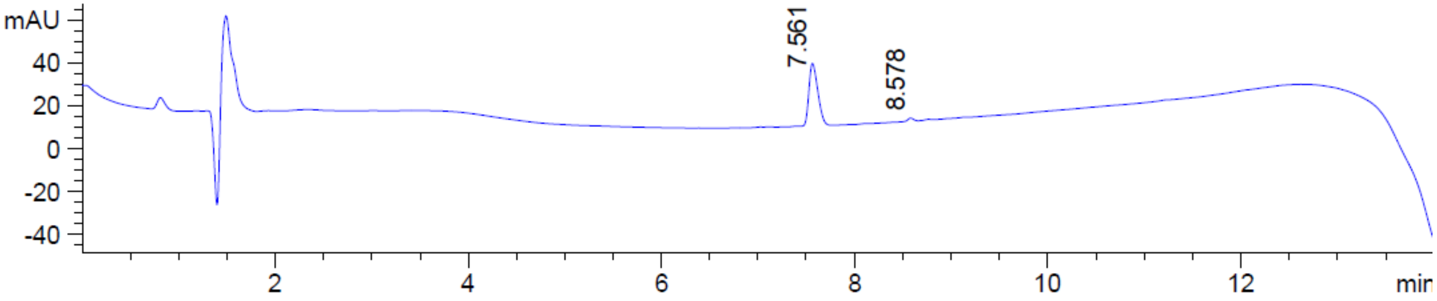

[(3*R*, 2'*S*)-5] LCMS chromatogram [10-90% MeCN (0.1% FA)/water (0.1% FA), 14 min]; RT = 5.3 on a Sunfire C18 analytical column (100Å, 3.5 µm, 4.6 mm X 100 mm).

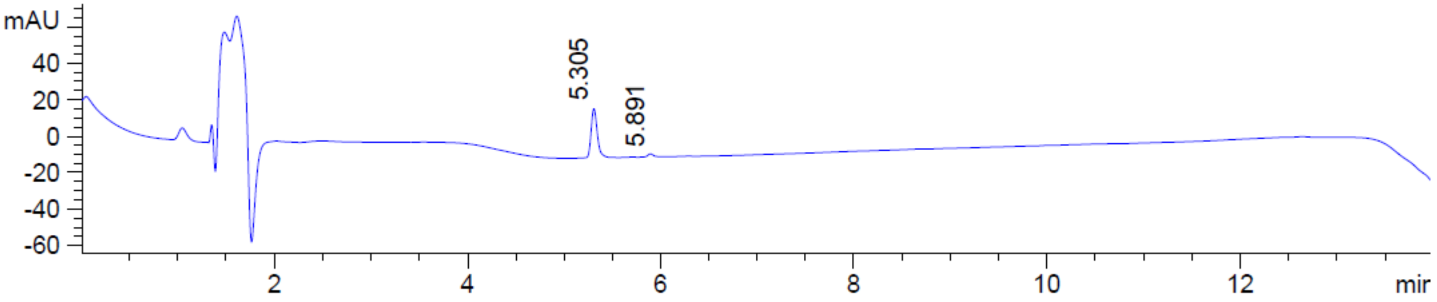

[(3*S*)-Agl[3]]-1 [(3*S*, 2’*R*)-5]

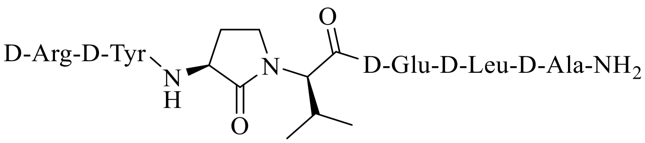

[(3*S*, 2’*R*)-5] LCMS chromatogram [10-90% MeOH (0.1% FA)/water (0.1% FA), 14 min]; RT = 8 on a Sunfire C18 analytical column (100Å, 3.5 µm, 4.6 mm X 100 mm).

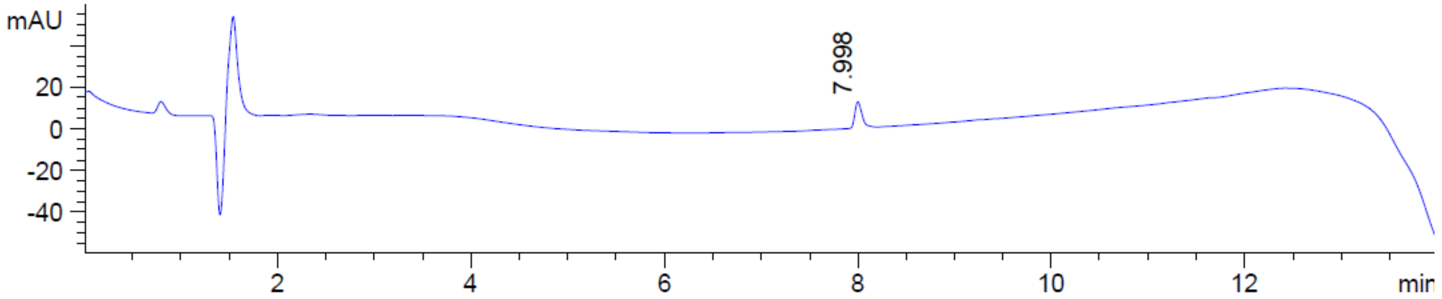

[(3*S*, 2’*R*)-5] LCMS chromatogram [10-90% MeCN (0.1% FA)/water (0.1% FA), 14 min]; RT = 5.4 on a Sunfire C18 analytical column (100Å, 3.5 µm, 4.6 mm X 100 mm).

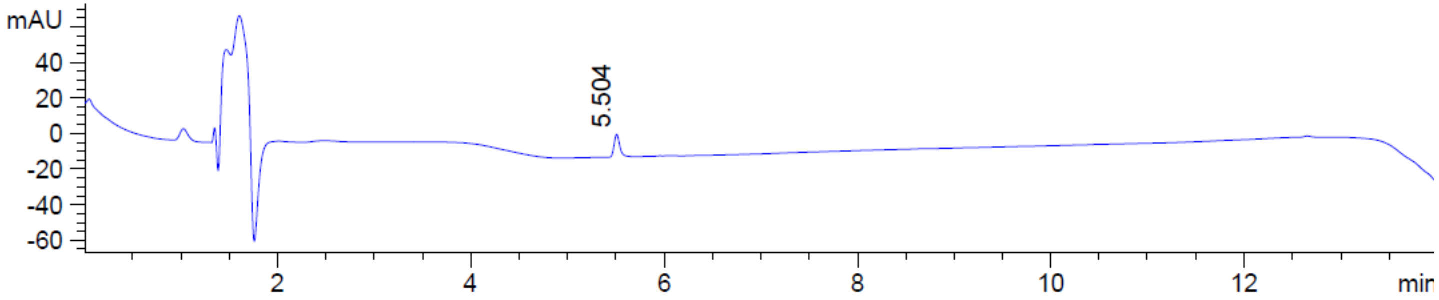

[(3*S*)-Agl[3]-L-Val]-1 [(3*S*, 2’*S*)-5]

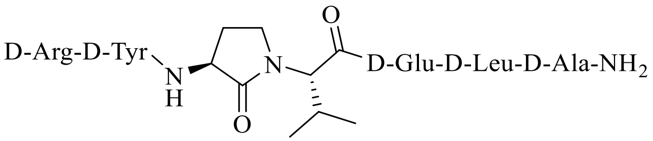

[(3*S*, 2’*S*)-5] LCMS chromatogram [10-90% MeOH (0.1% FA)/water (0.1% FA), 10 min]; RT = 7.9 on a Sunfire C18 analytical column (100Å, 3.5 µm, 4.6 mm X 100 mm).

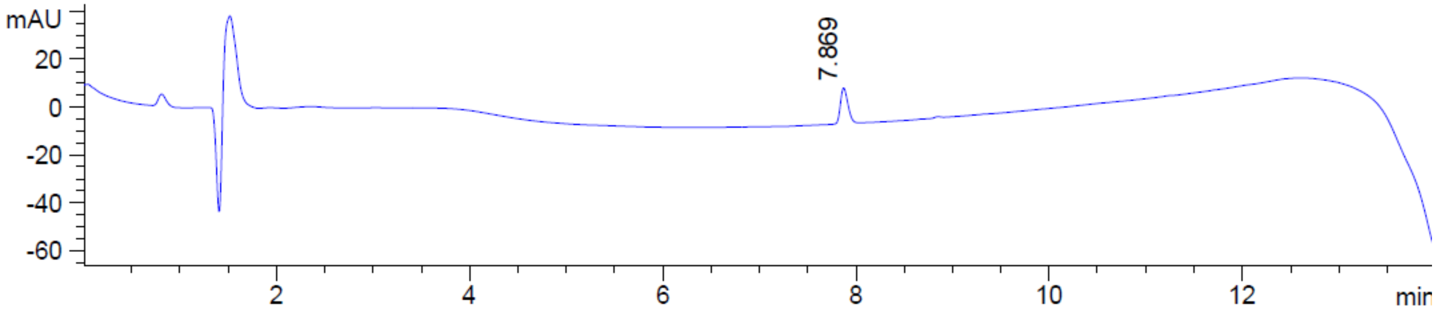

[(3*S*, 2’*S*)-5] LCMS chromatogram [10-90% MeCN (0.1% FA)/water (0.1% FA), 14 min]; RT = 5.5 on a Sunfire C18 analytical column (100Å, 3.5 µm, 4.6 mm X 100 mm).

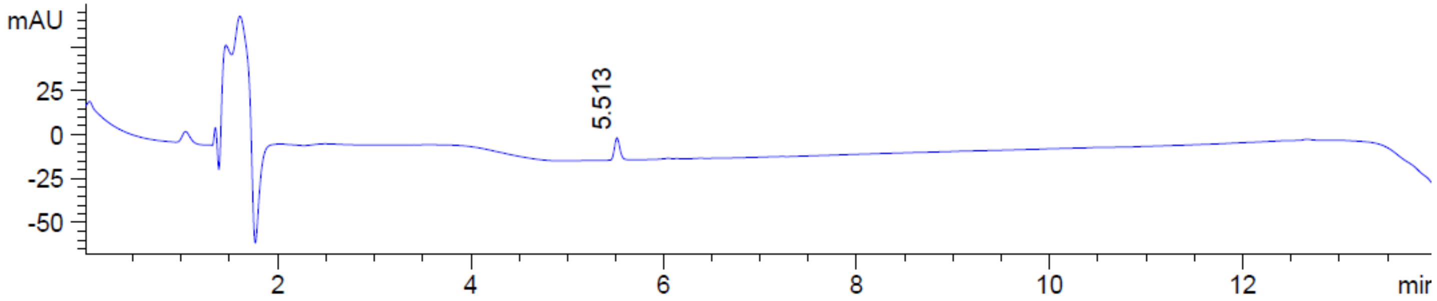

[(3*R*, 4*R*)-Hgl^{3}]-1 [(3*R*, 4*R*, 2'*R*)-6]

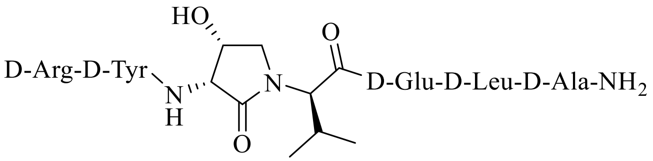

[(3*R*, 4*R*, 2'*R*)-6] LCMS chromatogram [10-90% MeOH (0.1% FA)/water (0.1% FA), 14 min]; RT = 7.8 on a Sunfire C18 analytical column (100Å, 3.5 µm, 4.6 mm X 100 mm).

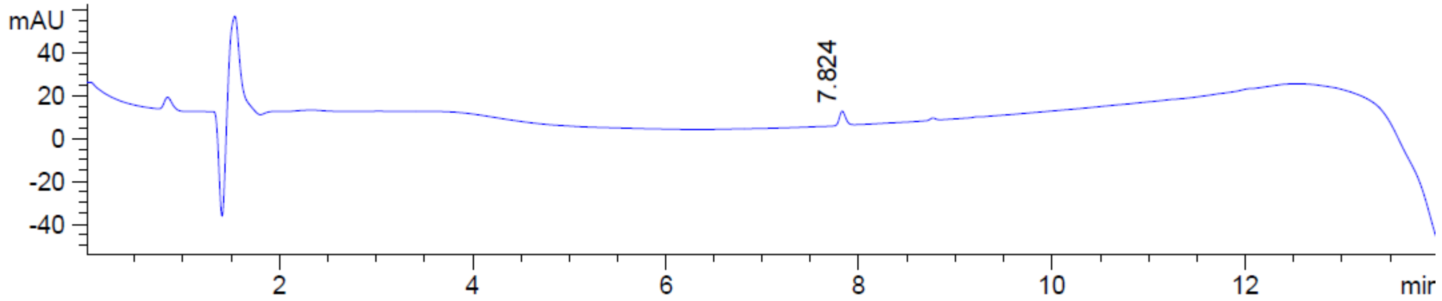

[(3*R*, 4*R*, 2'*R*)-6] LCMS chromatogram [10-90% MeCN (0.1% FA)/water (0.1% FA), 14 min]; RT = 5.4 on a Sunfire C18 analytical column (100Å, 3.5 µm, 4.6 mm X 100 mm).

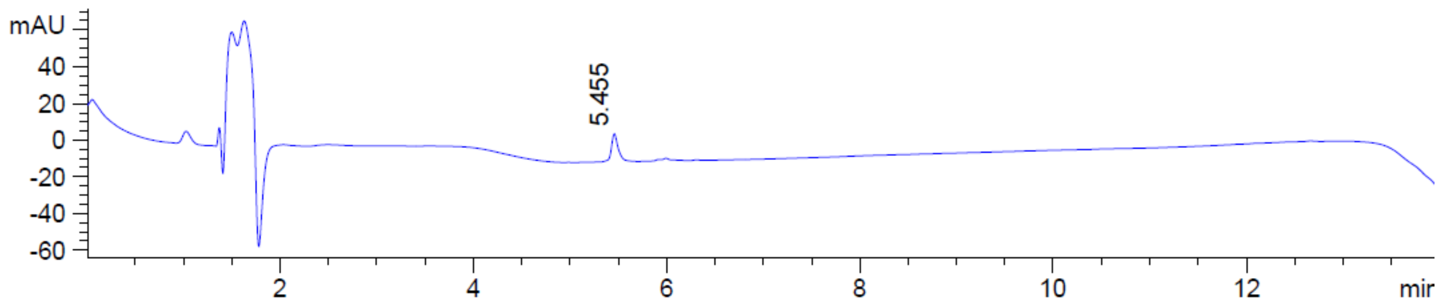

[(3*R*, 4*S*)-Hgl^{3}]-1 [(3*R*, 4*S*, 2’*R*)-6]

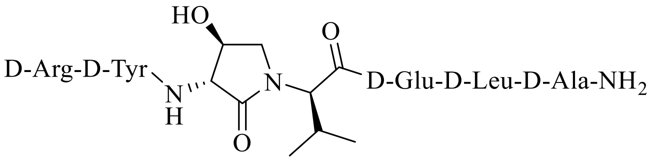

[(3*R*, 4*S*, 2’*R*)-6] LCMS chromatogram [10-90% MeOH (0.1% FA)/water (0.1% FA), 14 min]; RT = 7.5 on a Sunfire C18 analytical column (100Å, 3.5 µm, 4.6 mm X 100 mm).

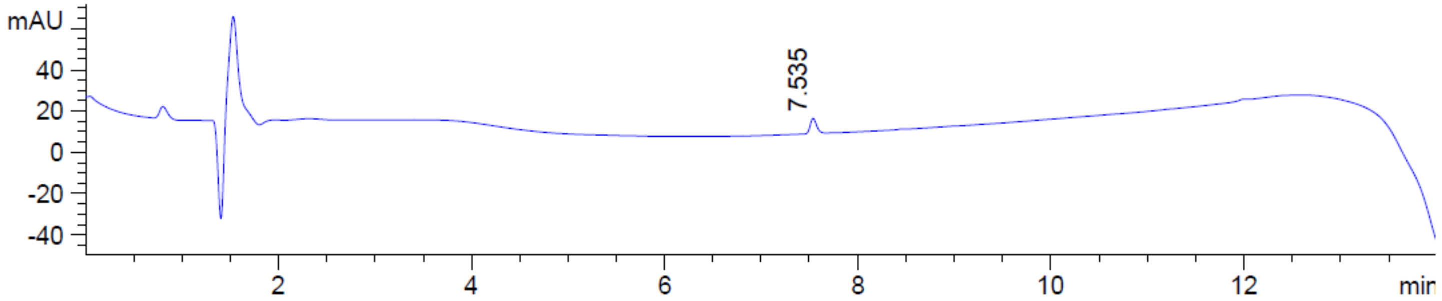

[(3*R*, 4*S*, 2’*R*)-6] LCMS chromatogram [10-90% MeCN (0.1% FA)/water (0.1% FA), 14 min]; RT = 5.2 on a Sunfire C18 analytical column (100Å, 3.5 µm, 4.6 mm X 100 mm).

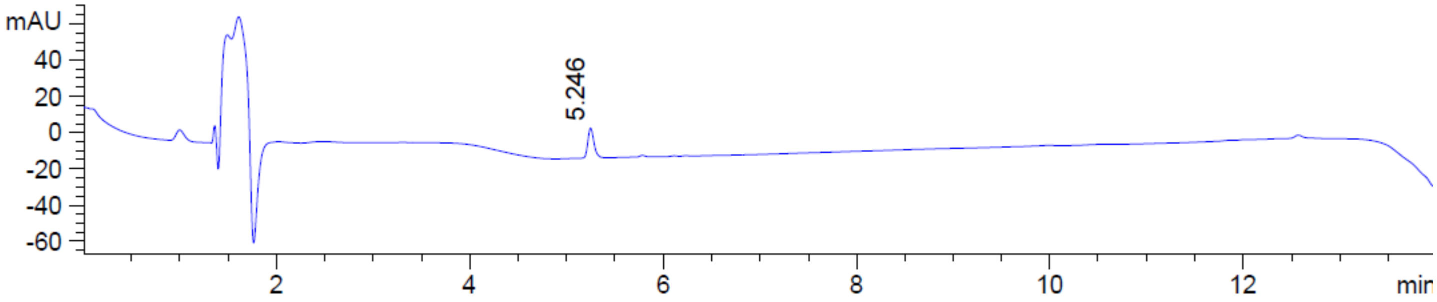

[(3*R*, 4*R*)-Hgl^{3}-(*S*)-Val]-1 [(3*R*, 4*R*, 2'*S*)-6]

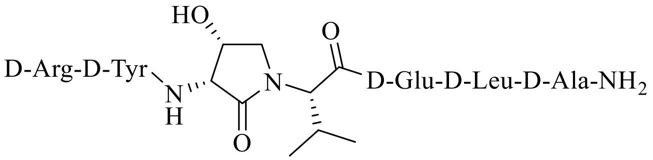

[(3*R*, 4*R*, 2'*S*)-6] LCMS chromatogram [10-90% MeOH (0.1% FA)/water (0.1% FA), 14 min]; RT = 6.8 on a Sunfire C18 analytical column (100Å, 3.5 µm, 4.6 mm X 100 mm).

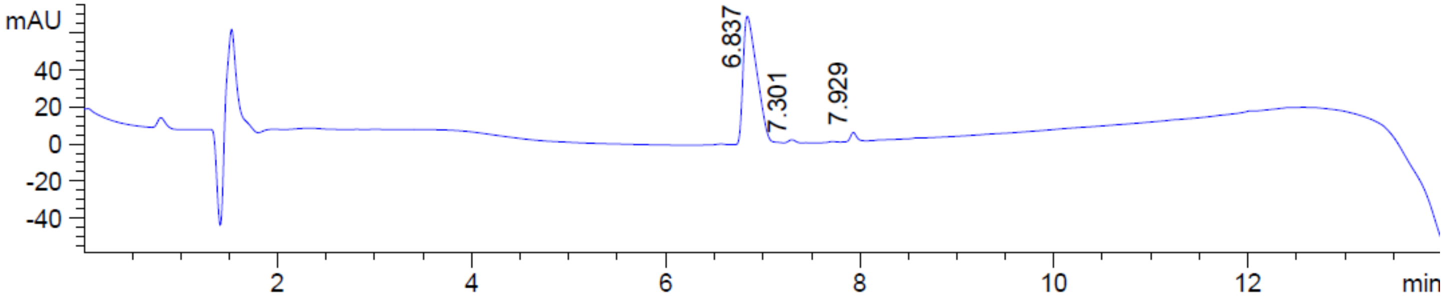

[(3*R*, 4*R*, 2'*S*)-6] LCMS chromatogram [10-90% MeCN (0.1% FA)/water (0.1% FA), 14 min]; RT = 4.9 on a Sunfire C18 analytical column (100Å, 3.5 µm, 4.6 mm X 100 mm).

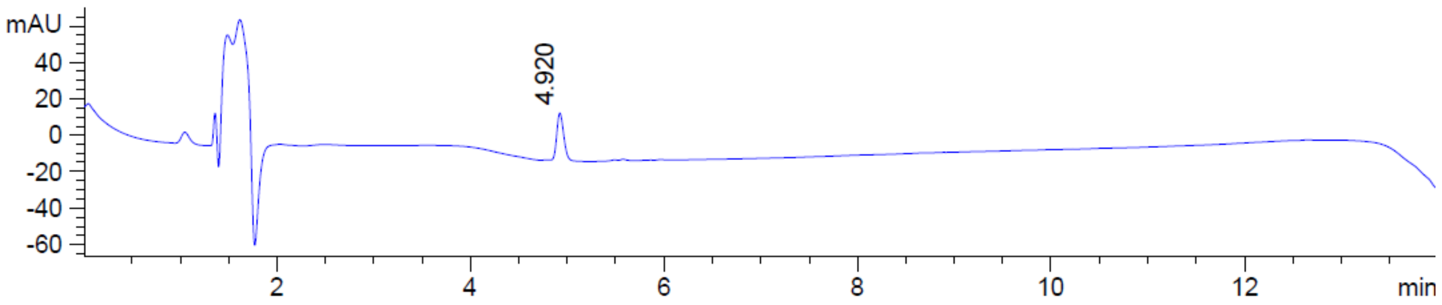

[(3*R*, 4*S*)-Hgl[3]-(*S*)-Val]-1 [(3*R*, 4*S*, 2'*S*)-6]

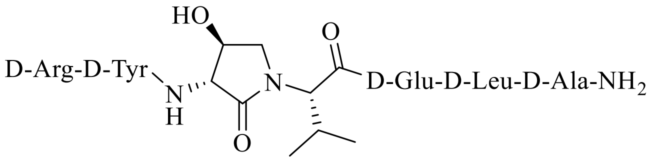

[(3*R*, 4*S*, 2'*S*)-6] LCMS chromatogram [10-90% MeOH (0.1% FA)/water (0.1% FA), 14 min]; RT = 6.9 on a Sunfire C18 analytical column (100Å, 3.5 µm, 4.6 mm X 100 mm).

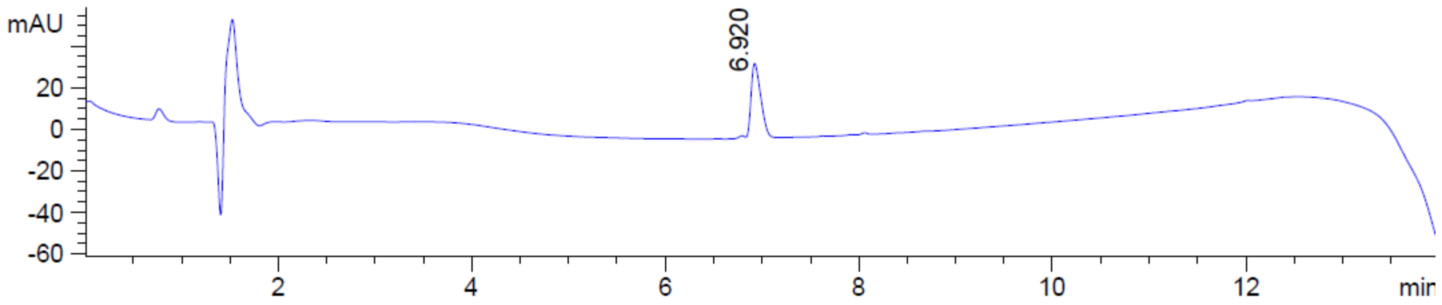

[(3*R*, 4*S*, 2'*S*)-6] LCMS chromatogram [10-90% MeCN (0.1% FA)/water (0.1% FA), 14 min]; RT = 4.9 on a Sunfire C18 analytical column (100Å, 3.5 µm, 4.6 mm X 100 mm).

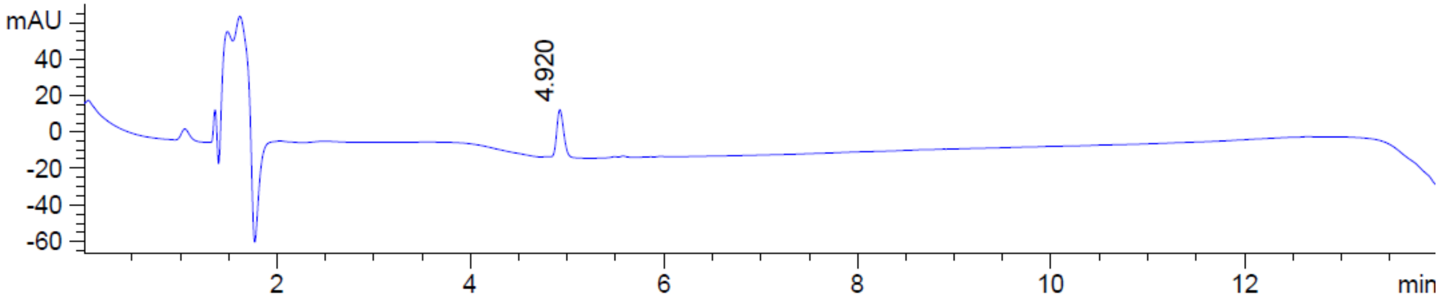

[(3*S*, 4*R*)-Hgl^{3}]-1 [(3*S*, 4*R*, 2’*R*)-6]

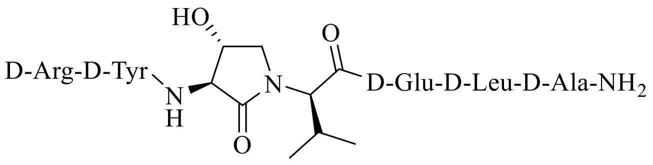

[(3*S*, 4*R*, 2’*R*)-6] LCMS chromatogram [10-90% MeOH (0.1% FA)/water (0.1% FA), 14 min]; RT = 7.8 on a Sunfire C18 analytical column (100Å, 3.5 µm, 4.6 mm X 100 mm).

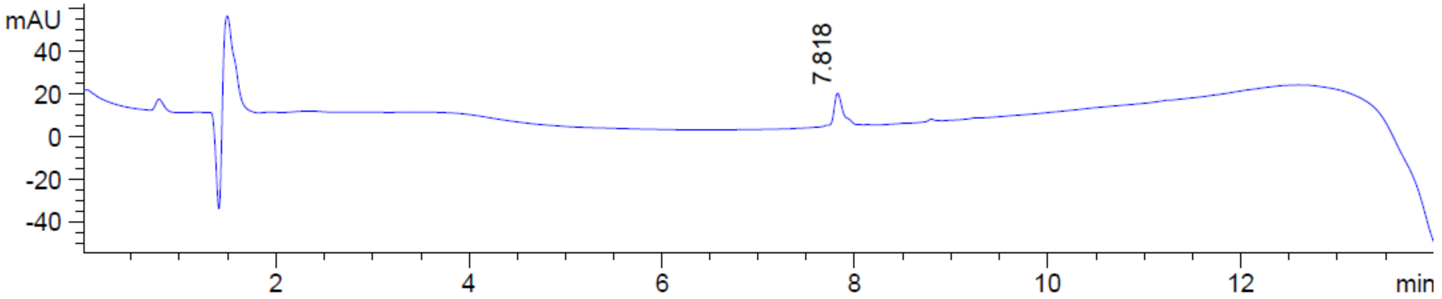

[(3*S*, 4*R*, 2’*R*)-6] LCMS chromatogram [10-90% MeCN (0.1% FA)/water (0.1% FA), 14 min]; RT = 5.4 on a Sunfire C18 analytical column (100Å, 3.5 µm, 4.6 mm X 100 mm).

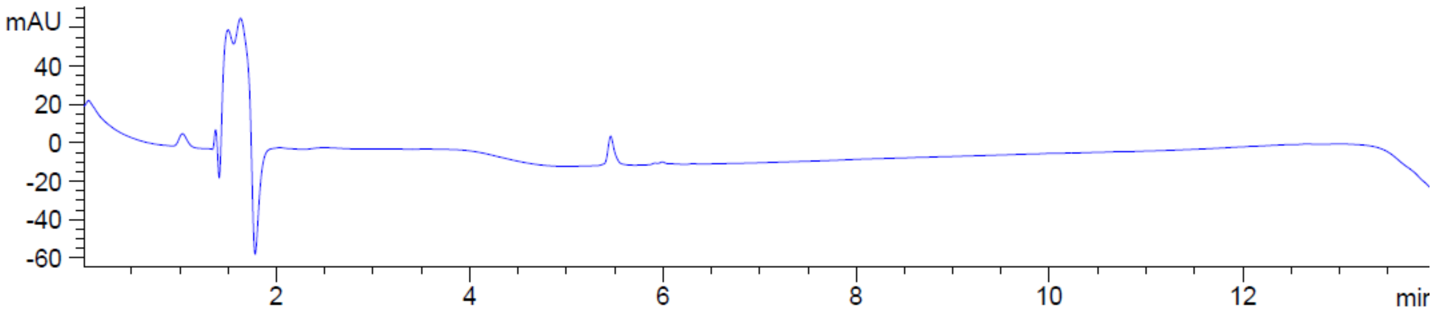

[(3*S*, 4*S*)-Hgl[3]]-1 [(3*S*, 4*S*, 2'*R*)-6]

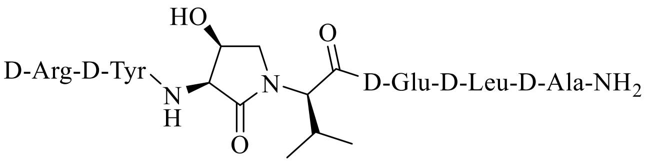

[(3*S*, 4*S*, 2'*R*)-6] LCMS chromatogram [10-90% MeOH (0.1% FA)/water (0.1% FA), 14 min]; RT = 7.3 on a Sunfire C18 analytical column (100Å, 3.5 µm, 4.6 mm X 100 mm).

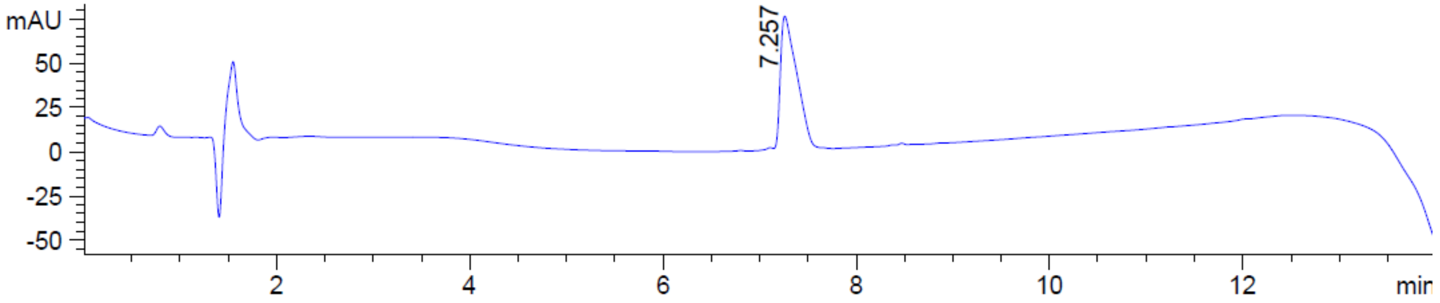

[(3*S*, 4*S*, 2'*R*)-6] LCMS chromatogram [10-90% MeCN (0.1% FA)/water (0.1% FA), 14 min]; RT = 5.2 on a Sunfire C18 analytical column (100Å, 3.5 µm, 4.6 mm X 100 mm).

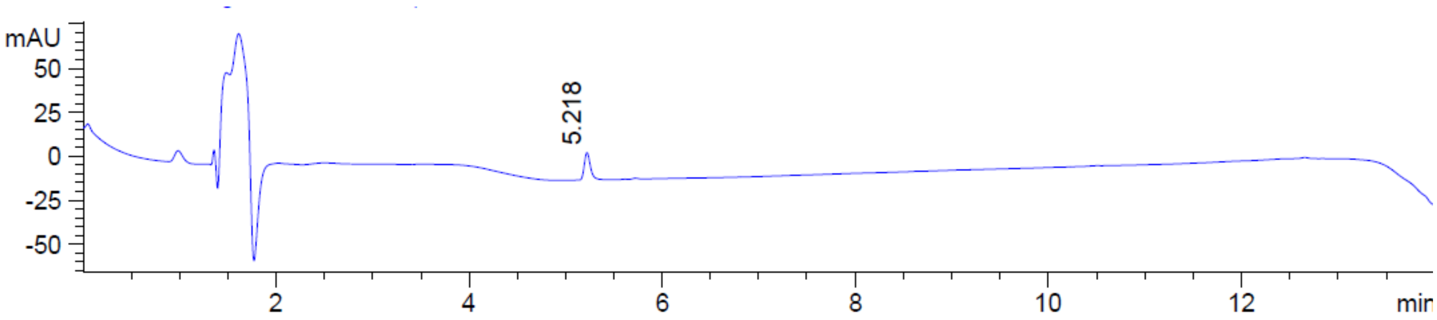

[(3*S*, 4*R*)-Hgl^{3}-(*S*)-Val]-1 [(3*S*, 4*R*, 2’*S*)-6]

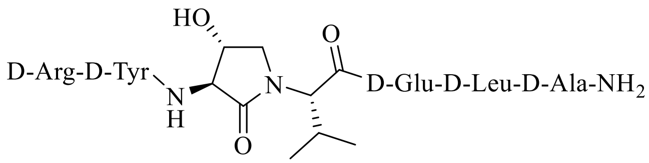

[(3*S*, 4*R*, 2’*S*)-6] LCMS chromatogram [10-90% MeOH (0.1% FA)/water (0.1% FA), 14 min]; RT = 7 on a Sunfire C18 analytical column (100Å, 3.5 µm, 4.6 mm X 100 mm).

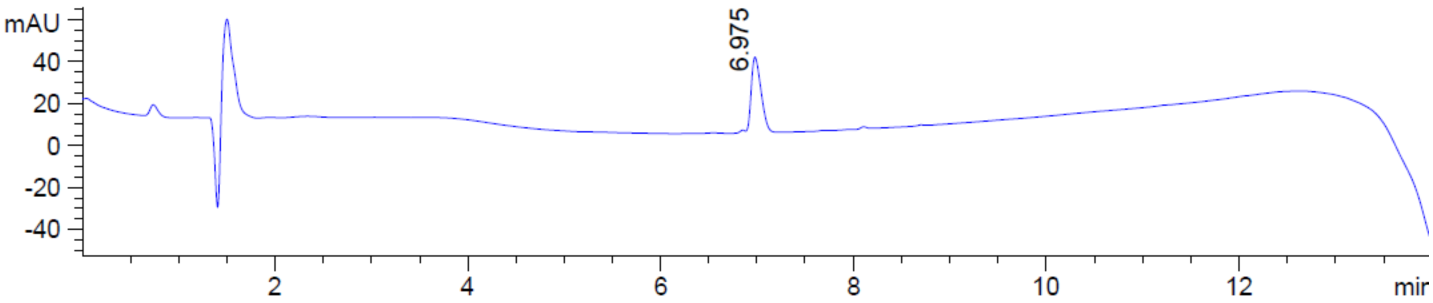

[(3*S*, 4*R*, 2’*S*)-6] LCMS chromatogram [10-90% MeCN (0.1% FA)/water (0.1% FA), 14 min]; RT = 4.9 on a Sunfire C18 analytical column (100Å, 3.5 µm, 4.6 mm X 100 mm).

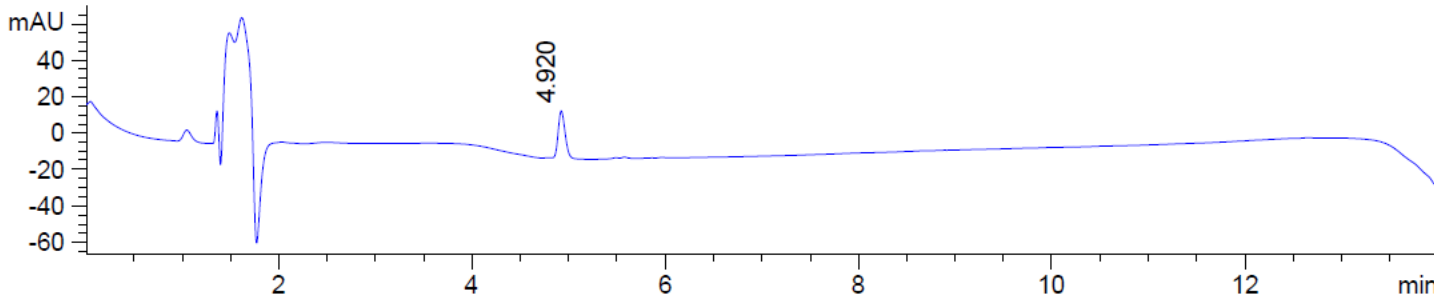

[(3*S*, 4*S*)-Hgl^{3}-(*S*)-Val]-1 [(3*S*, 4*S*, 2'*S*)-6]

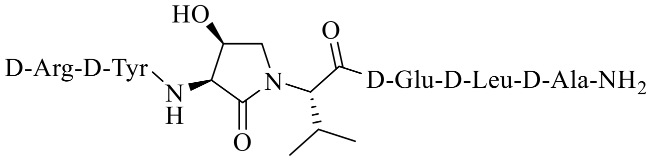

[(3*S*, 4*S*, 2'*S*)-6] LCMS chromatogram [10-90% MeOH (0.1% FA)/water (0.1% FA), 14 min]; RT = 6.6 on a Sunfire C18 analytical column (100Å, 3.5 µm, 4.6 mm X 100 mm).

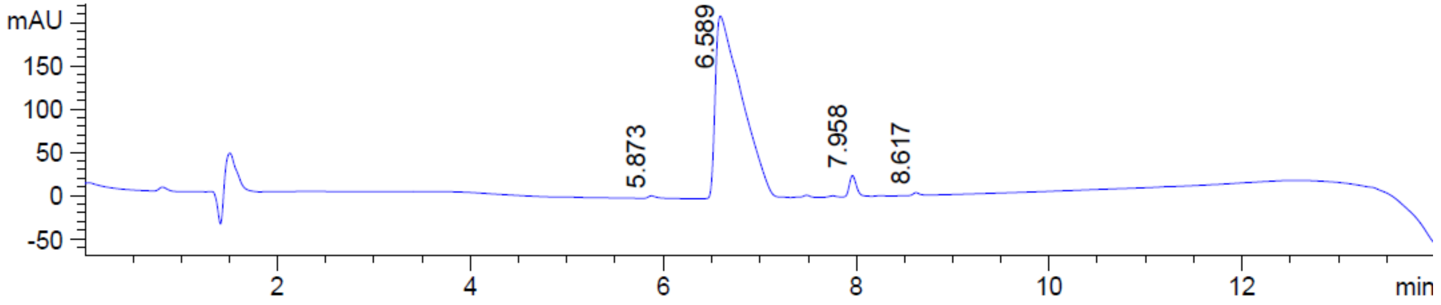

[(3*S*, 4*S*, 2'*S*)-6] LCMS chromatogram [10-90% MeCN (0.1% FA)/water (0.1% FA), 14 min]; RT = 5.2 on a Sunfire C18 analytical column (100Å, 3.5 µm, 4.6 mm X 100 mm).

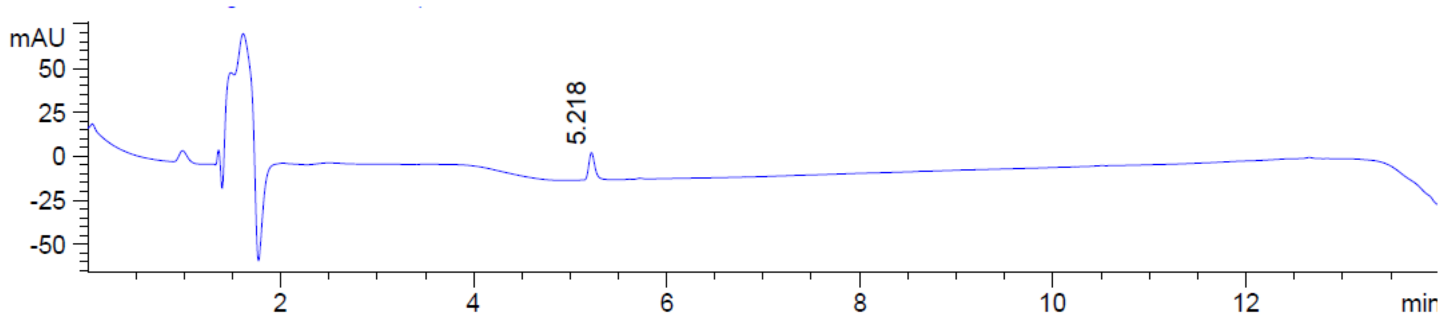
mAU
50
25
0
-25
-50
5.218
2
4
6
8
10
12
min

^{1}H NMR (300 MHz, MeOD) **(*R*, *R*)-7**

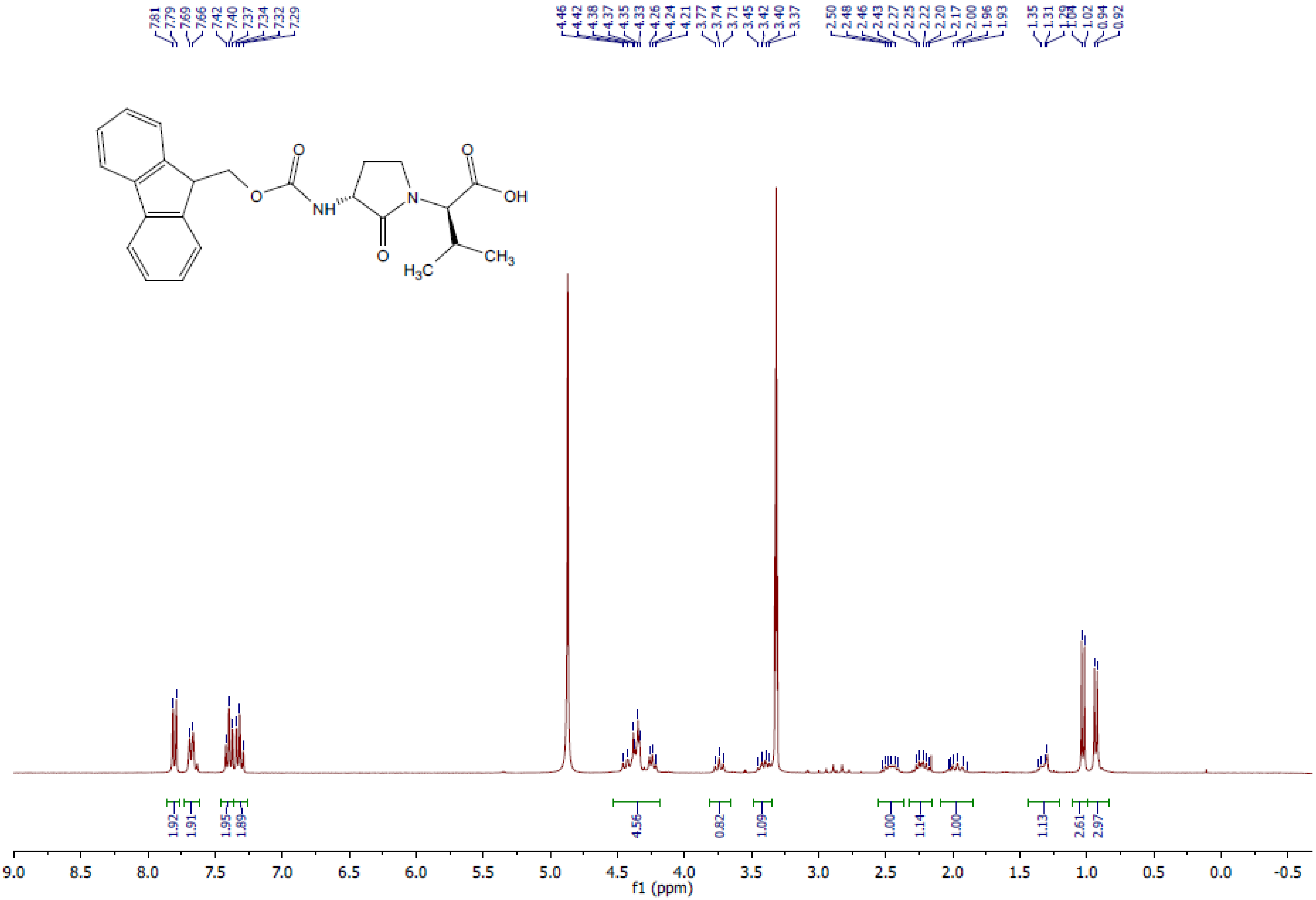

^{13}C NMR (75 MHz, MeOD) ***(R, R)*-7**

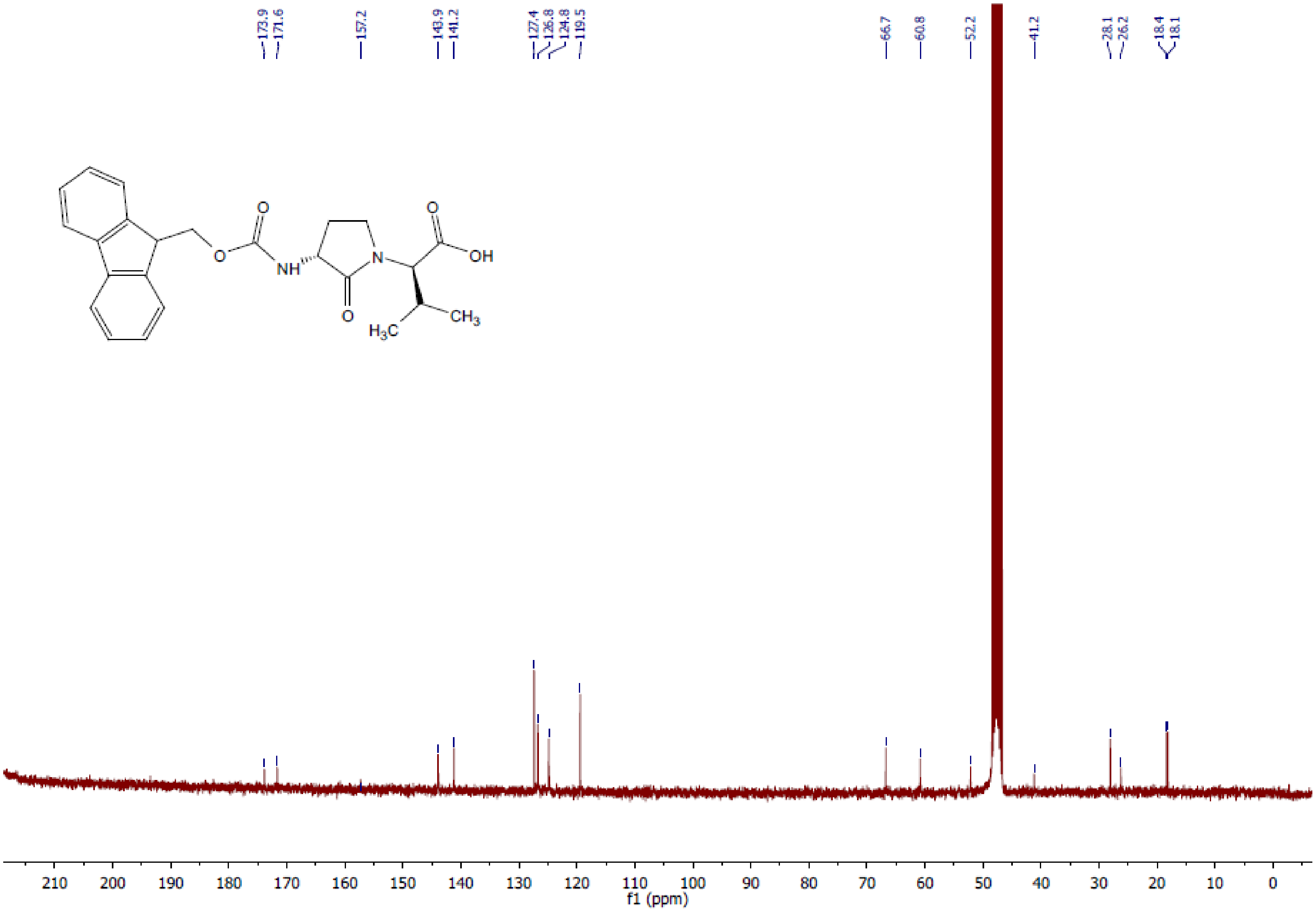

^{1}H NMR (300 MHz, MeOD) ***(R, S)*-7**

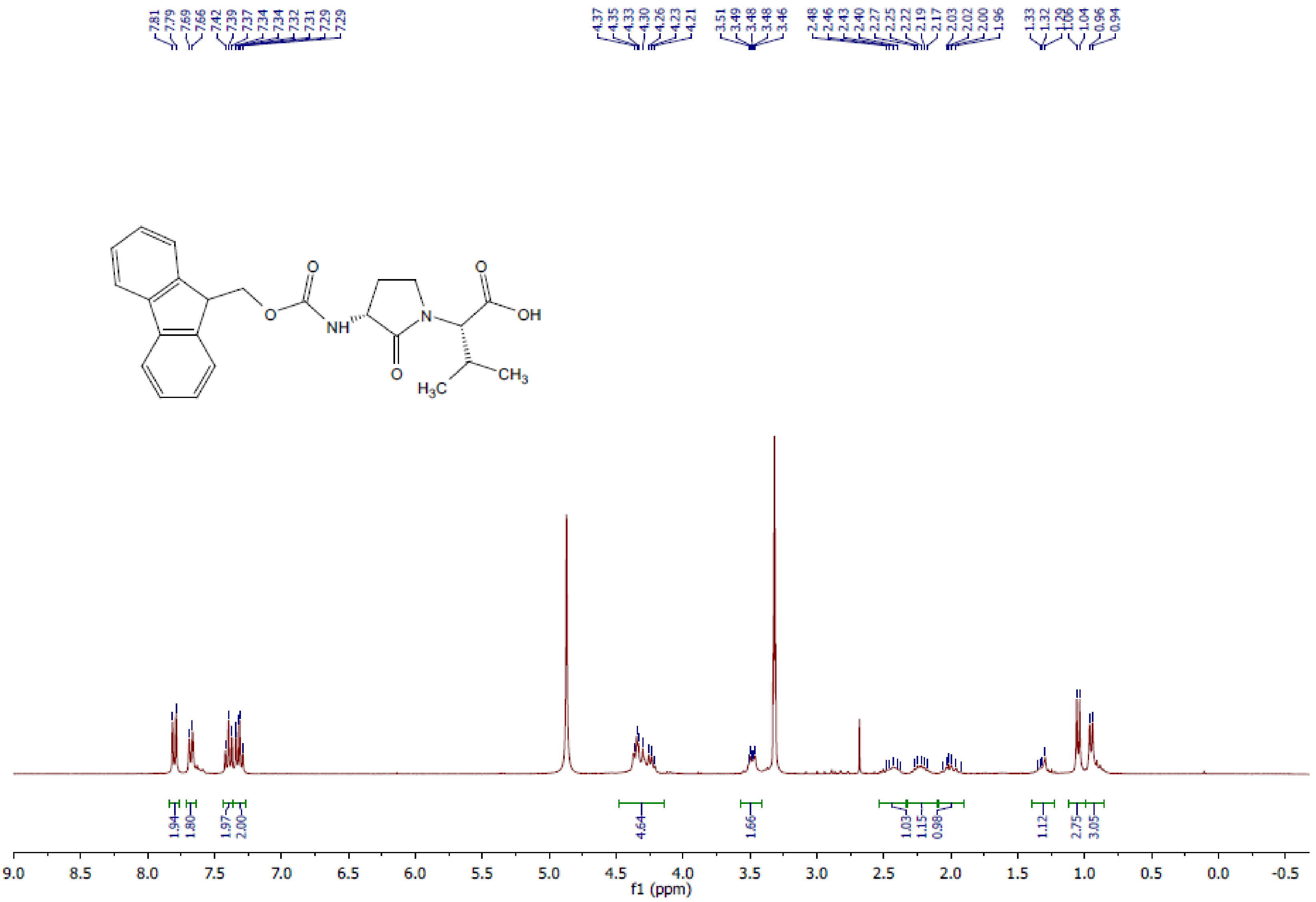

^{13}C NMR (75 MHz, MeOD) ***(R, S)*-7**

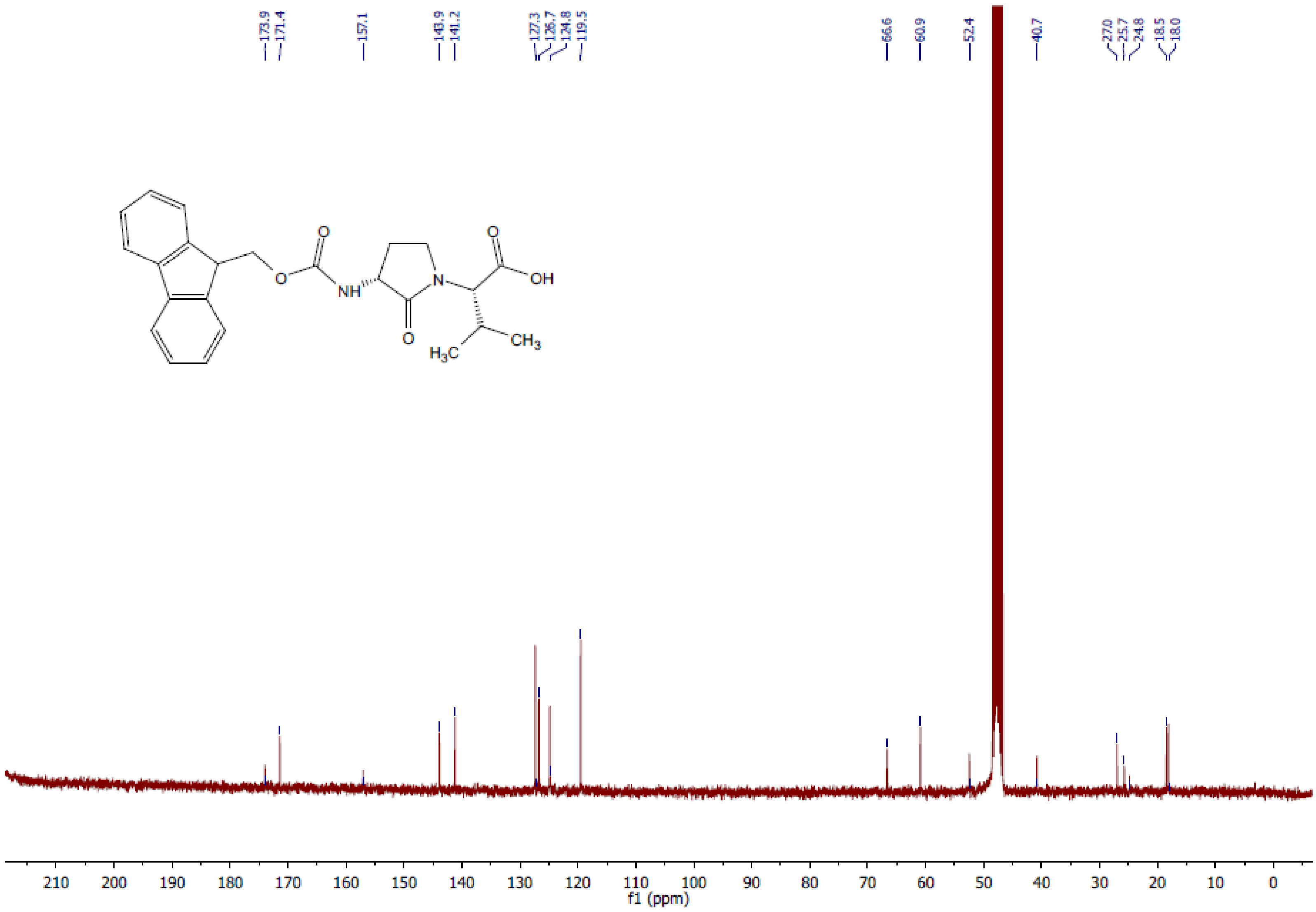

^{1}H NMR (300 MHz, $CDCl_3$) **(*R*, *R*)-10**

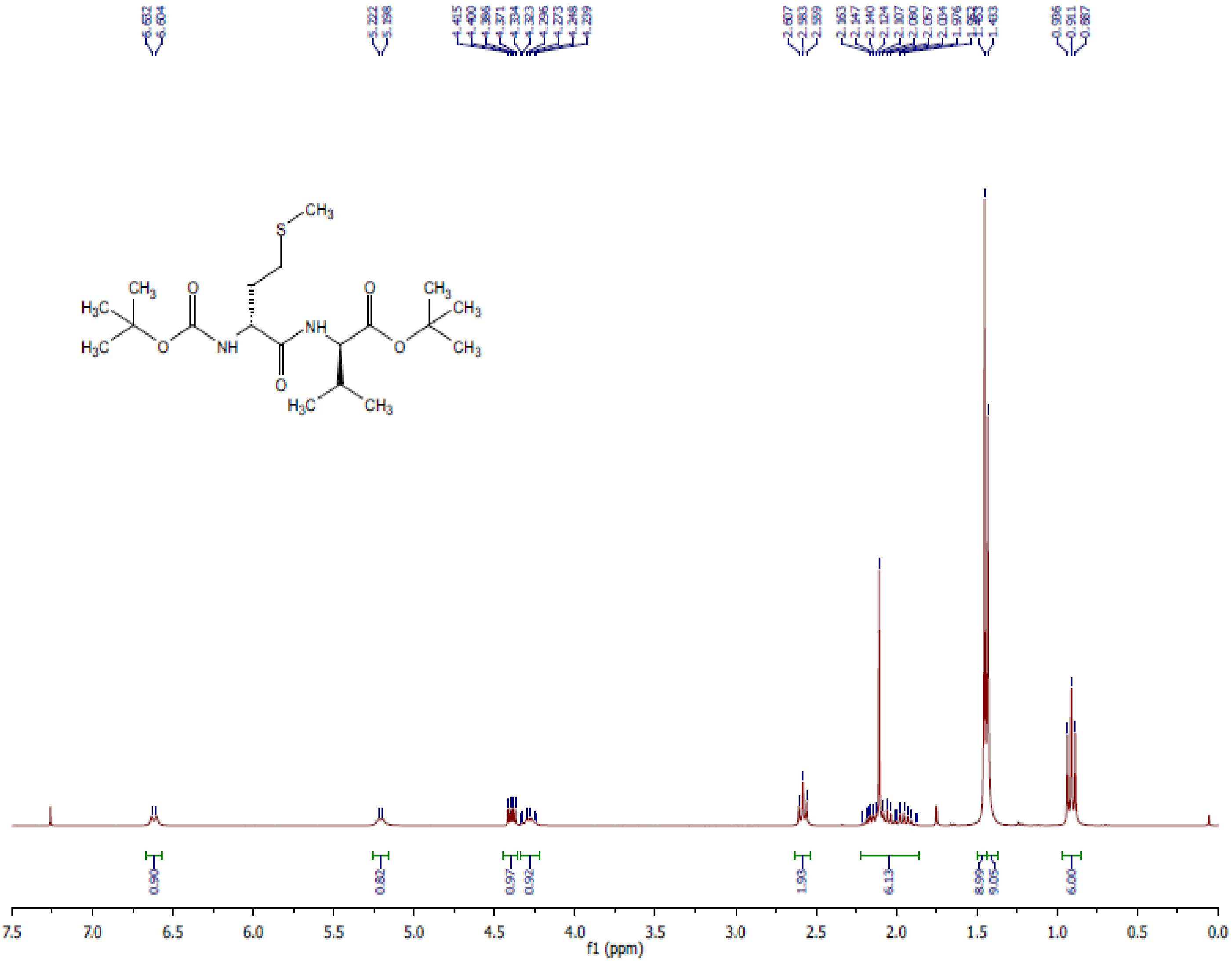

^{13}C NMR (75 MHz, $CDCl_3$) ***(R, R)*-10**

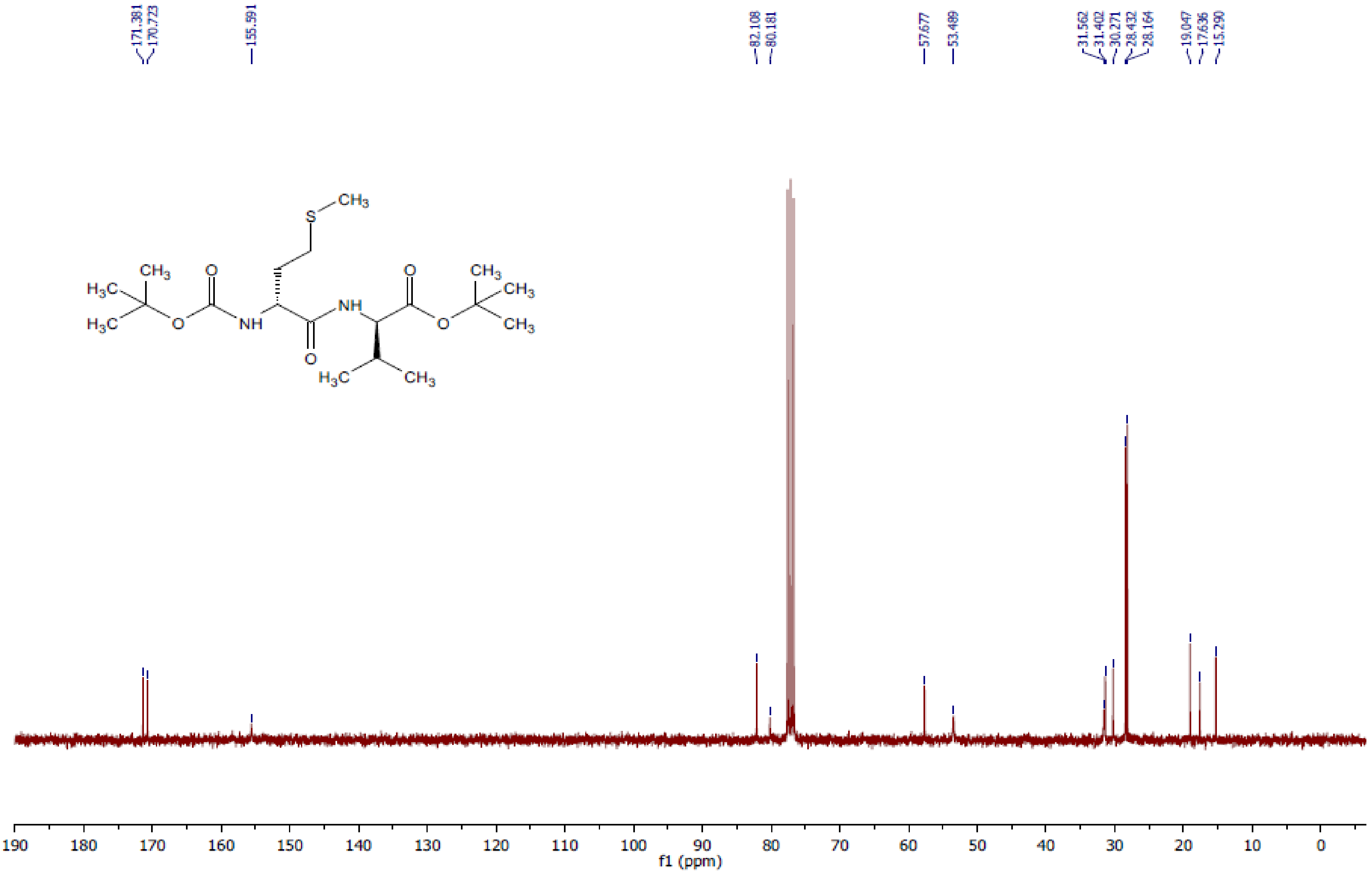

^{1}H NMR (300 MHz, CDCl3) ***(R, S*****)-10**

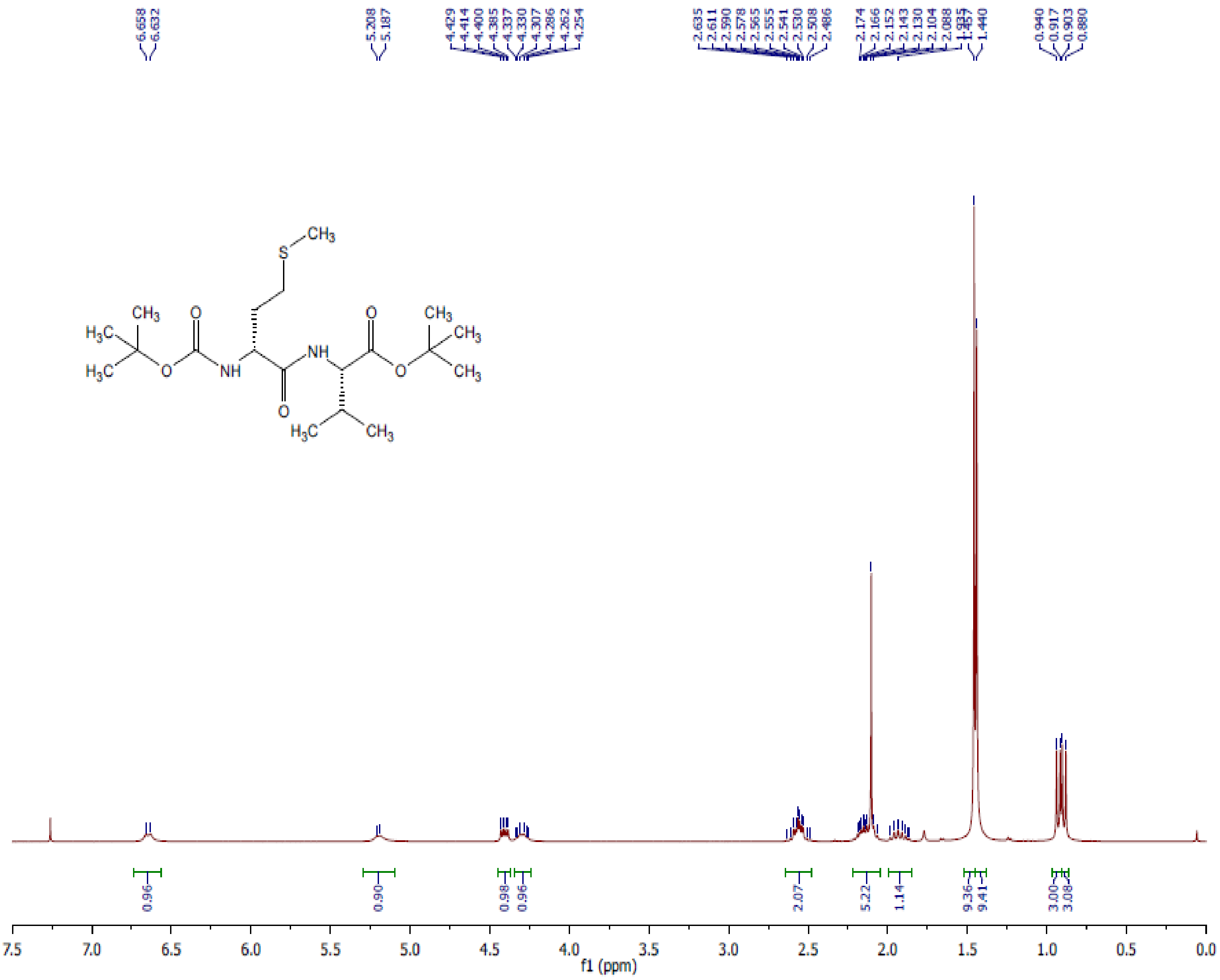

^{13}C NMR (75 MHz, $CDCl_3$) **(*R*, *S*)-10**

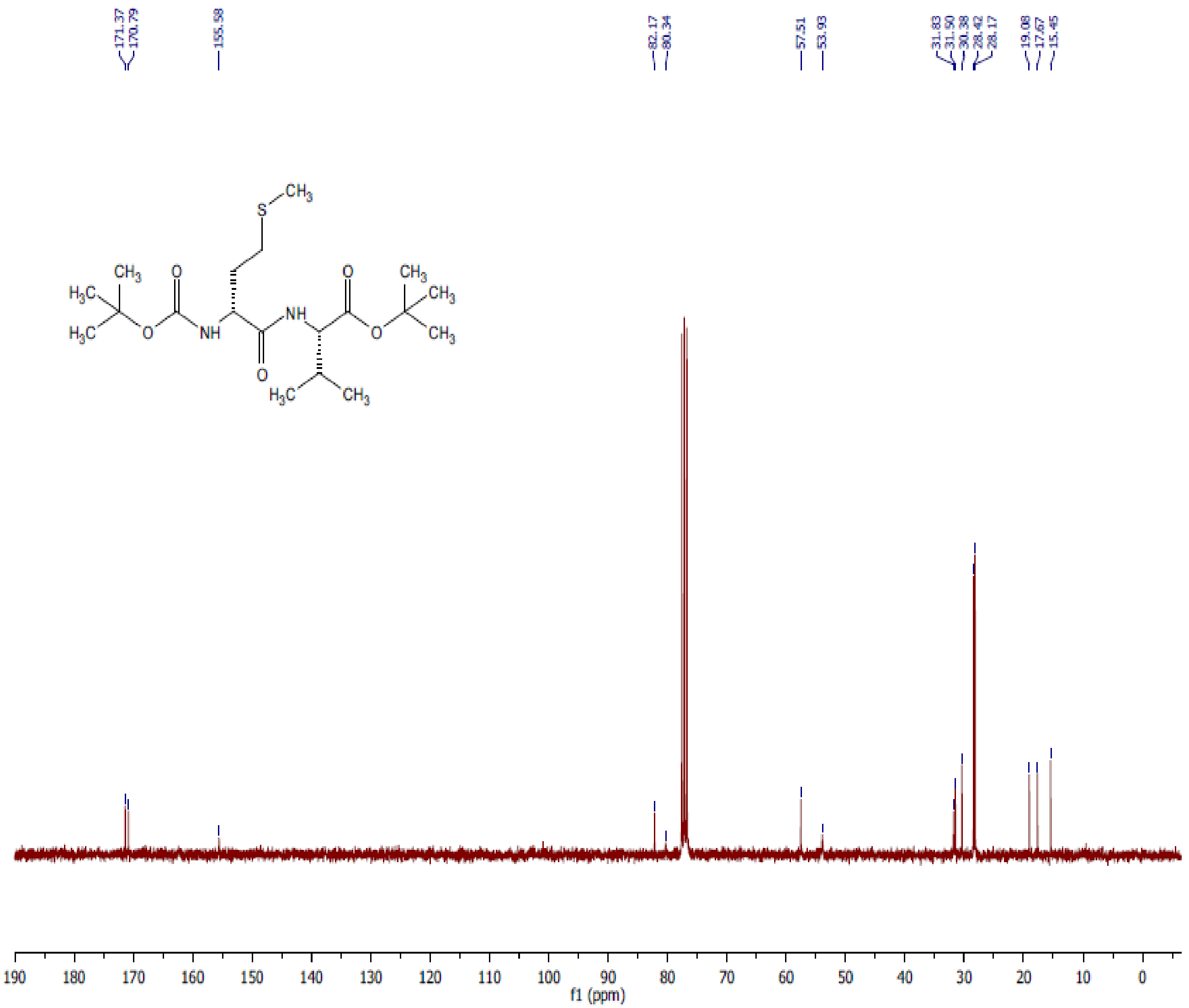

^{1}H NMR (300 MHz, $CDCl_3$) **(*R*, *R*)-11**

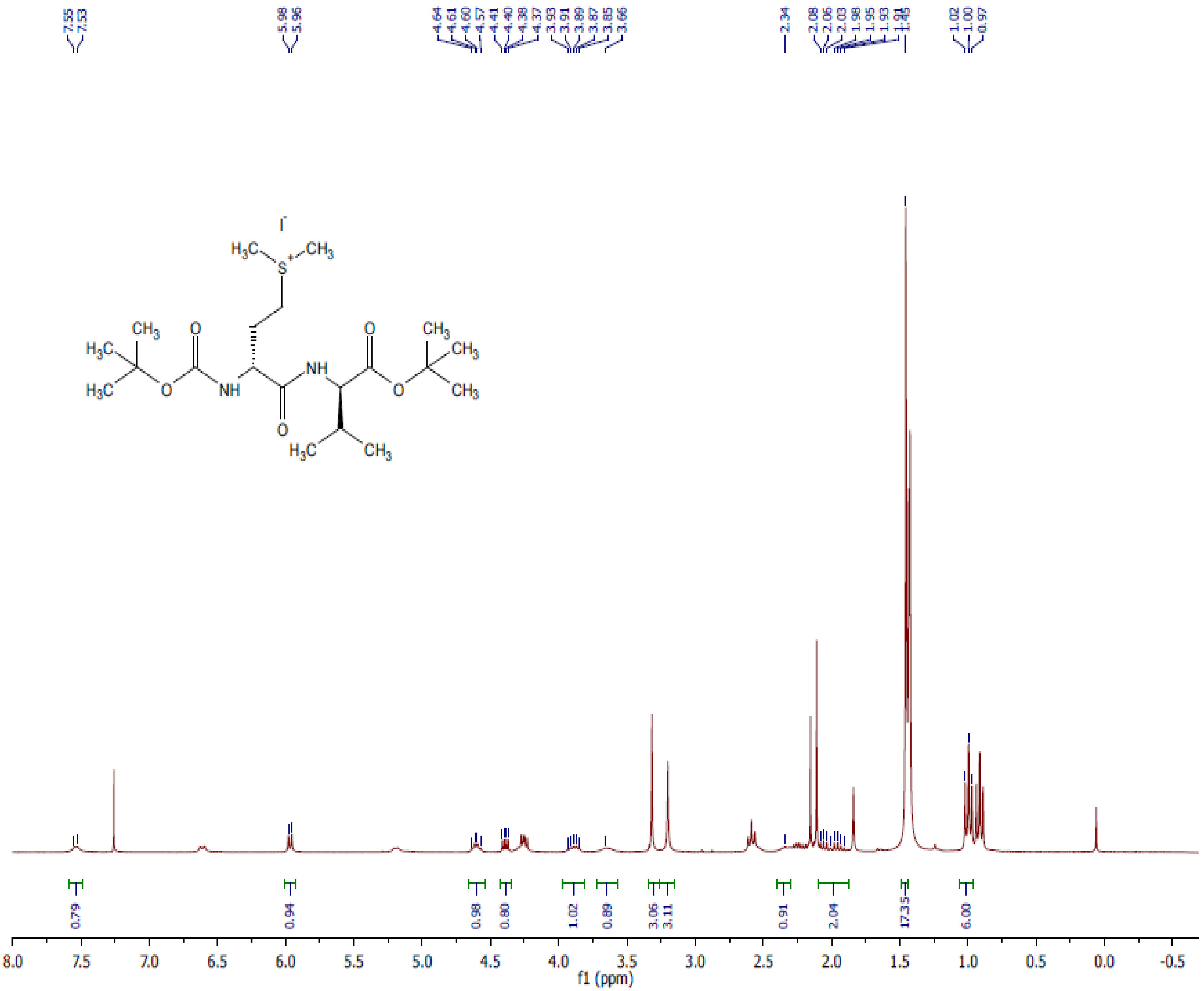

b

^{1}H NMR (300 MHz, $CDCl_3$) ***(R, S)*-11**

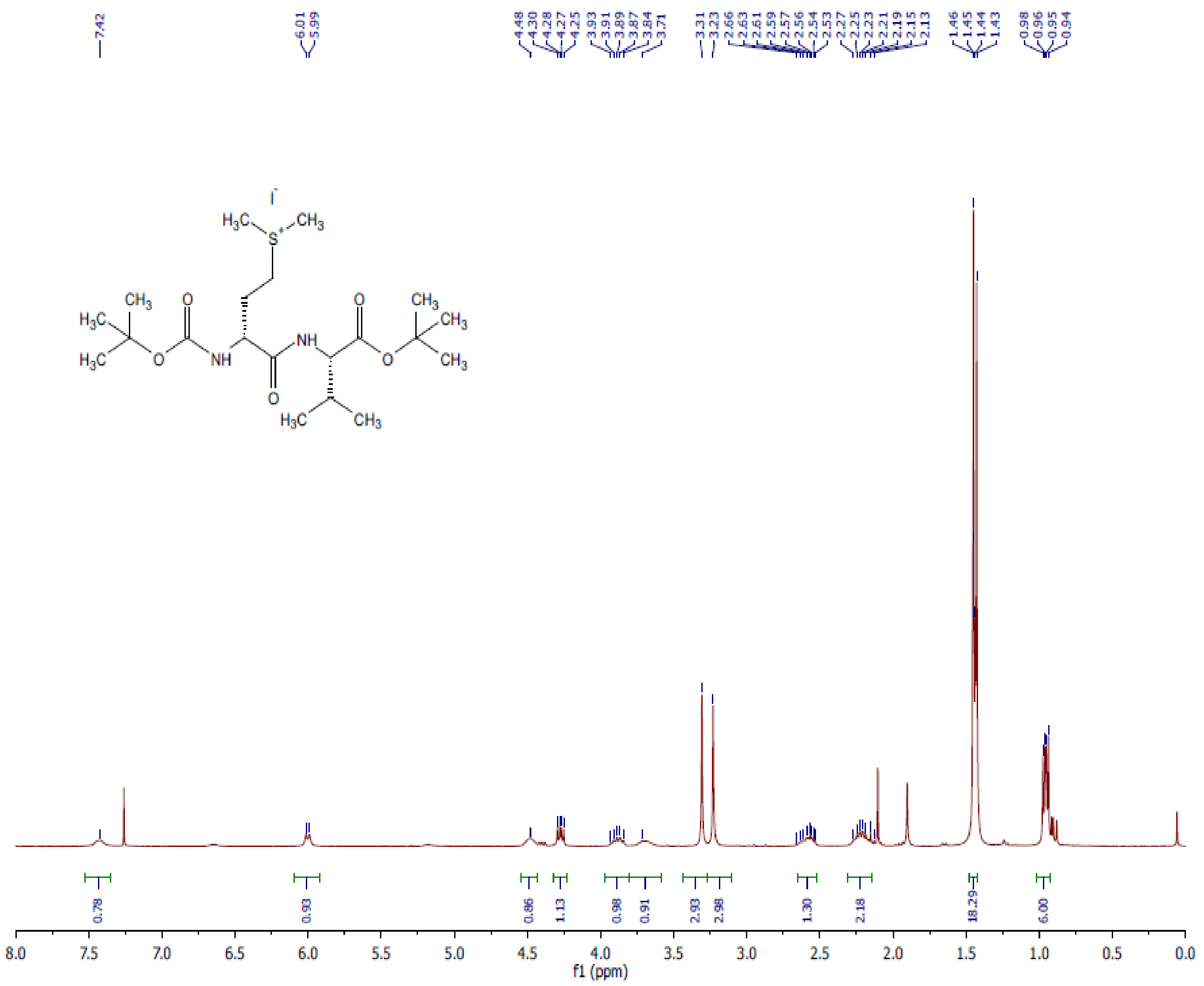

^{1}H NMR (300 MHzMeOD) **[(3*R*, 4*R*, 2'*S*)-12]**

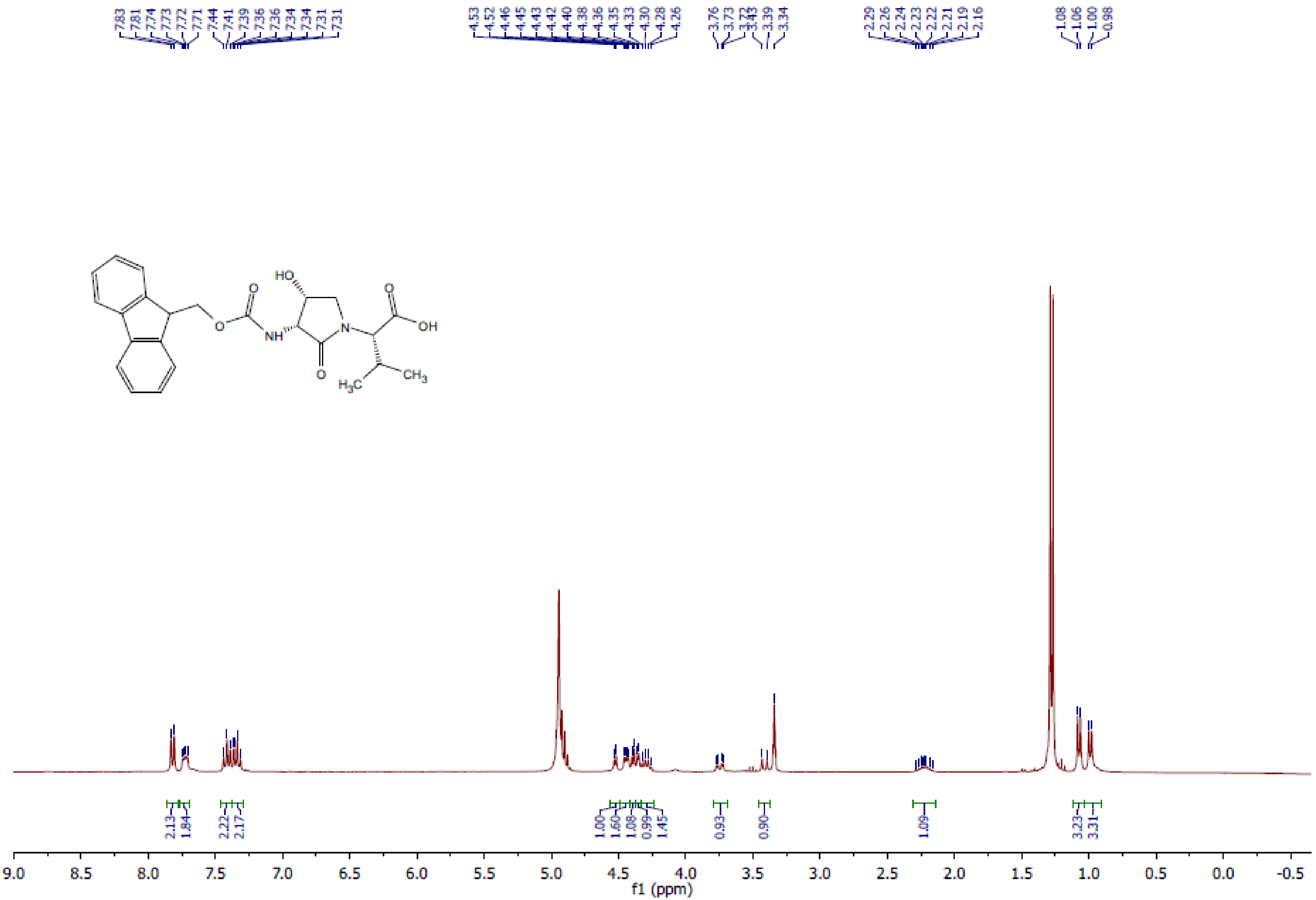

^{13}C NMR (75 MHz, MeOD) **[(3*R*, 4*R*, 2'*S*)-12]**

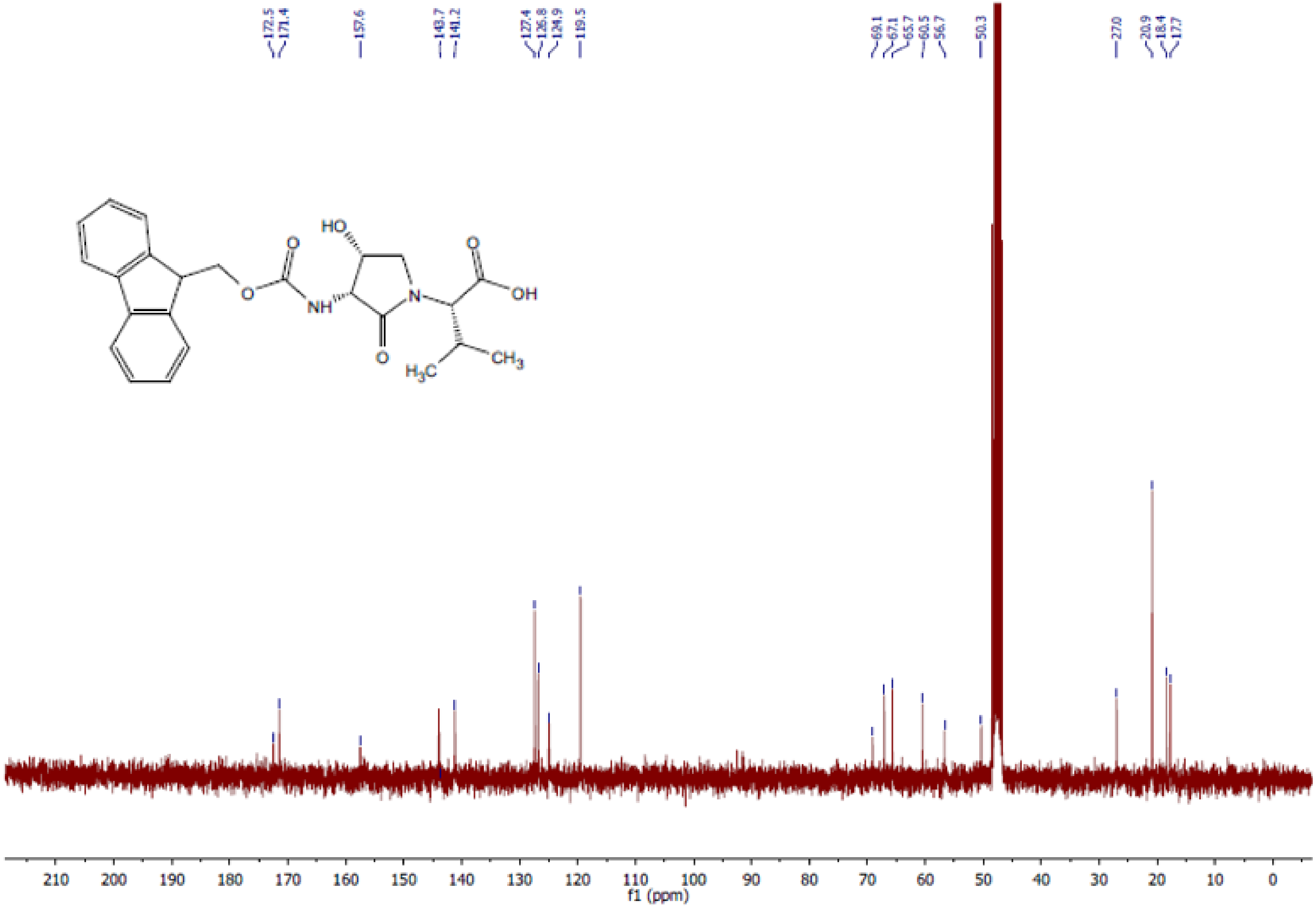

^{1}H NMR (300 MHz, $CDCl_3$) ***(R, R)*-13**

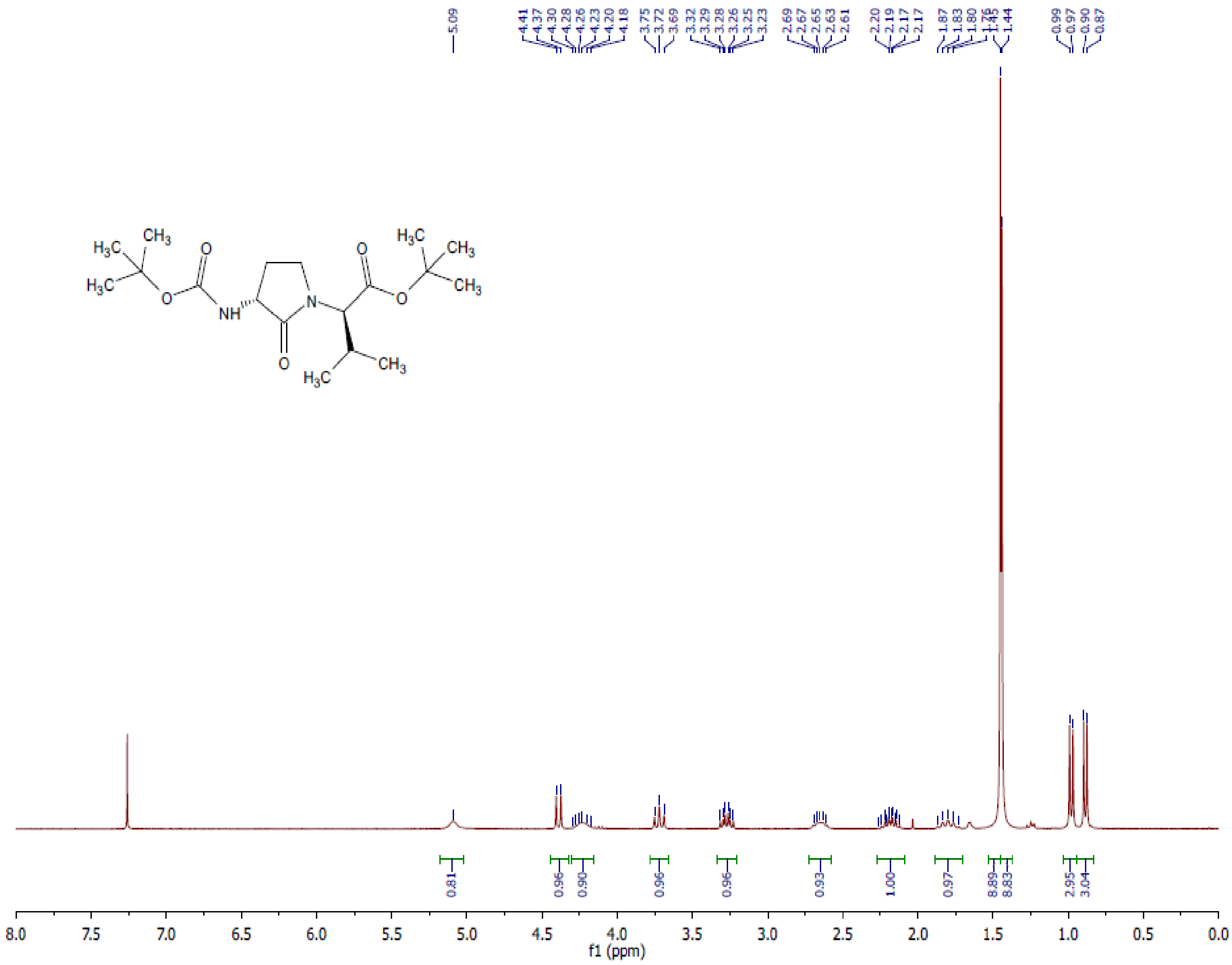

^{13}C NMR (75 MHz, $CDCl_3$) **(*R*, *R*)-13**

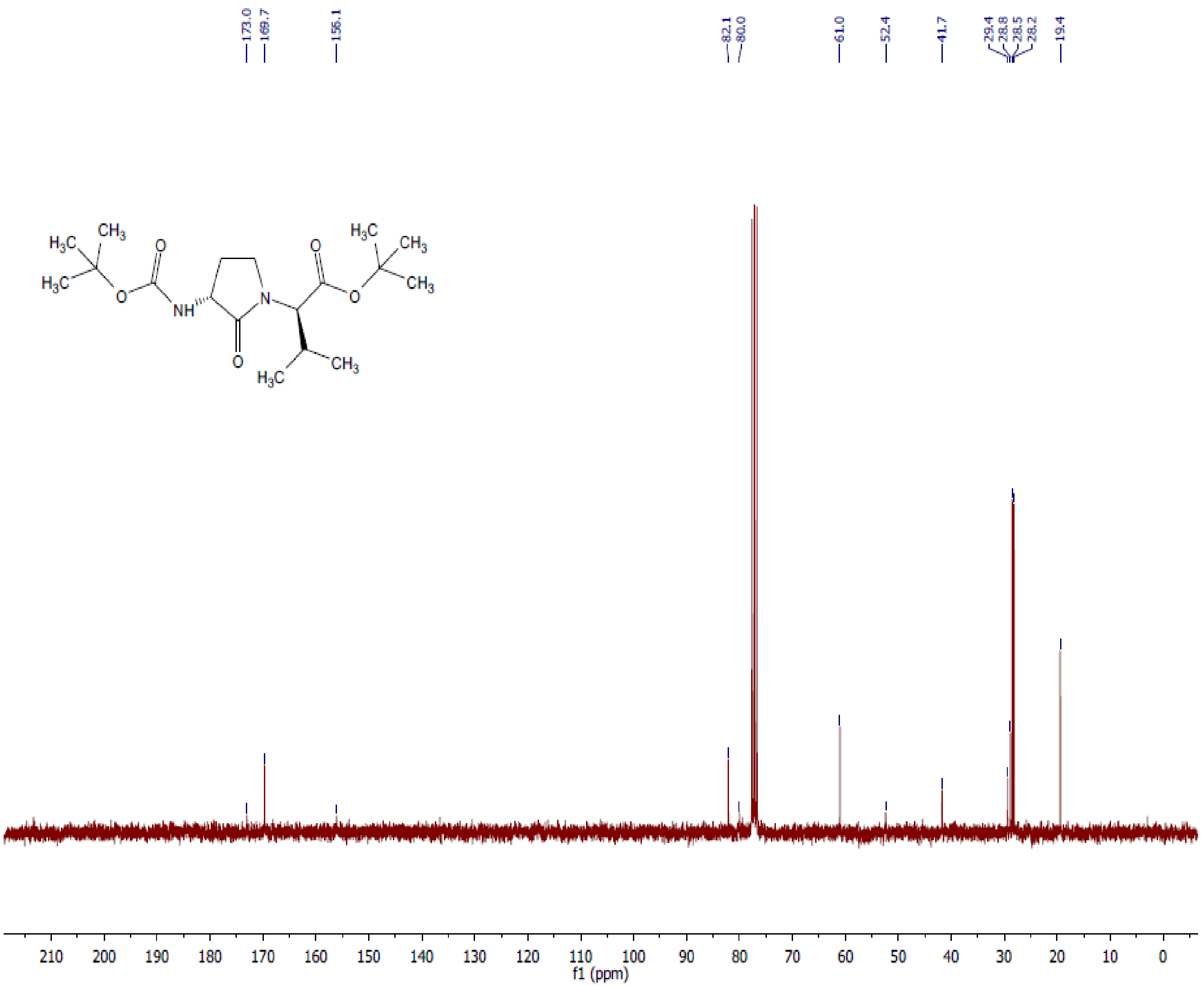

^{1}H NMR (300 MHz, $CDCl_3$) ***(R, S)*-13**

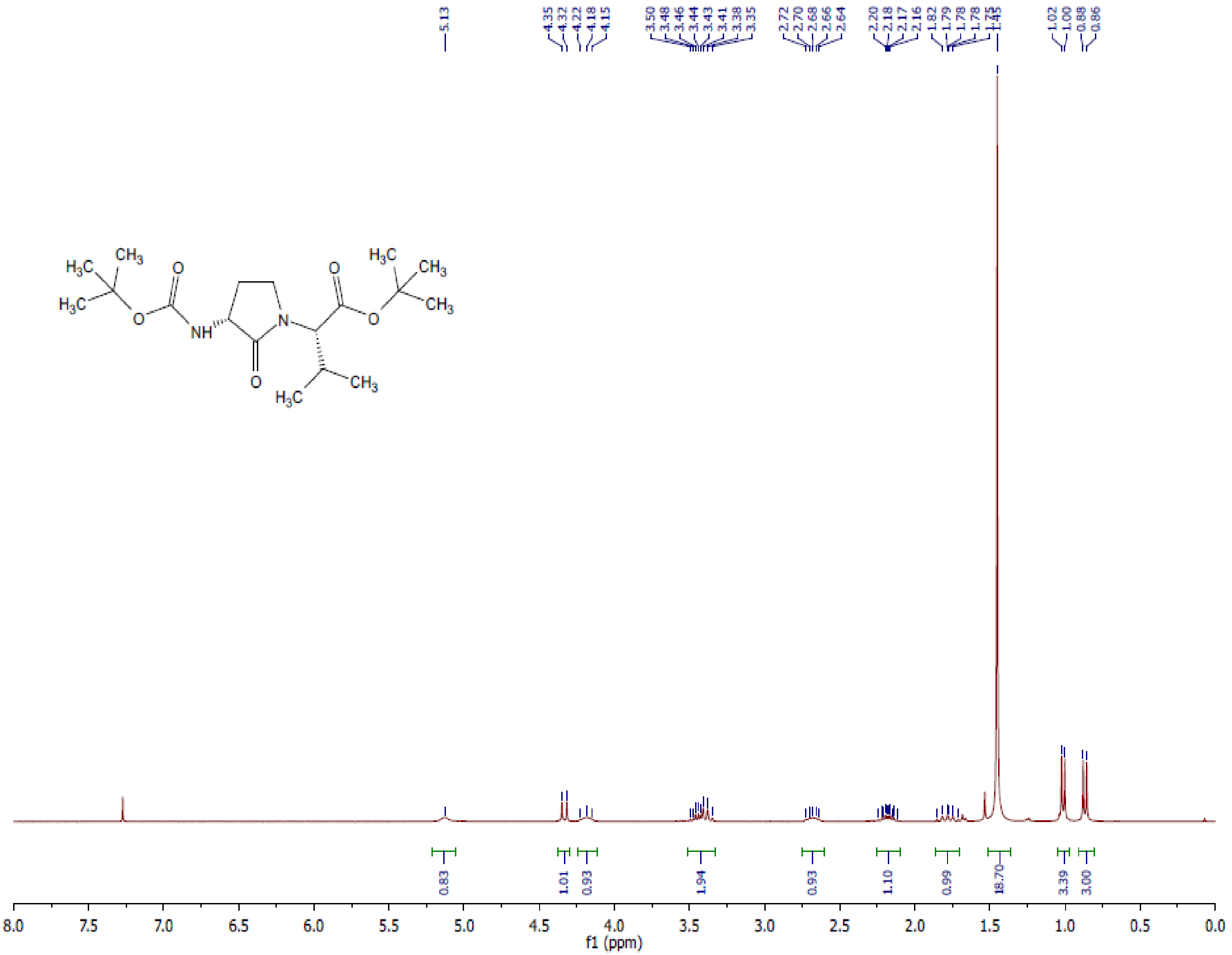

^{13}C NMR (75 MHz, $CDCl_3$) ***(R, S)*-13**

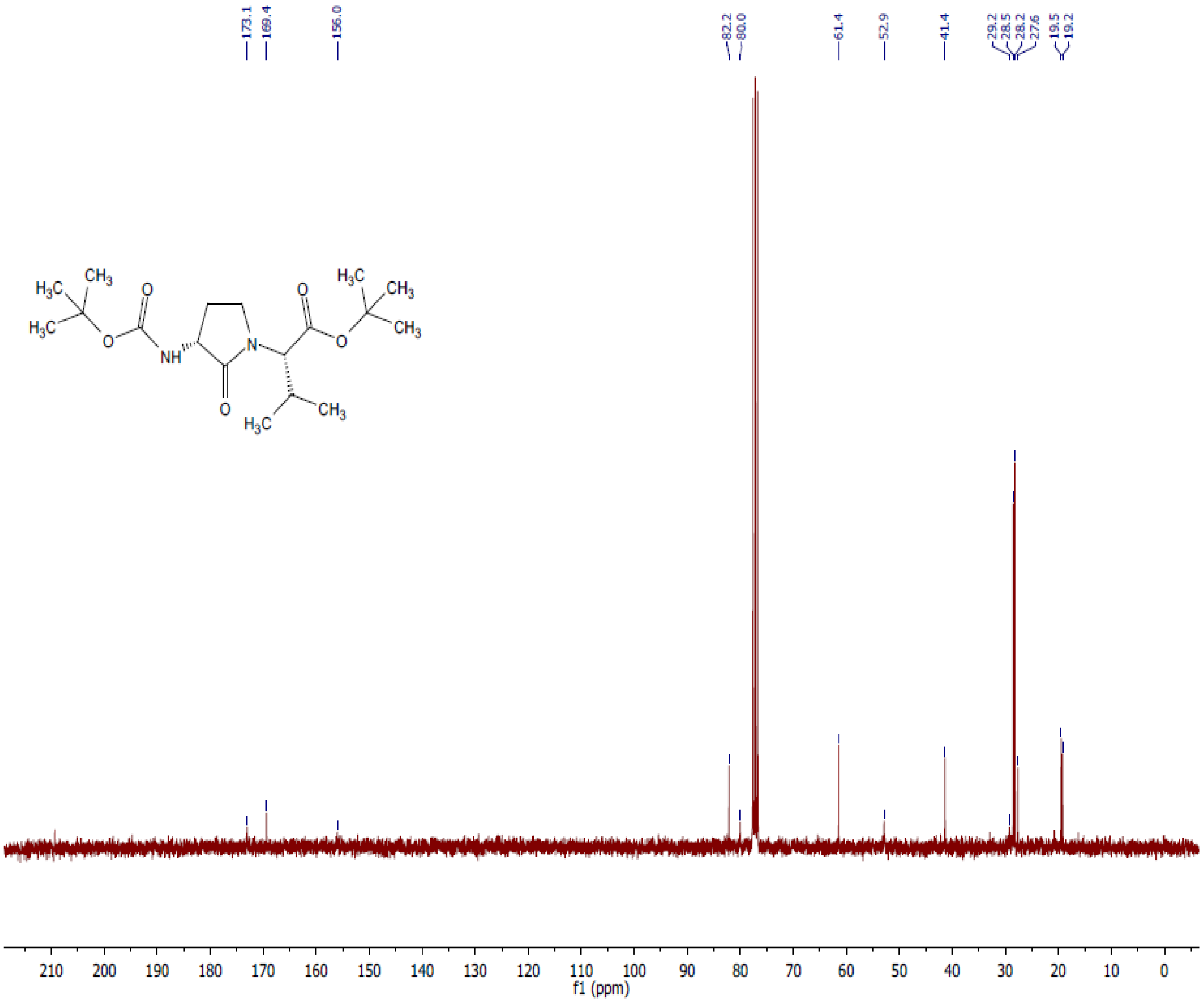

^{1}H NMR (300 MHz, MeOD) **(*R*, *R*)-14**

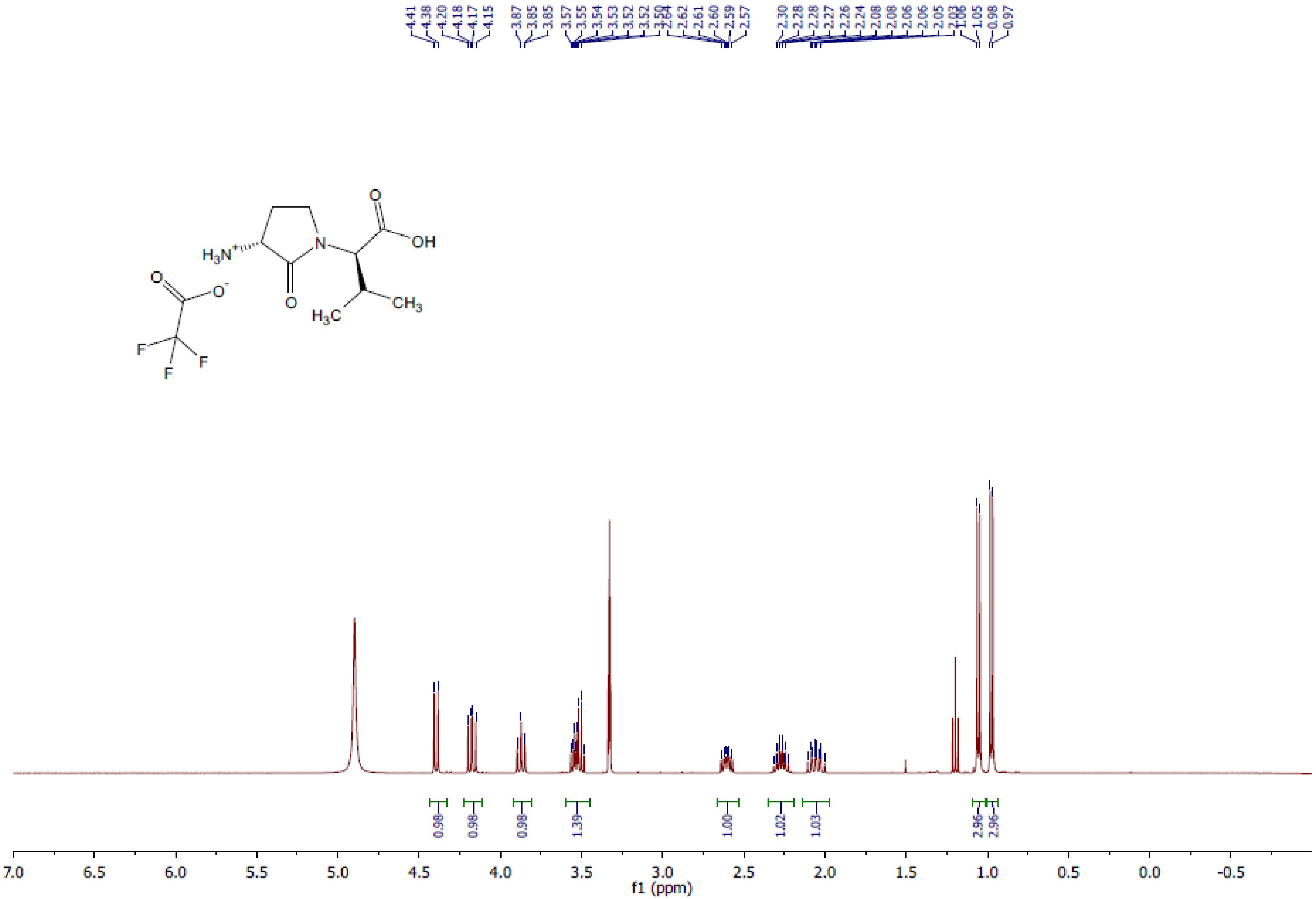

^{13}C NMR (75 MHz, MeOD) **(*R*, *R*)-14**

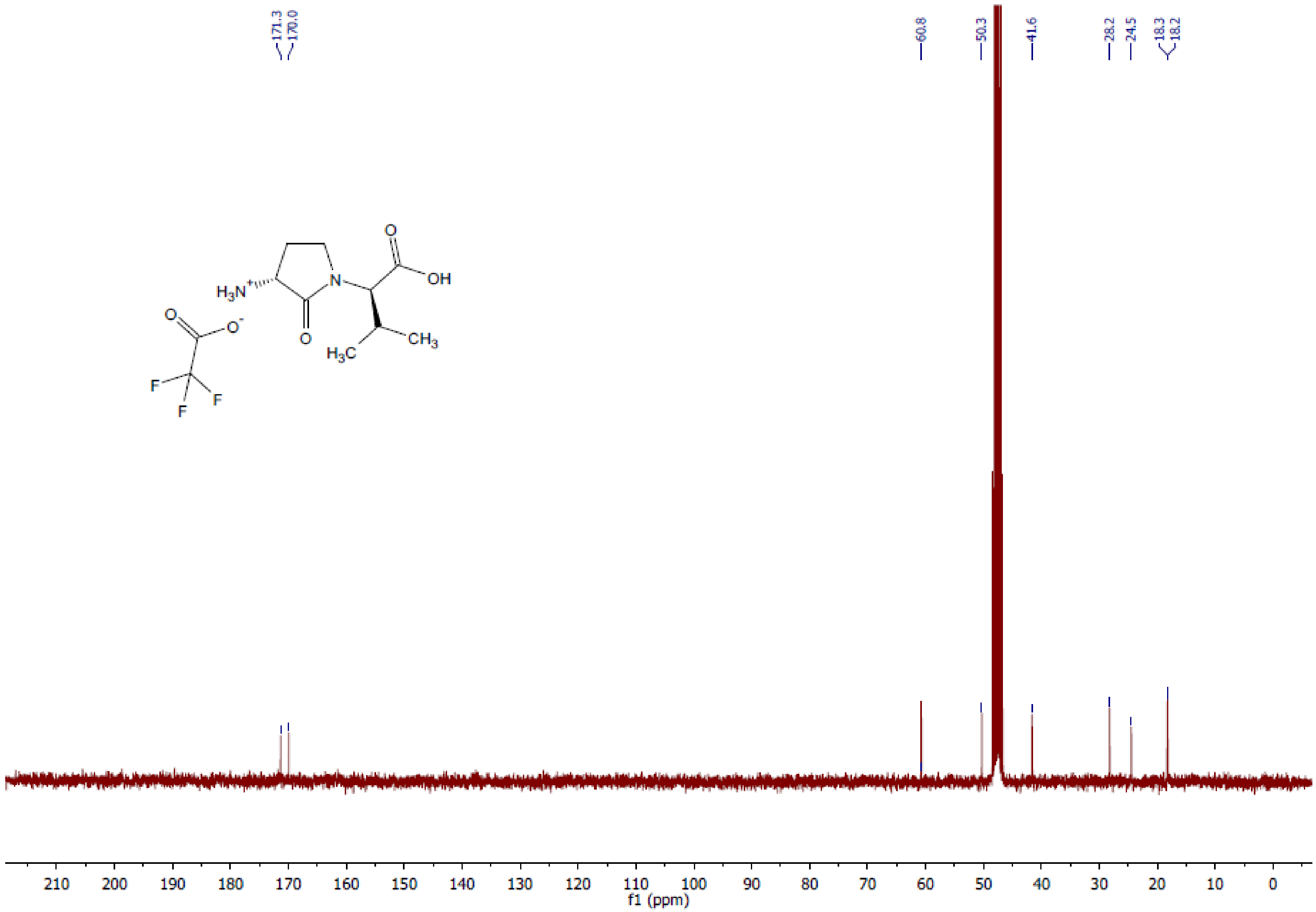

^{1}H NMR (300 MHz, MeOD) ***(R, S)*-14**

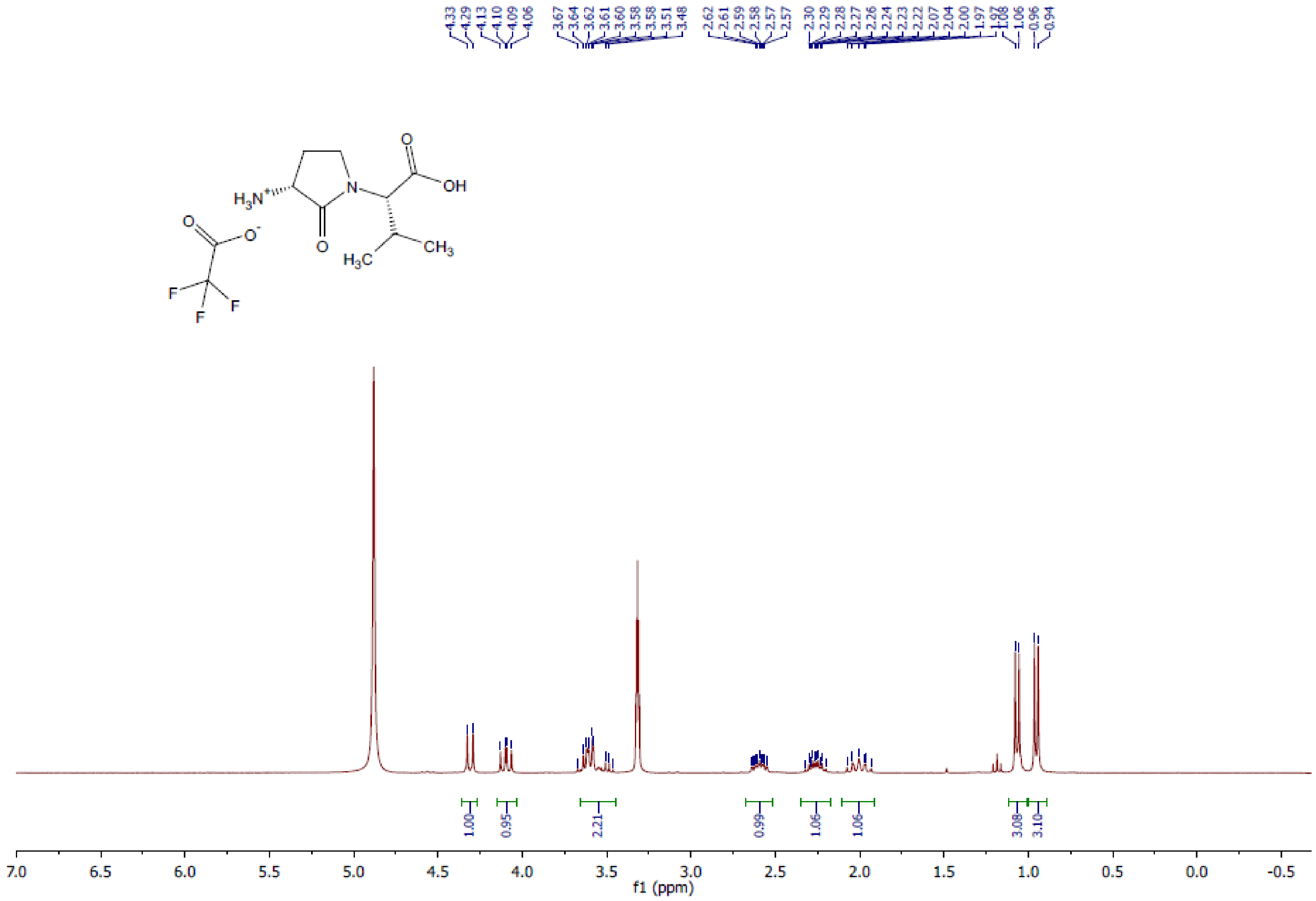

^{13}C NMR (75 MHz, MeOD) **(*R*, *S*)-14**

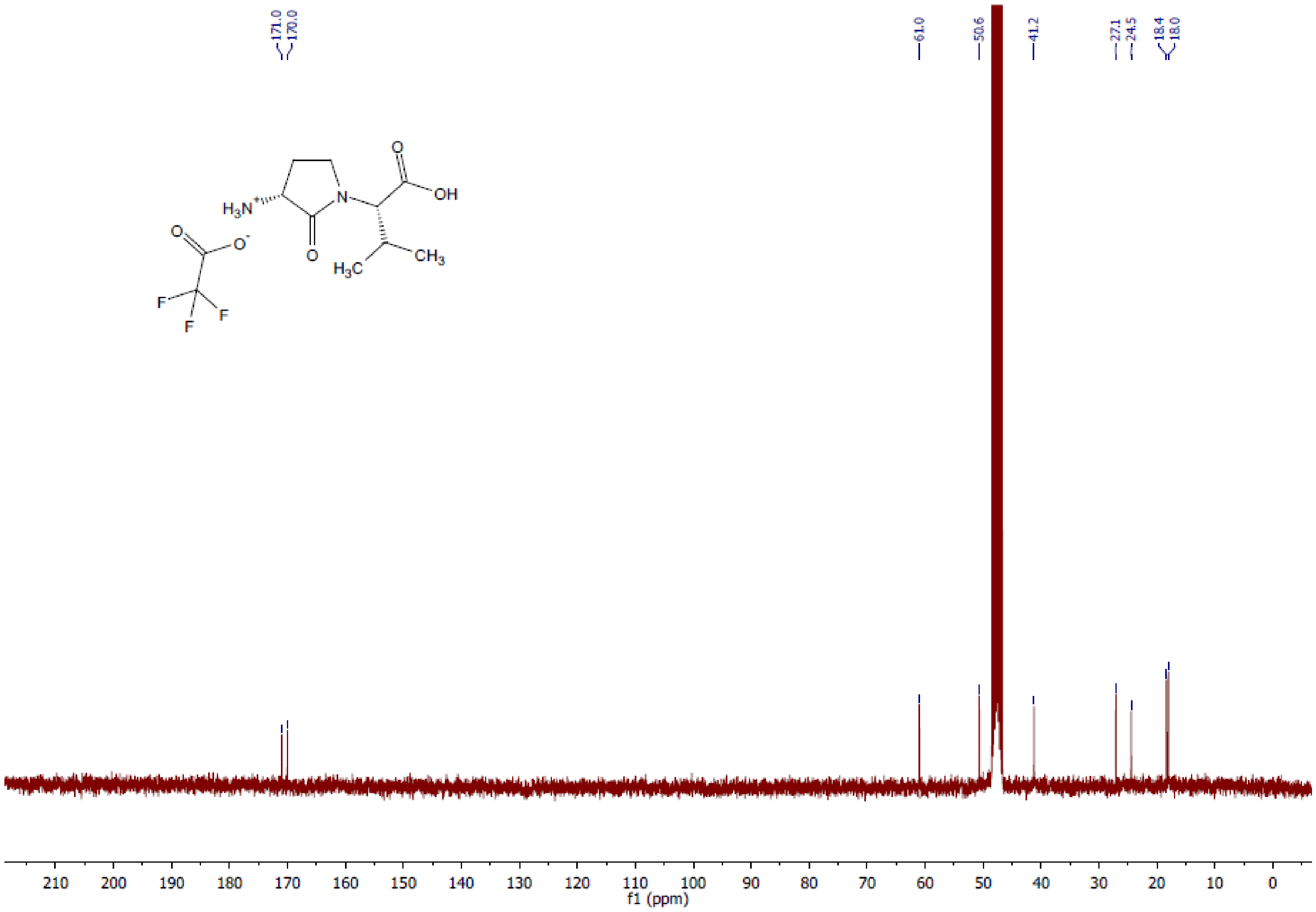

^{1}H NMR (300 MHz, $CDCl_3$) **(*R*)-15**

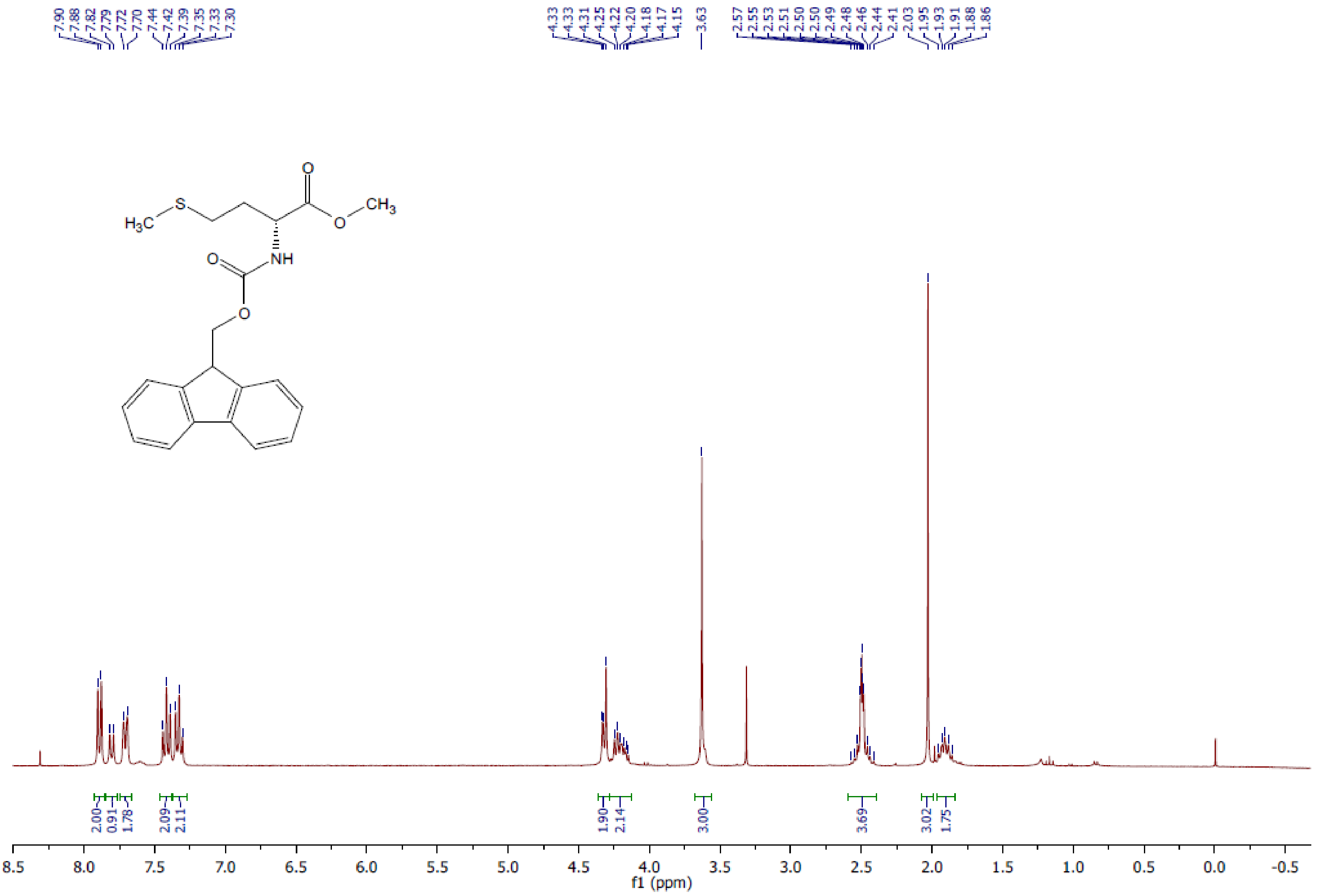

^{13}C NMR (75 MHz, $CDCl_3$) **(*R*)-15**

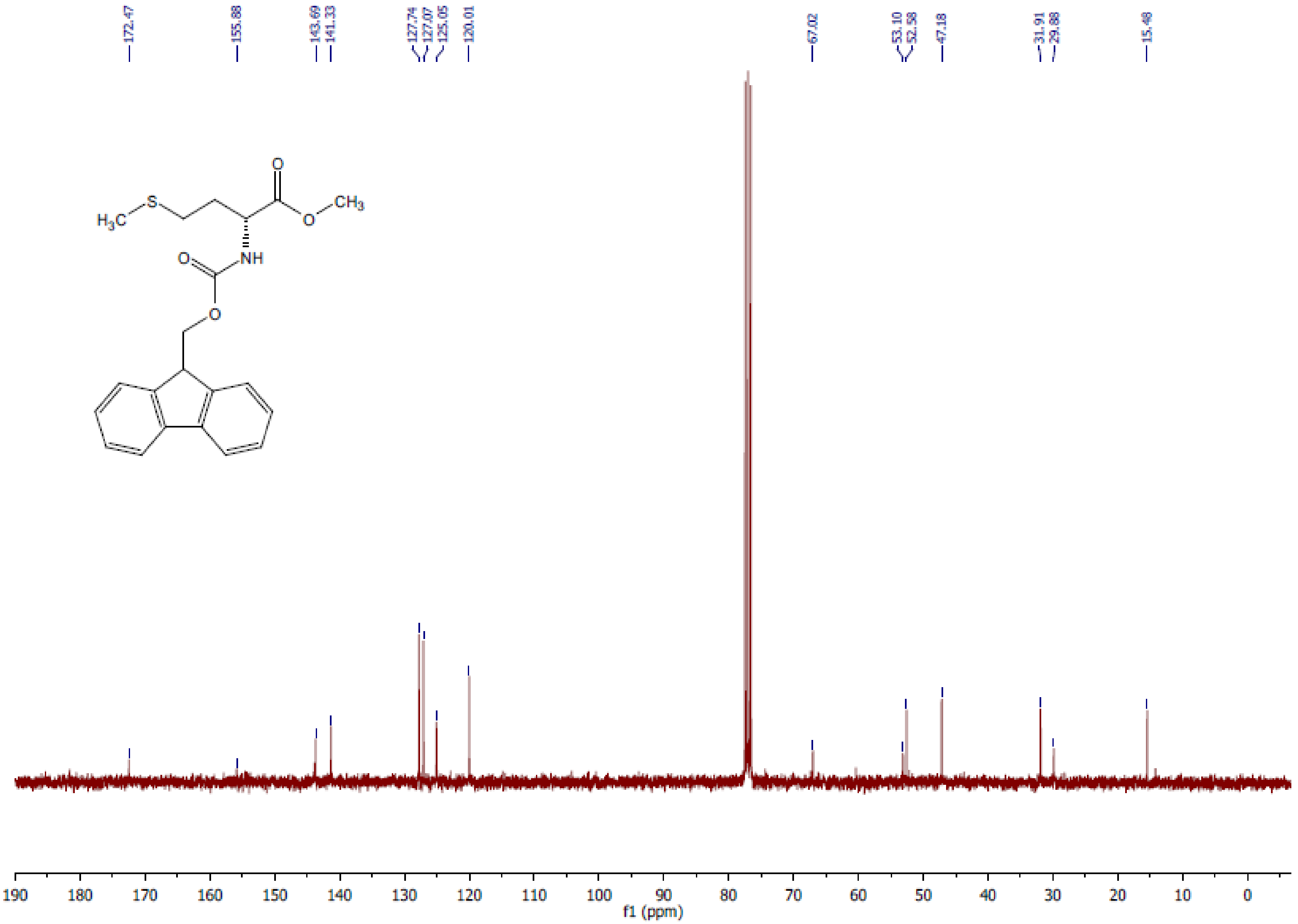

^{1}H NMR (300 MHz, $CDCl_3$) **(*R*)-16**

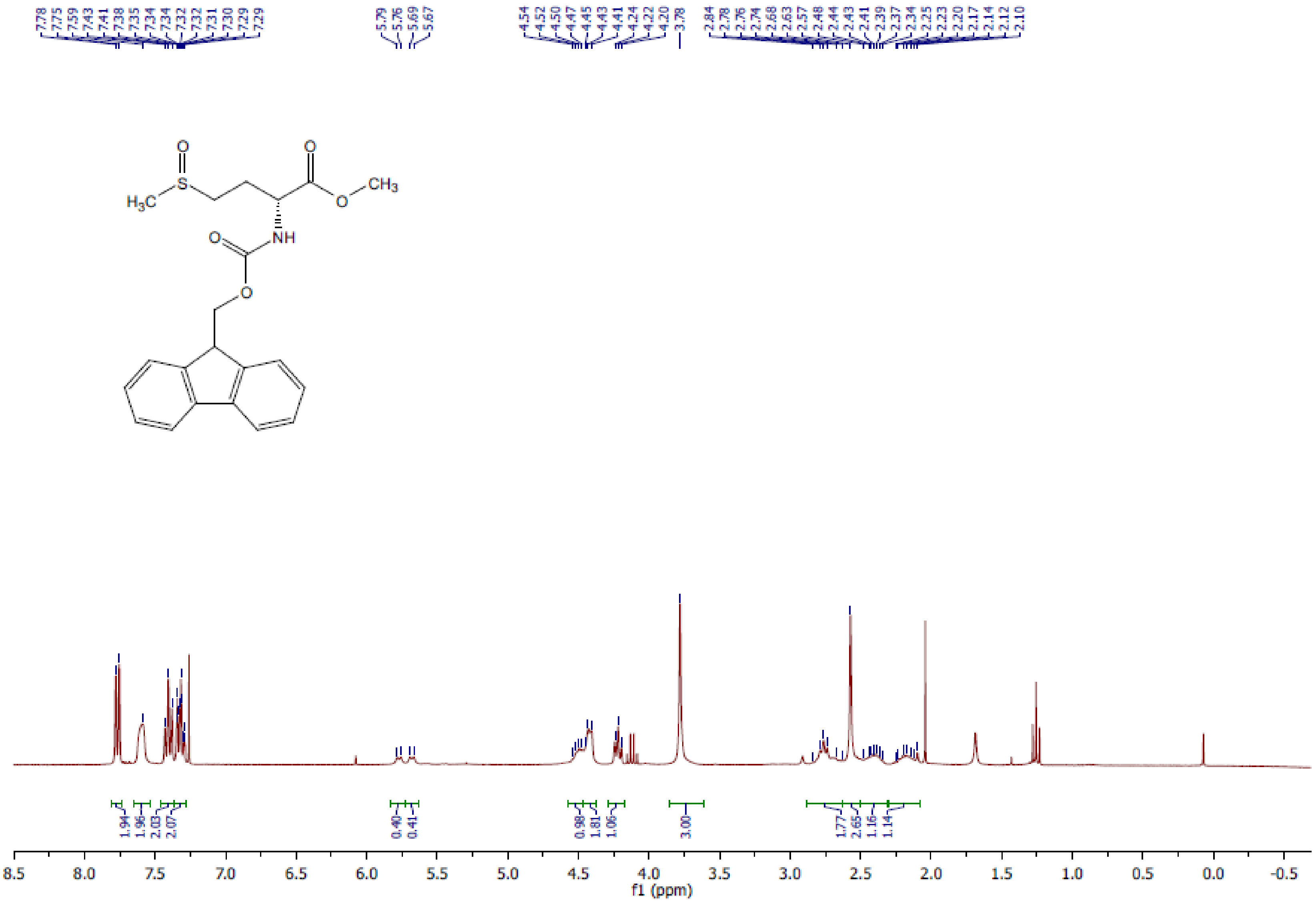

^{13}C NMR (75 MHz, $CDCl_3$) **(*R*)-16**

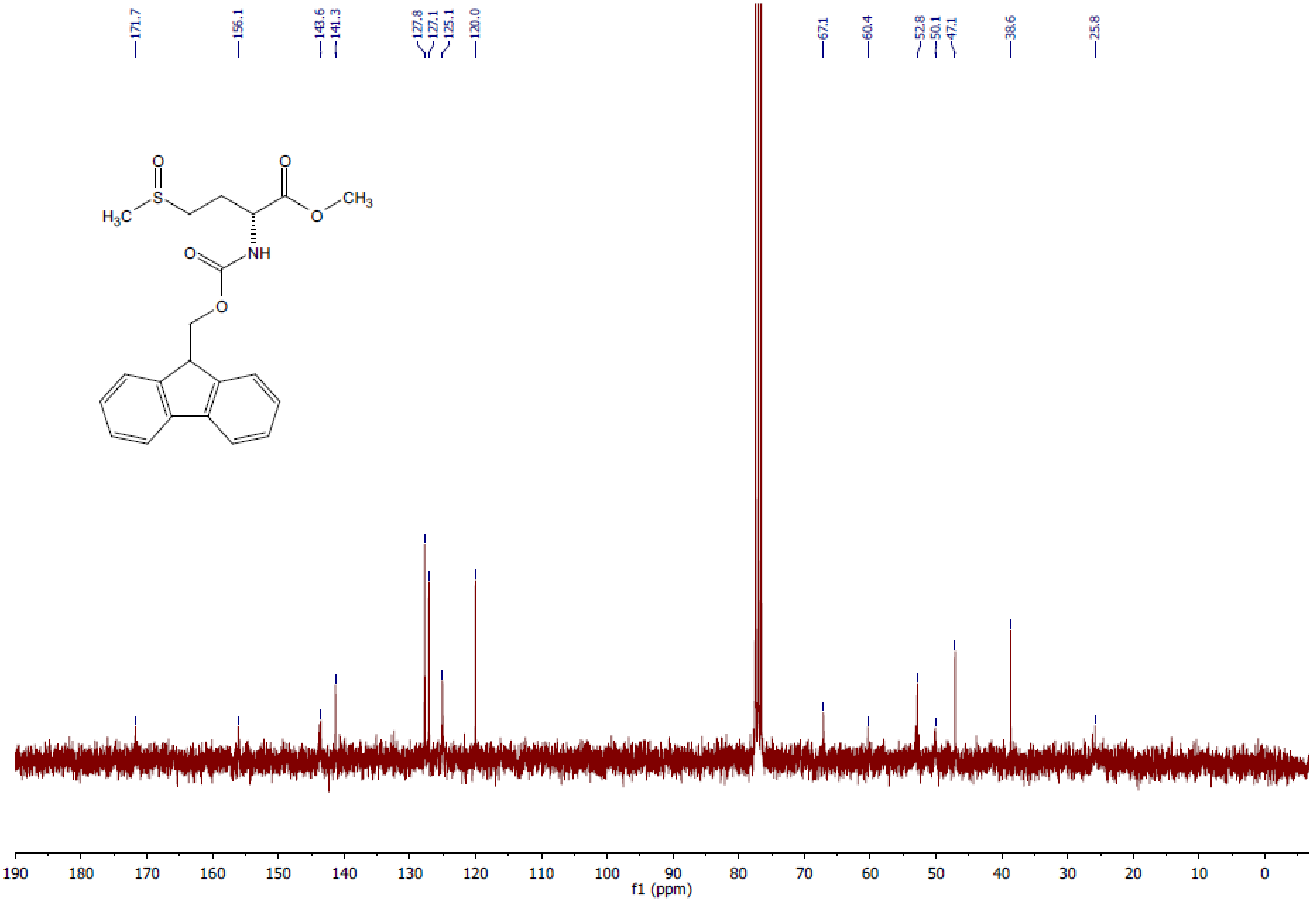

^{1}H NMR (300 MHz, $CDCl_3$) ***(R)*-17**

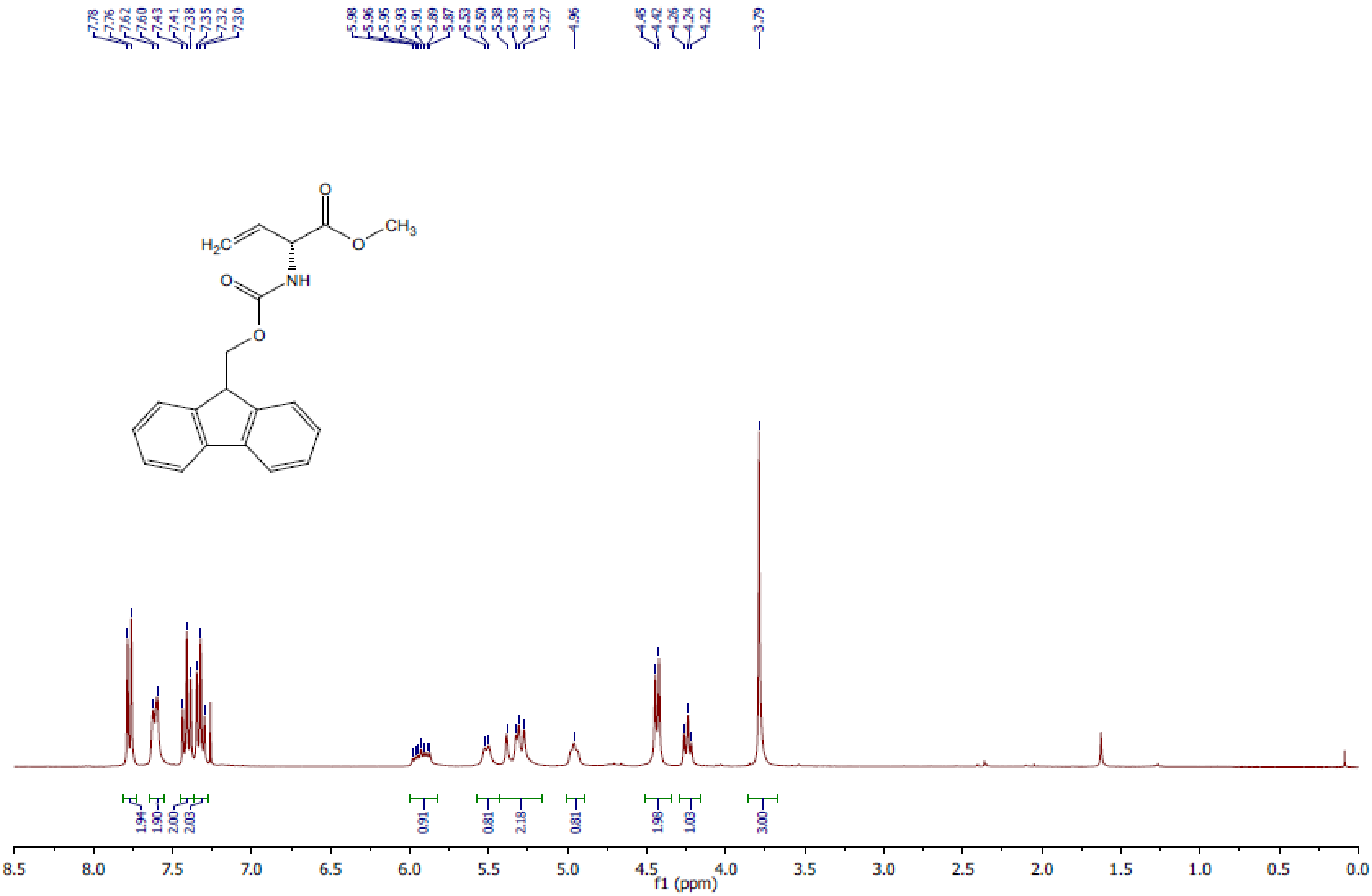

^{13}C NMR (75 MHz, $CDCl_3$) **(*R*)-17**

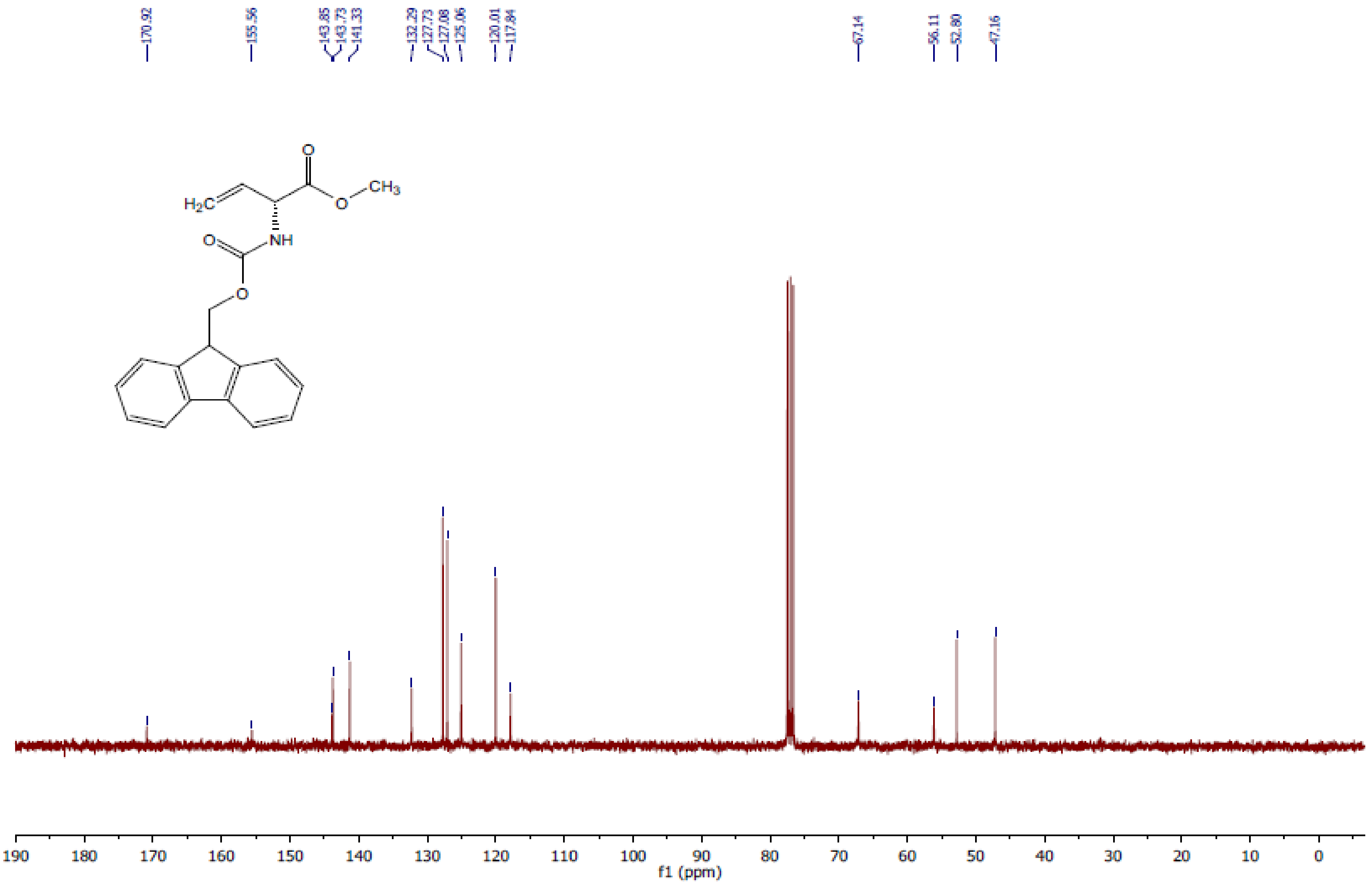

^{1}H NMR (500 MHz, $CDCl_3$) **(*R*, *R*)-18**

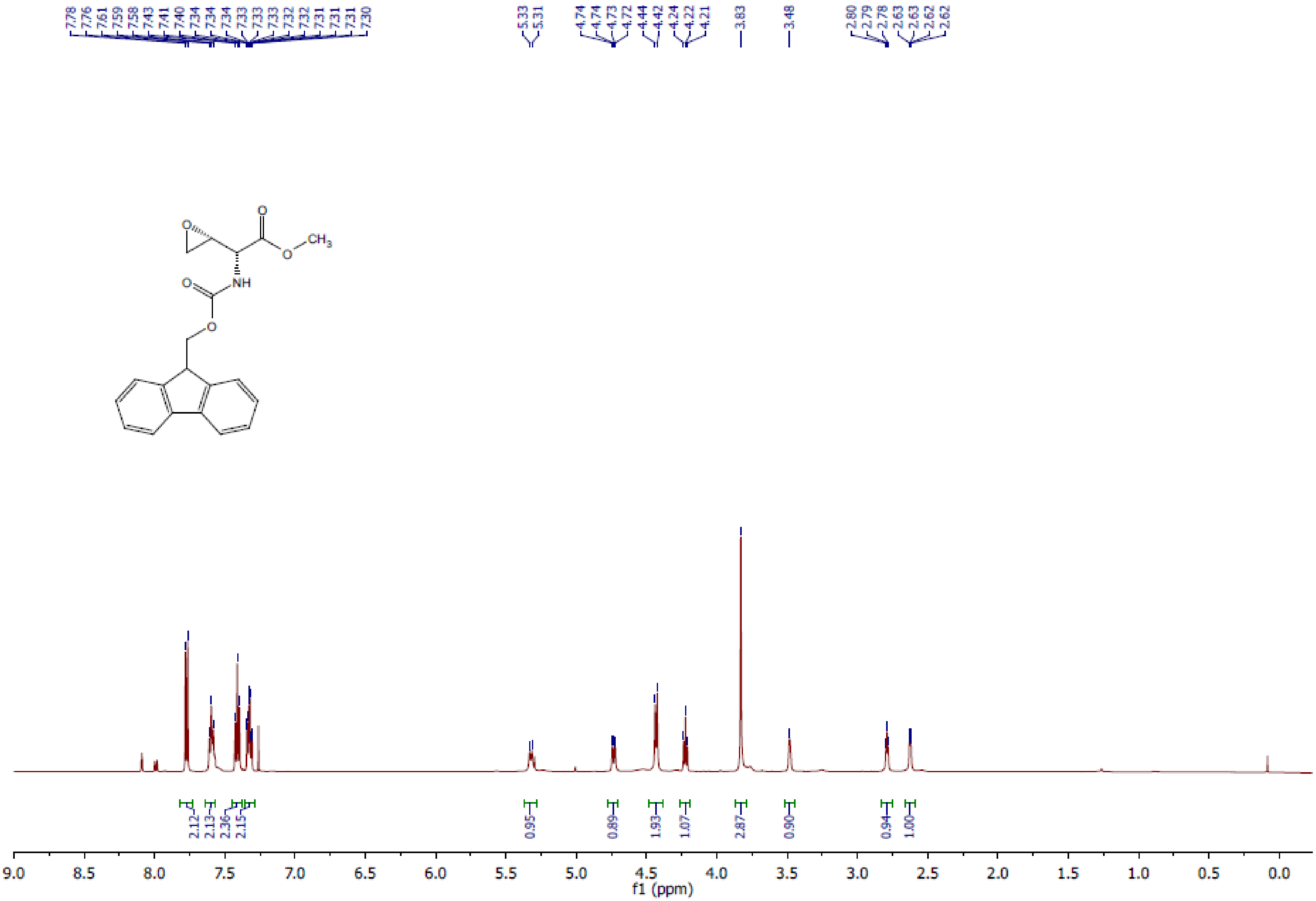

^{13}C NMR (126 MHz, $CDCl_3$) **(*R*, *R*)-18**

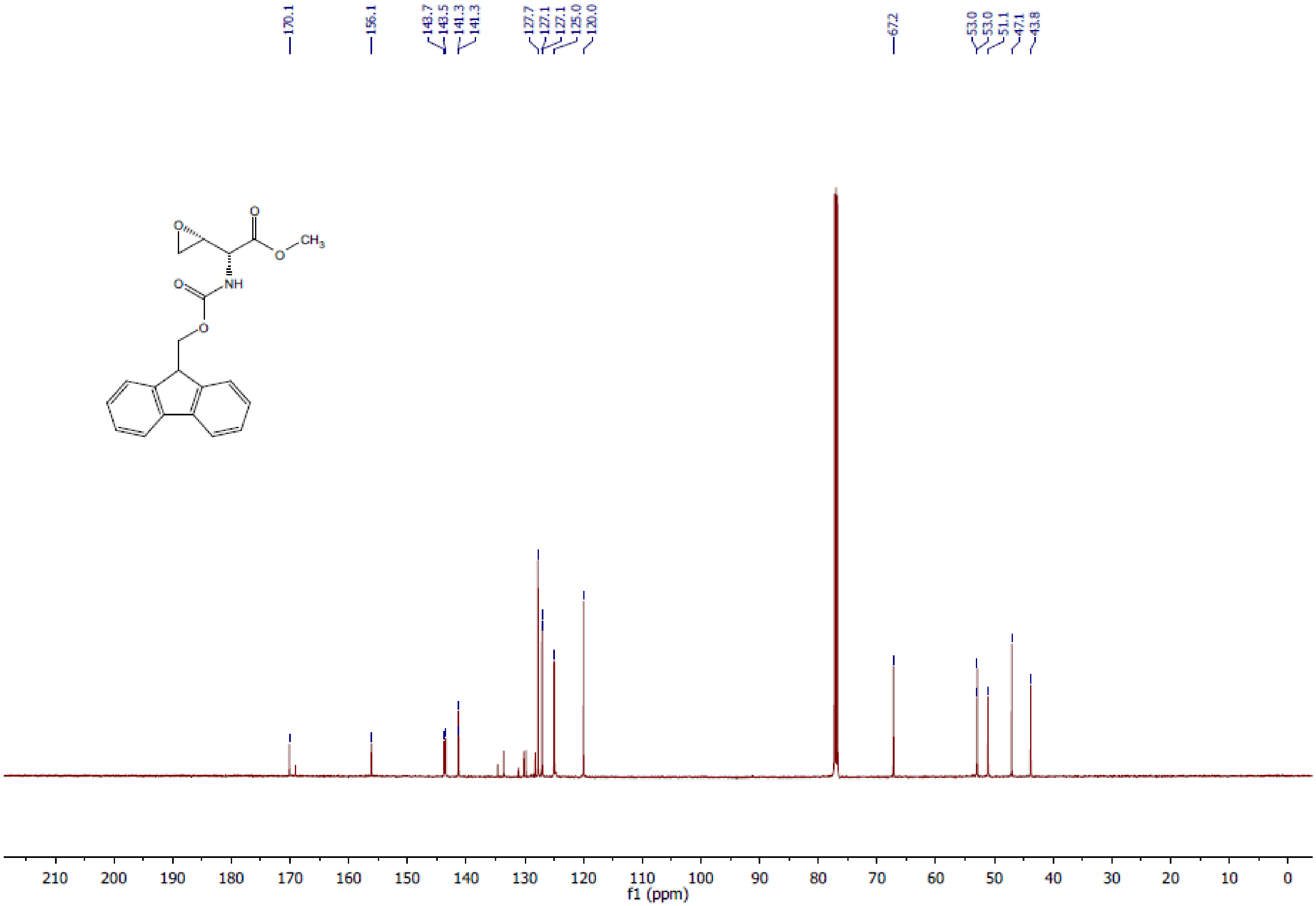

^{1}H NMR (500 MHz, $CDCl_3$) **(*R*, *S*)-18**

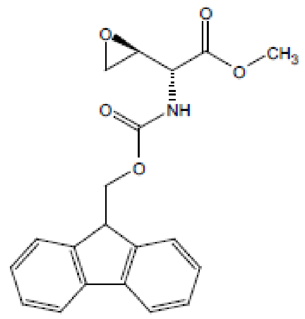

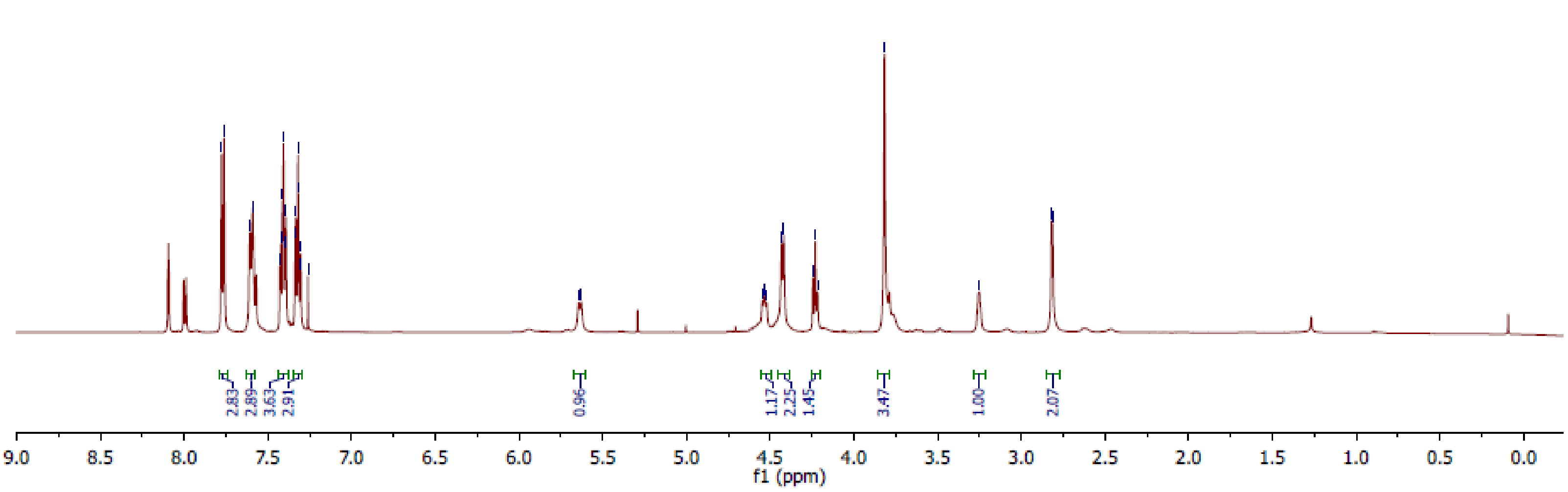

^{13}C NMR (126 MHz, $CDCl_3$) **(*R*, *S*)-18**

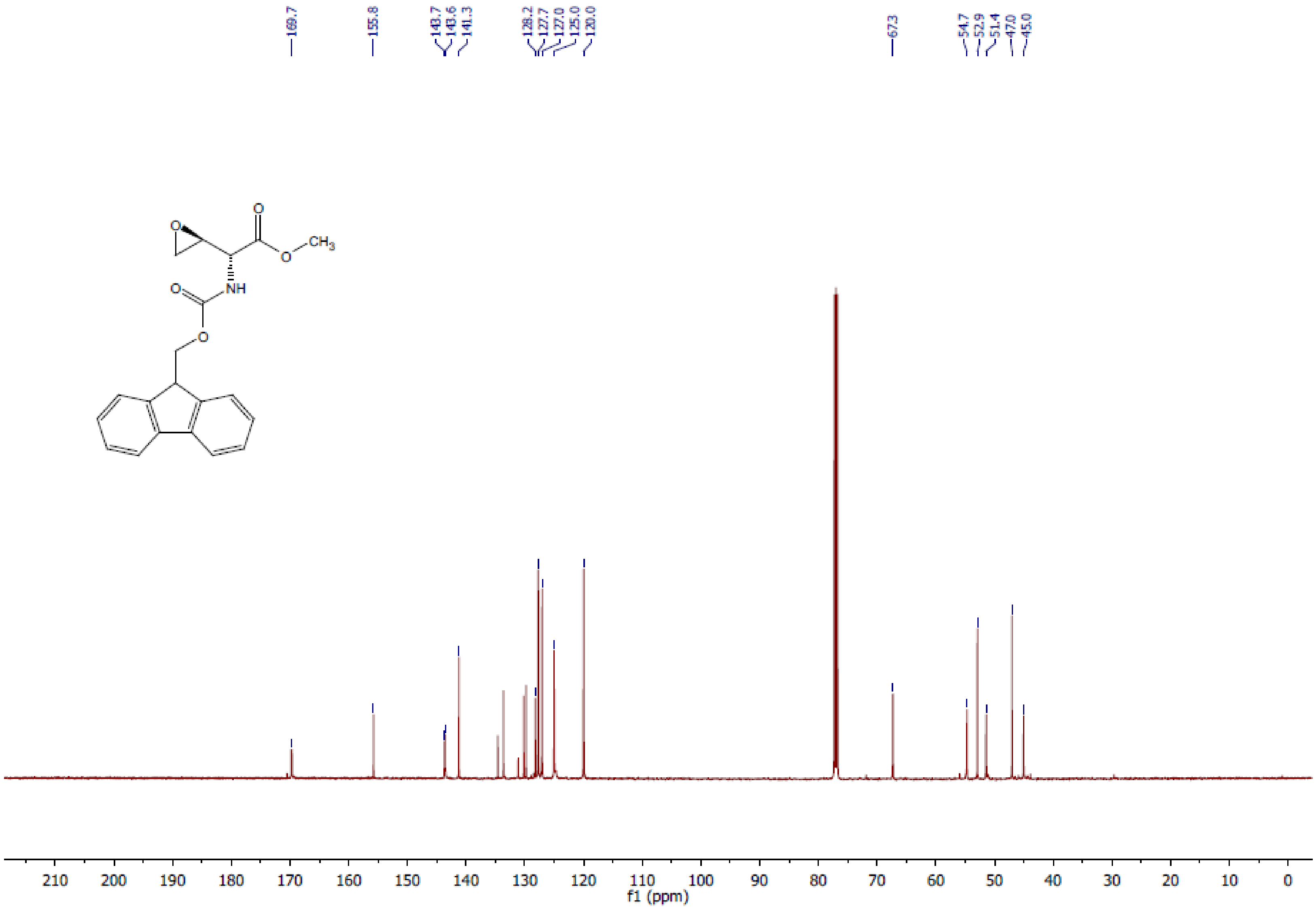

^{1}H NMR (500 MHz, $CDCl_3$) **[(3*R*, 4*S*, 2'*R*)-19]**

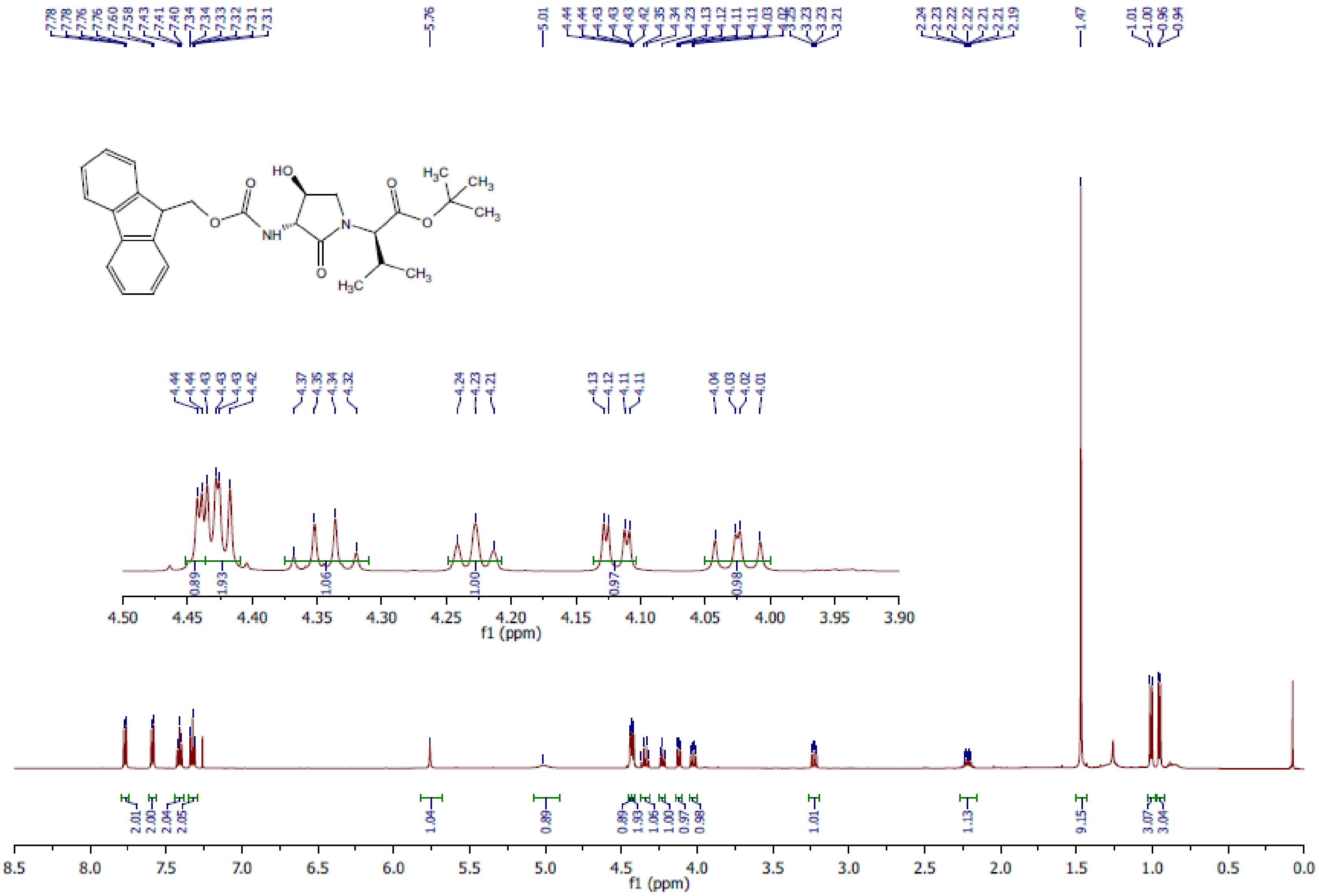

^{13}C NMR (126 MHz, $CDCl_3$) **[(3*R*, 4*S*, 2'*R*)-19]**

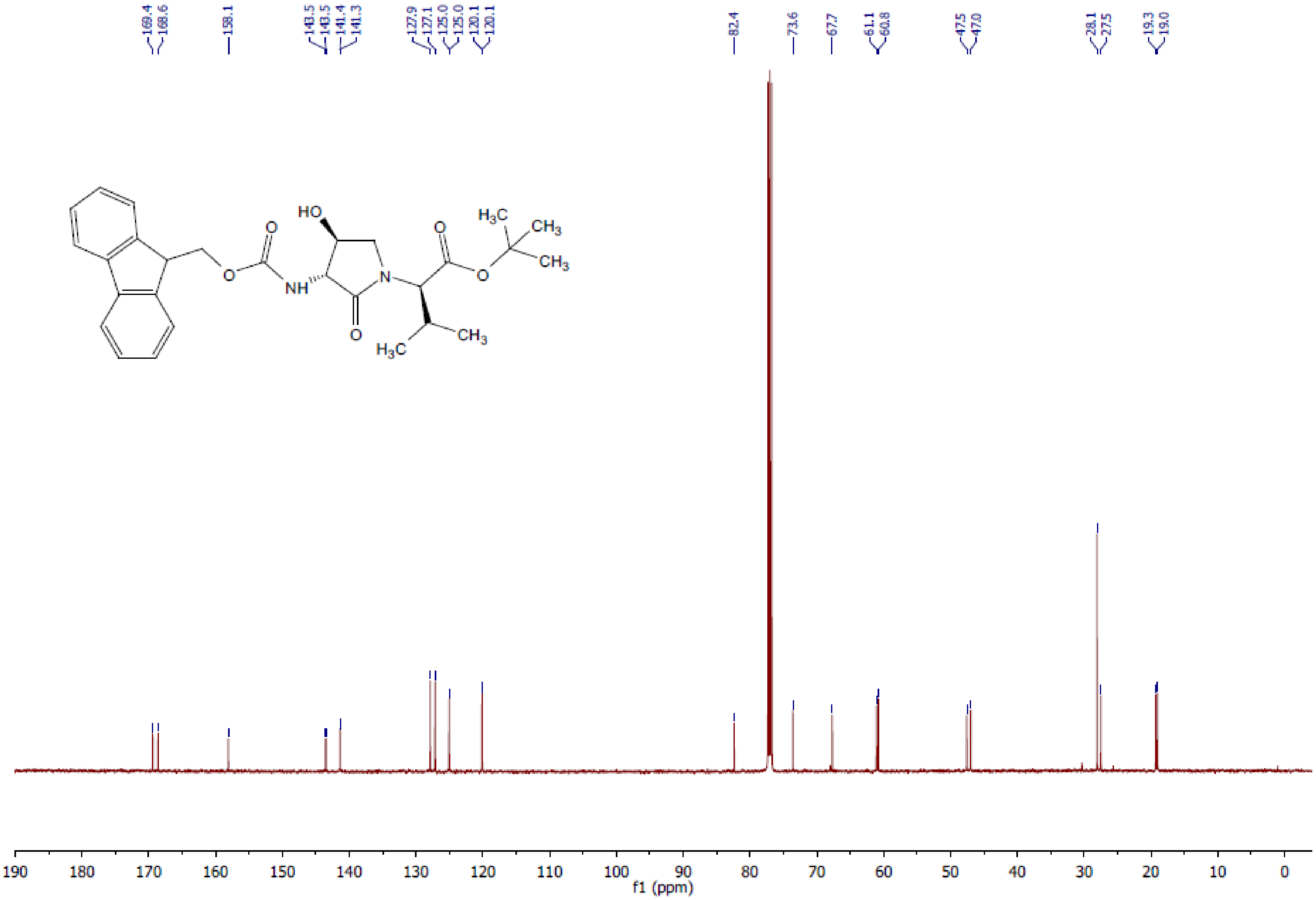

^{1}H NMR (500 MHz, $CDCl_3$) **[(3*R*, 4*S*, 2'*S*)-19]**

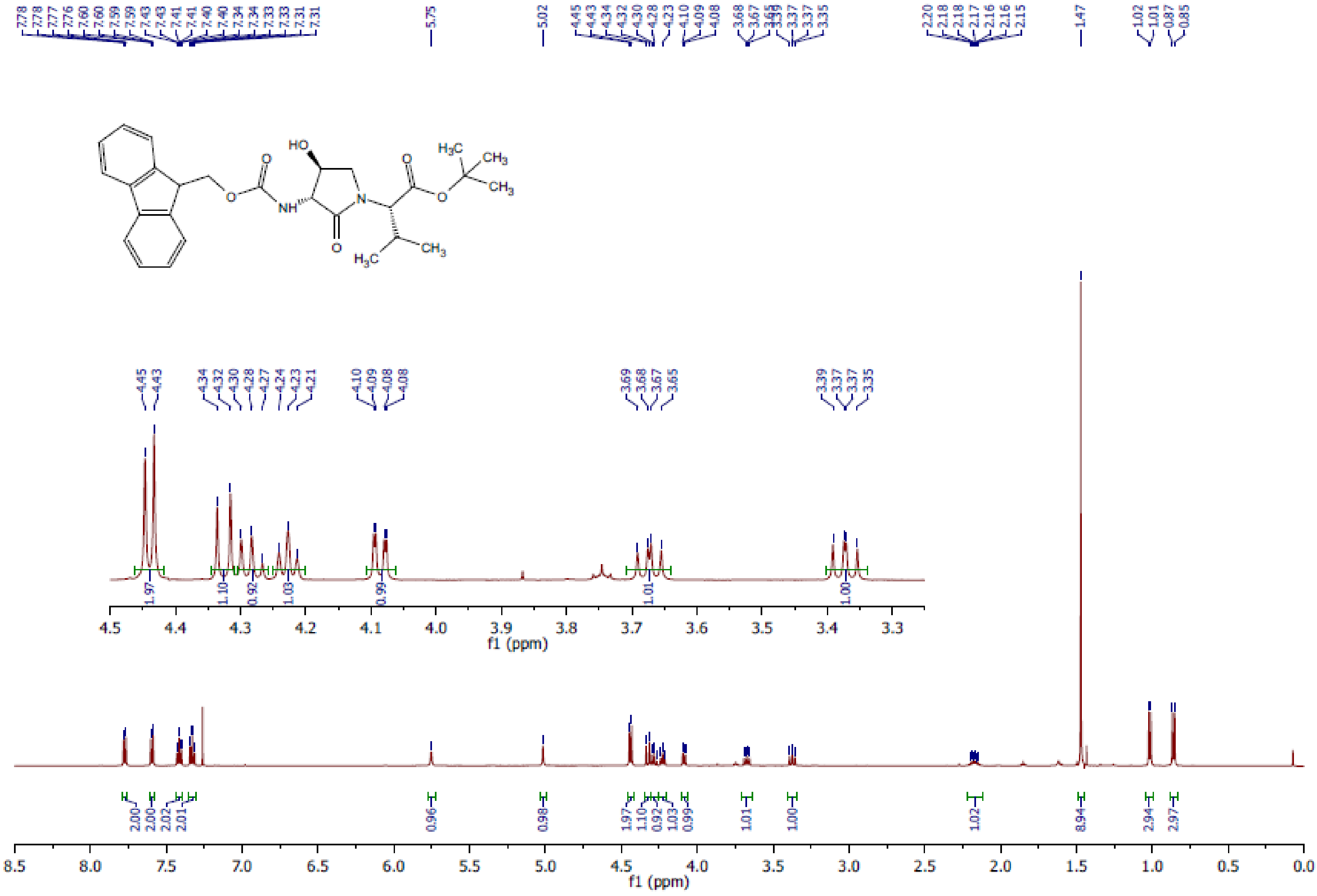

^{13}C NMR (126 MHz, $CDCl_3$) **[(3*R*, 4*S*, 2'*S*)-19]**

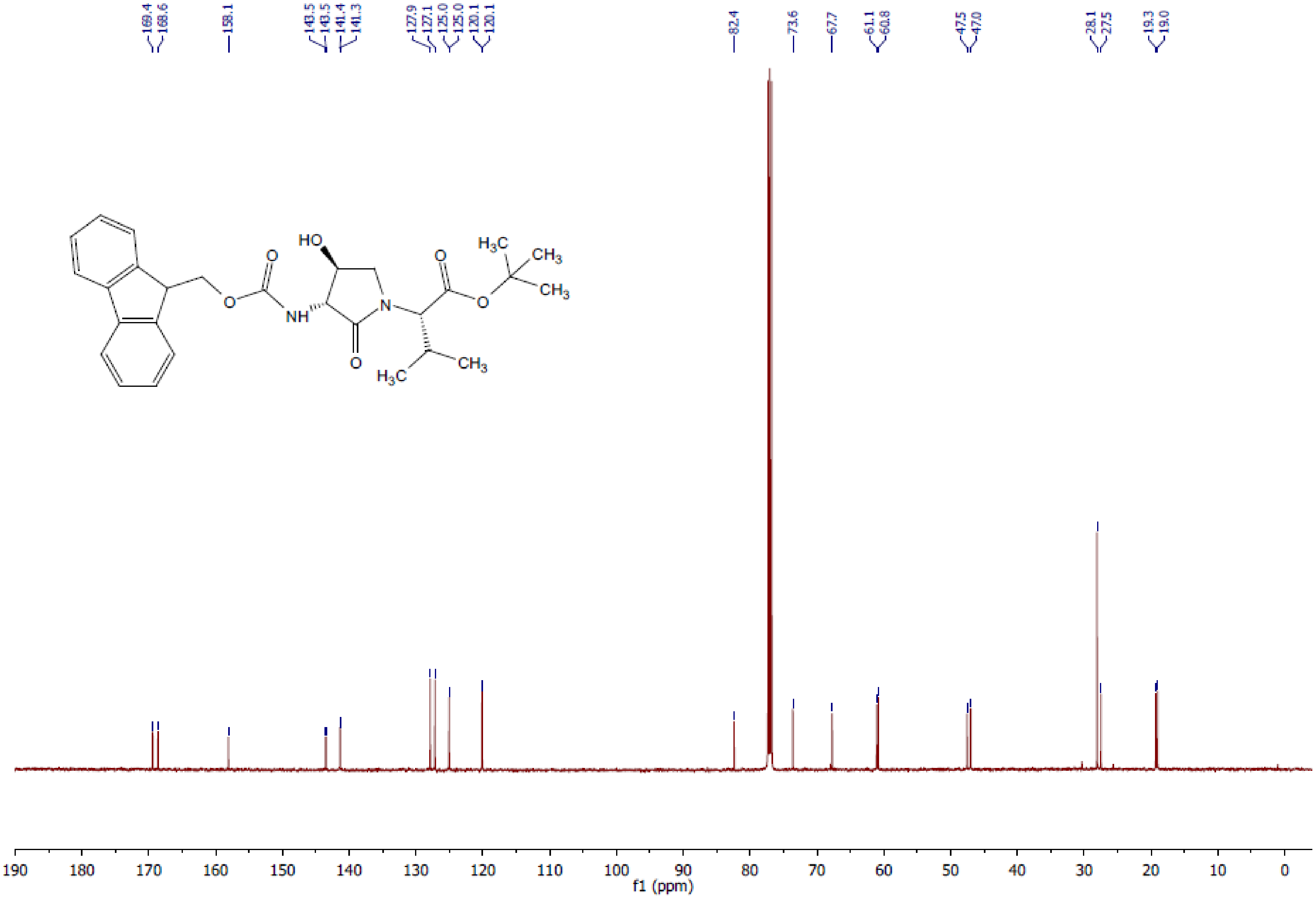

^{1}H NMR (500 MHz, $CDCl_3$) **[(3*S*, 4*R*, 2'*R*)-19]**

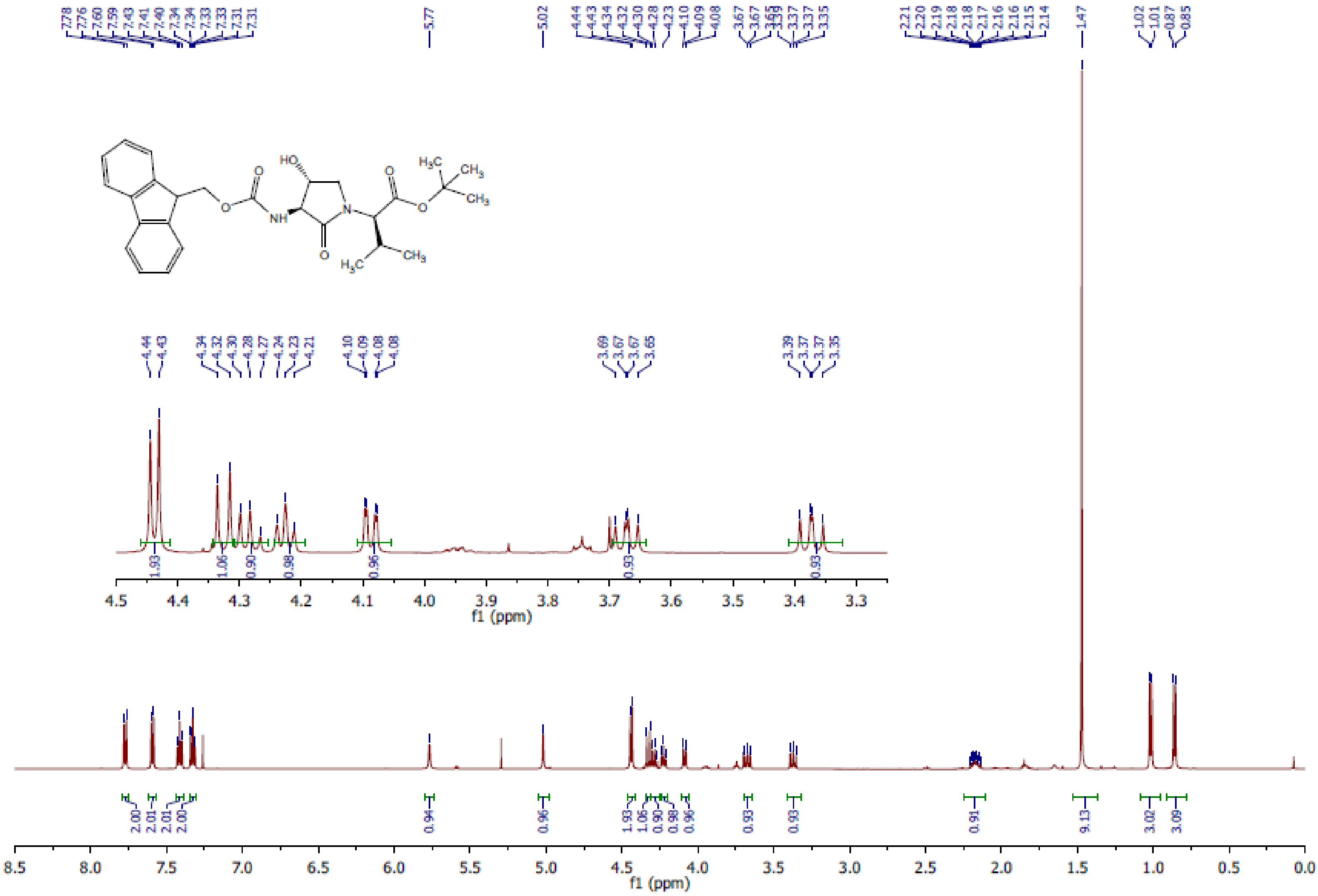

^{13}C NMR (75 MHz, $CDCl_3$) **[(3*S*, 4*R*, 2'*R*)-19]**

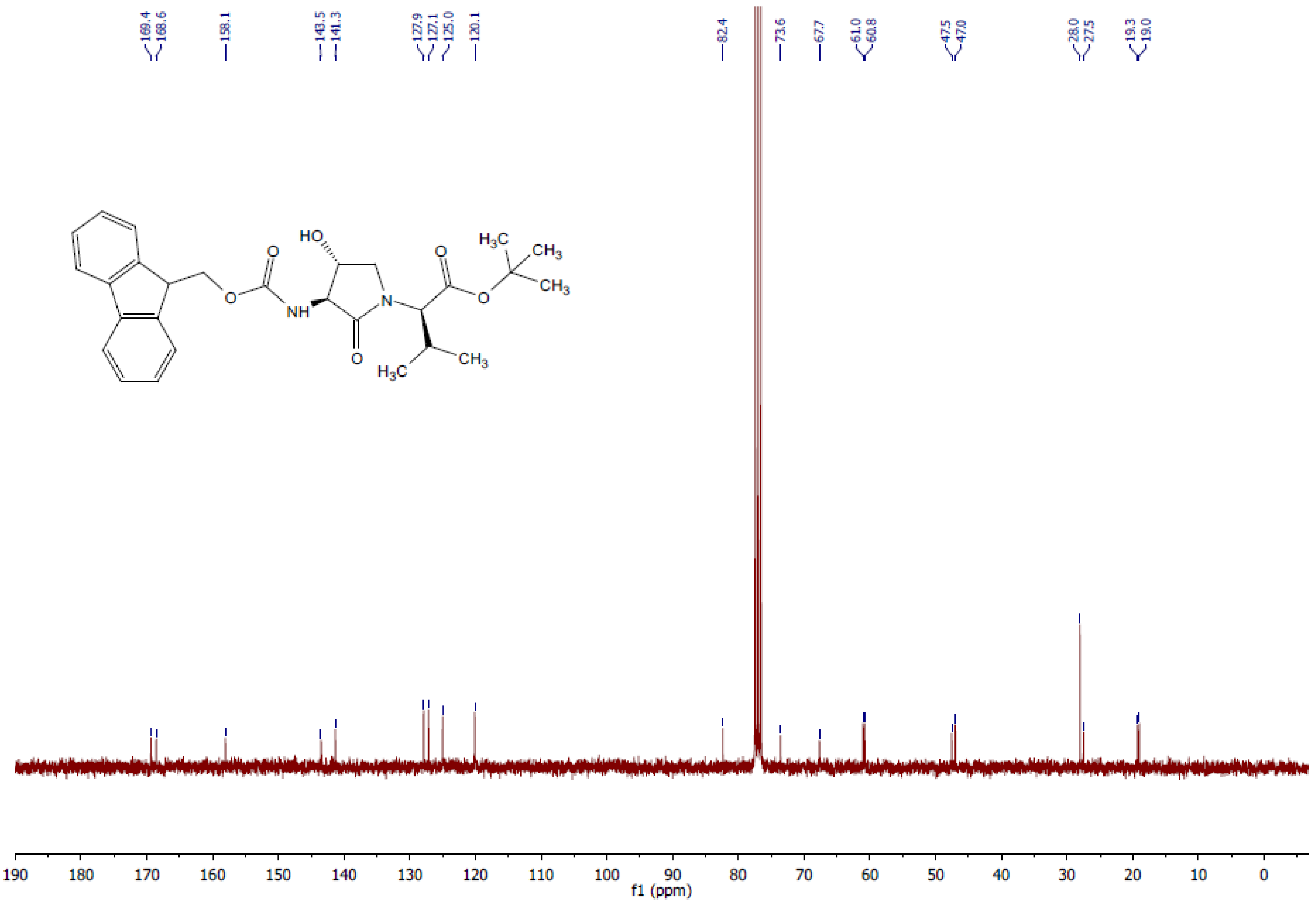

^{1}H NMR (500 MHz, $CDCl_3$) **[(3*S*, 4*R*, 2'*S*)-19]**

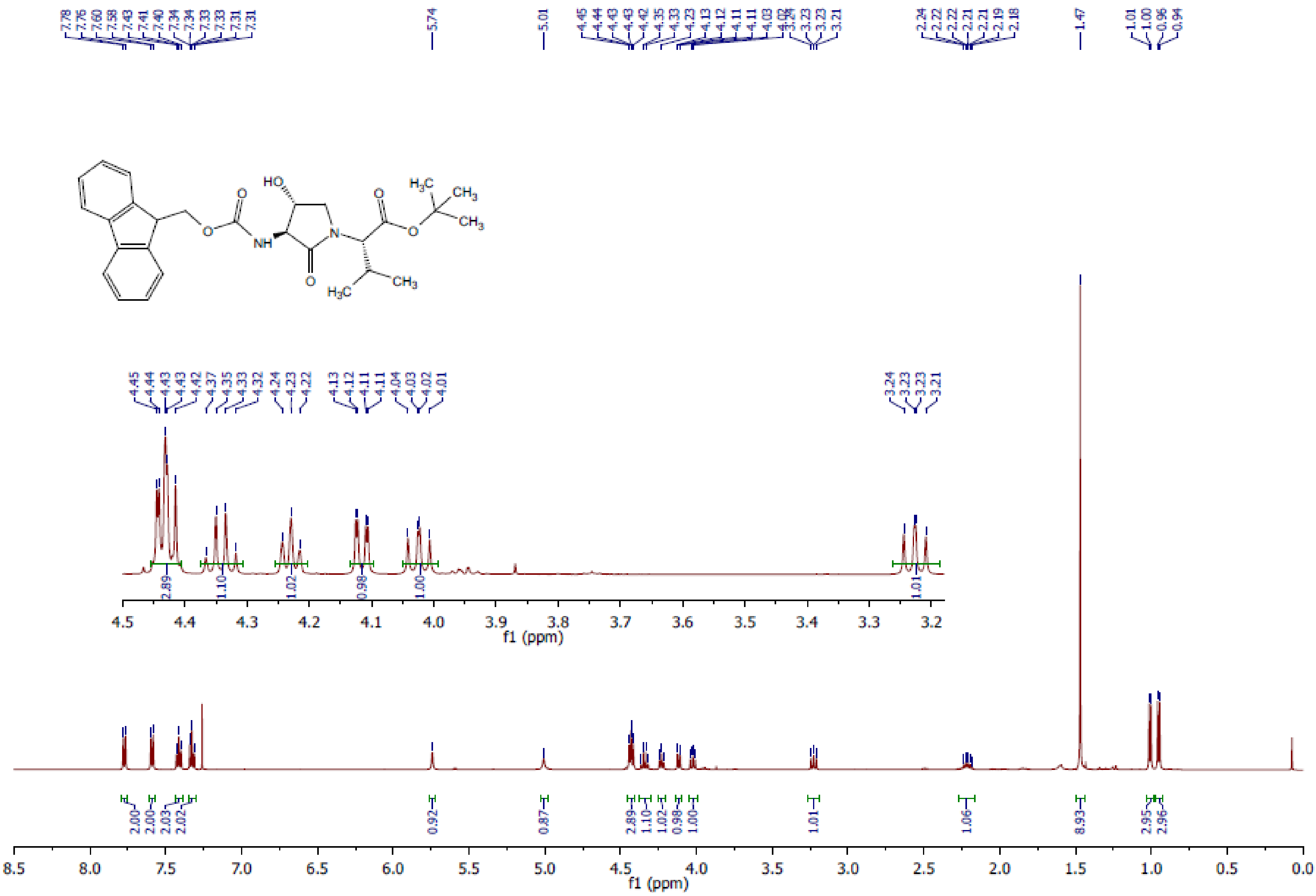

^{13}C NMR (126 MHz, CDCl3) **[(3*S*, 4*R*, 2'*S*)-19]**

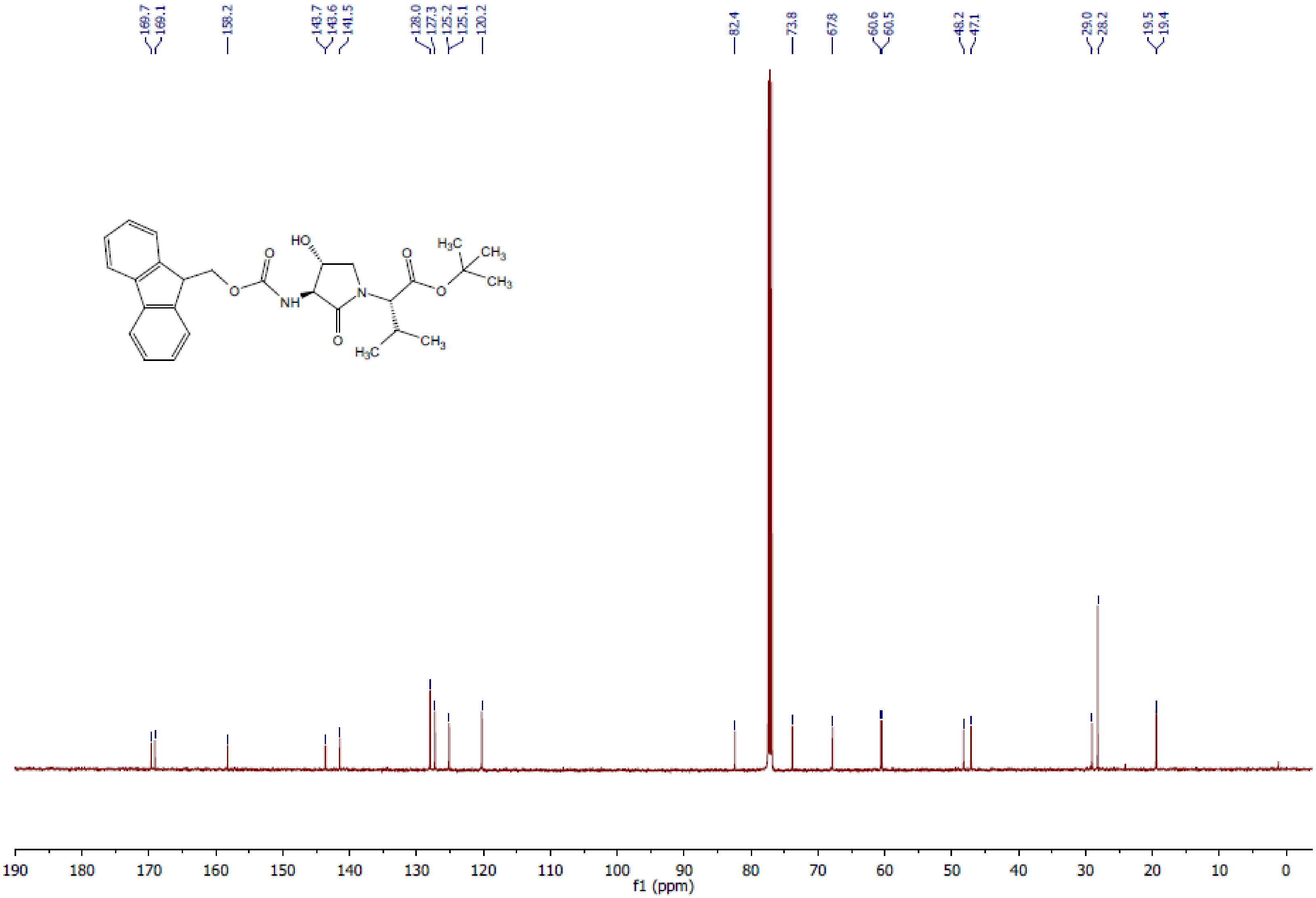

^{1}H NMR (400 MHz, DMSO) **[(3*R*, 4*R*, 2'*R*)-19]**

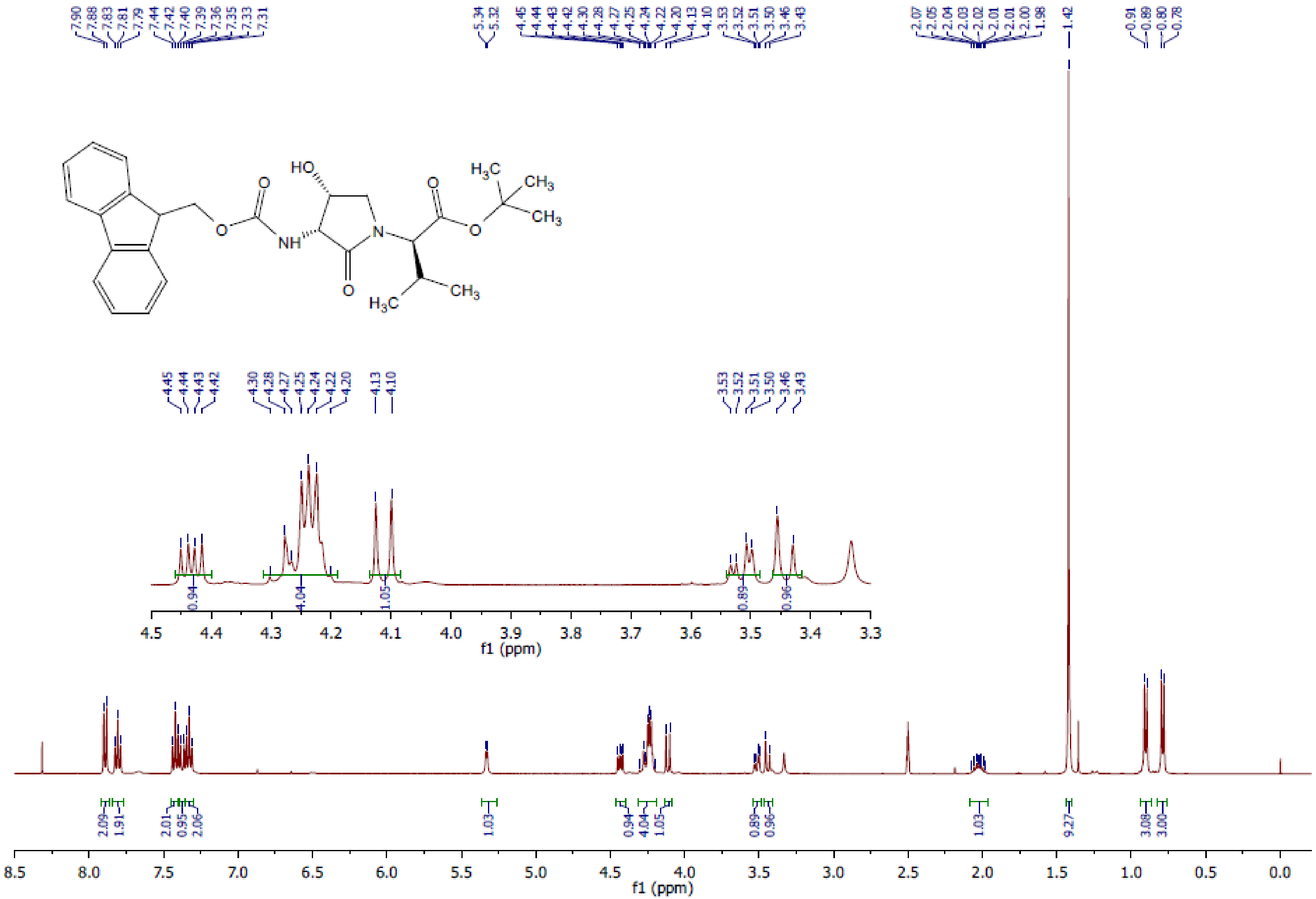

^{13}C NMR (75 MHz, $CDCl_3$) **[(3*R*, 4*R*, 2'*R*)-19]**

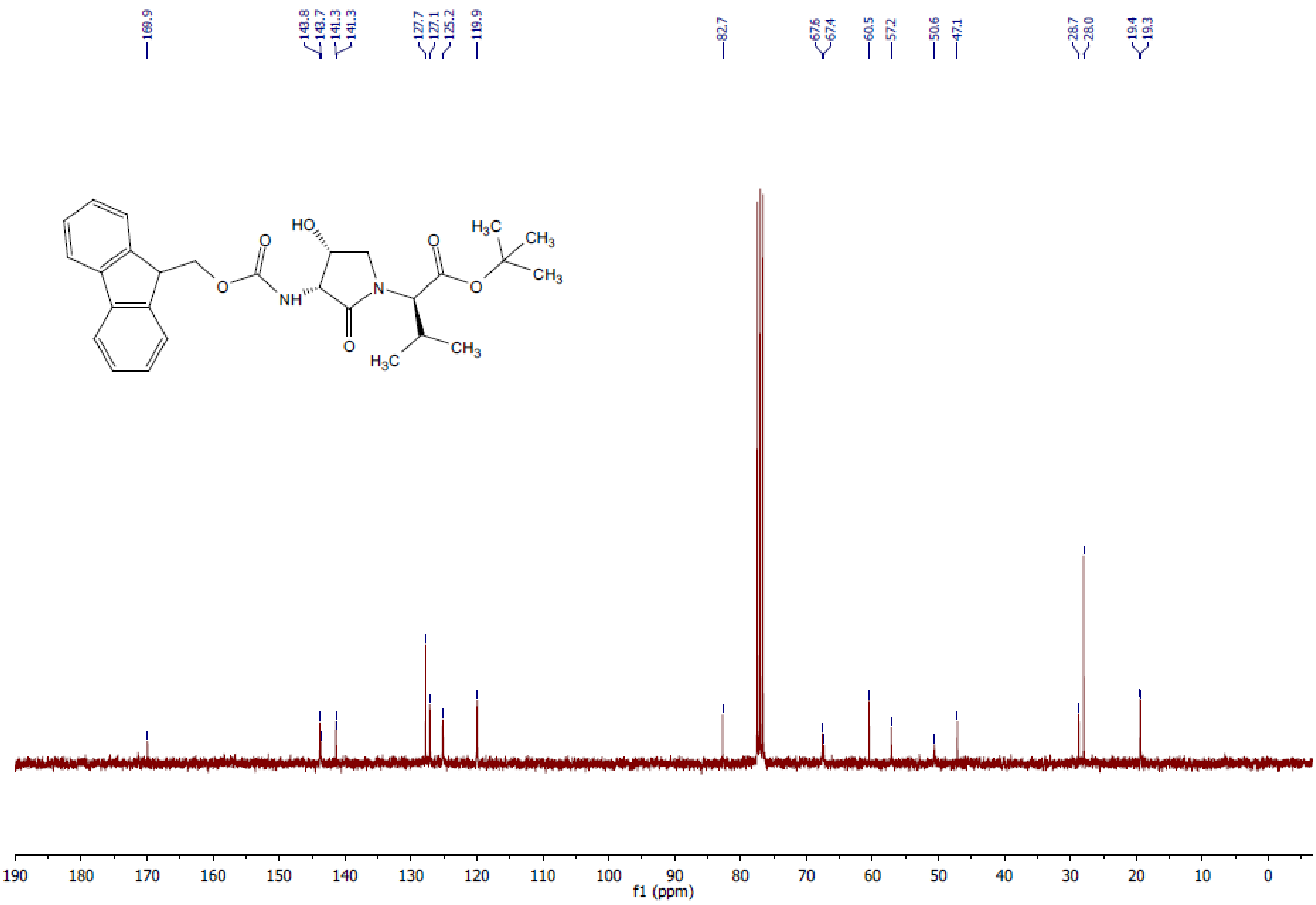

^{1}H NMR (300 MHz, DMSO) **[(3*R*, 4*R*, 2'*S*)-19]**

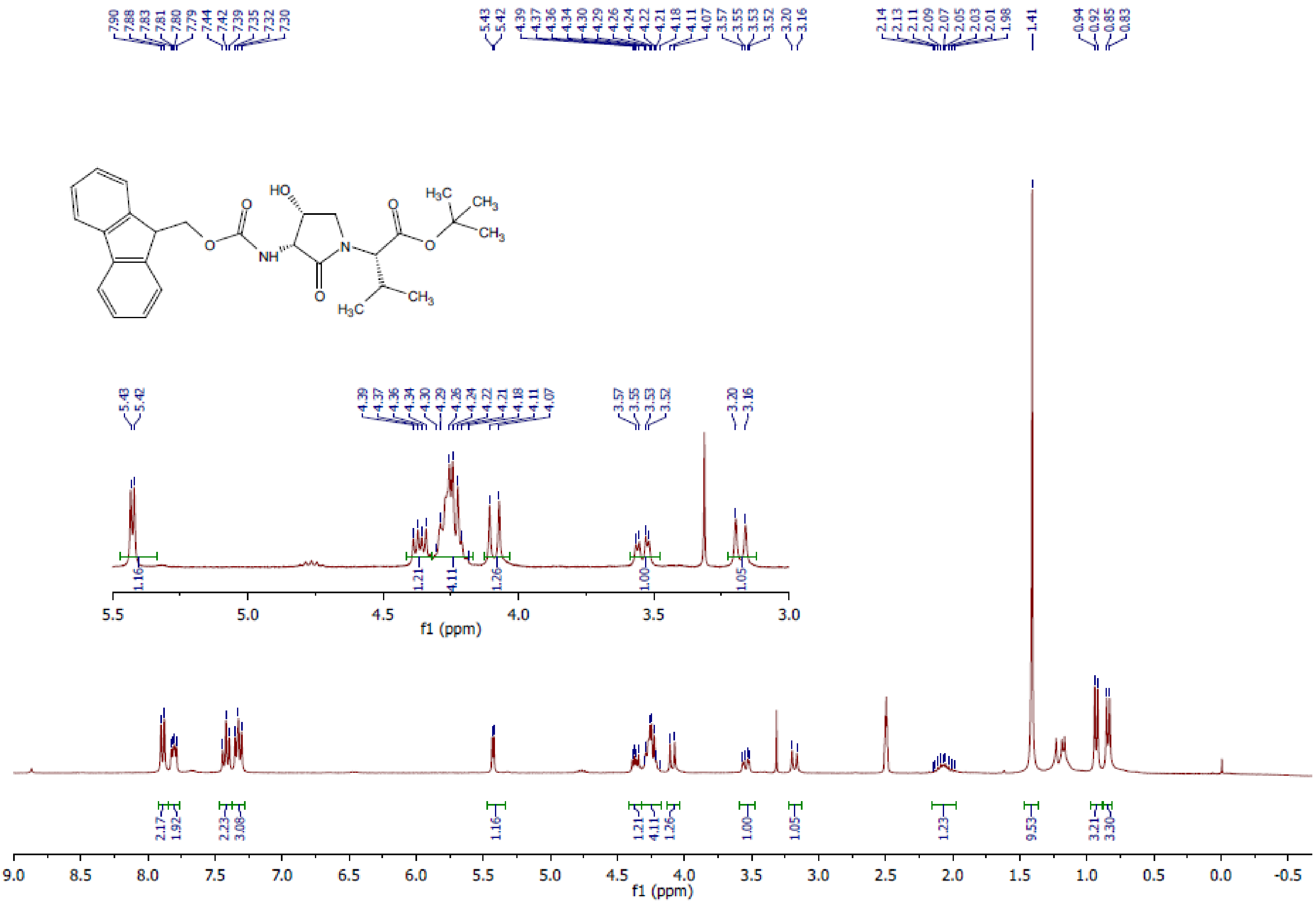

^{13}C NMR (75 MHz, $CDCl_3$) **[(3*R*, 4*R*, 2'*S*)-19]**

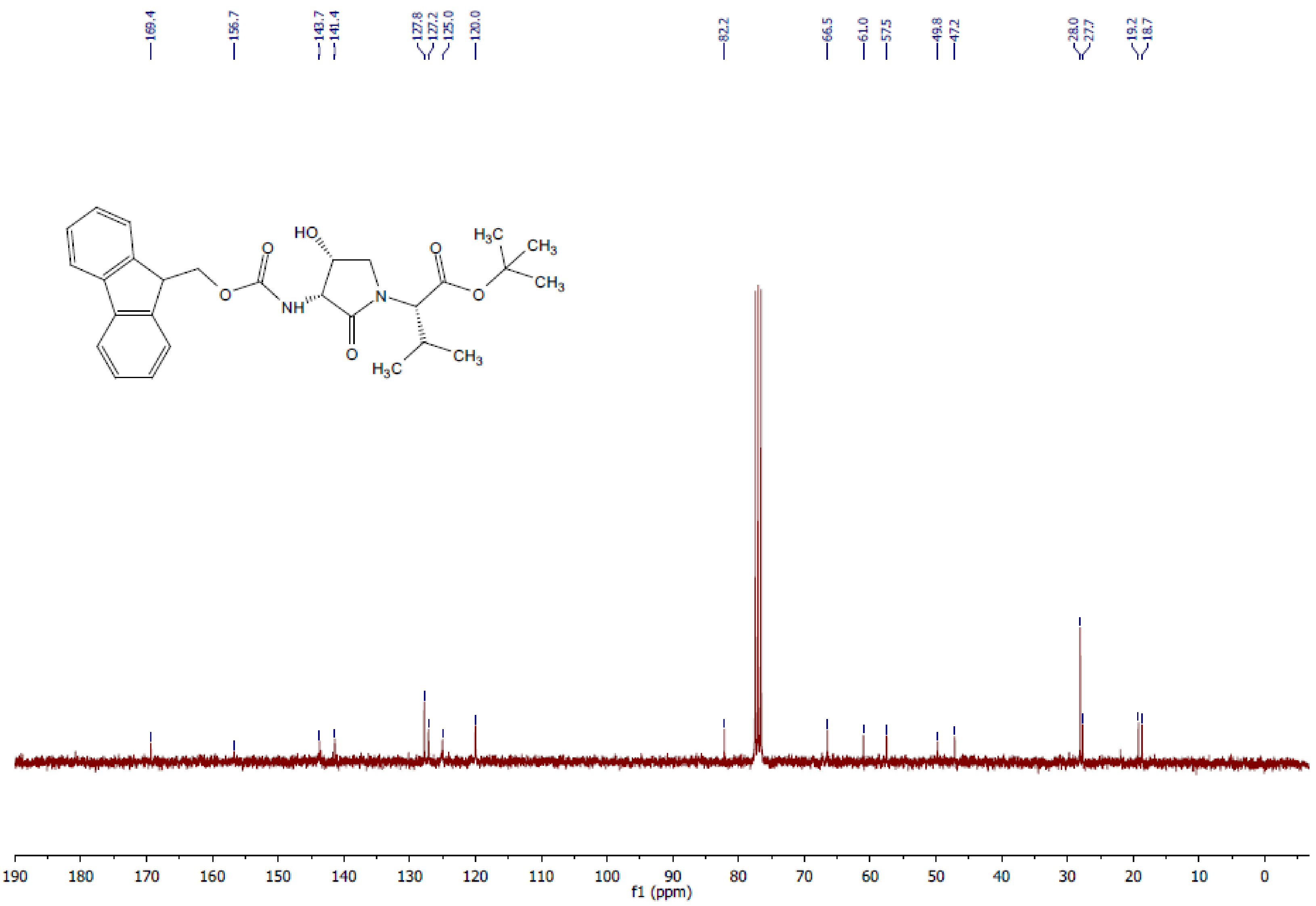

^{1}H NMR (300 MHz, $CDCl_3$) **[(3*S*, 4*S*, 2'*R*)-19]**

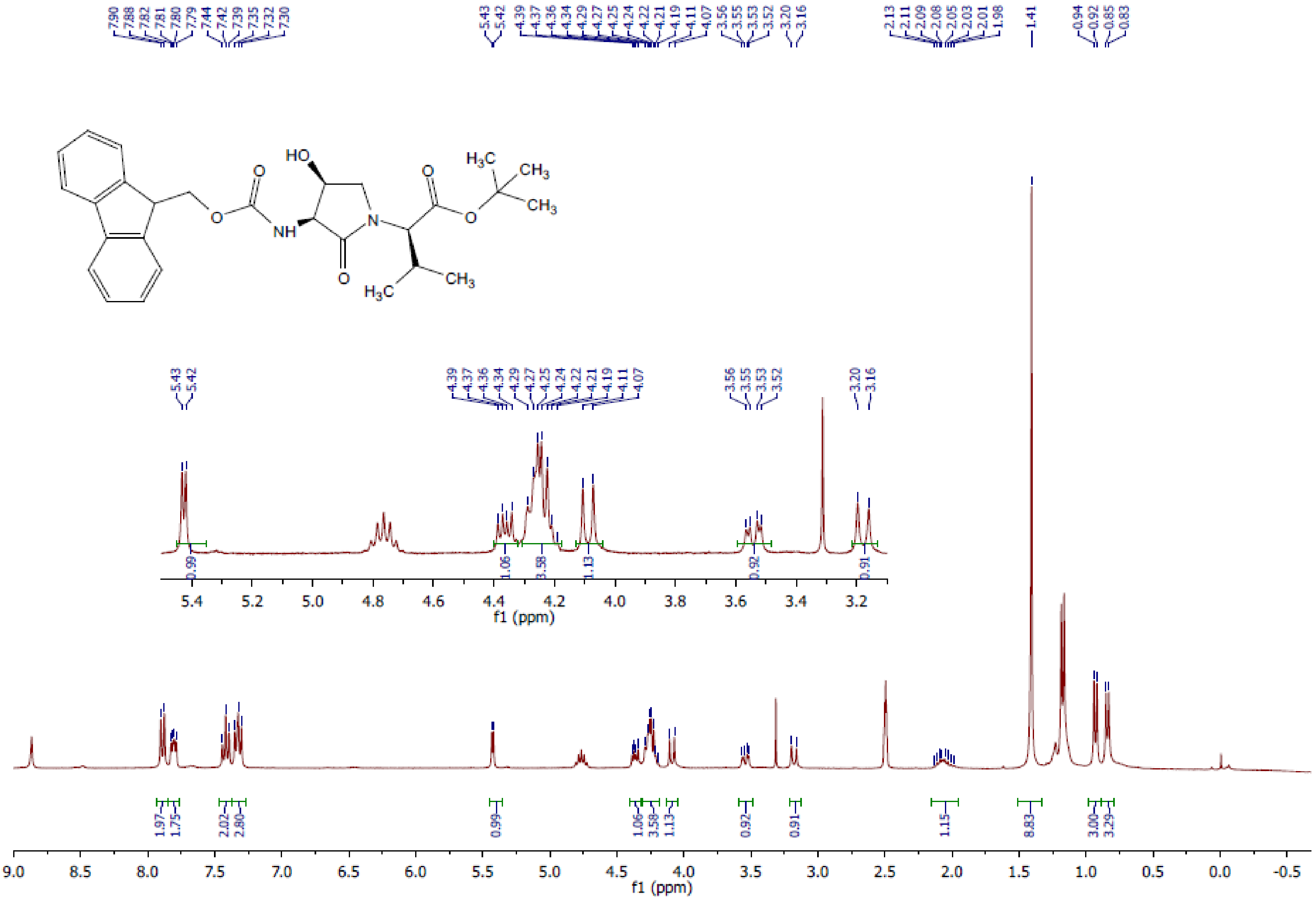

^{13}C NMR (75 MHz, CDCl$_3$) **[(3*S*, 4*S*, 2'*R*)-19]**

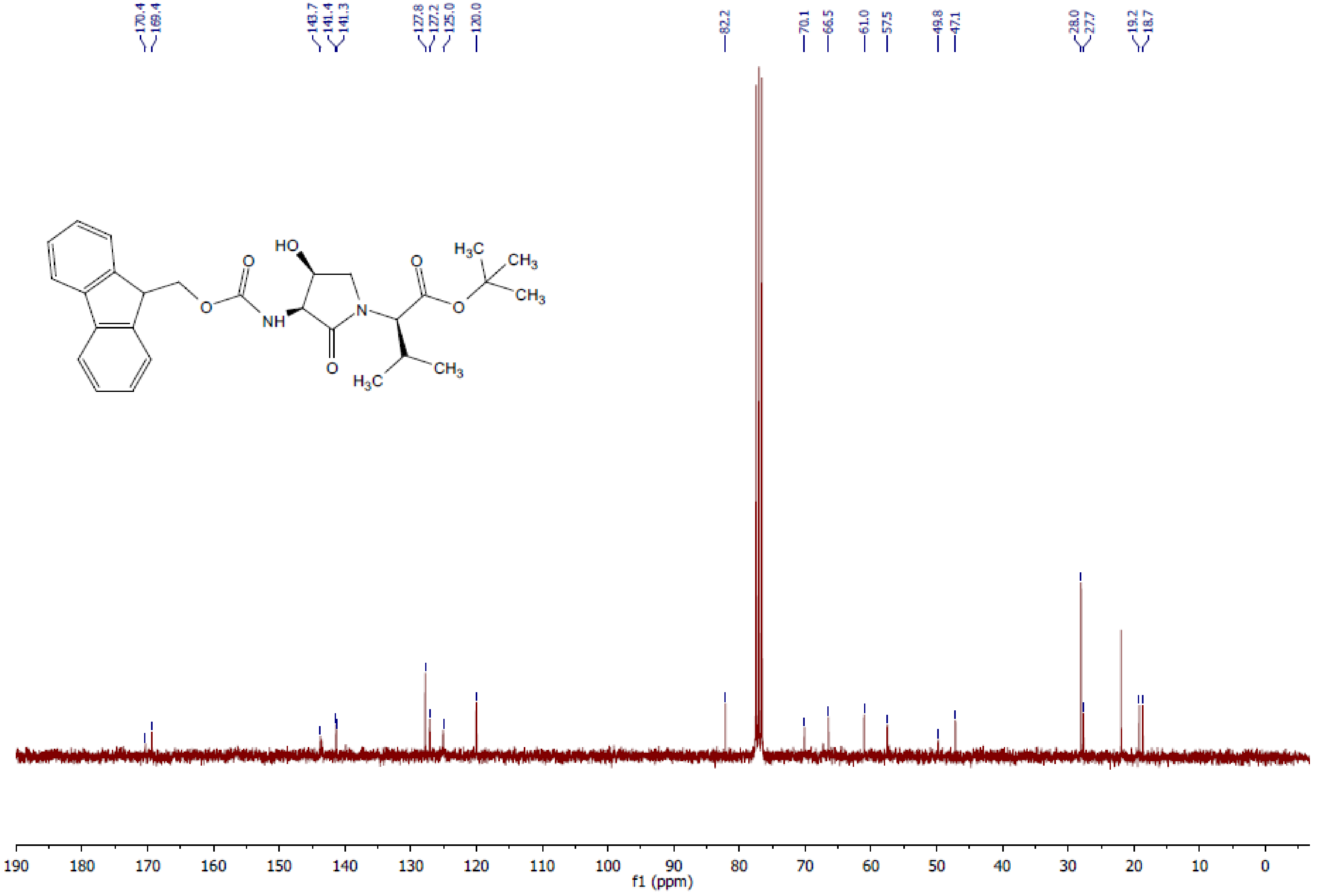

^{1}H NMR (300 MHz, DMSO) **[(3*S*, 4*S*, 2'*S*)-19]**

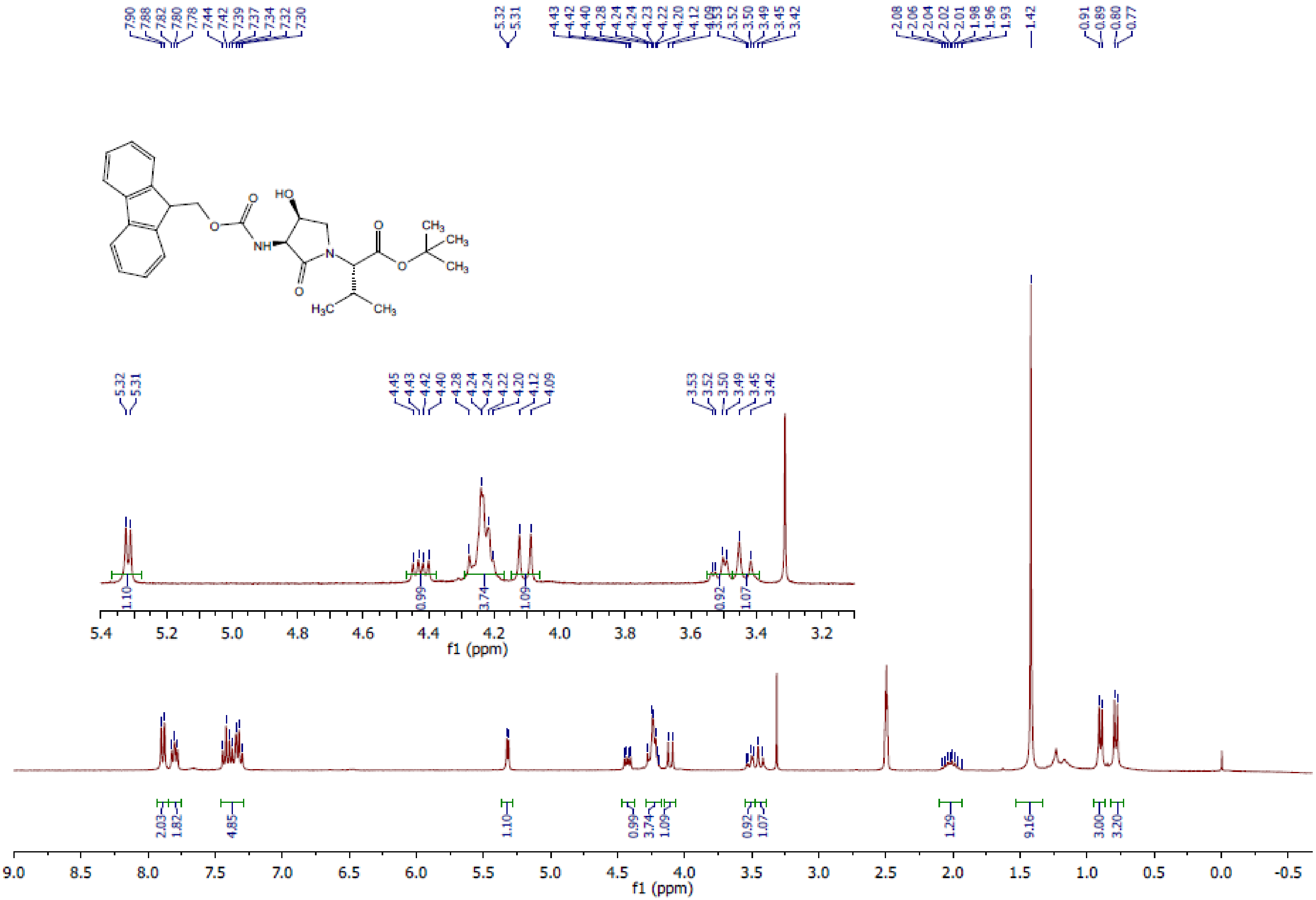

^{13}C NMR (75 MHz, $CDCl_3$) **[(3*S*, 4*S*, 2'*S*)-19]**

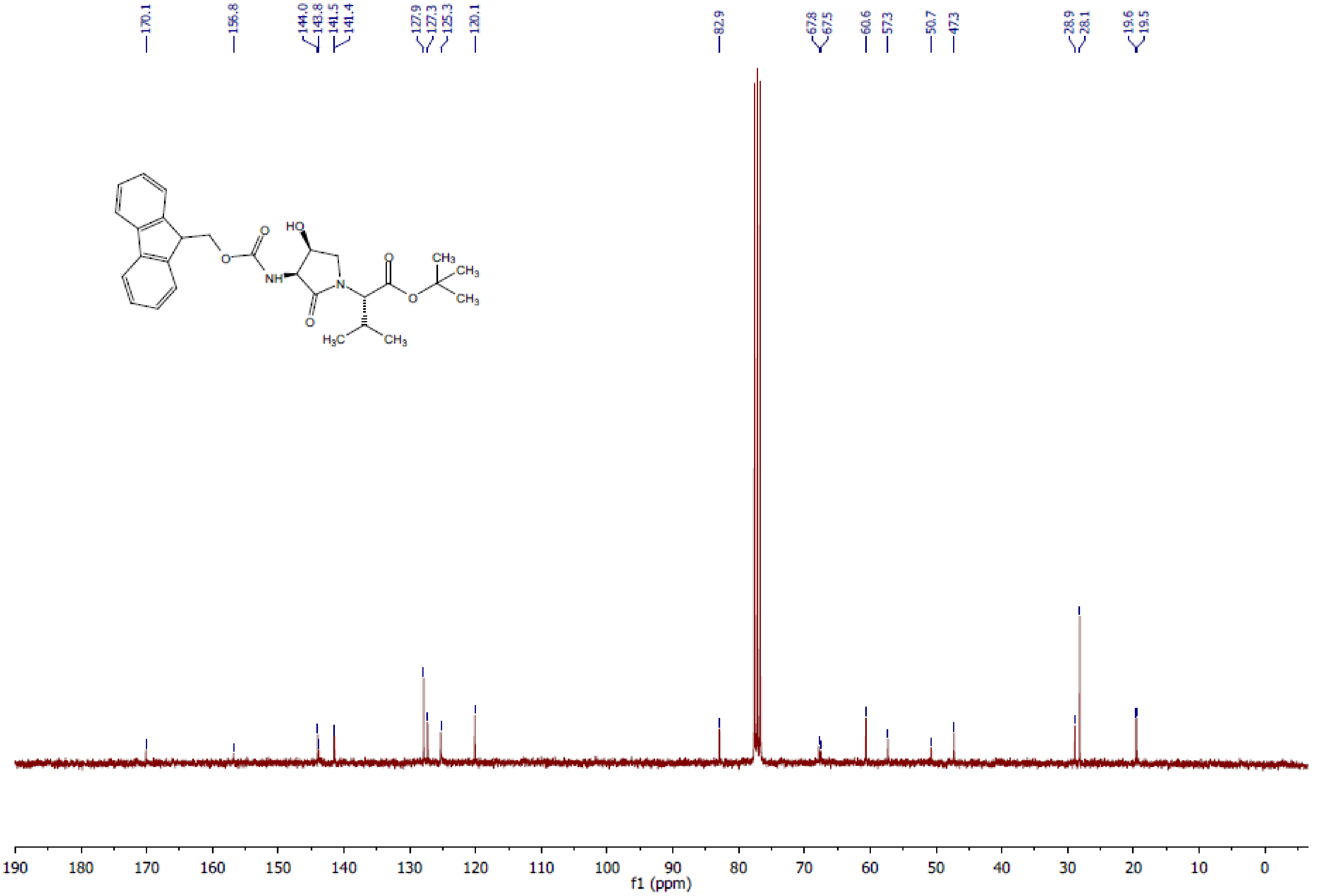

^{1}H NMR (300 MHz, $CDCl_3$) **[(3*R*, 4*R*, 2'*R*-20]**

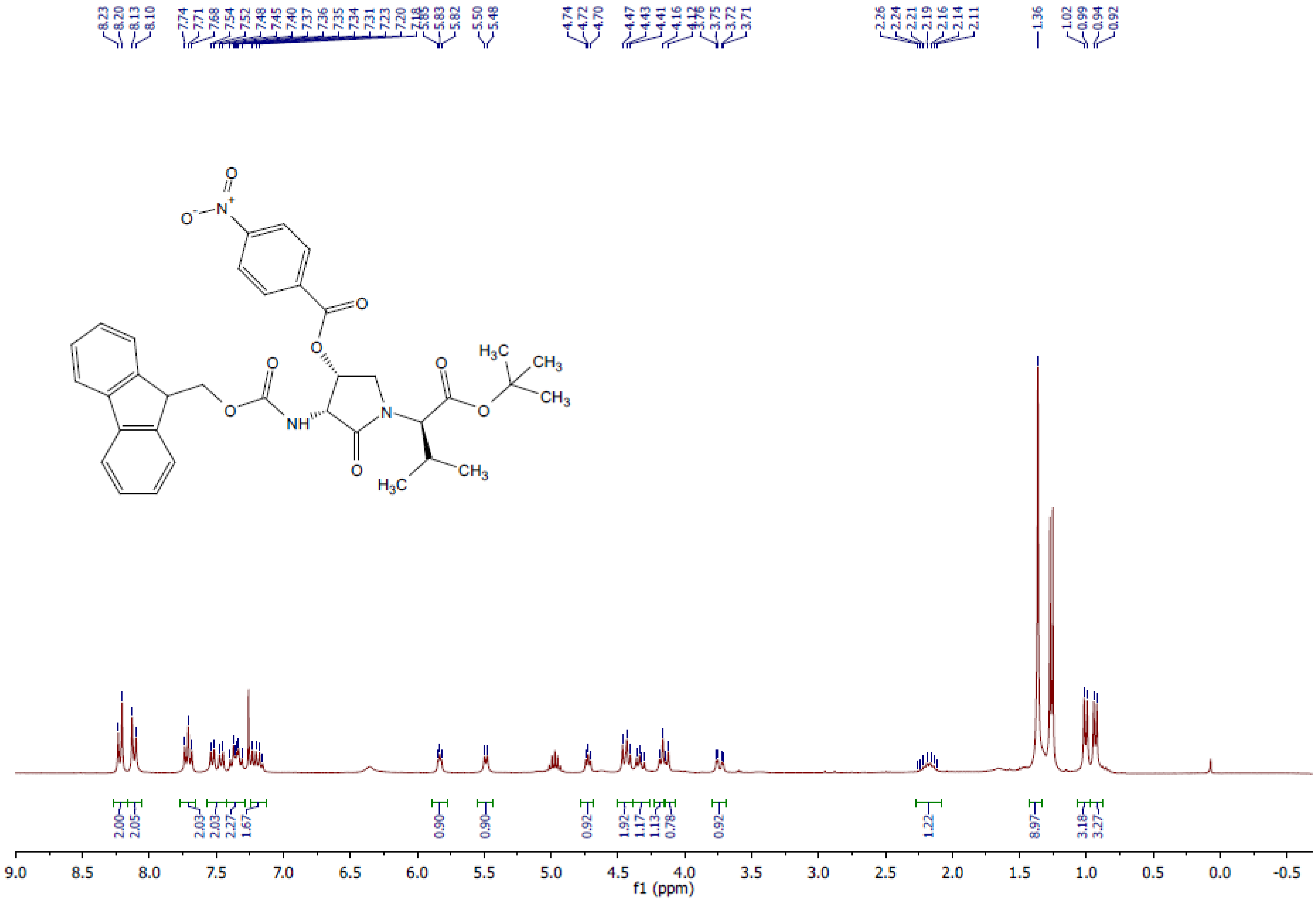

^{13}C NMR (75 MHz, $CDCl_3$) **[(3*R*, 4*R*, 2'*R*)-20]**

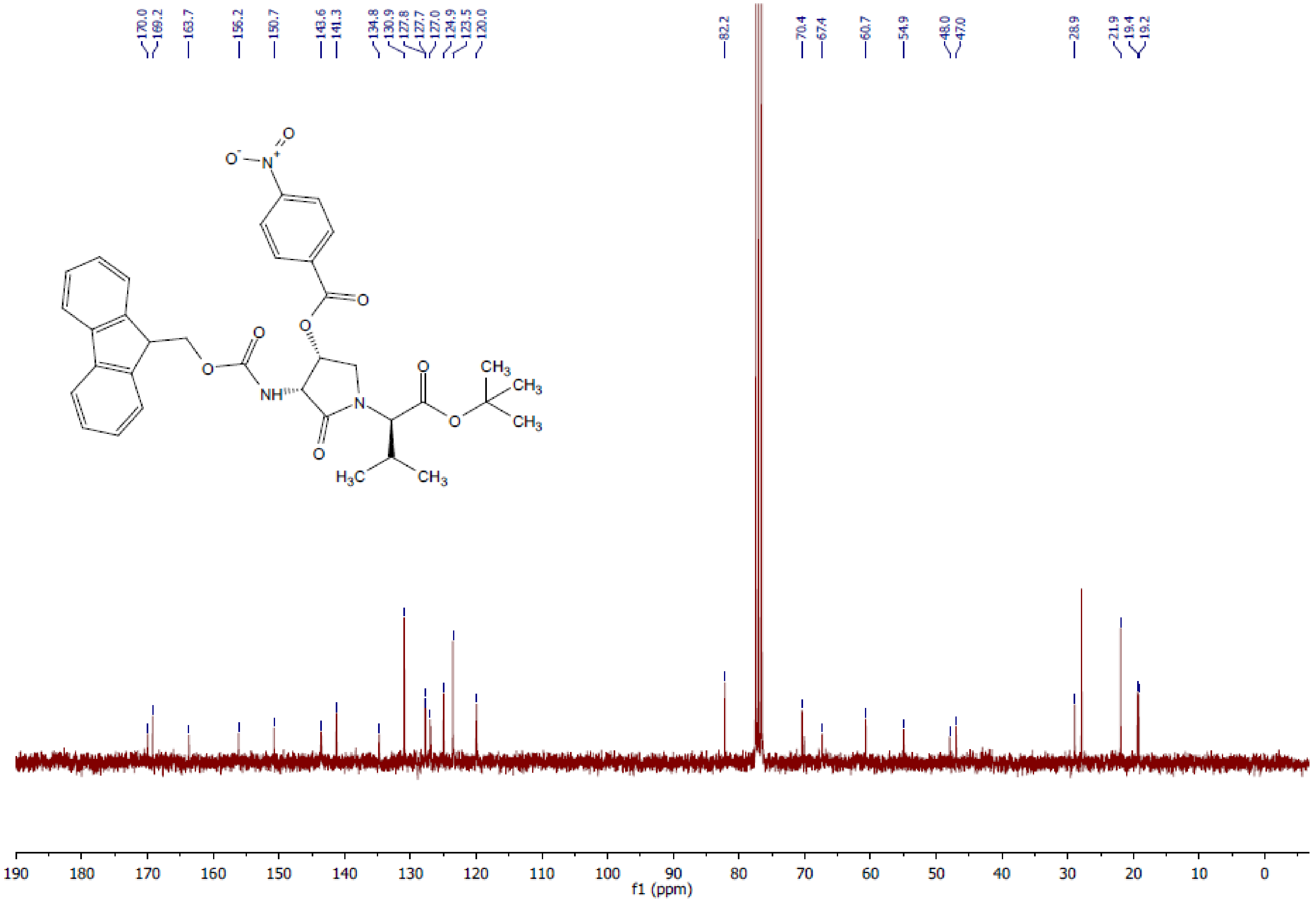

^{1}H NMR (300 MHz, $CDCl_3$) **[(3*R*, 4*R*, 2'*S*)-20]**

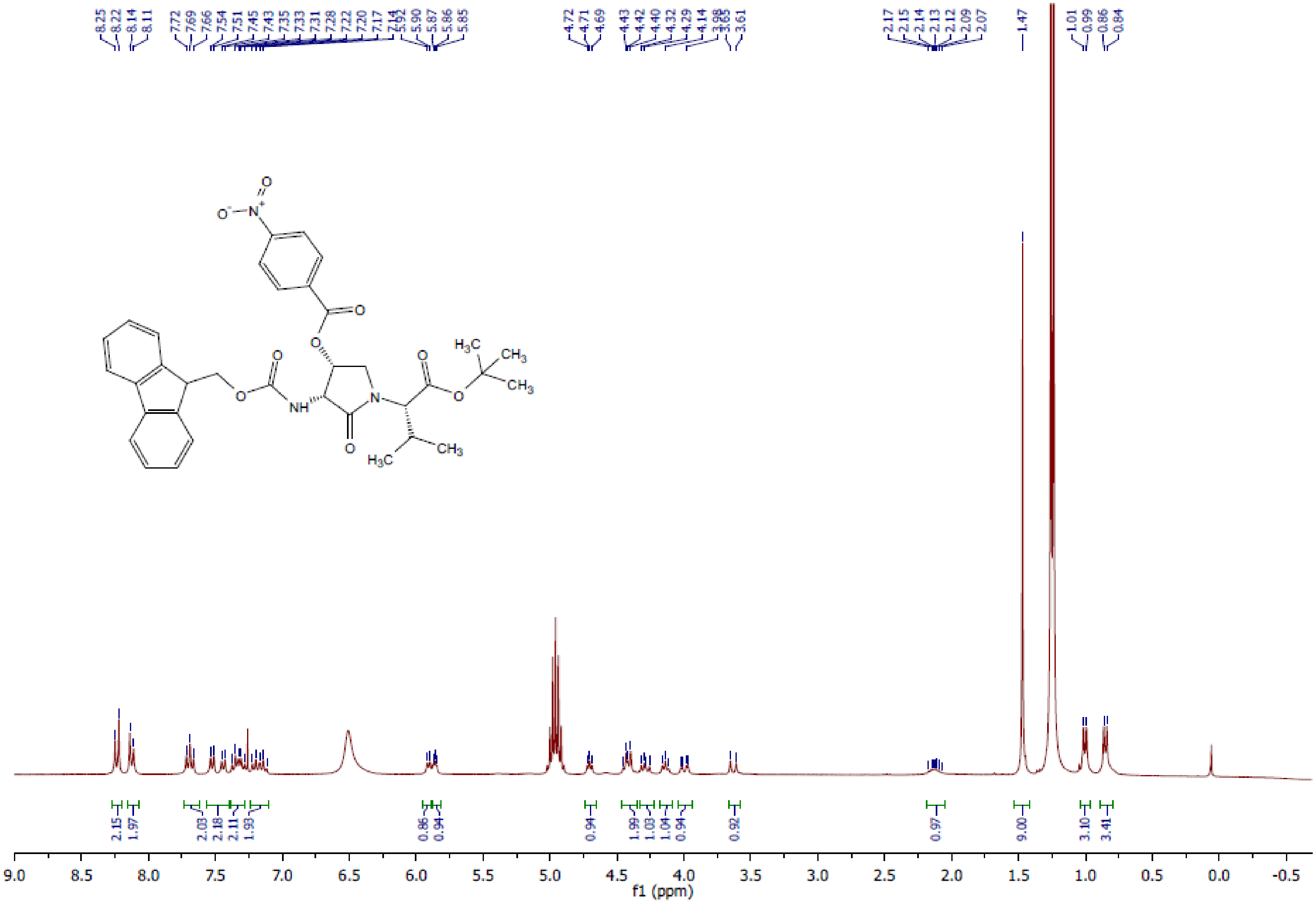

^{13}C NMR (75 MHz, $CDCl_3$) **[(3*R*, 4*R*, 2'*S*)-20**

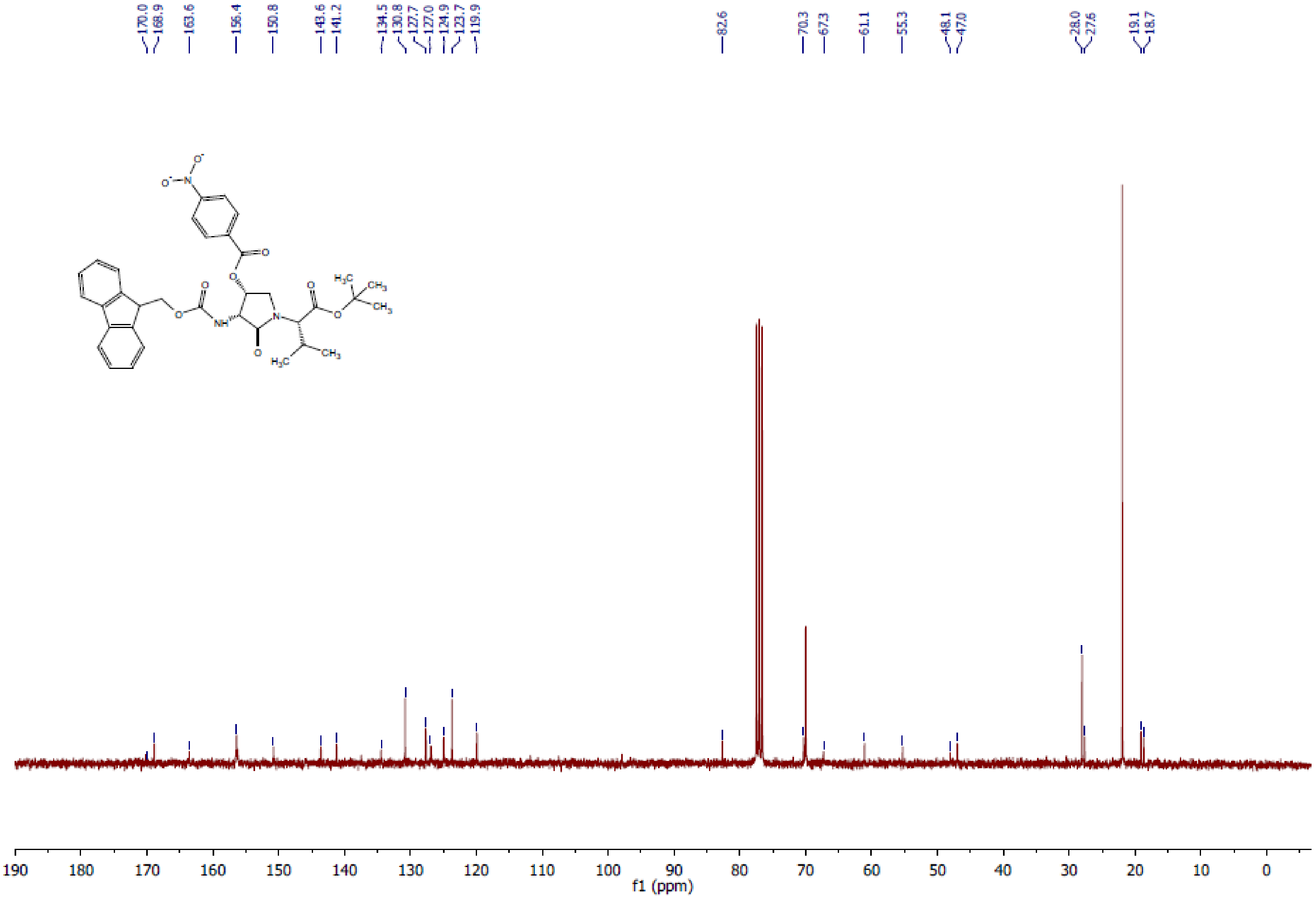

^{1}H NMR (300 MHz, $CDCl_3$) **[(3*S*, 4*S*, 2'*R*)-20]**

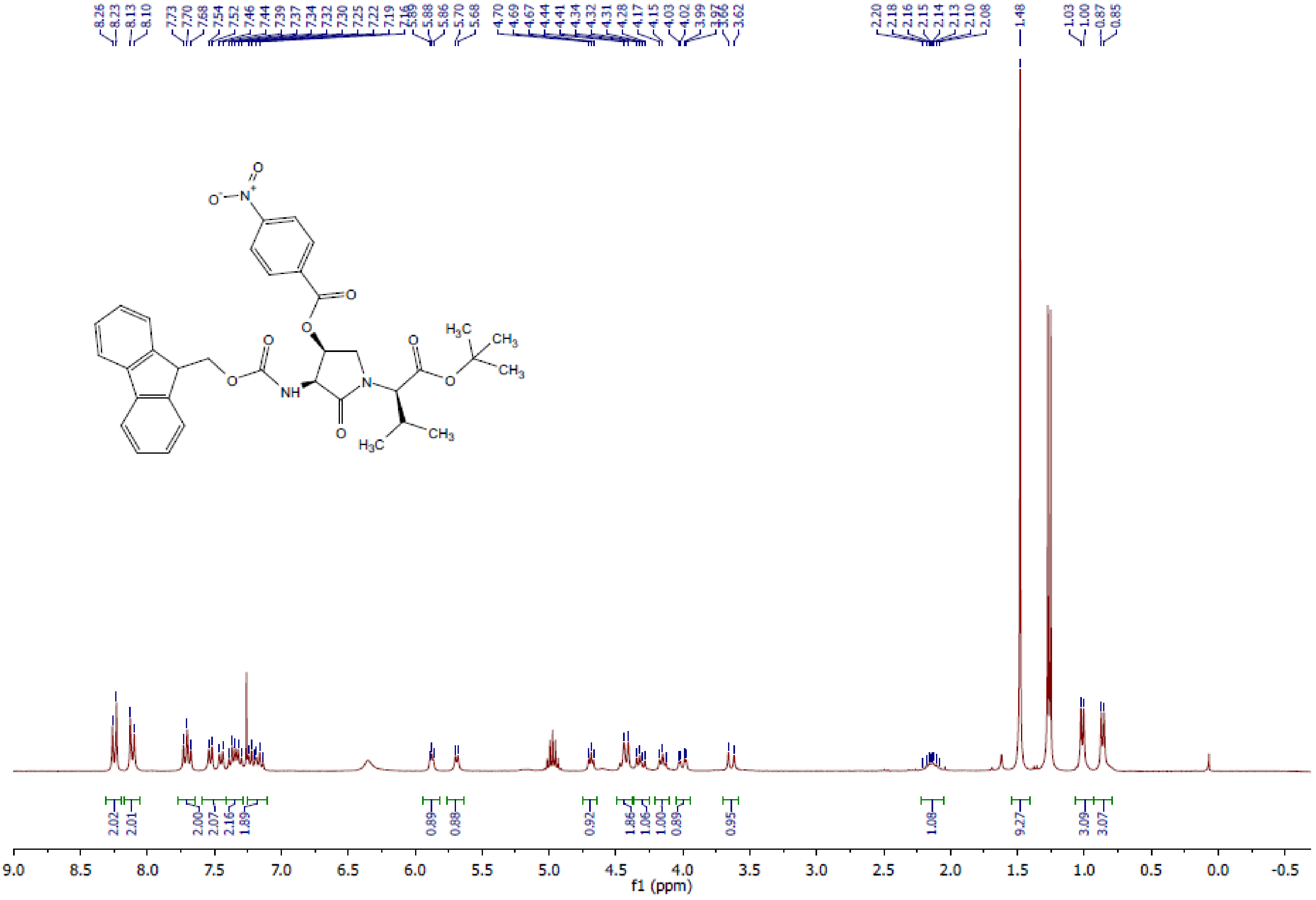

^{13}C NMR (75 MHz, $CDCl_3$) **[(3*S*, 4*S*, 2'*R*)-20]**

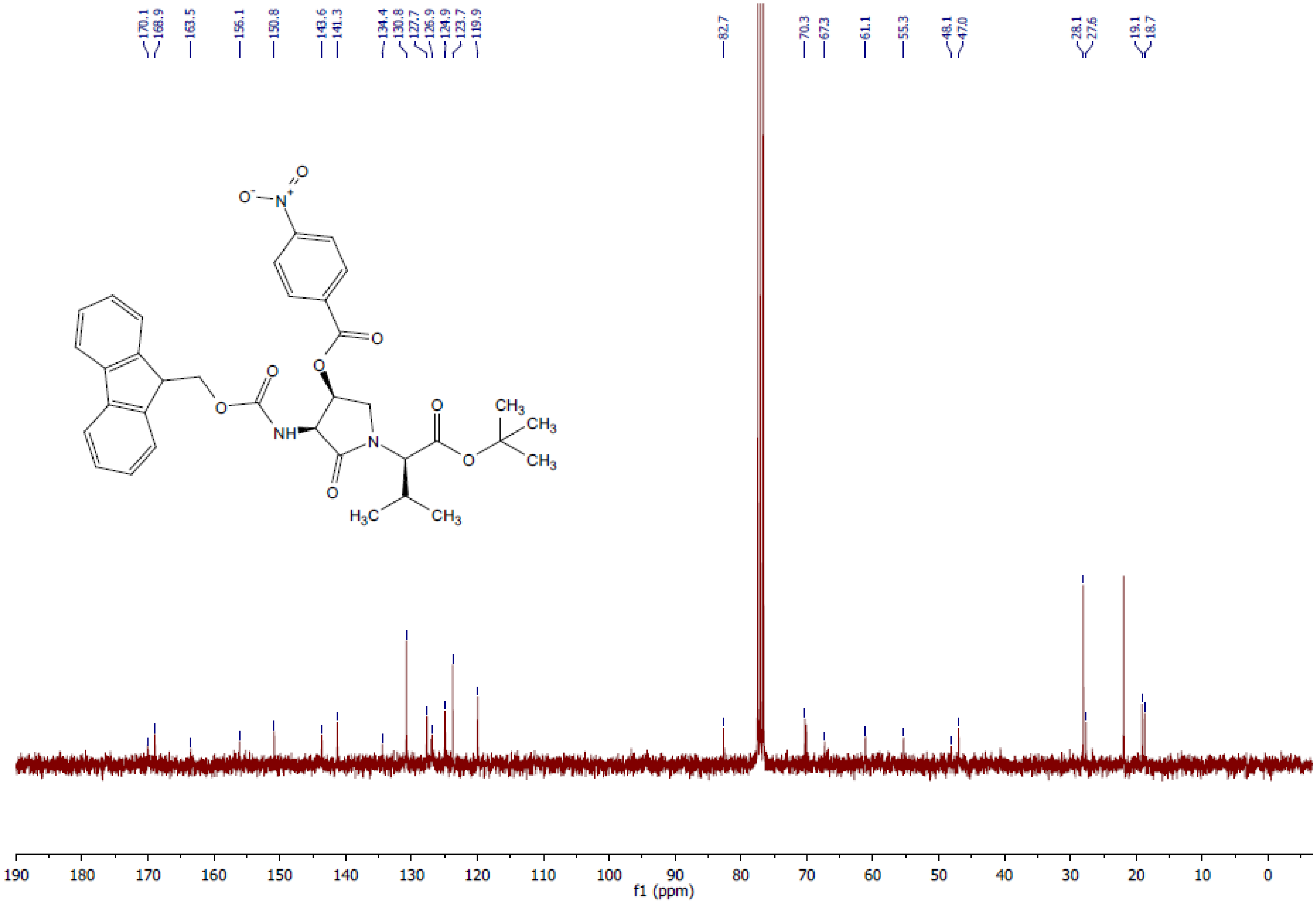

^{1}H NMR (300 MHz, $CDCl_3$) **[(3*S*, 4*S*, 2'*S*)-20]**

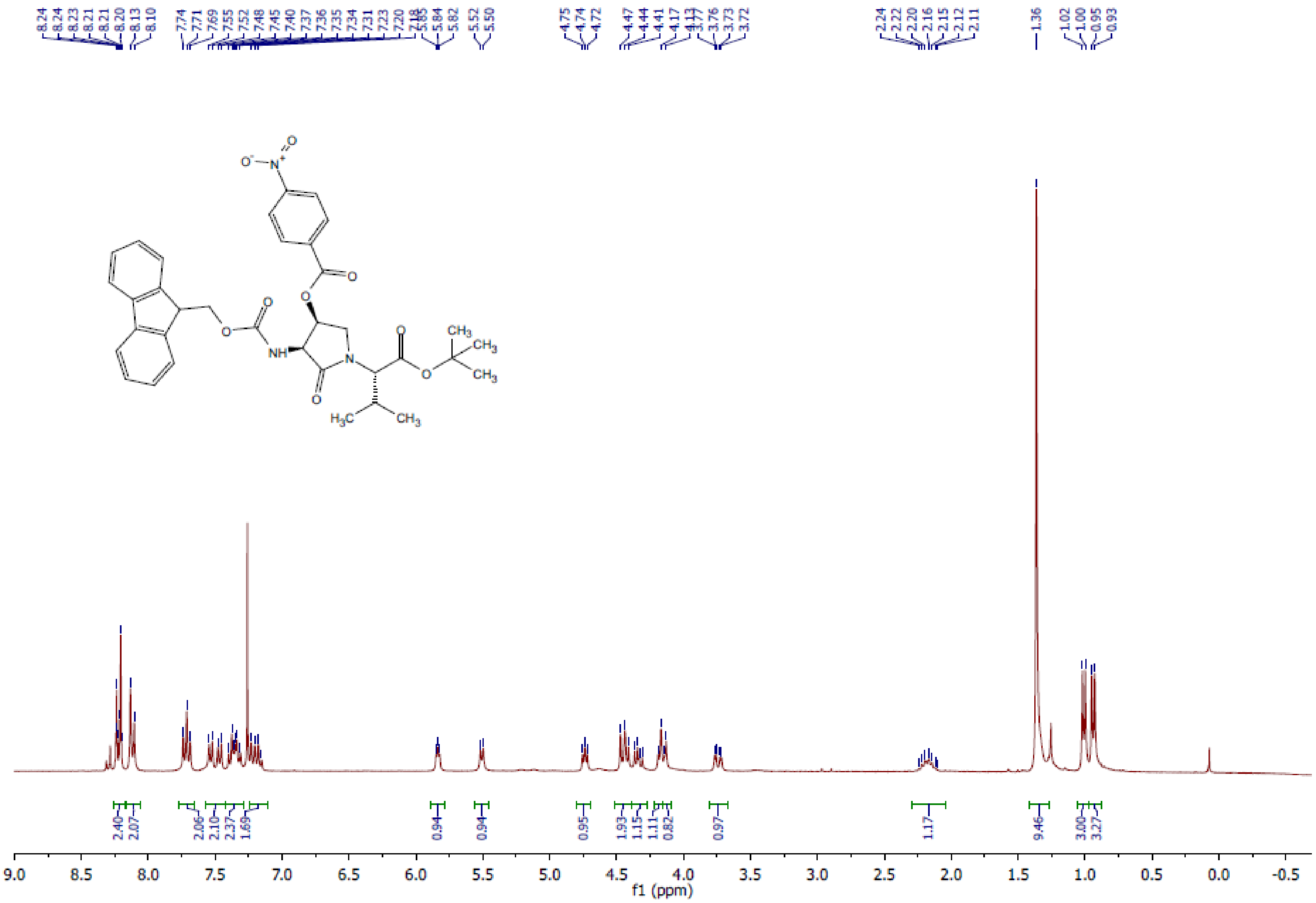

^{13}C NMR (75 MHz, $CDCl_3$) **[(3*S*, 4*S*, 2'*S*)-20]**

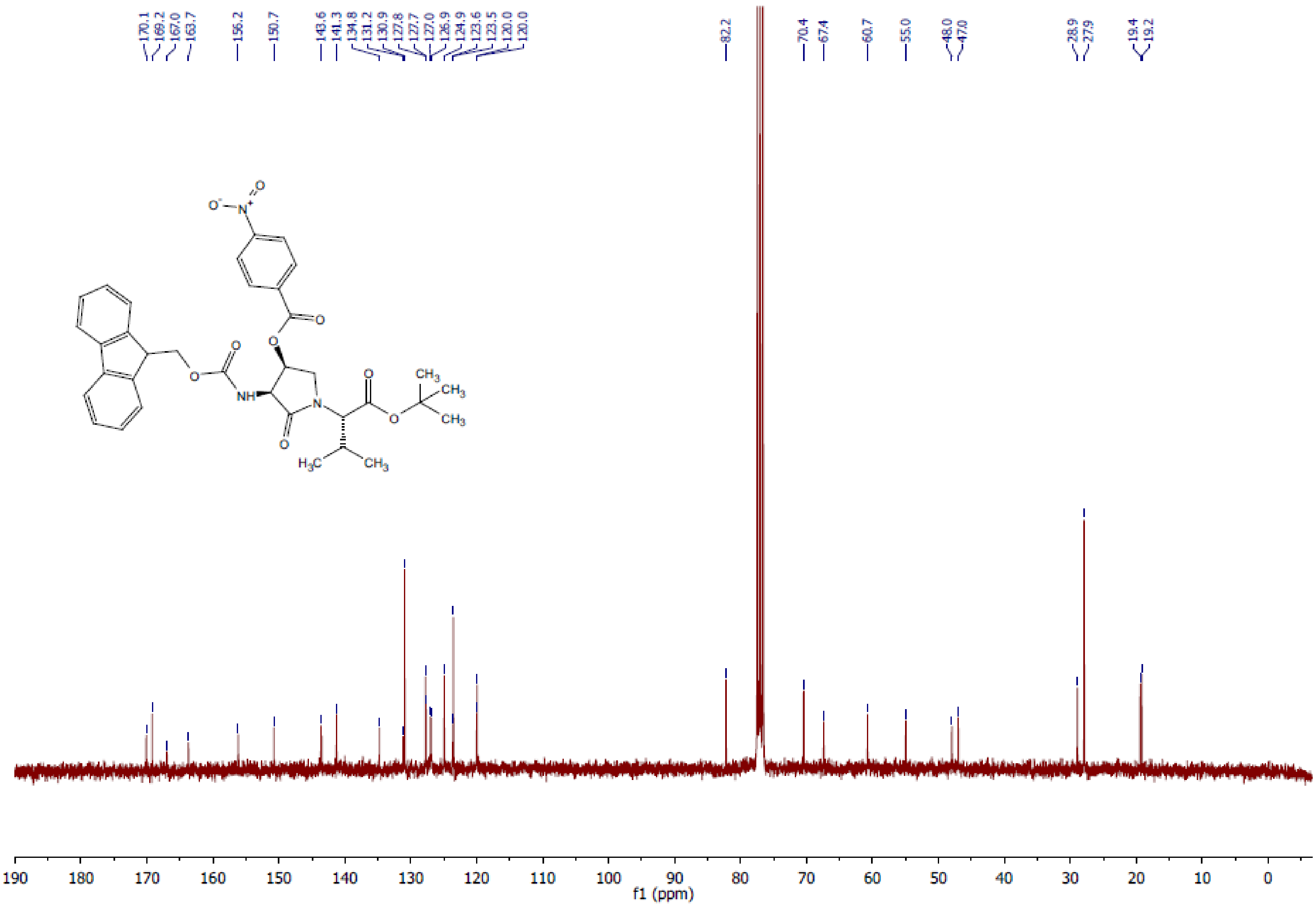
170.1
169.2
167.0
163.7
156.2
150.7
143.6
141.3
134.8
131.2
130.9
127.8
127.7
127.0
126.9
124.9
123.6
123.5
120.0
120.0
82.2
70.4
67.4
60.7
55.0
48.0
47.0
28.9
27.9
19.4
19.2
190 180 170 160 150 140 130 120 110 100 90 80 70 60 50 40 30 20 10 0
f1 (ppm)
