# Supplementary material for: Probing Anti-inflammatory Properties Independent of NF-κB Through Conformational Constraint of Peptide-Based Interleukin-1 Receptor Biased Ligands
Source: Front Chem. 2019 Feb 13;7:23. doi: 10.3389/fchem.2019.00023 (PMC6381024; doi:10.3389/fchem.2019.00023)
Supplement: Supplementary file 2 [file Image_1.pdf]

Supplementary Figure

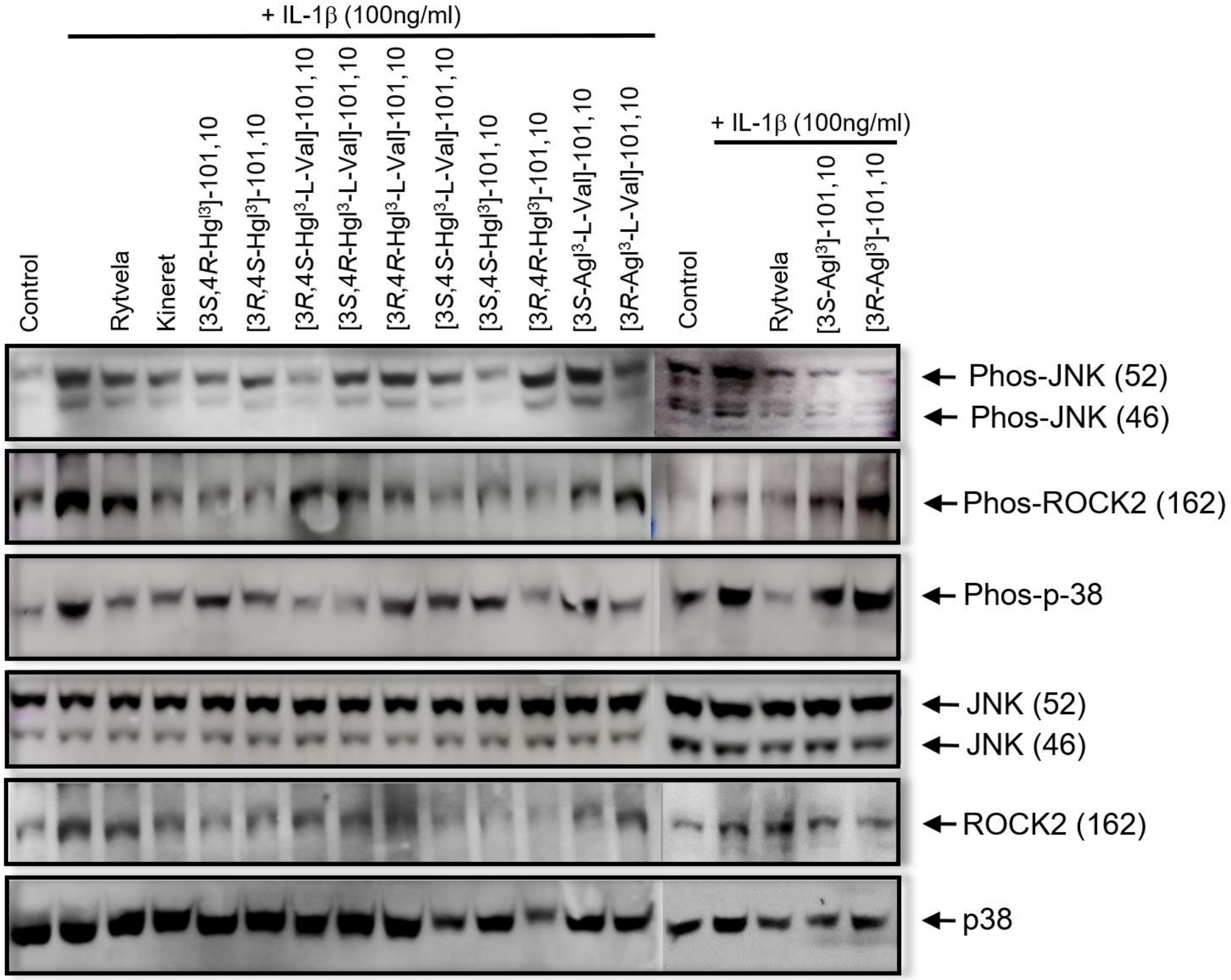

Supplementary Figure 1. Representative Western Blots of the JNK, ROCK2 and p38 proteins. RAW-blue cells were pre-treated with peptides **1, 5** or **6** for 30 min, then stimulated with IL-1β for 15 min. Cell lysates were prepared and lysates (30µg protein per well) were electrophoresed on 12% resolving gels, transferred to PVDF membranes and incubated with antibodies for these proteins and their phosphorylated forms. Numbers in parentheses indicate the molecular weight of the protein of interest in kilo-daltons (kDa).
